# Cortical abnormalities in adults and adolescents with major depression based on brain scans from 20 cohorts worldwide in the ENIGMA Major Depressive Disorder Working Group

**DOI:** 10.1038/mp.2016.60

**Published:** 2016-05-03

**Authors:** L Schmaal, D P Hibar, P G Sämann, G B Hall, B T Baune, N Jahanshad, J W Cheung, T G M van Erp, D Bos, M A Ikram, M W Vernooij, W J Niessen, H Tiemeier, A Hofman, K Wittfeld, H J Grabe, D Janowitz, R Bülow, M Selonke, H Völzke, D Grotegerd, U Dannlowski, V Arolt, N Opel, W Heindel, H Kugel, D Hoehn, M Czisch, B Couvy-Duchesne, M E Rentería, L T Strike, M J Wright, N T Mills, G I de Zubicaray, K L McMahon, S E Medland, N G Martin, N A Gillespie, R Goya-Maldonado, O Gruber, B Krämer, S N Hatton, J Lagopoulos, I B Hickie, T Frodl, A Carballedo, E M Frey, L S van Velzen, B W J H Penninx, M-J van Tol, N J van der Wee, C G Davey, B J Harrison, B Mwangi, B Cao, J C Soares, I M Veer, H Walter, D Schoepf, B Zurowski, C Konrad, E Schramm, C Normann, K Schnell, M D Sacchet, I H Gotlib, G M MacQueen, B R Godlewska, T Nickson, A M McIntosh, M Papmeyer, H C Whalley, J Hall, J E Sussmann, M Li, M Walter, L Aftanas, I Brack, N A Bokhan, P M Thompson, D J Veltman

**Affiliations:** 1 Department of Psychiatry and Neuroscience Campus Amsterdam, VU University Medical Center, Amsterdam, The Netherlands; 2 Imaging Genetics Center, Mark and Mary Stevens Neuroimaging and Informatics Institute, Keck School of Medicine of USC, Marina del Rey, CA, USA; 3 Neuroimaging Core Unit, Max Planck Institute of Psychiatry, Munich, Germany; 4 Department of Psychology, Neuroscience and Behaviour, McMaster University, Hamilton, ON, Canada; 5 Discipline of Psychiatry, University of Adelaide, Adelaide, SA, Australia; 6 Department of Psychiatry and Human Behavior, University of California, Irvine, CA, USA; 7 Department of Radiology, Erasmus MC University Medical Center, Rotterdam, The Netherlands; 8 Department of Epidemiology, Erasmus MC University Medical Center, Rotterdam, The Netherlands; 9 Department of Neurology, Erasmus MC University Medical Center, Rotterdam, The Netherlands; 10 Department of Medical Informatics, Erasmus MC University Medical Center, Rotterdam, The Netherlands; 11 Faculty of Applied Sciences, Delft University of Technology, Delft, The Netherlands; 12 Department of Child and Adolescent Psychiatry, Erasmus University Medical Center-Sophia Children's Hospital, Rotterdam, The Netherlands; 13 German Center for Neurodegenerative Diseases (DZNE), Site Rostock/Greifswald, Germany; 14 Department of Psychiatry and Psychotherapy, University Medicine Greifswald, Greifswald, Germany; 15 Institute for Diagnostic Radiology and Neuroradiology, University Medicine Greifswald, Greifswald, Germany; 16 Institute for Community Medicine, University Medicine Greifswald, Greifswald, Germany; 17 German Center for Cardiovascular Research (DZHK), partner site Griefswald, Greifswald, Germany; 18 German Center for Diabetes Research (DZD), partner site Griefswald, Greifswald, Germany; 19 Department of Psychiatry, University of Muenster, Muenster, Germany; 20 Department of Psychiatry, University of Marburg, Marburg, Germany; 21 Department of Clinical Radiology, University of Muenster, Muenster, Germany; 22 Queensland Brain Institute, The University of Queensland, Brisbane, QLD, Australia; 23 Center for Advanced Imaging, The University of Queensland, Brisbane, QLD, Australia; 24 Queensland Institute of Medical Research Berghofer, Brisbane, QLD, Australia; 25 Institute of Health and Biomedical Innovation, Queensland University of Technology, Brisbane, QLD, Australia; 26 Department of Psychiatry, Virginia Institute for Psychiatric and Behavioral Genetics, Richmond, VA, USA; 27 Centre for Translational Research in Systems Neuroscience and Psychiatry, Department of Psychiatry and Psychotherapy, University Medical Center (UMG), Georg-August-University, Göttingen, Germany; 28 Section for Experimental Psychopathology and Neuroimaging, Department of General Psychiatry, Heidelberg University Hospital, Heidelberg, Germany; 29 Clinical Research Unit, Brain and Mind Centre, University of Sydney, Camperdown, NSW, Australia; 30 Department of Psychiatry and Psychotherapy, Otto von Guericke University, Magdeburg, Germany; 31 Department of Psychiatry and Institute of Neuroscience, Trinity College, Dublin, Ireland; 32 Department of Psychiatry and Psychotherapy, University of Regensburg, Regensburg, Germany; 33 Neuroimaging Center, Section of Cognitive Neuropsychiatry, Department of Neuroscience, University Medical Center Groningen, University of Groningen, Groningen, The Netherlands; 34 Department of Psychiatry and Leiden Institute for Brain and Cognition, Leiden University Medical Center, Leiden, The Netherlands; 35 Orygen, The National Centre of Excellence in Youth Mental Health, Melbourne, VIC, Australia; 36 Centre for Youth Mental Health, The University of Melbourne, Melbourne, VIC, Australia; 37 Melbourne Neuropsychiatry Centre, Department of Psychiatry, The University of Melbourne, Melbourne, VIC, Australia; 38 UT Center of Excellence on Mood Disoders, Department of Psychiatry and Behavioral Sciences, University of Texas Health Science Center at Houston, Houston, TX, USA; 39 Department of Psychiatry and Psychotherapy, Charité Universitätsmedizin Berlin, Berlin, Germany; 40 Department of Psychiatry and Psychotherapy, University of Bonn, Bonn, Germany; 41 Center for Integrative Psychiatry, University of Lübeck, Lübeck, Germany; 42 Department of Psychiatry and Psychotherapy, Agaplesion Diakonieklinikum Rotenburg, Rotenburg, Germany; 43 Department of Psychiatry and Psychotherapy, University Medical Center Freiburg, Freiburg, Germany; 44 Neurosciences Program and Department of Psychology, Stanford University, Stanford, CA, USA; 45 Department of Psychiatry, University of Calgary, Calgary, AB, Canada; 46 University Department of Psychiatry, Warneford Hospital, Oxford, UK; 47 Division of Psychiatry, University of Edinburgh, Royal Edinburgh Hospital, Edinburgh, UK; 48 Centre for Cogntive Ageing and Cogntive Epidemiology, University of Edinburgh, Edinburg, UK; 49 Division of Systems Neuroscience of Psychopathology, Translational Research Center, University Hospital of Psychiatry, University of Bern, Bern, Switzerland; 50 Neuroscience and Mental Health Research Institute, Cardiff University, Cardiff, UK; 51 Department of Psychiatry, NHS Borders, Melrose, UK; 52 Leibniz Institute for Neurobiology, Magdeburg, Germany; 53 Department of Psychiatry, University Tübingen, Tübingen, Germany; 54 Department of Experimental and Clinical Neuroscience, Scientific Research Institute of Physiology and Basic Medicine, Novosibirsk, Russia; 55 Mental Health Research Institute, Tomsk, Russia; 56 Faculty of Psychology, National Research Tomsk State University, Tomsk, Russia; 57 Department of General Medicine, Siberian State Medical University, Tomsk, Russia

## Abstract

The neuro-anatomical substrates of major depressive disorder (MDD) are still not well understood, despite many neuroimaging studies over the past few decades. Here we present the largest ever worldwide study by the ENIGMA (Enhancing Neuro Imaging Genetics through Meta-Analysis) Major Depressive Disorder Working Group on cortical structural alterations in MDD. Structural T1-weighted brain magnetic resonance imaging (MRI) scans from 2148 MDD patients and 7957 healthy controls were analysed with harmonized protocols at 20 sites around the world. To detect consistent effects of MDD and its modulators on cortical thickness and surface area estimates derived from MRI, statistical effects from sites were meta-analysed separately for adults and adolescents. Adults with MDD had thinner cortical gray matter than controls in the orbitofrontal cortex (OFC), anterior and posterior cingulate, insula and temporal lobes (Cohen's *d* effect sizes: −0.10 to −0.14). These effects were most pronounced in first episode and adult-onset patients (>21 years). Compared to matched controls, adolescents with MDD had lower total surface area (but no differences in cortical thickness) and regional reductions in frontal regions (medial OFC and superior frontal gyrus) and primary and higher-order visual, somatosensory and motor areas (*d*: −0.26 to −0.57). The strongest effects were found in recurrent adolescent patients. This highly powered global effort to identify consistent brain abnormalities showed widespread cortical alterations in MDD patients as compared to controls and suggests that MDD may impact brain structure in a highly dynamic way, with different patterns of alterations at different stages of life.

## Introduction

Major depressive disorder (MDD) is the single most common psychiatric disorder, affecting approximately 350 million people each year.^1^ Even so, its pathogenesis and profile of effects in the brain are still not clear. Therefore, in 2013, we initiated the MDD Working Group within the Enhancing Neuro Imaging Genetics through Meta-Analysis (ENIGMA) consortium (http://enigma.ini.usc.edu/) in which researchers around the world collaborate to boost statistical power to elucidate brain abnormalities in MDD. Recently, we reported subcortical volume differences between MDD patients and healthy controls that were related to clinical characteristics, based on data from 8927 individuals using an individual participant data-based meta-analysis approach. Subcortical volume differences were the greatest in the hippocampus, with the strongest effects in recurrent or early-onset patients.^2^ Here we present results on cortical structural differences in an even larger sample (*N*=10 105).

With regard to cortical structural abnormalities in MDD, prior magnetic resonance imaging (MRI) studies, summarized in retrospective meta-analyses of individually published works, mainly implicate the (para)limbic circuitry, including dorsomedial prefrontal cortex (PFC), orbitofrontal cortex (OFC) and (rostral) anterior cingulate cortex (ACC), albeit with large variability across studies.^3, 4, 5, 6, 7^ Findings are inconclusive regarding the temporal and lateral PFC.^4, 8^ Inconsistencies arise owing to differences in scanning and analysis methods, the limited power to detect subtle effects in small samples and clinical variations in medication status,^6^ lifetime disease burden,^6^ age at disease onset^9^ and adult vs adolescent study samples.^10^

Differences in data acquisition protocols and processing and differences in statistical analyses performed are a key source of heterogeneity. For example, different techniques for assessing morphometric deficits in MDD are used. Many studies use automated MRI analyses such as voxel-based morphometry,^11^ which avoid labour-intensive manual tracings and improve reproducibility. Others use surface-based methods that generate detailed maps of cortical thickness and surface area, which may differ in their underlying cellular mechanisms and genetic control.^12^ In addition, retrospective meta-analyses sometimes only include focused or hypothesis-driven studies adopting a region of interest approach (for example, ACC, OFC) with no information on other regions or studies that use coarse or unspecific anatomical regions such as ‘frontal lobe'.^3, 4, 5, 6, 7, 8^ These approaches may not resolve more subtle or localized patterns of effects.

Here we addressed some of these issues by performing the largest coordinated worldwide meta-analysis of cortical structural abnormalities in patients diagnosed with MDD relative to healthy controls. We extracted cortical thickness and surface area estimates in 2148 MDD patients and 7957 healthy individuals using harmonized data analysis strategies across all sites.^13^ Compared to healthy controls, adult MDD studies generally report cortical thinning, but adolescent MDD studies have reported both cortical thinning and thickening^14, 15, 16, 17^ during mid-to-late adolescence. These apparent differences prompted us to analyse adolescent and adult patients separately, with adults defined here as individuals aged >21 years. We set the age cut-off for adult versus adolescent analyses at ⩽21 years, based on 1) evidence of accelerated cortical thinning followed by decelerated thinning in young adulthood during normal brain development^18^ and 2) the presence of a positive correlation between depressive symptoms and ventromedial PFC in individuals with MDD up to 22 years old.^19^ Additional stratifying variables were single vs recurrent episodes, antidepressant medication use, index episode severity and, in the adult sample, adolescent- vs adult-onset.

## Materials and Methods

### Samples

The ENIGMA MDD Working Group currently includes 20 international groups with neuroimaging and clinical data from MDD patients and healthy controls (participating sites are mapped in Supplementary Figure S1). Overall, we analysed data from 10 105 people, including 2148 MDD patients and 7957 healthy controls. Each sample's demographics are detailed in Supplementary Table S1 and clinical characteristics in Supplementary Table S2. Supplementary Table S3 lists exclusion criteria for study enrolment. All participating sites obtained approval from local institutional review boards and ethics committees, and all study participants provided written informed consent.

### Image processing and analysis

Structural T1-weighted MRI brain scans were acquired at each site and analysed locally using harmonized analysis and quality-control protocols from the ENIGMA consortium; in this case, all cortical parcellations were performed with the freely available and validated segmentation software FreeSurfer (versions 5.1 and 5.3).^20^ Image acquisition parameters and software descriptions are given in Supplementary Table S4. Segmentations of 68 (34 left and 34 right) cortical gray matter regions based on the Desikan–Killiany atlas^21^ and two whole-hemisphere measures were visually inspected and statistically evaluated for outliers following standardized ENIGMA protocols (http://enigma.ini.usc.edu/protocols/imaging-protocols). Further details on image exclusion criteria and quality control may be found in Supplementary Information SI1.

### Statistical framework for meta-analysis

We examined group differences in cortical thickness and surface area between patients and controls within each sample using multiple linear regression models. In the primary analysis, the outcome measures were from each of 70 cortical regions of interest (68 regions and two whole-hemisphere average thickness or total surface area measures). A binary indicator of diagnosis (0=controls, 1=patients) was the predictor of interest. All models were adjusted for age and sex. Additional covariates were included whenever necessary to control for scanner differences within each sample. To ease comparisons with prior work,^2, 22^ effect size estimates were calculated using Cohen's *d* metric computed from the *t*-statistic of the diagnosis indicator variable from the regression models. Similarly, for models testing interactions (that is, sex-by-diagnosis and age-by-diagnosis) a multiplicative predictor was the predictor of interest with the main effect of each predictor included in the model and the effect size was calculated using the same procedure.

To detect potentially different effects of major depression with age, we separately analysed adolescent (age ⩽21 years) and adult participants (>21 years). Within the adolescent and adult divisions, we tested stratified models that split patients based on stage of illness (first episode vs recurrent). Furthermore, we examined associations between symptom severity at the time of scanning using the 17-item Hamilton Depression Rating Scale (HDRS-17)^23^ and the Beck Depression Inventory (BDI-II)^24^ and cortical thickness and surface area. Within the adult division, we stratified patients based on age at illness onset (adolescent-onset ⩽21 years; adult-onset >21 years^25^). Results of models that split patients based on antidepressant use at the time of their scan are reported in Supplementary Information SI1. Included samples and total sample sizes for each model are listed in the tables in 'Results' section. Throughout the manuscript, we report *P*-values corrected for multiple comparisons using the Benjamini–Hochberg procedure^26^ to ensure a false-discovery rate (FDR) limited at 5% for 70 measures (34 left hemisphere regions, 34 right hemisphere regions and 2 full-hemisphere measures, for left and right).

All regression models and effects size estimates were computed at each site separately and a final Cohen's *d* effect size estimate was obtained using an inverse variance-weighted random-effect meta-analysis model in R (metafor package, version 1.9-118). Only for the meta-analyses on correlation with symptom severity scores and number of episodes in recurrent patients, predictors were treated as continuous variables, so effect sizes were expressed as partial-correlation Pearson's *r* after removing nuisance variables (age, sex, and scan site). The final meta-analysed partial-correlation *r* was estimated with the same inverse variance-weighted random-effect meta-analysis model. See Supplementary Information SI1 for full meta-analysis details.

### Moderator analyses with meta-regression

The methods and results of the moderator analyses, using meta-regression analyses to test whether individual site characteristics explained a significant proportion of the variance in effect sizes across sites in the meta-analyses, are reported in Supplementary Information SI1.

## Results

### Adults

#### Cortical thickness and surface area differences between MDD patients and controls

**Figure 1:**
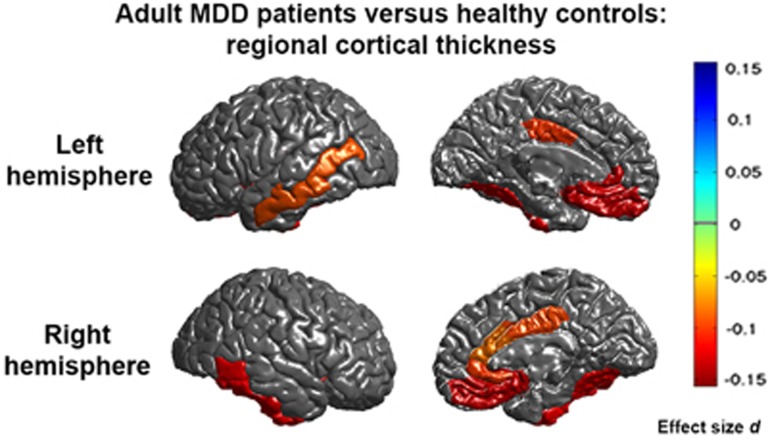
Meta-analysis effect sizes for regions with a significant (*P*_FDR_<0.05) cortical thinning in adult major depressive disorder (MDD) patients compared to healthy controls. Negative effect sizes *d* indicate cortical thinning in MDD compared to controls.

We found significant and consistent thinner cortices in the frontal and temporal lobes of adult depressed patients (*N*=1902) compared to controls (*N*=7658) in the bilateral medial OFC, fusiform gyrus, insula, rostral anterior and posterior cingulate cortex and unilaterally in the left middle temporal gyrus, right inferior temporal gyrus and right caudal ACC (see Figure 1 for significant regions, Supplementary Figure S11 for forest plots and Table 1 for full cortical thickness effects). Regions are listed in all tables in order of effect size, from the strongest to the weakest effect size. None of the regions analysed showed significant differences in cortical surface area (Supplementary Table S19) or evidence of sex-by-diagnosis or age-by-diagnosis interaction effects (Supplementary Tables S5, S6, S20 and S21).

#### First vs recurrent episode adult MDD

Adult patients with recurrent depression (*N*=1302) compared to controls (*N*=7450) revealed cortical thinning in left medial OFC (Supplementary Figures S2 and S12). First-episode patients (*N*=535) compared to controls (*N*=7253) showed more widespread cortical thinning in bilateral fusiform gyrus, rostral ACC and insula and left medial orbitofrontal and superior frontal cortex, right caudal anterior and posterior cingulate cortex and right isthmus cingulate cortex (Supplementary Figures S3 and S13). No differences were detected between recurrent and first-episode patients (Supplementary Table S9). Similar to the overall MDD group analysis, no cortical surface area differences were detected (Supplementary Tables S22–S24), and we found no significant correlations between thickness and surface area and the number of depressive episodes in recurrent patients (*N*=496; Supplementary Table S10).

#### Age of onset in adult MDD

Cortical thinning was observed in patients with an adult age of illness onset (>21 years, *N*=1214) compared to controls (*N*=3329) in bilateral insula, rostral anterior, posterior and isthmus cingulate cortex, fusiform gyrus, medial OFC, right caudal ACC and right inferior temporal gyrus (Supplementary Figures S4 and S14). We did not detect significant differences in cortical thickness in patients with an adolescent age of onset (⩽21 years, *N*=472), compared to controls (*N*=2885), (Supplementary Table S12) and when comparing adolescent-onset and adult-onset patients directly (Supplementary Table S13). Similarly, no surface area differences were detected in these subgroup analyses (Supplementary Tables S26–S28).

#### Correlation with symptom severity in adult patients

None of the cortical thickness measurements were correlated with symptom severity at study inclusion using the HDRS-17 (*N*=776) and BDI-II (*N*=943) questionnaires (Supplementary Tables S17 and S18). For surface area measurements, no associations were found with the HDRS-17 (Supplementary Table S32) and weak negative correlations were detected for BDI-II scores and surface area of the bilateral precuneus, left frontal pole and left postcentral gyrus (Supplementary Table S33, Supplementary Figures S7 and S17).

### Adolescents

#### Cortical thickness and surface area differences between adolescent MDD patients and controls

**Figure 2:**
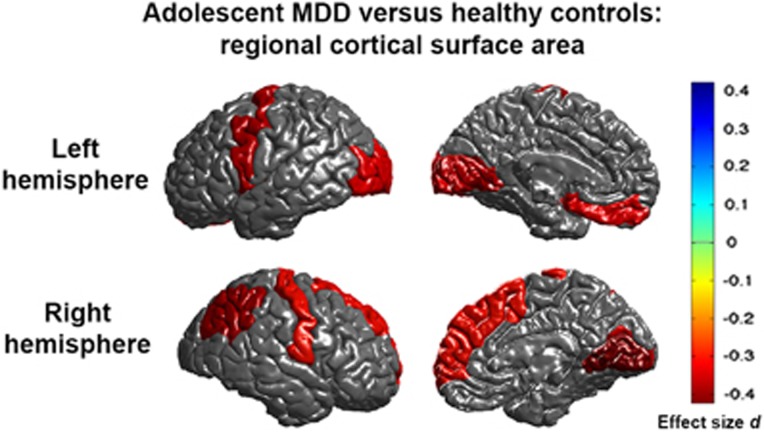
Meta-analysed effect sizes for regions with a significant (*P*_FDR_<0.05) decrease in cortical surface area in adolescent major depressive disorder (MDD) patients compared to healthy controls. Negative effect sizes *d* indicate lower cortical surface area in MDD compared to controls.

Left and right hemisphere total surface area was smaller in depressed adolescent patients (*N*=213) compared to adolescent controls (*N*=294). Regionally, surface area reductions were observed in bilateral lingual gyrus and pericalcarine gyrus, left lateral occipital cortex, left medial OFC, left precentral gyrus, right inferior parietal cortex, right superior frontal gyrus and right postcentral gyrus (see Figure 2 and Supplementary Figure S18 and Table 2 for full tabulation of effects). No cortical thickness differences were detected between adolescent MDD patients and controls (Supplementary Table S34). Further, no cortical regions showed age-by-diagnosis or sex-by-diagnosis interaction effects (Supplementary Tables S35, S36, S45 and S46).

#### First vs recurrent episode adolescent MDD

Adolescents with recurrent depression (*N*=104) showed reductions in left and right hemisphere overall surface area compared to controls (*N*=142). Regionally, surface area reductions were observed in bilateral inferior parietal cortex and caudal middle frontal gyrus and left fusiform gyrus, left lateral occipital cortex, left precuneus, left superior parietal cortex, left medial OFC, right banks of the superior temporal sulcus, right lingual gyrus, right pericalcarine gyrus and right postcentral gyrus (Supplementary Figures S8 and S19, Supplementary Table S48). First-episode patients (*N*=80) showed no detectable differences, when compared to controls (*N*=154) or the recurrent adolescent MDD group (Supplementary Tables S47 and S49). No cortical thickness differences were found in adolescent MDD for first-episode or recurrence subgroups (Supplementary Tables S37–S39); similarly, no correlations with the number of episodes were detected for surface area or thickness in recurrent adolescent MDD patients (Supplementary Tables S40 and S50).

#### Correlations with symptom severity in adolescent MDD

We did not detect significant differences in cortical thickness or surface area when examining the effects of symptom severity at study inclusion using the HDRS-17 (*N*=134) questionnaire (Supplementary Tables S44 and S54), whereas BDI-II scores were available only for a small group of adolescent patients (*N*=31), precluding meaningful comparisons.

### Moderating effects on cortical thickness and surface area

Results of the moderator analyses can be found in the Supplementary Information.

## Discussion

In the largest analysis to date of cortical structural measures, we applied an individual participant data-based meta-analytic approach to brain MRI data from >10 000 people, of whom around one-fifth were affected by MDD. We found significant differences in cortical brain structures in adolescent and adult MDD and specific associations with clinical characteristics.

### Cortical thickness

Adult MDD patients had cortical thickness deficits in 13 (of 68) regions examined. Cortical thinning was generally observed bilaterally, in regions that encompassed the medial PFC, rostral anterior and posterior cingulate cortex, insula and fusiform gyrus. Unilateral effects were observed in left middle temporal gyrus and right inferior temporal and right caudal ACC. Our findings of lower cortical thickness in medial PFC and ACC are consistent with prior meta-analyses.^3, 4, 5, 6, 7, 8^ Our findings extend previous findings by demonstrating structural abnormalities in the temporal lobe (middle and inferior temporal and fusiform gyri), posterior cingulate cortex and insula. The large sample also adds to our understanding of how reproducible and consistent these effects are likely to be when surveying cohorts worldwide.

A key feature of these regions is their close interaction with the limbic system, consistent with the general pathophysiological model of MDD that posits dysfunctional limbic–cortical circuits.^27, 28^ The dorsal and rostral ACC are functionally heterogeneous, supporting task monitoring, conflict detection, emotion regulation, social cognition and executive functions.^29^ The insular cortex is similarly multifunctional and engaged in visceroception, autonomic response regulation and attentional switches (for example, Menon and Uddin^30^). These regions show consistent structural differences in this cross-sectional morphometric study that may contribute to the broad spectrum of emotional, cognitive and behavioural disturbances observed in MDD.

Although effect sizes were relatively small (*d* −0.08 to −0.13, percentage of difference −0.5% to −1.3%, with overall low-to-medium heterogeneity among studies; that is, *I*^2^ for most regions between 0% and 50%) and in the range of previously reported hippocampal volume reduction,^2^ the medial OFC showed the largest effect sizes (*d* −0.13, percentage of difference −1.1%). The lower medial wall of the PFC (medial OFC according to the Desikan–Killiany atlas^21^ in FreeSurfer) contains the subgenual ACC (sgACC), subcallosal gyrus and medial OFC and has dense connections to the hypothalamus as the primary site of stress response regulation.^31^ These findings concur with postmortem findings of OFC structural deficits,^32^ OFC/sgACC-specific volumetric meta-analyses,^8, 33^ correlations between OFC thickness and cortisol levels^34^ and evidence of functional derangement of the sgACC in depression.^35^ Recently, right medial OFC thickness measured at baseline in healthy adolescent girls proved a strong predictor of the onset of depression in a multivariate model.^36^ The ventromedial PFC and OFC (including the sgACC) are critically involved in reinforcement learning,^37^ fear responsiveness and the adaptive control of emotions,^38^ which are disturbed in MDD, and have been associated with both a non-response to therapy^39, 40^ and a more unfavourable course of the illness.^41^ Distinct from our hippocampal volume finding,^2^ these effects were detectable already in first-episode patients with a medial OFC/ACC and insular focus, indifferent from recurrent patients who showed less widespread changes compared to controls. Further, no correlations with the number of episodes and no age-by-diagnosis effects were detected. Although these observations are based on cross-sectional data, we add to limited and conflicting reports of longitudinal volumetric changes in MDD^42, 43^ which suggest that progressive cortical abnormalities with growing disease load does not appear to be a general feature of depression.

With regard to age at onset, no significant differences were found between adult patients with an adolescent-onset (⩽21 years) and controls. In contrast, adult-onset was associated with significant cortical thinning in numerous frontal, cingulate and temporal regions. Interestingly, our prior work^2^ showed hippocampal volume alterations in adolescent-onset but not adult-onset patients. This result may suggest differential effects of stress-related remodelling or interactions with brain maturational mechanisms at different periods of disease onset. Cortical structural deficits were not found in adolescent-onset adult patients. This, however, may in part be due to lower statistical power in the smaller adolescent-onset compared with the adult-onset patient samples (*N*=472 vs *N*=1214). In addition, the lack of effects could perhaps be explained by the fact that adolescent-onset patients were younger than the adult-onset groups. Hence, greater cortical thinning in MDD may be more pronounced in adult-onset patients if the disease effects interact with increased aging of the brain,^40, 44^ but see also Truong *et al.*^9^ Following this logic, we performed a *post-hoc* moderator analysis examining the effects of mean age of patients in each sample on cortical thickness differences between adolescent-onset (adult) patients and controls. Samples with a higher mean age of patients indeed showed greater cortical thinning in the adolescent-onset group compared with controls (Supplementary Figure S22). Though not robust to conservative correction for multiple comparisons (trend-level *P*_FDR_=0.09 for the left medial OFC), this pattern fits the lack of detected thickness differences in our adolescent MDD vs adolescent controls analysis. Prior studies have reported mixed results with regard to cortical abnormalities in adolescent MDD, showing increased,^15, 17^ decreased^14, 15, 16^ or no differences in cortical thickness.^45^ Cortical thickness decreases linearly during adolescence^46, 47, 48, 49^ owing to synaptic pruning, myelination and other remodelling effects.^50^ In adolescent MDD, anxious and depressed symptoms have been associated with greater cortical thickness.^19^ In contrast, our current results and prior reports^3, 4, 5, 6, 7, 8^ provide consistent evidence for cortical thinning in adult MDD. These opposite effects would suggest a delay in maturation (that is, delay in thinning) of cortical thickness in adolescent MDD, resulting in greater cortical thickness during various stages of brain maturation but thinner cortex eventually. A possible explanation for the lack of cortical thickness effects in the current study is that 70% of our adolescents were 18–21 years, perhaps older than the most sensitive period to detect maturation delays.^17, 19^ Of note, although not significant, the left lateral OFC showed a medium effect size (*d* −0.31, percentage of difference −1.9%) for cortical thinning in adolescent MDD compared to controls, whereas cortical alterations in other regions were less clear.

### Cortical surface area

Adult MDD patients showed no surface area abnormalities compared to controls. However, adolescent patients revealed smaller left and right hemisphere total surface areas, reflecting a diffuse pattern of local surface area deficits (effect sizes *d* between −0.31 to −0.42, percentage of difference −3.3 to −5.9%). Similar to cortical thickness alterations in adult MDD we observed surface area deficits in medial OFC and superior frontal gyrus, but also in primary and higher order visual, somatosensory and motor areas. These deficits were observed in recurrent patients, suggesting a negative effect of multiple episodes.

Cortical thinning starts from 2 to 4 years of age and continues across the lifespan, but overall cortical surface area follows a nonlinear and nonmonotonic developmental trajectory. The cortical surface expands until about 12 years, remains relatively stable and then decreases with age.^46, 47, 48, 49^ Development of cortical thickness and surface area are genetically independent^12^ and result from different neurobiological processes,^50^ representing distinct features of cortical development and aging. Cortical surface area abnormalities were not detected in our early-onset adult MDD patients, despite greater statistical power than for the adolescent analyses, so smaller cortical surface area in adolescent MDD may indicate delayed cortical maturation (that is, delayed expansion). Some regions with surface area abnormalities, including medial occipital regions (lingual gyrus), inferior parietal cortex, precentral gyrus, medial OFC and superior frontal gyrus, mature over a more prolonged time course during adolescence^47, 49^ and may be especially prone to a delay in maturation in adolescent MDD. Such delayed maturation may alter functional connections with other regions through decreases in growth and branching of dendritic trees and the number of synapses associated with gray matter volume,^51^ which may persist into adult MDD even if surface area measures normalize when transitioning into adulthood. The absence of cortical surface area abnormalities in the adult MDD patients with an early age of onset of depression could indicate such normalization; importantly, however, we still detected weak negative associations between severity of depressive symptoms and bilateral precuneus, left frontal pole and left postcentral gyrus surface area.

To our knowledge, alterations in cortical surface area abnormalities have not been evaluated in adolescents with MDD. Surface area deficits of the ventromedial PFC and precuneus in children and adolescents have been associated with higher anxiety,^52^ of the lingual and temporal gyri in children with childhood maltreatment,^53^ of prefrontal regions in children experiencing early life adversity^54^ and of the OFC in adolescents with conduct disorder.^55^ Importantly, early life stress, symptoms of anxiety and externalizing problems in childhood and early adolescence are all risk factors for early-onset MDD.^56, 57^

Cortical thickness and surface area abnormalities were mainly observed in first-episode MDD and adolescent MDD, respectively; this may indicate that cortical alterations are a feature of more heterogeneous MDD samples, including adolescent and first-episode adult MDD individuals who may go on to other outcomes, including bipolar or psychotic disorders, instead of adult MDD samples with a more 'pure' depressive phenotype (in our study characterized by recurrent MDD and adult MDD with an adolescent-onset of depression in whom the illness is confirmed over time). Indeed, lower surface areas in many of the same regions we observed in adolescent MDD in the current study were prospectively predictive of poor functional outcomes in young people with a clinically defined risk of developing psychosis.^58^ Similar analyses currently underway in the ENIGMA Schizophrenia and ENIGMA Bipolar Disorder working groups may clarify whether regional cortical surface area and thickness are altered to a greater extent in individuals with schizophrenia and bipolar disorder than the alterations we observed in (adult) MDD. Nonetheless, prospective studies are needed to confirm this heterogeneity hypothesis.

### Limitations

We did not adjust the regional comparisons for average thickness or total surface, respectively, as our main question was directed towards regional MDD-related changes instead of identifying regional effects that exceed a global effect. In contrast to surface area measures, which are highly associated with global measures of the brain (for example, intracranial volume, as a proxy for overall brain size), cortical thickness does not scale proportionately with brain size.^59^ In the current study, global deficits in cortical surface area (indicated by smaller left and right total surface area) were observed in adolescents with MDD. Therefore, our surface area results need to be interpreted as a diffuse, global surface deficit in adolescent MDD, with potential additional regional accentuation.

Furthermore, we used a ⩽21-year cutoff for adolescent vs adult MDD (cf. 'Introduction' section) consistent with our previous work.^2^ Definitions of adolescent MDD in the literature are not consistent, so alternative definitions might yield different results. Ideally, age and age of onset effects on brain abnormalities in MDD should be examined using a dimensional approach. However, in the current meta-analysis the statistical analyses were performed within each site, precluding this approach as few samples covered the entire lifespan. In addition, the age distribution of the adolescents (9% between 12 and 16 years, 21% between 16 and 18 years, 70% ⩾18 years) and the limited adolescent sample size (while larger than prior reports) may not be ideally sensitive to detect age-by-diagnosis interaction and cortical thickness effects. Future addition of more adolescent MDD samples to reflect a balanced age distribution may aid in detecting cortical changes associated with MDD at different stages of brain development.

In addition, when combining already collected data across worldwide samples, data collection protocols are not prospectively harmonized. Imaging acquisition protocols and clinical assessments therefore differed across studies, which limits analysis of sources of heterogeneity. The current study did not allow a reliable investigation of antidepressant medication effects on cortical structure because of its cross-sectional design and lack of detailed information on history, duration and type and dosage of antidepressant treatment. Still, in Supplementary Information SI1 we report on comparisons between patients taking antidepressant, antidepressant-free patients and controls. Adult patients using antidepressants showed robust and widespread effects of cortical thinning, whereas non-users showed cortical thinning only in the left medial OFC. However, this cross-sectional finding should not be interpreted as contradicting generally observed neuroprotective effects of antidepressants.^60^ It is likely confounded by clinical standards recommending antidepressant use mainly for severe or chronic MDD. In adolescent MDD patients, surface area deficits were observed in antidepressant-free patients and not in adolescents taking antidepressants. Clearly, intervention studies with preantidepressant and postantidepressant treatment comparisons of antidepressants are required to draw valid conclusions on the impact of antidepressant use on cortical structure.

## Conclusions

Cortical structure is abnormal in numerous brain regions in adult and adolescent MDD. Medial OFC was consistently implicated across analyses—in adults, adolescents and analyses of clinical correlations. This finding reinforces the hypothesized prominent role of this region in depression throughout life. Other than subcortical volumetric effects, cortical thickness changes were robustly detectable in adult patients at their first episode. MDD may dynamically impact cortical development, and vice versa, with different patterns of alterations at different stages of life. Cortical thickness measurements showed greater differences than surface area measures in adult MDD, but consistent surface area deficits were found in adolescent MDD. Cortical thickness and surface area represent distinct morphometric features of the cortex and may be differentially affected by depression at various stages of life. Future (longitudinal) studies are needed to examine dynamic changes in the cortical regions we examined here and to relate such changes to symptom profiles, outcomes and treatment responses in MDD.

## Figures and Tables

**Table 1:** Full meta-analytic results for thickness of each structure for adult MDD patients vs controls comparison controlling for age, sex and scan center

	*Cohen's* d^a^ *(MDD* vs *CTL)*	*s.e.*	*95% CI*	*% Difference*	P*-value*	*FDR* P*-value*	I^*2*^	*No. of controls*	*No. of patients*
Left medial orbitofrontal cortex	−0.134	0.038	(−0.208 to −0.059)	−1.035	4.35E-04	0.015	23.025	7609	1888
Right medial orbitofrontal cortex	−0.131	0.047	(−0.224 to −0.039)	−1.102	0.006	0.035	46.981	7628	1896
Left rostral anterior cingulate cortex	−0.130	0.044	(−0.216 to −0.044)	−1.264	0.003	0.030	39.185	7656	1896
Right lateral orbitofrontal cortex	−0.120	0.048	(−0.214 to −0.026)	−0.704	0.012	0.058	48.548	7644	1902
Left fusiform gyrus	−0.117	0.030	(−0.176 to −0.058)	−0.576	9.51E-05	0.007	<0.001	7645	1896
Right inferior temporal gyrus	−0.117	0.036	(−0.188 to −0.046)	−0.633	1.29E-03	0.018	18.006	7640	1885
Right fusiform gyrus	−0.116	0.042	(−0.198 to −0.033)	−0.564	0.006	3.47E-02	34.668	7649	1898
Right insula	−0.115	0.041	(−0.195 to −0.035)	−0.624	0.005	0.035	31.271	7651	1895
Left insula	−0.111	0.034	(−0.177 to −0.045)	−0.582	9.34E-04	0.018	10.683	7652	1898
Left isthmus cingulate cortex	−0.104	0.046	(−0.194 to −0.014)	−0.797	0.024	0.100	44.546	7655	1897
Left posterior cingulate cortex	−0.099	0.030	(−0.158 to −0.04)	−0.618	0.001	0.018	0.030	7654	1900
Right rostral anterior cingulate cortex	−0.098	0.034	(−0.165 to −0.031)	−0.993	0.004	0.034	12.104	7651	1899
Right posterior cingulate cortex	−0.093	0.030	(−0.152 to −0.034)	−0.624	0.002	0.022	0.028	7654	1900
Left middle temporal gyrus	−0.090	0.031	(−0.151 to −0.028)	−0.531	0.004	0.034	2.685	7591	1822
Right middle temporal gyrus	−0.088	0.035	(−0.156 to −0.021)	−0.492	0.011	0.053	13.072	7639	1886
Right caudal anterior cingulate cortex	−0.080	0.030	(−0.139 to −0.021)	−0.892	0.008	0.041	<0.001	7655	1898
Right superior frontal gyrus	−0.078	0.034	(−0.145 to −0.011)	−0.379	0.023	0.099	12.509	7649	1900
Right banks superior temporal sulcus	−0.074	0.034	(−0.14 to −0.008)	−0.528	0.028	0.103	9.841	7613	1827
Left pars orbitalis	−0.073	0.044	(−0.158 to 0.012)	−0.533	0.094	0.212	38.655	7653	1900
Left parahippocampal gyrus	−0.072	0.033	(−0.137 to −0.007)	−0.895	0.030	0.103	8.631	7648	1896
Right isthmus cingulate cortex	−0.071	0.038	(−0.146 to 0.004)	−0.563	0.065	0.174	24.975	7651	1897
Right pars orbitalis	−0.070	0.042	(−0.152 to 0.013)	−0.501	0.100	0.212	35.595	7655	1900
Left superior frontal gyrus	−0.066	0.030	(−0.125 to −0.008)	−0.336	0.027	0.103	<0.001	7652	1899
Left inferior parietal cortex	−0.063	0.044	(−0.149 to 0.022)	−0.359	0.147	0.277	38.619	7638	1894
Left pars opercularis	−0.063	0.030	(−0.122 to −0.004)	−0.299	0.037	0.123	0.003	7655	1897
Right frontal pole	−0.062	0.036	(−0.133 to 0.009)	−0.650	0.085	0.204	18.189	7657	1899
Right parahippocampal gyrus	−0.061	0.030	(−0.12 to −0.002)	−0.665	0.042	0.134	0.010	7654	1897
Left banks superior temporal sulcus	−0.058	0.031	(−0.118 to 0.002)	−0.423	0.059	0.173	<0.001	7571	1781
Left hemisphere average thickness	−0.057	0.031	(−0.117 to 0.003)	−0.209	0.065	0.174	2.346	7658	1902
Right entorhinal cortex	−0.055	0.030	(−0.115 to 0.004)	−0.657	0.068	0.177	<0.001	7602	1862
Left pars triangularis	−0.054	0.030	(−0.112 to 0.005)	−0.326	0.074	0.185	<0.001	7651	1897
Right supramarginal gyrus	−0.053	0.044	(−0.139 to 0.032)	−0.273	0.223	0.370	38.371	7633	1874
Right transverse temporal gyrus	−0.051	0.030	(−0.11 to 0.008)	−0.413	0.088	0.204	<0.001	7622	1894
Left inferior temporal gyrus	−0.049	0.035	(−0.117 to 0.019)	−0.273	0.158	0.291	12.727	7630	1872
Right hemisphere average thickness	−0.049	0.033	(−0.113 to 0.015)	−0.179	0.135	0.263	8.061	7658	1902
Left lateral orbitofrontal cortex	−0.046	0.031	(−0.107 to 0.014)	−0.280	0.130	0.260	1.851	7638	1898
Left supramarginal gyrus	−0.045	0.037	(−0.118 to 0.027)	−0.244	0.220	0.370	19.395	7609	1864
Left caudal anterior cingulate cortex	−0.042	0.036	(−0.113 to 0.028)	−0.481	0.240	0.377	17.908	7650	1900
Right inferior parietal cortex	−0.041	0.044	(−0.127 to 0.044)	−0.235	0.343	0.501	39.065	7641	1897
Left entorhinal cortex	−0.041	0.038	(−0.115 to 0.033)	−0.471	0.276	0.420	21.951	7605	1866
Right rostral middle frontal gyrus	−0.038	0.045	(−0.127 to 0.051)	−0.183	0.401	0.561	42.738	7650	1899
Left rostral middle frontal gyrus	−0.037	0.030	(−0.096 to 0.022)	−0.178	0.224	0.370	0.467	7653	1899
Left transverse temporal gyrus	−0.035	0.030	(−0.094 to 0.024)	−0.277	0.243	0.377	<0.001	7635	1895
Right pars triangularis	−0.031	0.046	(−0.122 to 0.059)	−0.179	0.501	0.674	44.727	7645	1897
Right superior temporal gyrus	−0.031	0.030	(−0.09 to 0.029)	−0.184	0.314	0.468	<0.001	7587	1820
Left precuneus	−0.024	0.039	(−0.101 to 0.053)	−0.115	0.541	0.701	27.766	7649	1893
Left lateral occipital cortex	−0.023	0.044	(−0.109 to 0.063)	−0.131	0.605	0.756	39.881	7645	1898
Right precentral gyrus	−0.022	0.040	(−0.101 to 0.057)	−0.132	0.581	0.739	29.869	7643	1894
Left precentral gyrus	−0.020	0.031	(−0.08 to 0.04)	−0.115	0.516	0.681	2.091	7637	1895
Right pars opercularis	−0.017	0.044	(−0.103 to 0.069)	−0.087	0.694	0.823	39.291	7651	1896
Left caudal middle frontal gyrus	−0.014	0.041	(−0.094 to 0.067)	−0.069	0.741	0.844	32.111	7647	1898
Right lingual gyrus	−0.012	0.030	(−0.071 to 0.047)	−0.069	0.692	0.823	<0.001	7641	1894
Left frontal pole	−0.011	0.037	(−0.084 to 0.062)	−0.113	0.772	0.858	21.413	7656	1899
Right paracentral lobule	−0.006	0.030	(−0.064 to 0.053)	−0.031	0.854	0.910	<0.001	7651	1901
Left superior parietal cortex	−0.005	0.040	(−0.084 to 0.074)	−0.026	0.897	0.910	30.628	7645	1896
Left paracentral lobule	−0.003	0.035	(−0.072 to 0.066)	−0.017	0.932	0.932	14.789	7650	1899
Right lateral occipital cortex	0.005	0.033	(−0.06 to 0.071)	0.032	0.871	0.910	9.491	7650	1898
Right precuneus	0.005	0.032	(−0.057 to 0.068)	0.027	0.864	0.910	6.038	7646	1894
Left lingual gyrus	0.006	0.030	(−0.053 to 0.065)	0.034	0.843	0.910	0.003	7640	1895
Right caudal middle frontal gyrus	0.006	0.044	(−0.08 to 0.092)	0.032	0.889	0.910	39.270	7650	1900
Left temporal pole	0.012	0.037	(−0.06 to 0.084)	0.119	0.747	0.844	18.834	7614	1871
Left superior temporal gyrus	0.012	0.031	(−0.048 to 0.072)	0.075	0.687	0.823	0.004	7551	1806
Right temporal pole	0.013	0.038	(−0.062 to 0.088)	0.144	0.731	0.844	23.178	7631	1871
Right postcentral gyrus	0.028	0.030	(−0.031 to 0.087)	0.157	0.355	0.507	<0.001	7642	1897
Right superior parietal cortex	0.032	0.043	(−0.053 to 0.117)	0.159	0.463	0.635	38.069	7645	1895
Left postcentral gyrus	0.036	0.030	(−0.023 to 0.095)	0.200	0.227	0.370	<0.001	7630	1894
Left cuneus	0.047	0.030	(−0.012 to 0.106)	0.288	0.120	0.246	<0.001	7652	1897
Right cuneus	0.049	0.030	(−0.009 to 0.108)	0.303	0.100	0.212	0.005	7654	1896
Right pericalcarine cortex	0.081	0.059	(−0.035 to 0.198)	0.593	0.171	0.307	66.717	7633	1896
Left pericalcarine cortex	0.094	0.049	(−0.003 to 0.191)	0.662	0.057	0.173	51.503	7645	1894

Abbreviations: CI, confidence interval; CTL, controls; FDR, false-discovery rate; MDD, major depressive disorder.

a Adjusted Cohen's *d* is reported.

**Table 2:** Full meta-analytic results for surface area of each structure for adolescent MDD patients vs controls comparison controlling for age, sex and scan center

	*Cohen's* d *(MDD vs CTL)*	*s.e.*	*95% CI*	*% Difference*	P*-value*	*FDR* P*-value*	I^*2*^	*No. of controls*	*No. of patients*
Right lingual gyrus	−0.422	0.108	(−0.633 to −0.211)	−5.870	9.12E-05	0.006	0.004	294	213
Right inferior parietal cortex	−0.384	0.108	(−0.595 to −0.173)	−5.320	3.64E-04	0.013	0.001	293	213
Left precentral gyrus	−0.369	0.108	(−0.581 to −0.157)	−4.071	6.51E-04	0.015	0.005	291	212
Left lingual gyrus	−0.367	0.116	(−0.595 to −0.139)	−5.165	0.002	0.020	11.038	294	213
Right cuneus	−0.353	0.160	(−0.667 to −0.038)	−5.049	0.028	0.103	49.494	292	212
Left pericalcarine cortex	−0.339	0.108	(−0.55 to −0.128)	−5.862	0.002	0.020	0.012	294	213
Left cuneus	−0.336	0.182	(−0.693 to 0.021)	−5.031	0.065	0.162	60.682	294	213
Right pericalcarine cortex	−0.332	0.108	(−0.543 to −0.12)	−5.548	0.002	0.020	0.005	293	212
Left lateral occipital cortex	−0.330	0.108	(−0.541 to −0.119)	−4.253	0.002	0.020	0.002	294	212
Left medial orbitofrontal cortex	−0.329	0.109	(−0.542 to −0.116)	−5.162	0.002	0.020	<0.001	283	210
Right hemisphere total surface area	−0.325	0.108	(−0.536 to −0.114)	−3.386	0.003	0.020	<0.001	294	213
Left hemisphere total surface area	−0.320	0.107	(−0.531 to −0.11)	−3.332	0.003	0.020	<0.001	294	213
Right postcentral gyrus	−0.305	0.108	(−0.517 to −0.094)	−3.651	0.005	0.027	<0.001	289	212
Right superior frontal gyrus	−0.305	0.107	(−0.516 to −0.095)	−3.916	0.005	0.027	<0.001	294	213
Right caudal middle frontal gyrus	−0.288	0.136	(−0.555 to −0.02)	−5.188	0.035	0.116	32.049	294	211
Left precuneus	−0.278	0.107	(−0.488 to −0.068)	−3.540	0.010	0.052	<0.001	294	213
Right banks superior temporal sulcus	−0.266	0.108	(−0.477 to −0.055)	−4.176	0.014	0.068	0.001	293	203
Right medial orbitofrontal cortex	−0.250	0.111	(−0.468 to −0.032)	−3.850	0.025	0.096	3.586	282	211
Left postcentral gyrus	−0.248	0.108	(−0.459 to −0.037)	−2.887	0.021	0.094	<0.001	293	212
Left rostral middle frontal gyrus	−0.247	0.109	(−0.461 to −0.033)	−3.483	0.024	0.096	2.612	294	213
Left caudal middle frontal gyrus	−0.247	0.107	(−0.457 to −0.037)	−4.068	0.021	0.094	<0.001	294	213
Left superior parietal cortex	−0.245	0.167	(−0.573 to 0.083)	−2.954	0.143	0.263	53.595	294	213
Right middle temporal gyrus	−0.227	0.107	(−0.438 to −0.017)	−3.235	0.034	0.116	<0.001	293	206
Left superior frontal gyrus	−0.222	0.107	(−0.433 to −0.012)	−2.722	0.038	0.122	<0.001	293	212
Right superior parietal cortex	−0.221	0.149	(−0.512 to 0.07)	−2.578	0.137	0.260	41.842	293	213
Right rostral middle frontal gyrus	−0.218	0.153	(−0.519 to 0.083)	−3.091	0.155	0.265	45.512	292	213
Right precentral gyrus	−0.213	0.107	(−0.424 to −0.003)	−2.426	0.047	0.142	<0.001	290	213
Left banks superior temporal sulcus	−0.212	0.111	(−0.43 to 0.005)	−3.670	0.056	0.162	0.001	279	190
Left inferior parietal cortex	−0.205	0.111	(−0.422 to 0.013)	−2.937	0.065	0.162	4.975	294	213
Right pars orbitalis	−0.201	0.107	(−0.412 to 0.009)	−3.034	0.061	0.162	<0.001	294	211
Left paracentral gyrus	−0.200	0.107	(−0.41 to 0.011)	−2.797	0.063	0.162	<0.001	294	212
Left fusiform gyrus	−0.196	0.148	(−0.485 to 0.094)	−2.839	0.186	0.280	41.160	293	213
Left frontal pole	−0.194	0.123	(−0.435 to 0.048)	−3.013	0.116	0.243	19.476	294	213
Left caudal anterior cingulate cortex	−0.187	0.151	(−0.484 to 0.11)	−3.527	0.217	0.297	43.982	291	213
Right inferior temporal gyrus	−0.185	0.107	(−0.395 to 0.025)	−3.129	0.084	0.202	<0.001	291	213
Left rostral anterior cingulate cortex	−0.180	0.166	(−0.505 to 0.145)	−3.977	0.277	0.365	53.020	293	213
Right temporal pole	−0.180	0.107	(−0.39 to 0.03)	−2.926	0.093	0.217	<0.001	294	213
Left superior temporal gyrus	−0.179	0.109	(−0.393 to 0.035)	−2.237	0.101	0.221	<0.001	283	198
Right superior temporal gyrus	−0.179	0.108	(−0.39 to 0.032)	−2.104	0.097	0.219	0.588	292	208
Right precuneus	−0.178	0.134	(−0.44 to 0.085)	−2.306	0.184	0.280	30.353	294	213
Right posterior cingulate cortex	−0.175	0.114	(−0.399 to 0.049)	−2.696	0.125	0.243	8.684	293	213
Left pars triangularis	−0.166	0.107	(−0.376 to 0.044)	−2.557	0.122	0.243	0.005	293	213
Right parahippocampal gyrus	−0.165	0.107	(−0.376 to 0.045)	−2.706	0.123	0.243	<0.001	294	212
Right caudal anterior cingulate cortex	−0.155	0.107	(−0.365 to 0.056)	−2.998	0.149	0.263	<0.001	293	213
Right lateral occipital cortex	−0.154	0.107	(−0.364 to 0.056)	−2.035	0.150	0.263	<0.001	294	213
Left middle temporal gyrus	−0.152	0.110	(−0.367 to 0.064)	−2.255	0.168	0.279	<0.001	283	196
Right fusiform gyrus	−0.145	0.107	(−0.355 to 0.066)	−2.157	0.178	0.280	<0.001	294	211
Left supramarginal gyrus	−0.141	0.107	(−0.351 to 0.069)	−2.061	0.187	0.280	<0.001	292	213
Right insula	−0.141	0.107	(−0.352 to 0.069)	−1.930	0.188	0.280	<0.001	292	213
Right paracentral gyrus	−0.140	0.107	(−0.351 to 0.071)	−1.967	0.192	0.280	<0.001	291	213
Left entorhinal cortex	−0.137	0.108	(−0.348 to 0.074)	−3.056	0.202	0.289	<0.001	292	209
Right pars triangularis	−0.135	0.107	(−0.345 to 0.075)	−2.189	0.207	0.290	<0.001	293	213
Left temporal pole	−0.131	0.107	(−0.34 to 0.079)	−2.086	0.222	0.298	<0.001	294	213
Left inferior temporal gyrus	−0.110	0.108	(−0.322 to 0.101)	−1.824	0.305	0.396	<0.001	290	212
Right rostral anterior cingulate cortex	−0.110	0.136	(−0.376 to 0.156)	−2.501	0.418	0.504	31.569	292	212
Right isthmus cingulate cortex	−0.110	0.118	(−0.341 to 0.121)	−1.774	0.350	0.437	13.248	293	213
Left transverse temporal gyrus	−0.100	0.107	(−0.311 to 0.11)	−1.660	0.349	0.437	0.002	294	213
Left parahippocampal gyrus	−0.099	0.113	(−0.321 to 0.123)	−2.112	0.383	0.470	7.948	294	211
Left posterior cingulate cortex	−0.085	0.172	(−0.422 to 0.253)	−1.246	0.624	0.704	56.442	293	213
Left pars opercularis	−0.075	0.107	(−0.286 to 0.135)	−1.208	0.485	0.575	<0.001	293	211
Right lateral orbitofrontal cortex	−0.065	0.124	(−0.309 to 0.178)	−0.917	0.600	0.689	20.640	294	211
Right transverse temporal gyrus	−0.060	0.107	(−0.27 to 0.149)	−1.000	0.572	0.668	<0.001	294	213
Left pars orbitalis	−0.051	0.139	(−0.324 to 0.221)	−0.774	0.712	0.791	34.678	292	213
Left insula	−0.036	0.154	(−0.337 to 0.265)	−0.405	0.815	0.852	45.762	291	213
Right pars opercularis	−0.031	0.107	(−0.241 to 0.179)	−0.495	0.773	0.845	<0.001	292	213
Right supramarginal gyrus	−0.029	0.108	(−0.24 to 0.182)	−0.413	0.789	0.849	<0.001	288	211
Right entorhinal cortex	−0.021	0.109	(−0.235 to 0.193)	−0.462	0.847	0.872	0.001	291	209
Left lateral orbitofrontal cortex	0.010	0.180	(−0.342 to 0.363)	0.152	0.955	0.955	60.017	294	213
Left isthmus cingulate cortex	0.016	0.121	(−0.221 to 0.253)	0.266	0.896	0.909	16.747	293	212
Right frontal pole	0.064	0.258	(−0.441 to 0.569)	0.989	0.803	0.851	80.174	294	213

Abbreviations: CI, confidence interval; CTL, controls; FDR, false-discovery rate; MDD, major depressive disorder.

Adjusted Cohen's *d* is reported.

## Supplementary Material

Supplementary Informations
