# Supplementary material for: Cortical abnormalities in adults and adolescents with major depression based on brain scans from 20 cohorts worldwide in the ENIGMA Major Depressive Disorder Working Group
Source: Mol Psychiatry. 2016 May 3;22(6):900–9. doi: 10.1038/mp.2016.60 (PMC5444023; doi:10.1038/mp.2016.60)
Supplement: Supplementary Informations [file mp201660x1.docx]

Supplementary Information

**Supplementary Information SI1:**

**Image Exclusion Criteria:**

Each image segmentation was individually examined by a neuroimaging expert at each site by overlaying the segmentation label of each structure on the T1-weighted brain scan. Further, we collected study-wide statistics (means and standard deviations) as well as histogram plots in order to identify non-normally distributed data and major outliers. A subject was considered a statistical outlier if its volume was >2.698 standard deviations away from the global mean. For each subject that was marked as a statistical outlier, individual sites were asked to re-inspect the subject’s segmentation in order to verify that it was properly segmented. If a subject was a statistical outlier, but was properly segmented it was kept in the analysis. Otherwise the subject was removed.

**Additional Meta-analysis Details:**

Using this meta-analytical framework we were able to combine data from multiple sites and weigh individual effect size estimates by level of precision. All meta-analysis models were fit using the restricted maximum likelihood method (REML^1^). Percent differences were calculated for each effect size difference in order to restate the difference in terms of percent change in brain volume. Percent difference is calculated such that (% Diff)_trait_ = (Cohen’s d_trait_ x std dev._trait_)/mean_trait_ and then multiplied by 100 for each trait. The mean and standard deviation for each trait used in the percent difference calculation are taken from the 4,228 healthy controls in the Rotterdam Study. In addition to meta-analyzed Cohen’s *d* effect size estimates and percent differences, we calculated heterogeneity scores (I^2^) for each structure, which provide the percent of the total variance in effect size that can be explained by heterogeneity alone^2^. Lower values of I^2^ indicate lower variance in the effect size estimation across studies.

**Results with regard to antidepressant medication use**

*Subgroup analyses of adult MDD regarding antidepressant use*

Antidepressant users showed thinning of left and right hemisphere average thickness (N=951) compared to controls (N=7,223). Regionally, bilateral effects (i.e. for both left and right hemisphere) were found in the fusiform gyrus, insula, medial orbitofrontal cortex, middle temporal gyrus, pars orbitalis, posterior cingulate cortex, rostral anterior cingulate cortex and superior frontal gyrus, left pars opercularis, left pars triangularis, right transverse temporal gyrus, right caudal anterior cingulate cortex, right banks of the superior temporal sulcus, right supramarginal gyrus, right isthmus cingulate cortex and right inferior temporal gyrus (**Figure S5 and S15**). Patients not taking antidepressants (N=916) demonstrated lower thickness of the left medial orbitofrontal cortex (**Figure S6 and S16**), yet, no differences were found in the direct comparison between antidepressant and non-antidepressant users (**Table S16**). No surface area effects were detected in antidepressant-related subgroup analyses (**Tables S29-S31**).

*Subgroup analyses of adolescent MDD regarding antidepressant use*

Adolescent antidepressant users (N=82) showed no surface area differences compared with control subjects (N=142) (**Table S51**). Adolescent patients not taking antidepressants (N=119) showed reduced surface area in left lateral occipital cortex, left precentral gyrus, left pericalcarine gyrus, left medial orbitofrontal cortex, right lingual gyrus, right postcentral gyrus and right inferior parietal cortex (**Figure S9 and S20, Table S52**). Adolescent antidepressant users compared to non-users showed larger cortical surface area in the right precentral gyrus and right precuneus (**Figure S10 and S21, Table S53**). None of the regions analyzed showed evidence of significant differences in cortical thickness for tests involving antidepressant medication (**Tables S41-S43**).

**Moderator analyses**

*Methods*

We used meta-regression analyses to test whether field strength of MR images, voxel size, FreeSurfer version used for image processing, percentage of patients acutely depressed, percentage of patients with a co-occurring anxiety disorder, percentage of patients taking atypical antipsychotics, and type of patient (i.e. patients from general population, outpatients, or hospitalized patients) explained a significant proportion of the variance in effect sizes across sites in the meta-analysis for MDD vs. healthy controls (including covariates), separate for adult and adolescent samples. Each moderator variable was separately included as a fixed effect predictor in a meta-regression model. A significance threshold for each moderator hypothesis was determined by false discovery rate (FDR) procedure at q=0.05.

*Results*

Sample characteristics including voxel size, FreeSurfer version used for image processing, percentage of patients acutely depressed, percentage of patients with a co-occurring anxiety disorder, percentage of patients taking antipsychotics, and type of patient (i.e. general population, outpatient, or hospital patient) did not significantly moderate effect size estimates of cortical thickness or surface area differences in adult or adolescent depressed patients compared to controls (**Tables S55-S58**). We found evidence of a significant moderating effect of imaging field strength indicating greater cortical thinning of the right caudal MFG (d=0.167, P=0.019) and left and right precentral gyrus (d=0.109, P=0.019; d=0.152, P=0.019) in adult MDD compared to controls in samples with a higher field strength (**Table S55**).

**Power Analysis**

We performed *post hoc* power analysis to estimate the sample sizes required to replicate the effects observed in this study. Sample size estimates are the number of subjects required in each group (in a case-control comparison) to detect an effect with 80% power at a nominal significance level (*P*=0.05) for a two-sided t-test assuming unequal variance. All power estimates were obtained using the *pwr* package (version 1.1.1) in R.

With 2,140 MDD patients and 8,031 controls we were able to detect cortical differences as small as Cohen’s d=0.0682 at a nominal significance level P-value=0.05 and 80% power (and Cohen’s d=0.103 at a Bonferroni significance threshold for 70 tests P=0.05/70=7.14x10-4). When focusing on the adult samples (1,927 patients and 7,730 controls) we were able to detect cortical differences as small as Cohen’s d=0.0713 at a nominal significance level P-value=0.05 and 80% power (and Cohen’s d=0.108 at a Bonferroni significance threshold for 70 tests P=7.14x10-4). When focusing on the adolescent samples (213 patients and 301 controls) we were able to detect cortical differences as small as Cohen’s d=0.251 at a nominal significance level P-value=0.05 and 80% power (and Cohen’s d=0.380 at a Bonferroni significance threshold for 70 tests P=7.14x10-4).

References

1. Harville DA (1977) Maximum Likelihood Approaches to Variance Component Estimation and to Related Problems. *Journal of the American Statistical Association* 72(358):320-338.

2. Higgins JP & Thompson SG (2002) Quantifying heterogeneity in a meta-analysis. *Statistics in medicine* 21(11):1539-1558.

**Supplementary Information SI2:**

**Acknowledgements:**

The **ENIGMA-Major Depressive Disorder working** group gratefully acknowledges support from the NIH Big Data to Knowledge (BD2K) award (U54 EB020403 to Paul Thompson).

**NESDA:** The infrastructure for the NESDA study (www.nesda.nl) is funded through the Geestkracht program of the Netherlands Organisation for Health Research and Development (Zon-Mw, grant number 10-000-1002) and is supported by participating universities (VU University Medical Center, GGZ inGeest, Arkin, Leiden University Medical Center, GGZ Rivierduinen, University Medical Center Groningen) and mental health care organizations, see [www.nesda.nl](http://www.nesda.nl). Lianne Schmaal is supported by The Netherlands Brain Foundation Grant number F2014(1)-24 and the Neuroscience Campus Amsterdam grant (IPB-SE-15-PSYCH-Schmaal).

**QTIM:** QTIM was funded by the Australian National Health and Medical Research Council (Project Grants No. 496682 and 1009064) and US National Institute of Child Health and Human Development (RO1HD050735). Baptiste Couvy-Duchesne and Lachlan Strike are supported by a PhD scholarship from the University of Queensland. We are grateful to the twins for their generosity of time and willingness to participate in our study. We also thank the many research assistants, radiographers, and other staff at QIMR Berghofer Medical Research Institute and the Centre for Advanced Imaging, University of Queensland.

**MMDP 3T:** Ontario Mental Health Foundation.

**Bipolar Family Study:** The Bipolar Family Study received funding from the European Union‘s Seventh Framework Programme for research under grant agreement n°602450. This study is also supported by Wellcome Trust award 104036/Z/14/Z.

**CODE:** The CODE cohort was collected from studies funded by Lundbeck and the German Research Foundation (WA 1539/4-1, SCHN 1204/3-1). Elizabeth Schramm is supported by a Grant of the Deutsche Forschungsgemeinschaft / German Research Association (SCHR 443/11-1).

**MPIP:** The MPIP Munich Morphometry Sample comprises patients included in Munich Antidepressant Response Signature study and the Recurrent Unipolar Depression (RUD) Case-Control study, and control subjects acquired at the Ludwig-Maximilians-University, Munich, Department of Psychiatry. We wish to acknowledge Rosa Schirmer, Elke Schreiter, Reinhold Borschke and Ines Eidner for image acquisition and data preparation, and Benno Pütz, Nazanin Karbalai, Darina Czamara, Till Andlauer and Bertram Müller-Myhsok for distributed computing support. We thank Dorothee P. Auer for initiation of the RUD study. The MARS study is supported by a grant of the Exzellenz-Stiftung of the Max Planck Society. This work has also been funded by the Federal Ministry of Education and Research (BMBF) in the framework of the National Genome Research Network (NGFN), FKZ 01GS0481.

**SHIP:** SHIP: The Study of Health in Pomerania (SHIP) is supported by the German Federal Ministry of Education and Research (grants 01ZZ9603, 01ZZ0103 and 01ZZ0403) the Ministry of Cultural Affairs as well as the Social Ministry of the Federal State of Mecklenburg-West Pomerania. MRI scans were supported by Siemens Healthcare, Erlangen, Germany. SHIP-LEGEND was supported by the German Research Foundation (GR1912/5-1).

**Rotterdam Study:** The Rotterdam Study is supported by the Erasmus MC and Erasmus University Rotterdam; Netherlands Organisation for Scientific Research (NWO); Netherlands Organisation for Health Research and Development (ZonMW); Research Institute for Diseases in the Elderly (RIDE); Netherlands Genomics Initiative; Ministry of Education, Culture and Science; Ministry of Health, Welfare and Sports; European Commission (DG XII); and Municipality of Rotterdam.

**Muenster Cohort:** The Muenster Neuroimaging Cohort was supported by grants from the German Research Foundation (DFG; grant FOR 2107; DA1151/5-1 to UD) and Innovative Medizinische Forschung (IMF) of the Medical Faculty of Münster (DA120903 to UD, DA111107 to UD, and DA211012 to UD).

**Stanford**: NIMH Grant R01MH59259 to Ian Gotlib, and the National Science Foundation Integrative Graduate Education and Research Traineeship (NSF IGERT) Recipient Award 0801700 and National Science Foundation Graduate Research Fellowship Program (NSF GRFP) DGE-1147470 to Matthew Sacchet.

**Melbourne:** The study was funded by National Health and Medical Research Council of Australia (NHMRC) Project Grants 1064643 (PI Harrison) and 1024570 (PI Davey).

**Houston:** Supported in part by NIMH grant R01 085667, The Dunn Foundation, and the Pat Rutherford, Jr. Endowed Chair in Psychiatry to JCS.

**Imaging genetics Dublin and Clinical Depression Dublin:** The study was supported by a Science Foundation Ireland (SFI) Stokes Professorship Grant to Thomas Frodl.

**Novosibirsk:** Russian Science Foundation grant #16-15-00128 to Lyubomir Aftanas.

**Sydney:**This study was supported by the following National Health & Medical Research Council funding sources: Program Grant (No. 566529), Centres of Clinical Research Excellence Grant (No. 264611), Australia Fellowship (No. 464914) and Clinical Research Fellowship (No. 402864).

**Conflicts of interest:**

All authors have no conflicts of interest related to this study. Jair Soares has participated in research funded by Forest, Merck, BMS, GSK and has been a speaker for Pfizer and Abbott. Andrew McIntosh has received support from Lilly, Janssen, Pfizer and Saccade Diagnostics. Carsten Konrad received fees for an educational program from Aristo Pharma, Janssen-Cilag, Lilly, MagVenture, Servier, and Trommsdorff as well as travel support and speakers honoraria from Janssen, Lundbeck and Servier. Theo G.M. van Erp has consulted for Roche Pharmaceuticals, Ltd., and has a contract with Otsuka Phamaceutical Co., Ltd (OPCJ). Knut Schnell has consulted for Roche Pharmaceuticals and Servier Pharmaceuticals. Henrik Walter has received a speaker honorarium from Servier. 9987---

**Author contributions:**

Protocol design, quality testing, and meta-analysis: L.S., D.P.H., N.J., J.W.C., T.G.M.V.E.

Data collection, processing, analysis and funding: L.S., D.P.H., P.G.S., G.B.H., B.T.B., N.J., J.W.C., T.G.M.V.E., D.B., M.A.I., M.W.V., W.J.N., H.T., A.H., K.W., H.J.G., D.J., R.B., M.S., H.V., D.G., U.D., V.A., N.O., W.H., H.K., D.H., M.C., B.C-D., M.E.R., L.T.S., M.J.W., N.T.M., G.I.D.Z., K.L.M., S.E.M., N.G.M., N.A.G., R.G-M., O.G., B.K., S.N.H., J.L., I.B.H., T.F., A.C., E.M.F., L.S.V.V., B.W.J.H.P., M-J.V.T., N.J.V.D.W., C.G.D., B.J.H., B.M., B.C., J.C.S., I.M.V., H.W., D.S., B.Z., C.K., E.S., C.N., K.S., M.D.S., I.H.G., G.M.M., B.R.G., T.N., A.M.M., M.P., H.C.W., J.H., J.E.S., M.L., M.W., L.A., I.B., N.B., P.T., D.J.V.

Manuscript preparation: L.S., D.P.H., P.G.S., G.B.H., B.T.B., P.M.T., D.J.V.

All authors contributed edits and approved the content of the manuscript.
