# Supplementary material for: Cortical abnormalities in adults and adolescents with major depression based on brain scans from 20 cohorts worldwide in the ENIGMA Major Depressive Disorder Working Group
Source: Mol Psychiatry. 2016 May 3;22(6):900–9. doi: 10.1038/mp.2016.60 (PMC5444023; doi:10.1038/mp.2016.60)
Supplement: Supplementary Tables [file mp201660x2.docx]

Supplementary Tables

**Content:**

- **Demographic and clinical characteristics:**

**Supplementary Table S1:** ENIGMA - Major Depressive Disorder Working Group Demographics. Age (in years), sex, and MDD patients-control breakdown for participating sites

**Supplementary Table S2**: ENIGMA - Major Depressive Disorder Working Group Clinical characteristics of MDD patients. Percentage of MDD patients using antidepressant medication, percentage of first episode and recurrent episode MDD patients, percentage of acutely depressed and remitted MDD patients, severity of symptoms, and age of onset of MDD breakdown for participating sites

- **Exclusion criteria and image acquisition:**

**Supplementary Table S3**: Instrument for diagnosing Major Depressive Disorder and exclusion criteria by site.

**Supplementary Table S4**: Image acquisition and processing by site.

- **Adult meta-analyses results for thickness:**

**Supplementary Table S5**: Full meta-analytic results for thickness of each structure for the Diagnosis by Sex interaction controlling for age, sex and scan center. Adjusted Cohen's d is reported.

**Supplementary Table S6**: Full meta-analytic results for thickness of each structure for the Diagnosis by Age interaction controlling for age, sex and scan center. Adjusted Cohen's d is reported.

**Supplementary Table S7**: Full meta-analytic results for thickness of each structure for first episode MDD patients versus Controls comparison controlling for age, sex and scan center. Adjusted Cohen's d is reported.

**Supplementary Table S8**: Full meta-analytic results for thickness of each structure for recurrent episode MDD patients versus Controls comparison controlling for age, sex and scan center. Adjusted Cohen's d is reported.

**Supplementary Table S9**: Full meta-analytic results for thickness of each structure for first episode MDD versus recurrent episode MDD patients comparison controlling for age, sex and scan center. Adjusted Cohen's d is reported.

**Supplementary Table S10**: Full meta-analytic results for thickness of each structure for the association with number of episodes in recurrent episode MDD patients controlling for age, sex and scan center. Adjusted Cohen's d is reported.

**Supplementary Table S11**: Full meta-analytic results for thickness of each structure for MDD patients with a late age of onset (>21; LAO) versus Controls comparison controlling for age, sex and scan center. Adjusted Cohen's d is reported.

**Supplementary Table S12**: Full meta-analytic results for thickness of each structure for MDD patients with an early age of onset (≤21; EAO) versus Controls comparison controlling for age, sex and scan center. Adjusted Cohen's d is reported.

**Supplementary Table S13**: Full meta-analytic results for thickness of each structure for MDD patients with an early age of onset (≤21; EAO) versus MDD patients with a late age of onset (≤21; LAO) comparison controlling for age, sex and scan center. Adjusted Cohen's d is reported.

**Supplementary Table S14**: Full meta-analytic results for thickness of each structure for MDD patients taking antidepressants at time of scanning versus Controls comparison controlling for age, sex and scan center. Adjusted Cohen's d is reported.

**Supplementary Table S15**: Full meta-analytic results for thickness of each structure for MDD patients not taking antidepressants at time of scanning versus Controls comparison controlling for age, sex and scan center. Adjusted Cohen's d is reported.

**Supplementary Table S16**: Full meta-analytic results for thickness of each structure for MDD patients taking antidepressants MDD patients not taking antidepressants at time of scanning comparison controlling for age, sex and scan center. Adjusted Cohen's d is reported.

**Supplementary Table S17**: Full meta-analytic results for thickness of each structure associated with severity of symptoms at study inclusion measured by the HDRS-17 controlling for age, sex and scan center. Adjusted Cohen's d is reported.

**Supplementary Table S18**: Full meta-analytic results for thickness of each structure associated with severity of symptoms at study inclusion measured by the BDI-II controlling for age, sex and scan center. Adjusted Cohen's d is reported.

- **Adult meta-analyses results for surface area:**

**Supplementary Table S19**: Full meta-analytic results for surface area of each structure for MDD patients versus Controls comparison controlling for age, sex and scan center. Adjusted Cohen's d is reported.

**Supplementary Table S20**: Full meta-analytic results for surface area of each structure for the Diagnosis by Sex interaction controlling for age, sex and scan center. Adjusted Cohen's d is reported.

**Supplementary Table S21**: Full meta-analytic results for surface area of each structure for the Diagnosis by Age interaction controlling for age, sex and scan center. Adjusted Cohen's d is reported.

**Supplementary Table S22**: Full meta-analytic results for surface area of each structure for first episode MDD patients versus Controls comparison controlling for age, sex and scan center. Adjusted Cohen's d is reported.

**Supplementary Table S23**: Full meta-analytic results for surface area of each structure for recurrent episode MDD patients versus Controls comparison controlling for age, sex and scan center. Adjusted Cohen's d is reported.

**Supplementary Table S24**: Full meta-analytic results for surface area of each structure for first episode MDD versus recurrent episode MDD patients comparison controlling for age, sex and scan center. Adjusted Cohen's d is reported.

**Supplementary Table S25**: Full meta-analytic results for thickness of each structure for the association with number of episodes in recurrent episode MDD patients controlling for age, sex and scan center. Adjusted Cohen's d is reported.

**Supplementary Table S26**: Full meta-analytic results for surface area of each structure for MDD patients with an adult age of onset (>21) versus Controls comparison controlling for age, sex and scan center. Adjusted Cohen's d is reported.

**Supplementary Table S27**: Full meta-analytic results for surface area of each structure for MDD patients with an adolescent age of onset (≤21) versus Controls comparison controlling for age, sex and scan center. Adjusted Cohen's d is reported.

**Supplementary Table S28**: Full meta-analytic results for surface area of each structure for MDD patients with an adolecent age of onset (≤21) versus MDD patients with an adult age of onset (>21) comparison controlling for age, sex and scan center. Adjusted Cohen's d is reported.

**Supplementary Table S29**: Full meta-analytic results for surface area of each structure for MDD patients taking antidepressants at time of scanning versus Controls comparison controlling for age, sex and scan center. Adjusted Cohen's d is reported.

**Supplementary Table S30**: Full meta-analytic results for surface area of each structure for MDD patients not taking antidepressants at time of scanning versus Controls comparison controlling for age, sex and scan center. Adjusted Cohen's d is reported.

**Supplementary Table S31**: Full meta-analytic results for surface area of each structure for MDD patients taking antidepressants MDD patients not taking antidepressants at time of scanning comparison controlling for age, sex and scan center. Adjusted Cohen's d is reported.

**Supplementary Table S32**: Full meta-analytic results for surface area of each structure associated with severity of symptoms at study inclusion measured by the HDRS-17 controlling for age, sex and scan center. Adjusted Cohen's d is reported.

**Supplementary Table S33**: Full meta-analytic results for surface area of each structure associated with severity of symptoms at study inclusion measured by the BDI-II controlling for age, sex and scan center. Adjusted Cohen's d is reported.

- **Adolescent meta-analyses results for thickness:**

**Supplementary Table S34**: Full meta-analytic results for thickness of each structure for MDD patients versus Controls comparison controlling for age, sex and scan center. Adjusted Cohen's d is reported.

**Supplementary Table S35**: Full meta-analytic results for thickness of each structure for the Diagnosis by Sex interaction controlling for age, sex and scan center. Adjusted Cohen's d is reported.

**Supplementary Table S36**: Full meta-analytic results for thickness of each structure for the Diagnosis by Age interaction controlling for age, sex and scan center. Adjusted Cohen's d is reported.

**Supplementary Table S37**: Full meta-analytic results for thickness of each structure for first episode MDD patients versus Controls comparison controlling for age, sex and scan center. Adjusted Cohen's d is reported.

**Supplementary Table S38**: Full meta-analytic results for thickness of each structure for recurrent episode MDD patients versus Controls comparison controlling for age, sex and scan center. Adjusted Cohen's d is reported.

**Supplementary Table S39**: Full meta-analytic results for thickness of each structure for first episode MDD versus recurrent episode MDD patients comparison controlling for age, sex and scan center. Adjusted Cohen's d is reported.

**Supplementary Table S40**: Full meta-analytic results for thickness of each structure for the association with number of episodes in recurrent episode MDD patients controlling for age, sex and scan center. Adjusted Cohen's d is reported.

**Supplementary Table S41**: Full meta-analytic results for thickness of each structure for MDD patients taking antidepressants at time of scanning versus Controls comparison controlling for age, sex and scan center. Adjusted Cohen's d is reported.

**Supplementary Table S42**: Full meta-analytic results for thickness of each structure for MDD patients not taking antidepressants at time of scanning versus Controls comparison controlling for age, sex and scan center. Adjusted Cohen's d is reported.

**Supplementary Table S43**: Full meta-analytic results for thickness of each structure for MDD patients taking antidepressants MDD patients not taking antidepressants at time of scanning comparison controlling for age, sex and scan center. Adjusted Cohen's d is reported.

**Supplementary Table S44**: Full meta-analytic results for thickness of each structure associated with severity of symptoms at study inclusion measured by the HDRS-17 controlling for age, sex and scan center. Adjusted Cohen's d is reported.

- **Adolescent meta-analyses results for surface area:**

**Supplementary Table S45**: Full meta-analytic results for surface area of each structure for the Diagnosis by Sex interaction controlling for age, sex and scan center. Adjusted Cohen's d is reported.

**Supplementary Table S46**: Full meta-analytic results for surface area of each structure for the Diagnosis by Age interaction controlling for age, sex and scan center. Adjusted Cohen's d is reported.

**Supplementary Table S47**: Full meta-analytic results for surface area of each structure for first episode MDD patients versus Controls comparison controlling for age, sex and scan center. Adjusted Cohen's d is reported.

**Supplementary Table S48**: Full meta-analytic results for surface area of each structure for recurrent episode MDD patients versus Controls comparison controlling for age, sex and scan center. Adjusted Cohen's d is reported.

**Supplementary Table S49**: Full meta-analytic results for surface area of each structure for first episode MDD versus recurrent episode MDD patients comparison controlling for age, sex and scan center. Adjusted Cohen's d is reported.

**Supplementary Table S50**: Full meta-analytic results for thickness of each structure for the association with number of episodes in recurrent episode MDD patients controlling for age, sex and scan center. Adjusted Cohen's d is reported.

**Supplementary Table S51**: Full meta-analytic results for surface area of each structure for MDD patients taking antidepressants at time of scanning versus Controls comparison controlling for age, sex and scan center. Adjusted Cohen's d is reported.

**Supplementary Table S52**: Full meta-analytic results for surface area of each structure for MDD patients not taking antidepressants at time of scanning versus Controls comparison controlling for age, sex and scan center. Adjusted Cohen's d is reported.

**Supplementary Table S53**: Full meta-analytic results for surface area of each structure for MDD patients taking antidepressants MDD patients not taking antidepressants at time of scanning comparison controlling for age, sex and scan center. Adjusted Cohen's d is reported.

**Supplementary Table S54**: Full meta-analytic results for surface area of each structure associated with severity of symptoms at study inclusion measured by the HDRS-17 controlling for age, sex and scan center. Adjusted Cohen's d is reported.

- **Moderator Analyses**

**Supplementary Table S55:** Full results from the moderator analyses of field strength of scanner, FreeSurfer version used for processing, voxel size, percent of acute patients, percent of patients with anxiety, percent of patients taking antipsychotics, and type of patient in cortical thickness measures of adult MDD patients and controls.

**Supplementary Table S56:** Full results from the moderator analyses of field strength of scanner, FreeSurfer version used for processing, voxel size, percent of acute patients, percent of patients with anxiety, percent of patients taking antipsychotics, and type of patient in cortical surface area measures of adult MDD patients and controls.

**Supplementary Table S57:** Full results from the moderator analyses of field strength of scanner, FreeSurfer version used for processing, voxel size, percent of acute patients, percent of patients with anxiety, percent of patients taking antipsychotics, and type of patient in cortical thickness measures of adolescent MDD patients and controls.

**Supplementary Table S58:** Full results from the moderator analyses of field strength of scanner, FreeSurfer version used for processing, voxel size, percent of acute patients, percent of patients with anxiety, percent of patients taking antipsychotics, and type of patient in cortical surface area measures of adolescent MDD patients and controls.

**Table S1.** ENIGMA - Major Depressive Disorder Working Group Demographics. Age (in years), sex, and MDD patients-control breakdown for participating sites, separately for adult and adolescent samples.

|  |  | **Adult samples (age>21)** | | | | | | **Adolescent samples (age≤21)** | | | | | |
| --- | --- | --- | --- | --- | --- | --- | --- | --- | --- | --- | --- | --- | --- |
| **Study** | **Sample** | **Age Controls (Mean ± SD)** | **Age MDD**  **(Mean ± SD)** | **% Female Controls** | **% Female MDD** | **Total N Controls** | **Total N MDD** | **Age Controls (Mean ± SD)** | **Age MDD**  **(Mean ± SD)** | **% Female Controls** | **% Female MDD** | **Total N Controls** | **Total N MDD** |
| **1** | **NESDA** | 40.6 ± 9.5 | 38.8 ± 9.5 | 64 | 65 | 64 | 141 |  |  |  |  |  |  |
| **2** | **Imaging Genetics Dublin** | 38.8 ± 12.3 | 41.6 ± 10.8 | 52 | 63 | 46 | 52 |  |  |  |  |  |  |
| **3** | **Clinical Depression Dublin** | 30.8 ± 8.1 | 34.8 ± 7.9 | 44 | 41 | 89 | 32 |  |  |  |  |  |  |
| **4** | **CODE** | 41.9 ± 12.9 | 41.2 ± 11.8 | 56 | 64 | 70 | 101 |  |  |  |  |  |  |
| **5** | **CLING** | 25.8 ± 5.2 | 38.7 ± 10.2 | 58 | 47 | 281 | 43 |  |  |  |  |  |  |
| **6** | **SHIP** | 55.5 ± 12.8 | 53.7 ± 11.7 | 44 | 71 | 448 | 138 |  |  |  |  |  |  |
| **7** | **SHIP-trend** | 50.7 ± 14.3 | 49.2 ± 12.2 | 44 | 65 | 936 | 312 |  |  |  |  |  |  |
| **8** | **Sydney** | 50.6 ± 22.4 | 49.16 ± 12.2 | 54 | 63 | 92 | 123 | 20.1 ± 1.0 | 17.3 ± 2.4 | 77 | 70 | 13 | 90 |
| **9** | **QTIM** | 24.7 ± 1.9 | 24.9 ± 2.1 | 69 | 82 | 164 | 28 | 19.4 ± 1.8 | 19.2 ± 1.6 | 62 | 77 | 140 | 26 |
| **10** | **Rotterdam study** | 64.4 ± 11.0 | 60.5 ± 9.9 | 53 | 71 | 4,228 | 66 |  |  |  |  |  |  |
| **11** | **Bipolar Family Study** | 24.5 ± 1.4 | 24.8 ± 2.1 | 66 | 67 | 44 | 12 |  |  |  |  |  |  |
| **12** | **DepOx** ^a^ | 32.3 ± 10.2 | 33.6 ± 10.2 | 58 | 71 | 26 | 28 |  | 20.4 ± 0.5 |  | 40 |  | 10 |
| **13** | **MPIP** | 49.5 ± 12.8 | 48.6 ± 13.3 | 58 | 56 | 219 | 360 |  |  |  |  |  |  |
| **14** | **MMDP 3T** | 34.0 ± 10.8 | 40.8 ± 11.0 | 61 | 55 | 36 | 38 | 19.4 ± 1.9 | 19.3 ± 1.9 | 67 | 46 | 12 | 13 |
| **15** | **Houston** ^a^ | 39.3 ± 11.9 | 42.7 ± 11.6 | 67 | 70 | 99 | 67 |  | 19.7 ± 1.1 |  | 72 |  | 11 |
| **16** | **Sexpect** | 33.8 ± 7.2 | 39.2 ± 11.1 | 15 | 42 | 20 | 19 |  |  |  |  |  |  |
| **17** | **Melbourne** | 23.2 ± 1.1 | 23.1 ± 1.0 | 52 | 53 | 33 | 17 | 17.8 ± 1.8 | 18.1 ± 2.0 | 54 | 57 | 69 | 67 |
| **18** | **Muenster cohort** | 36.5 ± 11.7 | 39.2 ± 11.2 | 56 | 57 | 692 | 265 | 19.8 ± 1.5 | 19.4 ± 1.4 | 65 | 70 | 60 | 20 |
| **19** | **Novosibirsk** | 43.6 ± 9.1 | 47.7 ± 10.9 | 71 | 81 | 17 | 16 |  |  |  |  |  |  |
| **20** | **Stanford** | 37.9 ± 10.0 | 38.1 ± 9.6 | 61 | 57 | 59 | 53 |  |  |  |  |  |  |
|  | **Combined** |  |  |  |  | **7,663** | **1,911** |  |  |  |  | **294** | **237** |

^a^ Samples only included adolescent MDD (no adolescent controls) and were included in the adolescent meta-analyses on results of regression analysis with number of episodes in recurrent MDD and symptom severity.

**Table S2.** ENIGMA - Major Depressive Disorder Working Group Clinical characteristics of MDD patients. Percentage of MDD patients using antidepressant medication, percentage of first episode and recurrent episode MDD patients, percentage of acutely depressed and remitted MDD patients, percentage of patients with a co-occurring anxiety disorder, age of onset of MDD and severity of symptoms breakdown for participating sites, separately for adult and adolescent samples.

| **Study** | **Sample** | **% Antidepressants users** | **% Antipsychotics users** | **% First episode MDD/Recurrent episode MDD** | **% Acute MDD/ Remitted MDD** | **Age of onset MDD (mean ± SD)** | **% co-occurring anxiety disorder** | **HDRS-17**^a^ **Severity MDD (mean ± SD)** | **BDI-II**^b^ **Severity MDD (mean ± SD)** |
| --- | --- | --- | --- | --- | --- | --- | --- | --- | --- |
|  | **Adult samples** |  |  |  |  |  |  |  |  |
| **1** | **NESDA** | 38 | 0 | 42/58 | 100/0 | 24.9 ± 11.2 | 60 |  |  |
| **2** | **Imaging Genetics Dublin** | 71 | 0 | 15/85 | 100/0 | 25.3 ± 12.8 | 0 | 23.6 ± 5.0 |  |
| **3** | **Clinical Depression Dublin** | 91 | 0 | 45/55 | 100/0 | 31.2 ± 8.6 | 0 | 20.6 ± 5.2 |  |
| **4** | **CODE** | 0 | 0 | 0/100 | 100/0 | NA | 0 |  |  |
| **5** | **CLING** | 93 | 16 | 47/53 | 93/7 | 32.2 ± 9.9 | 19 | 20.3 ± 4.5 | 21.6 ± 10.1 |
| **6** | **SHIP** | 17 | 1 | 56/44 | NA | 38.2 ± 13.2 | 37 |  | 11.7 ± 10.4 |
| **7** | **SHIP-trend** | 17 | 5 | 36/64 | NA | 36.2 ± 14.3 | NA |  | 12.4 ± 8.1 |
| **8** | **Sydney** | 66 | 31 | 22/76 | 12/84 | 32.3 ± 11.1 | 15 | 11.1 ± 7.0 |  |
| **9** | **QTIM** | 18 | 0 | NA | NA | 19.5 ± 4.4 | 36 | NA | NA |
| **10** | **Rotterdam study** | 29 | 21 | 44/56 | 100/0 | NA | 12 | NA | NA |
| **11** | **Bipolar Family Study** | 8 | 1 | NA | NA | 22.8 ± 2.7 | 0 | 3.9 ± 4.0 |  |
| **12** | **DepOx** | 0 | 0 | 46/54 | 100/0 | 28.1 ± 9.3 | 1 | 23.0 ± 4.1 |  |
| **13** | **MPIP** | 85 | 7 | 26/74 | 86/14 | 35.5 ± 14.0 | 14 | 26.6 ± 7.6 | 14.3 ± 10.9 |
| **14** | **MMDP 3T** | 71 | NA | 29/71 | 100/0 | 25.2 ± 11.6 | 13 | 12.5 ± 7.1 |  |
| **15** | **Houston** | 0 | 0 | 21/71 | 70/1 | 23.0 ± 11.0 | 2 | 10.0 ± 7.9 | 19.2 ± 13.5 |
| **16** | **Sexpect** | 100 | 0 | 16/84 | 100/0 | 31.6 ± 11.2 | 20 | 11.8 ± 5.5 | 21.4 ± 12.7 |
| **17** | **Melbourne** | 18 | 0 | 18/76 | 100/0 | 18.5 ± 3.1 | 30 | NA | NA |
| **18** | **Muenster cohort** | 91 | 27 | 22/78 | 92/8 | 30.5 ± 11.7 | 38 | 21.6 ± 7.3 | 24.8 ± 10.4 |
| **19** | **Novosibirsk** | 56 | 24 | 6/94 | 56/44 | 40.6 ± 11.9 | 0 | 26.2 ± 5.1 | NA |
| **20** | **Stanford** | 38 | 0 | 9/87 | 100/0 | 19.9 ± 9.3 | 27 | NA | 25.7 ± 10.1 |
|  | **Adolescent samples** | |  |  |  |  |  |  |  |
| **1** | **Sydney** | 52 | 19 | 34/64 | 25/68 | 13.9 ± 2.4 | 22 | 13.9 ± 6.6 | NA |
| **2** | **QTIM** | 23 | 0 | NA | NA | 17.1 ± 2.7 | 35 | NA | NA |
| **3** | **DepOx** ^c^ | 0 | 0 | 60/40 | 100/0 | 18.7 ± 2.7 | NA | 22.8 ± 5.0 | NA |
| **4** | **MMDP 3T** | 15 | NA | 85/15 | 100/0 | 15.3 ± 3.6 | 8 | 10.1 ± 9.1 | NA |
| **5** | **Houston** ^c^ | 0 | 0 | 36/64 | 82/9 | 13.8 ± 2.0 | NA | 12.5 ± 8.2 | 23.9 ± 18.5 |
| **6** | **Melbourne** | 28 | 0 | 40/52 | 100/0 | 16.2 ± 2.6 | 33 | NA | NA |
| **7** | **Muenster cohort** | 80 | 18 | 50/50 | 85/15 | 16.4 ± 3.2 | 44 | 21.1 ± 7.7 | 29.2 ± 11.2 |

^a^ Measured with the Hamilton Depression Rating Scale (HDRS-17; range: 0-52)

^b^ Measured with the Beck Depression Inventory (BDI-II; range: 0-63)

^c^ Samples only included adolescent MDD (no adolescent controls) and were included in the adolescent meta-analyses on results of regression analysis with number of episodes in recurrent MDD and symptom severity.

**Table S3**: Instrument for diagnosing Major Depressive Disorder and exclusion criteria by site

| **Sample** | **Instrument for diagnosing MDD** | **Exclusion criteria** |
| --- | --- | --- |
| **NESDA** | CIDI interview | MDD subjects: presence of axis-I disorders other than MDD, panic disorder, social anxiety disorder, or generalized anxiety disorder and any use of psychotropic medication other than stable use of SSRIs or infrequent benzodiazepine use (i.e., equivalent to 2 doses of 10 mg of oxazepam 3 times per week or use within 48 hours prior to scanning).  Control subjects: no Axis-I diagnosis, no medication use.  All subjects: presence or history of major internal or neurological disorder, dependence on or recent abuse (past year) of alcohol and/or drugs, hypertension, and general MRI contraindications. |
| **Imaging Genetics Dublin** | SCID-1 interview | MDD subjects: comorbid psychiatric disorders (Axis I or Axis II, other than MDD), Treatment with antipsychotics or mood stabilizers, age <18 or >65,  Control subjects: no Axis-I diagnosis, no medication use.  All subjects: history of neurological or other severe medical illness, head injury or severe substance abuse in their lifetime history and general MRI contraindications. |
| **Clinical Depression Dublin** | SCID-1 interview | MDD subjects: comorbid psychiatric disorders (Axis I or Axis II, other than MDD), Treatment with antipsychotics or mood stabilizers, age <18 or >65,  Control subjects: no Axis-I diagnosis, no medication use.  All subjects: history of neurological or other severe medical illness, head injury or severe substance abuse in their lifetime history and general MRI contraindications. |
| **CODE** | SCID interview | MDD: Presence of any other Axis-1 diagnosis; Acute risk for suicide (in contrast to suicidal ideation); History of psychotic symptoms, bipolar disorder, or dementia; Schizotypal, antisocial or borderline personality disorder; Use of psychotropic medication within two weeks prior to the start of the study; No current psychotherapeutic treatment.  Control subjects: No history of or current Axis-1 or 2 disorders.  All subjects: History of or current neurological disorder or brain injury; Serious medical condition; Severe cognitive impairment; Substance-related abuse or dependence disorder; Use of psychotropic medication; Use of central-acting medication; Pregnancy; General MRI contraindications. |
| **CLING** | ICD-10 interview | MDD subjects: past or actual presence of other axis I diagnoses other than anxiety disorders, alcohol/cannabis abuse and tobacco dependence; neurological or other medical conditions that could be related to affective symptoms  Control subjects: no medical history, including neurological and psychiatric history, as well as no previous or actual use of psychotropic medication |
| **SHIP** | M-CIDI interview | MDD subjects: presence of axis-I disorders other than MDD, anxiety disorders, conversion, somatization and eating disorder.  Control subjects: no lifetime diagnosis of depression, no antidepressiva, and severity index=0  All subjects: We removed subjects with medical conditions (e.g. a history of cerebral tumor, stroke, Parkinson’s diseases, multiple sclerosis, epilepsy, hydrocephalus, enlarged ventricles, pathological lesions) or due to technical reasons (e.g. severe movement artifacts or inhomogeneity of the magnetic field). |
| **SHIP-trend** | M-CIDI interview | MDD subjects: no special exclusion criteria  Control subjects: no lifetime diagnosis of depression, no antidepressiva, and severity index=0  All subjects: We removed subjects with due to medical conditions (e.g. a history of cerebral tumor, stroke, Parkinson’s diseases, multiple sclerosis, epilepsy, hydrocephalus, enlarged ventricles, pathological lesions) or due to technical reasons (e.g. severe movement artifacts or inhomogeneity of the magnetic field). |
| **Sydney** | SCID interview | MDD subjects: presence of axis-I disorders other than MDD, panic disorder, social anxiety disorder, or generalized anxiety disorder.  Control subjects: no Axis-I diagnosis, no medication use.  Exclusion criteria for all subjects included medical instability (as determined by a psychiatrist), history of neurological disease (e.g. tumour, head trauma, epilepsy), medical illness known to impact cognitive and brain function (e.g. cancer), intellectual and/or developmental disability and insufficient English for neuropsychological assessment. All subjects were asked to abstain from drug or alcohol use for 48 hours prior to testing and informed about a drug screen protocol. |
| **QTIM** | CIDI interview | MDD subjects: presence of axis-I disorders other than MDD and anxiety disorders  Control subjects: antidepressant use, psychiatric disorders  All subjects: relatedness between subjects, left handedness, history of neurological or other severe medical illness, head injury or current or past diagnosis of substance abuse, use of cognition affecting medication and general MRI contraindications |
| **Rotterdam study** | SCAN interview | MDD subjects: Persons who screened positive for depressive symptoms on CESD but did not meet criteria for MDD from SCAN interview. Persons who screened positive for depressive symptoms on CESD and then did not undergo SCAN interview. Presence of axis-I disorders other than MDD and anxiety disorders (DSM-IV). Persons with MRI contraindications.  Control subjects: use of psychoanaleptics, MRI contraindications. |
| **Bipolar Family Study** | SCID interview | MDD subjects: presence of other axis I diagnoses.  Control subjects: no medical history, including neurological and psychiatric history, as well as no previous or actual use of psychotropic medication  All subjects: any major neurological disorder, learning disability, or any history of head injury that included loss of consciousness and any contraindications to MRI. |
| **DepOx** | SCID interview | MDD subjects: presence of axis-I disorders other than MDD and anxiety disorders (DSM-IV), clinically significant risk of suicidal behaviour, having contraindications to escitalopram treatment or being treated with psychotropic medication less than three weeks before the study (five weeks in the case of fluoxetine)  Control subjects: current or past history of Axis I disorder as defined by DSM-IV  Both groups: major somatic or neurological disorders, pregnancy or breast-feeding, contra-indications to MR imaging or concurrent medication which could alter emotional processing |
| **MPIP** | M-CIDI/SCAN interview | 1. Munich Antidepressant Response Signature (MARS) study  MDD subjects (clinical consensus diagnosis or M-CIDI (since 2008)): depressive syndromes secondary to any medical or neurological condition (e. g., in­toxi­cation, drug abuse, stroke), the presence of manic, hypomanic or mixed aﬀective symptoms, lifetime diagnosis of alcohol dependence, illicit drug abuse or the presence of severe medical conditions (e.g., ischemic heart disease). Patients with bipolar depression were excluded for the current MR study.  Control subjects: age > 65, MMSE<27, presence of severe somatic diseases or lifetime history of the following axis I disorders as assessed by the M-CIDI interview: alcohol dependence, drug abuse or dependence, possible psychotic disorder, mood disorder, anxiety disorder including OCD and PTSD, somatoform disorder, dissociative disorder NOS, and eating disorder  2. Recurrent unipolar depression (RUD) study  MDD subjects (SCAN interview): presence of manic episodes, mood incongruent psychotic symptoms, the presence of a lifetime diagnosis of intravenous drug abuse and depressive symptoms only secondary to alcohol or substance abuse or to medical illness or medication.  Control subjects: presence of severe somatic diseases or life-time history of anxiety and affective disorders according to the Composite International Diagnostic-Screener (CIDI-S)  All subjects: gross incidental MR findings such as territorial infarction, tumor, hydrocephalus, malformations and anatomical deviations (e.g. enlarged ventricles) that prevent appropriate image processing were additional exclusion criteria.  3. MR images of 9 additional controls acquired at the LMU, Munich, meeting equivalent criteria as the RUD control sample were included. |
| **MMDP 3T** | SCID interview | MDD subjects: presence of axis-I disorders other than MDD and anxiety disorders (DSM-IV).  Control subjects had to have no psychiatric history, as assessed with the SCID-I/P  Symptom severity was assessed using the 17-item Hamilton Depression Rating Scale (HDRS-17) and the Global Assessment of Functioning Scale (GAF). Control subjects also received these measures to rule out the presence of sub-threshold psychiatric illness.  Exclusion criteria for all groups were:  (1) substance-use related disorder within the past 6 months as determined by the SCID;  (2) lifetime history of substance dependence as measured by the SCID;  (3) PTSD as determined by the SCID;  (4) treatment with anti-cholinergic or typical (first generation) anti-psychotic medication;  (5) use of alcohol or illicit psychoactive substance within 48 h of testing;  (6) untreated medical illness such as uncontrolled diabetes or other endocrine disorders eg. Cushing’s;  (7) history of head injury with loss of consciousness;  (8) history of neurological disease; and  (9) past treatment with electroconvulsive therapy (ECT), transcranial magnetic stimulation, or psychotherapy within the past year.  (10) English comprehension lower than a grade 6 reading level. |
| **Houston** | SCID interview | MDD subjects: age below 18; lifetime or current diagnosis of psychotic disorder, or bipolar I or II disorder; substance abuse/dependence in 6 months prior to study inclusion; current major medical problems.  Control subjects: age below 18; current major medical problems; current psychiatric or neurologic disorder; history of psychiatric disorders in a first-degree relative; current major medical problems.  Both groups: MRI contra-indications |
| **Sexpect** | ICD-10 interview | MDD subjects: history of seizures, medication with glutamate modulating drugs (ketamine, riluzole, etc.) or benzodiazepines, prior electroconvulsive therapy (ECT) treatments and pregnancy, atypical forms of depression, any additional psychiatric disorder, and a history of substance abuse or dependence.  Control subjects: psychiatric illness.  Both groups: contraindications against MRI, major medical and neurological illness. |
| **Melbourne** | SCID interview | MDD subjects: lifetime or current SCID-I diagnosis of psychotic disorder, or bipolar I or II disorder.  Control subjects: any SCID-I diagnosis or medication use.  Both groups: Acute or unstable medical disorder; general MRI contraindications. |
| **Muenster cohort** | SCID interview | MDD subjects: presence of bipolar disorder, schizoaffective disorders and schizophrenia; substance-related disorders or current benzodiazepine treatment (wash out of at least three half-lives before study participation), and former electroconvulsive therapy.  Control subjects: any current or former psychiatric disorder.  Both groups: any neurological abnormalities, MRI contra-indications. |
| **Novosibirsk** | MINI, SCID, ICD-10 interviews | MDD subjects: Presence of axis-I disorders other than MDD, panic disorder, social anxiety disorder, or generalized anxiety disorder and any use of psychotropic medication other than stable use of SSRIs or infrequent benzodiazepine use; age 18 or below; alcohol or substance abuse/dependence within 6 months of study participation; current major medical problems.  Control subjects: age over 65; any current or former psychiatric disorder.  Both groups: MRI contra-indications. |
| **Stanford** | SCID interview | MDD subjects: presence of axis-I disorders other than MDD, anxiety and eating disorders .  Control subjects: control individuals did not meet diagnostic criteria for any current psychiatric disorder or past mood disorder.  Both groups: alcohol / substance abuse or dependence within six months prior to MRI scanning, history of head trauma with loss of consciousness > 5 min, aneurysm, or any neurological or metabolic disorders that require ongoing medication or that may affect the central nervous system (including thyroid disease, diabetes, epilepsy or other seizures, or multiple sclerosis), MRI contra-indications, or bad MRI data (e.g., extreme movement). |

MDD: Major Depressive Disorder; CIDI: the Composite International Diagnostic Interview; SCID: Structured Clinical Interview for DSM disorders; SCAN: Schedules for Clinical Assessment in Neuropsychiatry; MINI: M.I.N.I. International Neuropsychiatric Interview; CESD: Center for Epidemiologic Studies Depression scale; DSM: Diagnostic and Statistical Manual of Mental Disorders; MRI: Magnetic Resonance Imaging; OCD: Obsessive Compulsive Disorder; PTSD: Posttraumatic Stress Disorder.

**Table S4**: Image acquisition and processing by site

| **Sample** | **Scanner vendor and type** | **Acquisition parameters** | **Freesurfer version** | **Slice orientation** | **Operating system** |
| --- | --- | --- | --- | --- | --- |
| **NESDA** | 3T Phillips Achieva/Intera | 3D gradient-echo T1-weighted sequence. TR=9 msec; TE=3.5 msec; flip angle 8º, FOV = 256 mm; matrix: 25x62x56; in plane voxel size = 1 mm × 1 mm x 1 mm; 170 slices. | 5.3 | Sagittal | Linux-centos4_x86_64 |
| **Imaging Genetics Dublin** | 3T Phillips Achieva | A sagittal T1 3D TFE was used to scan all participants. TR=8.5 msec; TE=3.9 msec; FOV = 256 mm, AP: 256 mm, RL: 160 mm; matrix: 256×256. | 5.3 | Sagittal | Mac OS |
| **Clinical Depression Dublin** | 1.5T Siemens Vision | 3D-MPRAGE T1-weighted sequence. TR=11.6 msec; TE=4.9 msec; FOV=230 mm; matrix 512 x 512, slice thickness: 1.5 mm. | 5.3 | Coronal | Mac OS |
| **CODE** | 3T Siemens Trio (4 Sites), 3 T Philips Achieva (1 site) | Siemens: T1 mprage, voxel size 1 mm x 1 mm x 1 mm; TR=1900 msec; TE=2.52 msec; Sample 1: 192 slices, Sample 2: 176 slices (except 1 site: 192)  Philips: T1 3D-TFE, voxel size 1 mm x 1 mm x 1 mm; TR=8.3 msec; TE=3.8 msec; 170 slices. | 5.3 | Sagittal | Ubuntu 12.04 LTS (Linux 64bit) |
| **CLING** | 3T Siemens Tim Trio | Standard 3D T1-weighted turbo fast low angle shot (turbo FLASH); voxel size 1 mm x 1 mm x 1mm (based on the ADNI protocol (Jack et al. 2008); TR=225 msec; TE=3.26 msec, FOV=256 x 256 x 192 | 5.1 | Sagittal | Linux |
| **SHIP** | 1.5T Siemens Avanto | 3D T1-weighted (MP-RAGE/ axial plane); TR=1900 msec; TE=3.4 msec; Flip angle=15°; voxel size 1 mm x 1 mm x 1 mm | 5.3 | Axial | Centos6_x86_64 |
| **SHIP-trend** | 1.5T Siemens Avanto | 3D T1-weighted (MP-RAGE/ axial plane); TR=1900 msec; TE=3.4 msec; Flip angle=15°; voxel size 1 mm x 1 mm x 1 mm | 5.3 | Axial | Centos6_x86_64 |
| **Sydney** | 3T GE MR750 | 3D T1-weighted sequence. TR=7.2 msec; TE=2.78 msec; matrix =256; FOV=240; No. slices=196; thick=0.9mm; inplane resolution=0.9375 | 5.1 | Coronal | Linux_Ubuntu12.04_64 |
| **QTIM** | Bruker 4T Whole-body MRI | 3D T1 weighted sequence. TR=1500 msec; TE=3.35 msec; flip angle=8°, 256 or 240 (coronal or sagittal) slices, FOV=240 mm, matrix 256x256x256 (or 256x256x240) | 5.1 | Coronal, then sagittal following software upgrade. | Linux-centos4_x86_64-stable-pub-v5.1.0 |
| **Rotterdam study** | 1.5T Signa Excite -General Electric Healthcare,  Milwaukee, USA, software version 11x | 3D GRE T1 weighted sequence. TR= 13.8 msec; TE=2.8 msec; TI=400 ms; Flip angle=20°; FOV=25cm2; maxtrix 416 x 256; voxel size 1 mm x 1 mm x 1 mm. | 5.1 | Axial |  |
| **Bipolar Family Study** | 1.5T GE Signa | T1-weighted sequence. TR=500 msec; TE=4 msec; flip angle 8°; matrix 192 x 192; 180 slices; voxel size 1.25 mm x 1.25 mm x 1.20 mm; FOV=24, phase FOV 1 | 5.3 | Coronal | linux 6, x86_64, kernel 2.6.32 |
| **DepOx** | 3T Siemens Tim Trio | T1 weighted sequence. TR=1100 msec; TE=4.8 msec; TI=2040 msec; voxel size 0.78 mm x 0.8 mm x 0.78 mm on a 208 x 256 x 200 grid, | 5.3 | Transversal | Suse Linux x86_64 |
| **MPIP** | 1.5T GE and Siemens (the latter: only few cases) | #1: T1-weighted SPGR sagittal 3D volume. TR=1030 msec; TE=3.4 msec; 124 slices; matrix=256x256; FOV=23.0x23.0 cm2; voxel size=0.8975 mm x0.8975 mm x 1.2-1.4 mm; flip angle=90°; birdcage resonator.  #2: same scanner as #1, platform update Signa Excite, sagittal T1-weighted (spin echo sequence, TR=9.7 msec, TE=2.1 msec; FOV=25.0x25.0 cm2, voxel size=0.875 mm x0.875 mm x1.2 mm, 124-132 slices, flip angle=90°.    #3: Siemens 1.5 Tesla, Vario, 3D MPRAGE, TR=11.6 msec; TE=4.9 msec; FOV 23x23 cm2; matrix 512x512; 126 axial slices; voxel site 0.45 mm x 0.45 mm x 1.5 mm. (only N=2 subjects) | 5.3 | 1.5 GE: sagittal  1.5 Siemens: axial | Linux 2.6.37.1-1.2-desktop x86_64 |
| **MMDP 3T** | 3T MRI Signa GE Excite | Axial T-1 weighted sequence; 3D SPGR pulse; fast IRP sequence; TR = 7.012 msec; TE = 2.1 msec; Ti=450 msec; flip angle= 12°; FOV = 240; slice thickness = 2 mm no skip; frequency matrix = 320; phase matrix = 192; frequency direction = A/P | 5.3 | Axial | Mac OSX 10.9.5 |
| **Houston** | 1.5 T Philips Medical Systems Gyroscan Intera | T-1 weighted fast field echo sequence (3D T1-FFE) with repetition time (TR) = 25 ms, echo time (TE) = 5 ms, field of view (FOV) = 240 mm × 220 mm, gap = 0, and matrix size = 256 × 256. | 5.3 | Sagittal | Fedora 19 |
| **Sexpect** | 3 Tesla Siemens MAGNETOM Trio scanner (Siemens, Erlangen, Germany) | High resolution T1-weighted structural MRI scans of the brain were acquired for structural reference using a 3D-MPRAGE sequence (TE = 4.77 ms, TR = 2500 ms, T1 = 1100 ms, flip angle = 7°, bandwidth = 140 Hz/pixel, acquisition matrix = 256 × 256 × 192, isometric voxel size = 1.0 mm3). | 5.3 | Sagittal | Oracle Linux Server_x86_64 |
| **Melbourne** | 3T GE Signa Excite | 3D BRAVO sequence 140; TR=7900 ms; TE=3000 ms; flip angle=13º; FOV=256 mm; matrix=256 x 256 | 5.3 | Axial | Linux Debian x86 64 |
| **Muenster cohort** | 3T Philips Gyroscan Intera | 3D fast gradient echo sequence (turbo field echo), repetition time = 7.4 milliseconds, echo time = 3.4 milliseconds, flip angle = 9°, two signal averages, inversion prepulse every 814.5 milliseconds, acquired over a field of view of 256 (feet-head [FH]) × 204 (anterior-posterior [AP]) × 160 (right-left [RL]) mm, phase encoding in AP and RL direction, reconstructed to cubic voxels of .5 mm × .5 mm × .5 mm | 5.3 | Sagittal | Red Hat Enterprise Linux Server release 5.11 (Tikanga) |
| **Novosibirsk** | 3T GE Discovery™ MR750w | Whole-brain T1-weighted images - 3D fast spin gradient echo sequence (FSPGR BRAVO), repetition time = 9.5 ms, echo time = 3.7 ms, flip angle = 3°, acquired over a field of view of 256 (feet-head [FH]) × 256 (anterior-posterior [AP]) × 188 (right-left [RL]) mm, reconstructed to cubic voxels of 1 mm × 1 mm × 1 mm | 5.3 | Sagittal | OS X 10.10 |
| **Stanford** | 1.5T GE Signa Excite | Whole-brain T1-weighted images were collected using a spoiled gradient echo (SPGR) pulse sequence (116 sagittal slices; through-plane resolution = 1.5 mm; in-plane resolution = 0.86 x 0.86 mm; flip angle = 15 degrees; repetition time [TR] = 8.3-10.1 ms; echo time [TE] = 1.7-3.0; inversion time [TI] = 300 ms; matrix = 256 x 192). | 5.3 | Sagittal | Linux-centos6_x86_64 |

3D: three-dimensional; TR: repetition time; TE: echo time; FOV: field of view

**Adult meta-analyses results for cortical thickness**

**Supplementary Table S5**: Full meta-analytic results for thickness of each structure for the Diagnosis by Sex interaction controlling for age, sex and scan center. Adjusted Cohen's d is reported.

|  | **Cohen's d ^a^** | **Std. Err.** | **95% CI** | **% Difference** | **P-value** | **FDR P-value** | **I^2^** | **# Controls** | **# Patients** |
| --- | --- | --- | --- | --- | --- | --- | --- | --- | --- |
|  | **(Dx by Sex)** |  |  |  |  |  |  |  |  |
| **Right temporal pole** | -0.043 | 0.033 | [-0.108 - 0.022] | -0.473 | 0.192 | 0.940 | 7.968 | 7631 | 1871 |
| **Left temporal pole** | -0.035 | 0.030 | [-0.094 - 0.024] | -0.357 | 0.242 | 0.940 | 0.001 | 7614 | 1871 |
| **Left entorhinal cortex** | -0.025 | 0.039 | [-0.101 - 0.05] | -0.291 | 0.512 | 0.940 | 24.548 | 7605 | 1866 |
| **Right precentral gyrus** | -0.021 | 0.041 | [-0.102 - 0.059] | -0.127 | 0.605 | 0.940 | 32.385 | 7643 | 1894 |
| **Right lateral occipital cortex** | -0.021 | 0.030 | [-0.08 - 0.038] | -0.124 | 0.482 | 0.940 | 0.005 | 7650 | 1898 |
| **Left superior parietal cortex** | -0.018 | 0.045 | [-0.106 - 0.07] | -0.089 | 0.687 | 0.940 | 42.057 | 7645 | 1896 |
| **Left paracentral lobule** | -0.018 | 0.044 | [-0.104 - 0.069] | -0.101 | 0.686 | 0.940 | 39.981 | 7650 | 1899 |
| **Right fusiform gyrus** | -0.015 | 0.030 | [-0.074 - 0.043] | -0.075 | 0.606 | 0.940 | <0.001 | 7649 | 1898 |
| **Left precuneus** | -0.013 | 0.045 | [-0.101 - 0.076] | -0.061 | 0.776 | 0.941 | 42.336 | 7649 | 1893 |
| **Left rostral middle frontal gyrus** | -0.012 | 0.037 | [-0.086 - 0.061] | -0.060 | 0.738 | 0.940 | 21.927 | 7653 | 1899 |
| **Right inferior temporal gyrus** | -0.010 | 0.036 | [-0.081 - 0.061] | -0.055 | 0.780 | 0.941 | 18.031 | 7640 | 1885 |
| **Left rostral anterior cingulate cortex** | -0.005 | 0.030 | [-0.064 - 0.054] | -0.051 | 0.861 | 0.941 | 0.016 | 7656 | 1896 |
| **Left caudal middle frontal gyrus** | -0.004 | 0.030 | [-0.063 - 0.055] | -0.019 | 0.901 | 0.941 | <0.001 | 7647 | 1898 |
| **Left precentral gyrus** | 0.001 | 0.035 | [-0.067 - 0.07] | 0.007 | 0.972 | 0.972 | 14.750 | 7637 | 1895 |
| **Left superior frontal gyrus** | 0.001 | 0.030 | [-0.058 - 0.06] | 0.006 | 0.966 | 0.972 | 0.018 | 7652 | 1899 |
| **Right banks superior temporal sulcus** | 0.003 | 0.042 | [-0.08 - 0.085] | 0.019 | 0.950 | 0.972 | 33.750 | 7613 | 1827 |
| **Left fusiform gyrus** | 0.005 | 0.034 | [-0.062 - 0.072] | 0.025 | 0.883 | 0.941 | 11.461 | 7645 | 1896 |
| **Right pericalcarine cortex** | 0.005 | 0.038 | [-0.069 - 0.079] | 0.037 | 0.893 | 0.941 | 22.976 | 7633 | 1896 |
| **Left lateral occipital cortex** | 0.006 | 0.033 | [-0.059 - 0.072] | 0.036 | 0.853 | 0.941 | 10.057 | 7645 | 1898 |
| **Right postcentral gyrus** | 0.007 | 0.037 | [-0.066 - 0.079] | 0.038 | 0.856 | 0.941 | 20.605 | 7642 | 1897 |
| **Left pars triangularis** | 0.009 | 0.035 | [-0.061 - 0.078] | 0.052 | 0.810 | 0.941 | 16.169 | 7651 | 1897 |
| **Right entorhinal cortex** | 0.009 | 0.035 | [-0.059 - 0.076] | 0.104 | 0.801 | 0.941 | 12.684 | 7602 | 1862 |
| **Left insula** | 0.010 | 0.046 | [-0.081 - 0.101] | 0.052 | 0.829 | 0.941 | 44.836 | 7652 | 1898 |
| **Right caudal middle frontal gyrus** | 0.010 | 0.030 | [-0.049 - 0.069] | 0.053 | 0.736 | 0.940 | <0.001 | 7650 | 1900 |
| **Right rostral middle frontal gyrus** | 0.011 | 0.036 | [-0.06 - 0.081] | 0.052 | 0.766 | 0.941 | 17.494 | 7650 | 1899 |
| **Left pars opercularis** | 0.011 | 0.032 | [-0.052 - 0.075] | 0.055 | 0.722 | 0.940 | 6.408 | 7655 | 1897 |
| **Left superior temporal gyrus** | 0.014 | 0.041 | [-0.066 - 0.094] | 0.084 | 0.735 | 0.940 | 28.725 | 7551 | 1806 |
| **Right superior frontal gyrus** | 0.014 | 0.035 | [-0.055 - 0.083] | 0.070 | 0.683 | 0.940 | 15.399 | 7649 | 1900 |
| **Right hemisphere average thickness** | 0.015 | 0.040 | [-0.063 - 0.092] | 0.054 | 0.708 | 0.940 | 28.423 | 7658 | 1902 |
| **Right pars opercularis** | 0.016 | 0.035 | [-0.052 - 0.084] | 0.081 | 0.643 | 0.940 | 14.100 | 7651 | 1896 |
| **Left frontal pole** | 0.016 | 0.037 | [-0.057 - 0.089] | 0.171 | 0.664 | 0.940 | 21.644 | 7656 | 1899 |
| **Right supramarginal gyrus** | 0.016 | 0.041 | [-0.063 - 0.096] | 0.083 | 0.689 | 0.940 | 30.603 | 7633 | 1874 |
| **Left hemisphere average thickness** | 0.018 | 0.038 | [-0.058 - 0.093] | 0.065 | 0.648 | 0.940 | 25.246 | 7658 | 1902 |
| **Right lateral orbitofrontal cortex** | 0.018 | 0.032 | [-0.045 - 0.081] | 0.105 | 0.580 | 0.940 | 6.783 | 7644 | 1902 |
| **Left postcentral gyrus** | 0.018 | 0.039 | [-0.058 - 0.095] | 0.101 | 0.640 | 0.940 | 27.060 | 7630 | 1894 |
| **Right superior parietal cortex** | 0.018 | 0.043 | [-0.067 - 0.103] | 0.091 | 0.673 | 0.940 | 38.383 | 7645 | 1895 |
| **Right posterior cingulate cortex** | 0.019 | 0.033 | [-0.045 - 0.083] | 0.125 | 0.567 | 0.940 | 7.980 | 7654 | 1900 |
| **Left banks superior temporal sulcus** | 0.019 | 0.038 | [-0.056 - 0.094] | 0.140 | 0.618 | 0.940 | 22.082 | 7571 | 1781 |
| **Right insula** | 0.019 | 0.038 | [-0.055 - 0.094] | 0.105 | 0.610 | 0.940 | 23.198 | 7651 | 1895 |
| **Left parahippocampal gyrus** | 0.020 | 0.030 | [-0.039 - 0.079] | 0.252 | 0.500 | 0.940 | <0.001 | 7648 | 1896 |
| **Right caudal anterior cingulate cortex** | 0.021 | 0.030 | [-0.038 - 0.079] | 0.230 | 0.491 | 0.940 | <0.001 | 7655 | 1898 |
| **Right cuneus** | 0.023 | 0.048 | [-0.071 - 0.117] | 0.143 | 0.628 | 0.940 | 48.866 | 7654 | 1896 |
| **Left transverse temporal gyrus** | 0.024 | 0.030 | [-0.035 - 0.083] | 0.190 | 0.422 | 0.940 | <0.001 | 7635 | 1895 |
| **Right medial orbitofrontal cortex** | 0.025 | 0.038 | [-0.051 - 0.1] | 0.208 | 0.519 | 0.940 | 24.761 | 7628 | 1896 |
| **Left inferior temporal gyrus** | 0.025 | 0.030 | [-0.034 - 0.084] | 0.139 | 0.408 | 0.940 | <0.001 | 7630 | 1872 |
| **Left lateral orbitofrontal cortex** | 0.025 | 0.030 | [-0.033 - 0.084] | 0.154 | 0.396 | 0.940 | <0.001 | 7638 | 1898 |
| **Right superior temporal gyrus** | 0.026 | 0.038 | [-0.048 - 0.1] | 0.155 | 0.495 | 0.940 | 20.883 | 7587 | 1820 |
| **Right middle temporal gyrus** | 0.027 | 0.030 | [-0.032 - 0.086] | 0.149 | 0.374 | 0.940 | <0.001 | 7639 | 1886 |
| **Right parahippocampal gyrus** | 0.028 | 0.040 | [-0.05 - 0.106] | 0.305 | 0.482 | 0.940 | 28.763 | 7654 | 1897 |
| **Left supramarginal gyrus** | 0.029 | 0.033 | [-0.035 - 0.093] | 0.156 | 0.374 | 0.940 | 6.546 | 7609 | 1864 |
| **Right precuneus** | 0.029 | 0.042 | [-0.053 - 0.111] | 0.144 | 0.488 | 0.940 | 34.355 | 7646 | 1894 |
| **Left isthmus cingulate cortex** | 0.032 | 0.030 | [-0.027 - 0.09] | 0.243 | 0.291 | 0.940 | <0.001 | 7655 | 1897 |
| **Right inferior parietal cortex** | 0.032 | 0.035 | [-0.036 - 0.1] | 0.180 | 0.360 | 0.940 | 14.151 | 7641 | 1897 |
| **Left posterior cingulate cortex** | 0.034 | 0.038 | [-0.039 - 0.108] | 0.214 | 0.362 | 0.940 | 22.244 | 7654 | 1900 |
| **Left pars orbitalis** | 0.034 | 0.030 | [-0.024 - 0.093] | 0.251 | 0.252 | 0.940 | <0.001 | 7653 | 1900 |
| **Left caudal anterior cingulate cortex** | 0.034 | 0.030 | [-0.024 - 0.093] | 0.391 | 0.251 | 0.940 | <0.001 | 7650 | 1900 |
| **Left inferior parietal cortex** | 0.037 | 0.031 | [-0.023 - 0.097] | 0.209 | 0.230 | 0.940 | 1.826 | 7638 | 1894 |
| **Left medial orbitofrontal cortex** | 0.038 | 0.030 | [-0.021 - 0.097] | 0.293 | 0.210 | 0.940 | 0.003 | 7609 | 1888 |
| **Right paracentral lobule** | 0.039 | 0.038 | [-0.036 - 0.114] | 0.224 | 0.303 | 0.940 | 24.554 | 7651 | 1901 |
| **Right pars orbitalis** | 0.043 | 0.030 | [-0.016 - 0.102] | 0.311 | 0.150 | 0.940 | <0.001 | 7655 | 1900 |
| **Right lingual gyrus** | 0.044 | 0.037 | [-0.028 - 0.116] | 0.253 | 0.236 | 0.940 | 19.899 | 7641 | 1894 |
| **Right rostral anterior cingulate cortex** | 0.046 | 0.030 | [-0.013 - 0.105] | 0.467 | 0.125 | 0.940 | 0.006 | 7651 | 1899 |
| **Left middle temporal gyrus** | 0.050 | 0.030 | [-0.009 - 0.11] | 0.299 | 0.098 | 0.940 | <0.001 | 7591 | 1822 |
| **Right transverse temporal gyrus** | 0.051 | 0.046 | [-0.038 - 0.141] | 0.412 | 0.262 | 0.940 | 43.557 | 7622 | 1894 |
| **Right frontal pole** | 0.051 | 0.037 | [-0.022 - 0.125] | 0.534 | 0.172 | 0.940 | 22.228 | 7657 | 1899 |
| **Left lingual gyrus** | 0.062 | 0.051 | [-0.038 - 0.163] | 0.360 | 0.224 | 0.940 | 54.793 | 7640 | 1895 |
| **Right pars triangularis** | 0.065 | 0.032 | [0.003 - 0.128] | 0.377 | 0.041 | 0.715 | 5.618 | 7645 | 1897 |
| **Right isthmus cingulate cortex** | 0.073 | 0.030 | [0.014 - 0.132] | 0.578 | 0.015 | 0.352 | <0.001 | 7651 | 1897 |
| **Left pericalcarine cortex** | 0.086 | 0.032 | [0.023 - 0.149] | 0.606 | 0.007 | 0.252 | 5.977 | 7645 | 1894 |
| **Left cuneus** | 0.092 | 0.030 | [0.033 - 0.151] | 0.569 | 0.002 | 0.149 | <0.001 | 7652 | 1897 |

Dx: Diagnosis.

**Supplementary Table S6**: Full meta-analytic results for thickness of each structure for the Diagnosis by Age interaction controlling for age, sex and scan center. Adjusted Cohen's d is reported.

|  | **Cohen's d ^a^** | **Std. Err.** | **95% CI** | **% Difference** | **P-value** | **FDR P-value** | **I^2^** | **# Controls** | **# Patients** |
| --- | --- | --- | --- | --- | --- | --- | --- | --- | --- |
|  | **(Dx by Age)** |  |  |  |  |  |  |  |  |
| **Left transverse temporal gyrus** | -0.077 | 0.032 | [-0.14 - -0.014] | -0.608 | 0.017 | 0.674 | 6.337 | 7635 | 1895 |
| **Right transverse temporal gyrus** | -0.067 | 0.040 | [-0.145 - 0.011] | -0.541 | 0.091 | 0.914 | 28.931 | 7622 | 1894 |
| **Left superior temporal gyrus** | -0.061 | 0.031 | [-0.121 - -0.001] | -0.374 | 0.046 | 0.714 | <0.001 | 7551 | 1806 |
| **Right supramarginal gyrus** | -0.060 | 0.050 | [-0.158 - 0.037] | -0.311 | 0.226 | 0.990 | 52.186 | 7633 | 1874 |
| **Left insula** | -0.059 | 0.047 | [-0.152 - 0.034] | -0.306 | 0.215 | 0.990 | 47.450 | 7652 | 1898 |
| **Left rostral anterior cingulate cortex** | -0.051 | 0.060 | [-0.169 - 0.067] | -0.498 | 0.395 | 0.999 | 67.649 | 7656 | 1896 |
| **Right rostral anterior cingulate cortex** | -0.047 | 0.070 | [-0.184 - 0.091] | -0.475 | 0.505 | 0.999 | 76.819 | 7651 | 1899 |
| **Right posterior cingulate cortex** | -0.045 | 0.052 | [-0.147 - 0.057] | -0.302 | 0.386 | 0.999 | 56.407 | 7654 | 1900 |
| **Right isthmus cingulate cortex** | -0.039 | 0.064 | [-0.164 - 0.085] | -0.313 | 0.535 | 0.999 | 71.352 | 7651 | 1897 |
| **Left lateral occipital cortex** | -0.039 | 0.030 | [-0.098 - 0.02] | -0.225 | 0.192 | 0.961 | <0.001 | 7645 | 1898 |
| **Right caudal anterior cingulate cortex** | -0.035 | 0.063 | [-0.158 - 0.088] | -0.392 | 0.574 | 0.999 | 70.377 | 7655 | 1898 |
| **Left fusiform gyrus** | -0.032 | 0.030 | [-0.091 - 0.027] | -0.157 | 0.287 | 0.999 | <0.001 | 7645 | 1896 |
| **Left caudal anterior cingulate cortex** | -0.030 | 0.050 | [-0.128 - 0.068] | -0.341 | 0.548 | 0.999 | 52.668 | 7650 | 1900 |
| **Left inferior temporal gyrus** | -0.027 | 0.042 | [-0.108 - 0.055] | -0.149 | 0.521 | 0.999 | 32.522 | 7630 | 1872 |
| **Right inferior temporal gyrus** | -0.023 | 0.068 | [-0.156 - 0.111] | -0.123 | 0.740 | 0.999 | 75.091 | 7640 | 1885 |
| **Right insula** | -0.021 | 0.066 | [-0.151 - 0.109] | -0.115 | 0.750 | 0.999 | 73.808 | 7651 | 1895 |
| **Right lateral occipital cortex** | -0.020 | 0.030 | [-0.079 - 0.039] | -0.116 | 0.510 | 0.999 | <0.001 | 7650 | 1898 |
| **Left frontal pole** | -0.019 | 0.054 | [-0.126 - 0.088] | -0.201 | 0.726 | 0.999 | 60.202 | 7656 | 1899 |
| **Right medial orbitofrontal cortex** | -0.019 | 0.067 | [-0.15 - 0.113] | -0.155 | 0.783 | 0.999 | 74.541 | 7628 | 1896 |
| **Left supramarginal gyrus** | -0.018 | 0.030 | [-0.078 - 0.041] | -0.099 | 0.546 | 0.999 | <0.001 | 7609 | 1864 |
| **Left isthmus cingulate cortex** | -0.018 | 0.052 | [-0.119 - 0.083] | -0.139 | 0.727 | 0.999 | 55.759 | 7655 | 1897 |
| **Right paracentral lobule** | -0.018 | 0.030 | [-0.076 - 0.041] | -0.100 | 0.557 | 0.999 | <0.001 | 7651 | 1901 |
| **Right fusiform gyrus** | -0.018 | 0.030 | [-0.076 - 0.041] | -0.086 | 0.559 | 0.999 | <0.001 | 7649 | 1898 |
| **Left pars opercularis** | -0.017 | 0.048 | [-0.11 - 0.077] | -0.080 | 0.725 | 0.999 | 48.322 | 7655 | 1897 |
| **Right inferior parietal cortex** | -0.010 | 0.030 | [-0.069 - 0.048] | -0.059 | 0.727 | 0.999 | <0.001 | 7641 | 1897 |
| **Left temporal pole** | -0.007 | 0.030 | [-0.066 - 0.052] | -0.069 | 0.821 | 0.999 | <0.001 | 7614 | 1871 |
| **Left middle temporal gyrus** | -0.007 | 0.033 | [-0.071 - 0.058] | -0.040 | 0.837 | 0.999 | 7.096 | 7591 | 1822 |
| **Right frontal pole** | -0.006 | 0.035 | [-0.075 - 0.063] | -0.066 | 0.857 | 0.999 | 15.565 | 7657 | 1899 |
| **Right parahippocampal gyrus** | -0.006 | 0.053 | [-0.111 - 0.098] | -0.069 | 0.906 | 0.999 | 58.443 | 7654 | 1897 |
| **Left hemisphere average thickness** | -0.005 | 0.030 | [-0.064 - 0.054] | -0.019 | 0.862 | 0.999 | <0.001 | 7658 | 1902 |
| **Right superior temporal gyrus** | -0.005 | 0.042 | [-0.088 - 0.078] | -0.031 | 0.902 | 0.999 | 32.763 | 7587 | 1820 |
| **Left parahippocampal gyrus** | -0.003 | 0.054 | [-0.109 - 0.102] | -0.043 | 0.949 | 0.999 | 59.036 | 7648 | 1896 |
| **Left banks superior temporal sulcus** | -0.003 | 0.031 | [-0.063 - 0.057] | -0.025 | 0.911 | 0.999 | <0.001 | 7571 | 1781 |
| **Right pars triangularis** | -0.003 | 0.030 | [-0.062 - 0.056] | -0.016 | 0.928 | 0.999 | <0.001 | 7645 | 1897 |
| **Right postcentral gyrus** | -0.001 | 0.030 | [-0.06 - 0.058] | -0.007 | 0.966 | 0.999 | <0.001 | 7642 | 1897 |
| **Left superior parietal cortex** | 0.000 | 0.034 | [-0.067 - 0.066] | -0.002 | 0.990 | 0.999 | 11.244 | 7645 | 1896 |
| **Left inferior parietal cortex** | 0.000 | 0.030 | [-0.059 - 0.059] | 0.000 | 0.999 | 0.999 | <0.001 | 7638 | 1894 |
| **Left lateral orbitofrontal cortex** | 2.92E-04 | 0.058 | [-0.113 - 0.113] | 0.002 | 0.996 | 0.999 | 64.827 | 7638 | 1898 |
| **Left postcentral gyrus** | 5.18E-04 | 0.033 | [-0.065 - 0.066] | 0.003 | 0.988 | 0.999 | 10.054 | 7630 | 1894 |
| **Left medial orbitofrontal cortex** | 0.005 | 0.051 | [-0.095 - 0.105] | 0.037 | 0.926 | 0.999 | 54.080 | 7609 | 1888 |
| **Left paracentral lobule** | 0.006 | 0.040 | [-0.072 - 0.083] | 0.034 | 0.880 | 0.999 | 28.218 | 7650 | 1899 |
| **Right temporal pole** | 0.007 | 0.030 | [-0.052 - 0.066] | 0.076 | 0.818 | 0.999 | <0.001 | 7631 | 1871 |
| **Left entorhinal cortex** | 0.008 | 0.044 | [-0.079 - 0.095] | 0.093 | 0.856 | 0.999 | 40.437 | 7605 | 1866 |
| **Left pars orbitalis** | 0.009 | 0.046 | [-0.082 - 0.099] | 0.065 | 0.848 | 0.999 | 45.152 | 7653 | 1900 |
| **Right banks superior temporal sulcus** | 0.010 | 0.046 | [-0.08 - 0.1] | 0.072 | 0.825 | 0.999 | 42.901 | 7613 | 1827 |
| **Left lingual gyrus** | 0.014 | 0.030 | [-0.045 - 0.073] | 0.082 | 0.635 | 0.999 | <0.001 | 7640 | 1895 |
| **Left posterior cingulate cortex** | 0.015 | 0.059 | [-0.1 - 0.129] | 0.091 | 0.804 | 0.999 | 65.824 | 7654 | 1900 |
| **Left rostral middle frontal gyrus** | 0.015 | 0.036 | [-0.055 - 0.085] | 0.072 | 0.679 | 0.999 | 16.912 | 7653 | 1899 |
| **Left pars triangularis** | 0.015 | 0.035 | [-0.054 - 0.083] | 0.091 | 0.670 | 0.999 | 14.596 | 7651 | 1897 |
| **Right pericalcarine cortex** | 0.016 | 0.041 | [-0.065 - 0.098] | 0.120 | 0.692 | 0.999 | 33.140 | 7633 | 1896 |
| **Right middle temporal gyrus** | 0.019 | 0.030 | [-0.04 - 0.078] | 0.105 | 0.531 | 0.999 | <0.001 | 7639 | 1886 |
| **Left superior frontal gyrus** | 0.019 | 0.038 | [-0.056 - 0.094] | 0.097 | 0.616 | 0.999 | 23.979 | 7652 | 1899 |
| **Right cuneus** | 0.020 | 0.039 | [-0.056 - 0.096] | 0.123 | 0.603 | 0.999 | 25.380 | 7654 | 1896 |
| **Left pericalcarine cortex** | 0.028 | 0.030 | [-0.031 - 0.087] | 0.196 | 0.353 | 0.999 | <0.001 | 7645 | 1894 |
| **Right superior frontal gyrus** | 0.030 | 0.030 | [-0.029 - 0.088] | 0.144 | 0.322 | 0.999 | <0.001 | 7649 | 1900 |
| **Right hemisphere average thickness** | 0.030 | 0.030 | [-0.028 - 0.089] | 0.111 | 0.312 | 0.999 | <0.001 | 7658 | 1902 |
| **Right lingual gyrus** | 0.031 | 0.038 | [-0.044 - 0.105] | 0.177 | 0.421 | 0.999 | 23.515 | 7641 | 1894 |
| **Left precentral gyrus** | 0.031 | 0.030 | [-0.028 - 0.09] | 0.177 | 0.306 | 0.999 | <0.001 | 7637 | 1895 |
| **Left caudal middle frontal gyrus** | 0.033 | 0.030 | [-0.026 - 0.092] | 0.168 | 0.272 | 0.999 | <0.001 | 7647 | 1898 |
| **Left precuneus** | 0.034 | 0.030 | [-0.025 - 0.093] | 0.161 | 0.262 | 0.999 | <0.001 | 7649 | 1893 |
| **Left cuneus** | 0.039 | 0.030 | [-0.019 - 0.098] | 0.243 | 0.189 | 0.961 | <0.001 | 7652 | 1897 |
| **Right superior parietal cortex** | 0.041 | 0.030 | [-0.018 - 0.1] | 0.205 | 0.173 | 0.961 | <0.001 | 7645 | 1895 |
| **Right precentral gyrus** | 0.045 | 0.034 | [-0.022 - 0.112] | 0.268 | 0.190 | 0.961 | 12.769 | 7643 | 1894 |
| **Right caudal middle frontal gyrus** | 0.051 | 0.030 | [-0.008 - 0.11] | 0.268 | 0.090 | 0.914 | <0.001 | 7650 | 1900 |
| **Right pars orbitalis** | 0.057 | 0.039 | [-0.02 - 0.134] | 0.413 | 0.144 | 0.961 | 27.216 | 7655 | 1900 |
| **Right entorhinal cortex** | 0.060 | 0.038 | [-0.016 - 0.135] | 0.711 | 0.120 | 0.961 | 24.204 | 7602 | 1862 |
| **Right pars opercularis** | 0.060 | 0.031 | [0 - 0.12] | 0.300 | 0.051 | 0.714 | 1.982 | 7651 | 1896 |
| **Right rostral middle frontal gyrus** | 0.060 | 0.030 | [0.002 - 0.119] | 0.292 | 0.044 | 0.714 | <0.001 | 7650 | 1899 |
| **Right lateral orbitofrontal cortex** | 0.068 | 0.048 | [-0.027 - 0.162] | 0.398 | 0.159 | 0.961 | 49.166 | 7644 | 1902 |
| **Right precuneus** | 0.070 | 0.030 | [0.011 - 0.129] | 0.349 | 0.019 | 0.674 | <0.001 | 7646 | 1894 |

Dx: Diagnosis.

**Supplementary Table S7**: Full meta-analytic results for thickness of each structure for first episode MDD patients versus Controls comparison controlling for age, sex and scan center. Adjusted Cohen's d is reported.

|  | **Cohen's d ^a^** | **Std. Err.** | **95% CI** | **% Difference** | **P-value** | **FDR P-value** | **I^2^** | **# Controls** | **# Patients** |
| --- | --- | --- | --- | --- | --- | --- | --- | --- | --- |
|  | **(First episode MDD vs CTL)** |  |  |  |  |  |  |  |  |
| **Right isthmus cingulate cortex** | -0.190 | 0.067 | [-0.321 - -0.058] | -1.502 | 0.005 | 0.050 | 37.978 | 7250 | 534 |
| **Right insula** | -0.187 | 0.048 | [-0.282 - -0.092] | -1.018 | 1.10E-04 | 0.008 | <0.001 | 7253 | 535 |
| **Left insula** | -0.171 | 0.048 | [-0.266 - -0.076] | -0.893 | 3.93E-04 | 0.014 | <0.001 | 7253 | 535 |
| **Right posterior cingulate cortex** | -0.163 | 0.048 | [-0.257 - -0.068] | -1.089 | 7.40E-04 | 0.017 | <0.001 | 7250 | 535 |
| **Right fusiform gyrus** | -0.159 | 0.058 | [-0.273 - -0.045] | -0.777 | 0.006 | 0.050 | 21.721 | 7250 | 535 |
| **Left fusiform gyrus** | -0.158 | 0.048 | [-0.253 - -0.064] | -0.779 | 0.001 | 0.018 | <0.001 | 7249 | 535 |
| **Right pars opercularis** | -0.150 | 0.100 | [-0.345 - 0.045] | -0.752 | 0.132 | 0.290 | 71.529 | 7250 | 534 |
| **Right supramarginal gyrus** | -0.150 | 0.079 | [-0.305 - 0.005] | -0.769 | 0.058 | 0.164 | 53.625 | 7243 | 527 |
| **Left rostral anterior cingulate cortex** | -0.141 | 0.048 | [-0.235 - -0.046] | -1.372 | 0.004 | 0.049 | <0.001 | 7251 | 535 |
| **Right rostral anterior cingulate cortex** | -0.134 | 0.048 | [-0.228 - -0.039] | -1.354 | 0.006 | 0.050 | <0.001 | 7250 | 535 |
| **Left medial orbitofrontal cortex** | -0.131 | 0.048 | [-0.226 - -0.037] | -1.019 | 0.007 | 0.050 | <0.001 | 7222 | 532 |
| **Right caudal anterior cingulate cortex** | -0.129 | 0.048 | [-0.224 - -0.035] | -1.437 | 0.007 | 0.050 | <0.001 | 7252 | 535 |
| **Left superior frontal gyrus** | -0.128 | 0.048 | [-0.223 - -0.034] | -0.650 | 0.008 | 0.050 | <0.001 | 7247 | 534 |
| **Right frontal pole** | -0.127 | 0.063 | [-0.25 - -0.004] | -1.327 | 0.042 | 0.148 | 29.922 | 7253 | 533 |
| **Right pars orbitalis** | -0.119 | 0.058 | [-0.232 - -0.006] | -0.858 | 0.039 | 0.145 | 20.839 | 7252 | 535 |
| **Right lateral orbitofrontal cortex** | -0.117 | 0.051 | [-0.218 - -0.017] | -0.691 | 0.022 | 0.098 | 7.112 | 7240 | 535 |
| **Left frontal pole** | -0.117 | 0.048 | [-0.211 - -0.022] | -1.228 | 0.016 | 0.093 | <0.001 | 7251 | 534 |
| **Right inferior temporal gyrus** | -0.115 | 0.049 | [-0.211 - -0.019] | -0.626 | 0.018 | 0.098 | 1.369 | 7248 | 534 |
| **Right medial orbitofrontal cortex** | -0.112 | 0.048 | [-0.206 - -0.017] | -0.937 | 0.021 | 0.098 | <0.001 | 7237 | 535 |
| **Right superior frontal gyrus** | -0.111 | 0.048 | [-0.205 - -0.016] | -0.538 | 0.022 | 0.098 | <0.001 | 7244 | 535 |
| **Left banks superior temporal sulcus** | -0.108 | 0.049 | [-0.204 - -0.012] | -0.790 | 0.028 | 0.116 | <0.001 | 7195 | 512 |
| **Left middle temporal gyrus** | -0.104 | 0.049 | [-0.2 - -0.009] | -0.620 | 0.032 | 0.125 | <0.001 | 7212 | 523 |
| **Right middle temporal gyrus** | -0.096 | 0.048 | [-0.191 - -0.002] | -0.538 | 0.046 | 0.154 | <0.001 | 7244 | 533 |
| **Left isthmus cingulate cortex** | -0.095 | 0.048 | [-0.189 - 0] | -0.727 | 0.050 | 0.159 | <0.001 | 7251 | 535 |
| **Left posterior cingulate cortex** | -0.093 | 0.048 | [-0.187 - 0.002] | -0.581 | 0.054 | 0.162 | <0.001 | 7251 | 535 |
| **Left lateral orbitofrontal cortex** | -0.093 | 0.048 | [-0.187 - 0.002] | -0.559 | 0.056 | 0.162 | <0.001 | 7233 | 534 |
| **Right hemisphere average thickness** | -0.089 | 0.048 | [-0.183 - 0.006] | -0.325 | 0.065 | 0.175 | <0.001 | 7253 | 535 |
| **Right transverse temporal gyrus** | -0.085 | 0.048 | [-0.18 - 0.01] | -0.684 | 0.079 | 0.205 | <0.001 | 7217 | 532 |
| **Left hemisphere average thickness** | -0.083 | 0.048 | [-0.177 - 0.012] | -0.304 | 0.087 | 0.217 | <0.001 | 7253 | 535 |
| **Left caudal anterior cingulate cortex** | -0.078 | 0.065 | [-0.205 - 0.05] | -0.884 | 0.231 | 0.385 | 34.391 | 7246 | 534 |
| **Left pars opercularis** | -0.078 | 0.048 | [-0.172 - 0.017] | -0.370 | 0.108 | 0.260 | <0.001 | 7252 | 533 |
| **Right superior temporal gyrus** | -0.075 | 0.049 | [-0.171 - 0.02] | -0.451 | 0.122 | 0.286 | <0.001 | 7229 | 521 |
| **Left inferior parietal cortex** | -0.074 | 0.065 | [-0.202 - 0.054] | -0.422 | 0.255 | 0.404 | 34.678 | 7238 | 533 |
| **Right paracentral lobule** | -0.073 | 0.048 | [-0.168 - 0.021] | -0.417 | 0.128 | 0.290 | <0.001 | 7248 | 534 |
| **Right banks superior temporal sulcus** | -0.071 | 0.057 | [-0.183 - 0.04] | -0.508 | 0.208 | 0.364 | 17.288 | 7222 | 520 |
| **Right rostral middle frontal gyrus** | -0.070 | 0.048 | [-0.165 - 0.024] | -0.339 | 0.145 | 0.292 | <0.001 | 7245 | 533 |
| **Left transverse temporal gyrus** | -0.070 | 0.048 | [-0.165 - 0.024] | -0.555 | 0.146 | 0.292 | <0.001 | 7231 | 531 |
| **Right entorhinal cortex** | -0.070 | 0.048 | [-0.165 - 0.024] | -0.835 | 0.145 | 0.292 | <0.001 | 7240 | 534 |
| **Left inferior temporal gyrus** | -0.066 | 0.081 | [-0.226 - 0.093] | -0.371 | 0.414 | 0.579 | 56.612 | 7248 | 533 |
| **Right lingual gyrus** | -0.065 | 0.048 | [-0.16 - 0.03] | -0.375 | 0.181 | 0.339 | <0.001 | 7239 | 532 |
| **Left pars orbitalis** | -0.061 | 0.048 | [-0.156 - 0.033] | -0.450 | 0.203 | 0.364 | <0.001 | 7249 | 535 |
| **Right inferior parietal cortex** | -0.061 | 0.071 | [-0.2 - 0.078] | -0.346 | 0.390 | 0.558 | 44.048 | 7239 | 534 |
| **Left entorhinal cortex** | -0.060 | 0.057 | [-0.173 - 0.052] | -0.695 | 0.293 | 0.446 | 20.158 | 7240 | 535 |
| **Left rostral middle frontal gyrus** | -0.059 | 0.048 | [-0.154 - 0.035] | -0.287 | 0.220 | 0.375 | <0.001 | 7250 | 534 |
| **Left parahippocampal gyrus** | -0.058 | 0.078 | [-0.21 - 0.094] | -0.718 | 0.457 | 0.615 | 52.556 | 7251 | 534 |
| **Left pars triangularis** | -0.055 | 0.048 | [-0.15 - 0.039] | -0.338 | 0.250 | 0.404 | <0.001 | 7247 | 535 |
| **Left supramarginal gyrus** | -0.053 | 0.052 | [-0.155 - 0.05] | -0.283 | 0.316 | 0.460 | 8.843 | 7229 | 530 |
| **Left temporal pole** | -0.050 | 0.048 | [-0.144 - 0.045] | -0.502 | 0.304 | 0.453 | <0.001 | 7226 | 534 |
| **Right precuneus** | -0.049 | 0.072 | [-0.19 - 0.091] | -0.245 | 0.491 | 0.625 | 44.715 | 7244 | 533 |
| **Right caudal middle frontal gyrus** | -0.048 | 0.077 | [-0.198 - 0.102] | -0.255 | 0.527 | 0.634 | 51.344 | 7245 | 534 |
| **Left caudal middle frontal gyrus** | -0.045 | 0.063 | [-0.168 - 0.077] | -0.232 | 0.468 | 0.618 | 30.127 | 7243 | 534 |
| **Right pars triangularis** | -0.038 | 0.048 | [-0.132 - 0.057] | -0.216 | 0.437 | 0.599 | <0.001 | 7244 | 535 |
| **Left precentral gyrus** | -0.031 | 0.048 | [-0.126 - 0.063] | -0.179 | 0.518 | 0.634 | <0.001 | 7238 | 533 |
| **Right precentral gyrus** | -0.030 | 0.048 | [-0.125 - 0.064] | -0.180 | 0.531 | 0.634 | <0.001 | 7242 | 535 |
| **Right postcentral gyrus** | -0.027 | 0.074 | [-0.173 - 0.118] | -0.155 | 0.712 | 0.803 | 48.207 | 7239 | 533 |
| **Left lingual gyrus** | -0.026 | 0.048 | [-0.12 - 0.069] | -0.148 | 0.598 | 0.698 | <0.001 | 7236 | 530 |
| **Right lateral occipital cortex** | -0.016 | 0.085 | [-0.183 - 0.151] | -0.092 | 0.853 | 0.905 | 60.599 | 7245 | 535 |
| **Left superior temporal gyrus** | -0.015 | 0.049 | [-0.11 - 0.081] | -0.090 | 0.764 | 0.835 | <0.001 | 7196 | 522 |
| **Right parahippocampal gyrus** | -0.001 | 0.048 | [-0.095 - 0.094] | -0.007 | 0.990 | 0.990 | <0.001 | 7251 | 535 |
| **Left paracentral lobule** | 0.003 | 0.079 | [-0.151 - 0.158] | 0.018 | 0.967 | 0.981 | 53.921 | 7246 | 534 |
| **Left lateral occipital cortex** | 0.005 | 0.048 | [-0.089 - 0.1] | 0.030 | 0.913 | 0.940 | <0.001 | 7242 | 535 |
| **Left precuneus** | 0.007 | 0.048 | [-0.088 - 0.102] | 0.033 | 0.885 | 0.925 | <0.001 | 7244 | 531 |
| **Left postcentral gyrus** | 0.011 | 0.048 | [-0.084 - 0.105] | 0.058 | 0.827 | 0.890 | <0.001 | 7227 | 535 |
| **Right superior parietal cortex** | 0.015 | 0.048 | [-0.08 - 0.109] | 0.075 | 0.757 | 0.835 | <0.001 | 7241 | 535 |
| **Right temporal pole** | 0.020 | 0.049 | [-0.076 - 0.117] | 0.222 | 0.682 | 0.783 | 2.225 | 7239 | 533 |
| **Left superior parietal cortex** | 0.033 | 0.048 | [-0.061 - 0.128] | 0.163 | 0.491 | 0.625 | <0.001 | 7241 | 534 |
| **Left cuneus** | 0.036 | 0.058 | [-0.077 - 0.149] | 0.221 | 0.534 | 0.634 | 20.628 | 7251 | 534 |
| **Right cuneus** | 0.064 | 0.048 | [-0.03 - 0.158] | 0.393 | 0.184 | 0.339 | <0.001 | 7252 | 535 |
| **Left pericalcarine cortex** | 0.073 | 0.055 | [-0.034 - 0.18] | 0.513 | 0.182 | 0.339 | 14.283 | 7240 | 534 |
| **Right pericalcarine cortex** | 0.146 | 0.130 | [-0.108 - 0.4] | 1.065 | 0.260 | 0.404 | 83.706 | 7234 | 535 |

**^a^** Included Samples: CLING, Imaging Genetics Dublin, Clinical Depression Dublin, Houston, MMDP 3T, MPIP, Muenster Cohort, NESDA, DepOx, SHIP, SHIP-trend, Sydney, QTIM, Rotterdam study.

MDD: Major Depressive Disorder; CTL: Controls.

**Supplementary Table S8**: Full meta-analytic results for thickness of each structure for recurrent episode MDD patients versus Controls comparison controlling for age, sex and scan center. Adjusted Cohen's d is reported.

|  | **Cohen's d ^a^** | **Std. Err.** | **95% CI** | **% Difference** | **P-value** | **FDR P-value** | **I^2^** | **# Controls** | **# Patients** |
| --- | --- | --- | --- | --- | --- | --- | --- | --- | --- |
|  | **(Recurrent MDD vs CTL)** |  |  |  |  |  |  |  |  |
| **Right medial orbitofrontal cortex** | -0.177 | 0.074 | [-0.322 - -0.031] | -1.482 | 0.017 | 0.173 | 71.633 | 7430 | 1297 |
| **Left rostral anterior cingulate cortex** | -0.163 | 0.061 | [-0.283 - -0.042] | -1.583 | 0.008 | 0.128 | 57.772 | 7448 | 1297 |
| **Left medial orbitofrontal cortex** | -0.144 | 0.035 | [-0.213 - -0.075] | -1.118 | 4.03E-05 | 0.003 | <0.001 | 7411 | 1293 |
| **Right lateral orbitofrontal cortex** | -0.14 | 0.077 | [-0.292 - 0.011] | -0.825 | 0.07 | 0.244 | 74.303 | 7436 | 1302 |
| **Right rostral anterior cingulate cortex** | -0.137 | 0.079 | [-0.292 - 0.019] | -1.387 | 0.085 | 0.269 | 75.547 | 7443 | 1299 |
| **Right posterior cingulate cortex** | -0.131 | 0.065 | [-0.258 - -0.004] | -0.878 | 0.043 | 0.235 | 62.476 | 7446 | 1300 |
| **Left posterior cingulate cortex** | -0.128 | 0.063 | [-0.251 - -0.005] | -0.801 | 0.042 | 0.235 | 59.932 | 7446 | 1300 |
| **Right parahippocampal gyrus** | -0.127 | 0.068 | [-0.259 - 0.006] | -1.379 | 0.062 | 0.244 | 65.917 | 7446 | 1297 |
| **Left pars opercularis** | -0.119 | 0.063 | [-0.241 - 0.004] | -0.565 | 0.058 | 0.244 | 59.644 | 7448 | 1299 |
| **Left fusiform gyrus** | -0.117 | 0.053 | [-0.222 - -0.012] | -0.574 | 0.029 | 0.223 | 44.804 | 7438 | 1296 |
| **Right inferior temporal gyrus** | -0.116 | 0.047 | [-0.208 - -0.024] | -0.63 | 0.013 | 0.154 | 30.244 | 7433 | 1287 |
| **Right fusiform gyrus** | -0.107 | 0.056 | [-0.218 - 0.004] | -0.522 | 0.058 | 0.244 | 50.387 | 7442 | 1298 |
| **Right superior frontal gyrus** | -0.107 | 0.053 | [-0.21 - -0.004] | -0.517 | 0.043 | 0.235 | 43.457 | 7441 | 1300 |
| **Left isthmus cingulate cortex** | -0.105 | 0.035 | [-0.175 - -0.036] | -0.811 | 0.003 | 0.103 | 1.205 | 7448 | 1297 |
| **Left parahippocampal gyrus** | -0.103 | 0.075 | [-0.251 - 0.045] | -1.286 | 0.171 | 0.374 | 72.708 | 7441 | 1297 |
| **Right insula** | -0.101 | 0.055 | [-0.208 - 0.006] | -0.55 | 0.065 | 0.244 | 47.124 | 7443 | 1297 |
| **Left insula** | -0.1 | 0.044 | [-0.187 - -0.013] | -0.521 | 0.024 | 0.211 | 24.288 | 7444 | 1298 |
| **Right middle temporal gyrus** | -0.097 | 0.035 | [-0.166 - -0.029] | -0.543 | 0.005 | 0.127 | <0.001 | 7431 | 1290 |
| **Right banks superior temporal sulcus** | -0.092 | 0.035 | [-0.162 - -0.023] | -0.658 | 0.009 | 0.128 | <0.001 | 7407 | 1243 |
| **Left middle temporal gyrus** | -0.086 | 0.042 | [-0.169 - -0.002] | -0.508 | 0.044 | 0.235 | 17.149 | 7387 | 1235 |
| **Right caudal anterior cingulate cortex** | -0.079 | 0.044 | [-0.165 - 0.006] | -0.885 | 0.068 | 0.244 | 22.35 | 7447 | 1298 |
| **Left pars orbitalis** | -0.075 | 0.054 | [-0.18 - 0.031] | -0.546 | 0.167 | 0.374 | 46.223 | 7445 | 1300 |
| **Left lateral orbitofrontal cortex** | -0.068 | 0.057 | [-0.179 - 0.043] | -0.411 | 0.228 | 0.443 | 50.705 | 7430 | 1299 |
| **Right isthmus cingulate cortex** | -0.068 | 0.035 | [-0.137 - 0] | -0.541 | 0.051 | 0.244 | <0.001 | 7443 | 1299 |
| **Left hemisphere average thickness** | -0.065 | 0.042 | [-0.148 - 0.017] | -0.24 | 0.122 | 0.339 | 19.063 | 7450 | 1302 |
| **Right rostral middle frontal gyrus** | -0.062 | 0.077 | [-0.212 - 0.088] | -0.298 | 0.42 | 0.655 | 73.89 | 7442 | 1301 |
| **Left inferior parietal cortex** | -0.061 | 0.035 | [-0.129 - 0.008] | -0.344 | 0.083 | 0.269 | <0.001 | 7430 | 1296 |
| **Left pars triangularis** | -0.057 | 0.035 | [-0.125 - 0.012] | -0.346 | 0.104 | 0.317 | <0.001 | 7443 | 1297 |
| **Left superior frontal gyrus** | -0.054 | 0.035 | [-0.122 - 0.015] | -0.271 | 0.126 | 0.339 | <0.001 | 7444 | 1300 |
| **Left rostral middle frontal gyrus** | -0.051 | 0.035 | [-0.12 - 0.018] | -0.247 | 0.145 | 0.374 | <0.001 | 7446 | 1300 |
| **Right hemisphere average thickness** | -0.051 | 0.047 | [-0.142 - 0.041] | -0.185 | 0.28 | 0.497 | 30.865 | 7450 | 1302 |
| **Right entorhinal cortex** | -0.049 | 0.035 | [-0.118 - 0.021] | -0.577 | 0.17 | 0.374 | <0.001 | 7396 | 1268 |
| **Right supramarginal gyrus** | -0.046 | 0.054 | [-0.152 - 0.061] | -0.236 | 0.398 | 0.649 | 46.052 | 7427 | 1282 |
| **Left inferior temporal gyrus** | -0.045 | 0.078 | [-0.198 - 0.108] | -0.251 | 0.565 | 0.719 | 74.094 | 7422 | 1276 |
| **Right pars orbitalis** | -0.044 | 0.045 | [-0.133 - 0.044] | -0.32 | 0.323 | 0.539 | 26.173 | 7447 | 1300 |
| **Right frontal pole** | -0.042 | 0.055 | [-0.15 - 0.066] | -0.434 | 0.45 | 0.655 | 48.267 | 7449 | 1301 |
| **Right pars triangularis** | -0.041 | 0.07 | [-0.178 - 0.096] | -0.237 | 0.557 | 0.719 | 67.906 | 7437 | 1297 |
| **Left precentral gyrus** | -0.039 | 0.035 | [-0.107 - 0.03] | -0.221 | 0.27 | 0.497 | <0.001 | 7431 | 1297 |
| **Left banks superior temporal sulcus** | -0.038 | 0.036 | [-0.109 - 0.032] | -0.281 | 0.284 | 0.497 | <0.001 | 7373 | 1206 |
| **Left precuneus** | -0.038 | 0.051 | [-0.139 - 0.062] | -0.183 | 0.456 | 0.655 | 40.574 | 7441 | 1297 |
| **Right transverse temporal gyrus** | -0.038 | 0.035 | [-0.107 - 0.03] | -0.307 | 0.275 | 0.497 | <0.001 | 7414 | 1297 |
| **Left caudal middle frontal gyrus** | -0.037 | 0.058 | [-0.15 - 0.075] | -0.19 | 0.516 | 0.719 | 52.4 | 7440 | 1300 |
| **Left supramarginal gyrus** | -0.035 | 0.035 | [-0.105 - 0.034] | -0.19 | 0.319 | 0.539 | <0.001 | 7401 | 1271 |
| **Right inferior parietal cortex** | -0.033 | 0.045 | [-0.122 - 0.055] | -0.189 | 0.459 | 0.655 | 26.216 | 7433 | 1298 |
| **Right superior temporal gyrus** | -0.022 | 0.036 | [-0.092 - 0.048] | -0.131 | 0.538 | 0.719 | <0.001 | 7382 | 1242 |
| **Left paracentral lobule** | -0.021 | 0.035 | [-0.09 - 0.047] | -0.121 | 0.543 | 0.719 | <0.001 | 7443 | 1300 |
| **Right precentral gyrus** | -0.021 | 0.035 | [-0.09 - 0.048] | -0.125 | 0.55 | 0.719 | <0.001 | 7437 | 1294 |
| **Left caudal anterior cingulate cortex** | -0.018 | 0.054 | [-0.124 - 0.088] | -0.207 | 0.736 | 0.805 | 46.415 | 7442 | 1301 |
| **Left lateral occipital cortex** | -0.018 | 0.035 | [-0.087 - 0.05] | -0.105 | 0.602 | 0.737 | <0.001 | 7437 | 1298 |
| **Left transverse temporal gyrus** | -0.018 | 0.045 | [-0.106 - 0.07] | -0.14 | 0.692 | 0.789 | 26.122 | 7427 | 1299 |
| **Right caudal middle frontal gyrus** | -0.016 | 0.041 | [-0.097 - 0.065] | -0.084 | 0.698 | 0.789 | 17.012 | 7442 | 1302 |
| **Right pars opercularis** | -0.012 | 0.05 | [-0.11 - 0.085] | -0.062 | 0.803 | 0.851 | 36.967 | 7443 | 1297 |
| **Left entorhinal cortex** | -0.004 | 0.035 | [-0.073 - 0.066] | -0.042 | 0.918 | 0.945 | <0.001 | 7399 | 1272 |
| **Left superior parietal cortex** | -0.002 | 0.046 | [-0.093 - 0.088] | -0.011 | 0.961 | 0.961 | 29.489 | 7437 | 1298 |
| **Right precuneus** | 0.003 | 0.062 | [-0.118 - 0.124] | 0.015 | 0.96 | 0.961 | 58.548 | 7438 | 1296 |
| **Left superior temporal gyrus** | 0.013 | 0.067 | [-0.118 - 0.145] | 0.08 | 0.845 | 0.883 | 62.595 | 7350 | 1224 |
| **Right temporal pole** | 0.013 | 0.036 | [-0.057 - 0.083] | 0.143 | 0.716 | 0.796 | 1.398 | 7423 | 1277 |
| **Right lingual gyrus** | 0.015 | 0.048 | [-0.079 - 0.109] | 0.085 | 0.759 | 0.817 | 33.503 | 7433 | 1297 |
| **Right paracentral lobule** | 0.016 | 0.035 | [-0.053 - 0.084] | 0.09 | 0.649 | 0.77 | <0.001 | 7443 | 1302 |
| **Right lateral occipital cortex** | 0.018 | 0.035 | [-0.051 - 0.086] | 0.104 | 0.61 | 0.737 | <0.001 | 7442 | 1298 |
| **Left lingual gyrus** | 0.023 | 0.056 | [-0.087 - 0.132] | 0.131 | 0.684 | 0.789 | 49.468 | 7432 | 1300 |
| **Left temporal pole** | 0.032 | 0.041 | [-0.049 - 0.113] | 0.326 | 0.436 | 0.655 | 15.873 | 7409 | 1275 |
| **Right cuneus** | 0.034 | 0.067 | [-0.098 - 0.166] | 0.211 | 0.61 | 0.737 | 65.45 | 7446 | 1297 |
| **Left frontal pole** | 0.036 | 0.048 | [-0.057 - 0.129] | 0.381 | 0.446 | 0.655 | 32.492 | 7448 | 1300 |
| **Right postcentral gyrus** | 0.043 | 0.035 | [-0.025 - 0.112] | 0.244 | 0.218 | 0.437 | <0.001 | 7436 | 1299 |
| **Right superior parietal cortex** | 0.046 | 0.035 | [-0.023 - 0.115] | 0.23 | 0.189 | 0.4 | <0.001 | 7437 | 1295 |
| **Left postcentral gyrus** | 0.048 | 0.035 | [-0.02 - 0.117] | 0.266 | 0.169 | 0.374 | <0.001 | 7423 | 1294 |
| **Left cuneus** | 0.053 | 0.035 | [-0.015 - 0.122] | 0.33 | 0.126 | 0.339 | <0.001 | 7444 | 1300 |
| **Right pericalcarine cortex** | 0.057 | 0.045 | [-0.031 - 0.145] | 0.418 | 0.203 | 0.418 | 26.098 | 7425 | 1297 |
| **Left pericalcarine cortex** | 0.106 | 0.074 | [-0.038 - 0.251] | 0.747 | 0.149 | 0.374 | 71.38 | 7437 | 1295 |

**^a^** Included Samples: CODE, CLING, Imaging Genetics Dublin, Clinical Depression Dublin, Houston, Sexpect, MMDP 3T, Melbourne, MPIP, Muenster Cohort, NESDA, Novosibirsk, DepOx, SHIP, SHIP-trend, Sydney, Stanford, Rotterdam study.

MDD: Major Depressive Disorder; CTL: Controls.

**Supplementary Table S9**: Full meta-analytic results for thickness of each structure for first episode MDD versus recurrent episode MDD patients comparison controlling for age, sex and scan center. Adjusted Cohen's d is reported.

|  | **Cohen's d ^a^** | **Std. Err.** | **95% CI** | **% Difference** | **P-value** | **FDR P-value** | **I^2^** | **# First episode** | **# Recurrent episode** |
| --- | --- | --- | --- | --- | --- | --- | --- | --- | --- |
|  | **(First episode MDD vs Recurrent MDD)** |  |  |  |  |  |  |  |  |
| **Right pericalcarine cortex** | -0.050 | 0.105 | [-0.257 - 0.156] | -0.367 | 0.633 | 0.987 | 67.433 | 535 | 1109 |
| **Right parahippocampal gyrus** | -0.038 | 0.112 | [-0.257 - 0.182] | -0.410 | 0.737 | 0.987 | 71.405 | 535 | 1110 |
| **Right cuneus** | -0.031 | 0.054 | [-0.138 - 0.075] | -0.193 | 0.562 | 0.987 | <0.001 | 535 | 1110 |
| **Left isthmus cingulate cortex** | -0.027 | 0.054 | [-0.133 - 0.08] | -0.204 | 0.624 | 0.987 | <0.001 | 535 | 1110 |
| **Left posterior cingulate cortex** | -0.025 | 0.054 | [-0.131 - 0.081] | -0.156 | 0.646 | 0.987 | <0.001 | 535 | 1111 |
| **Right medial orbitofrontal cortex** | -0.012 | 0.054 | [-0.119 - 0.094] | -0.102 | 0.822 | 0.987 | <0.001 | 535 | 1107 |
| **Right temporal pole** | -0.009 | 0.090 | [-0.186 - 0.168] | -0.098 | 0.921 | 0.987 | 55.325 | 533 | 1109 |
| **Left precuneus** | -0.008 | 0.054 | [-0.115 - 0.098] | -0.039 | 0.880 | 0.987 | <0.001 | 531 | 1110 |
| **Left precentral gyrus** | -0.008 | 0.054 | [-0.114 - 0.098] | -0.046 | 0.883 | 0.987 | <0.001 | 533 | 1110 |
| **Left inferior parietal cortex** | -0.005 | 0.054 | [-0.111 - 0.102] | -0.026 | 0.932 | 0.987 | <0.001 | 533 | 1109 |
| **Right middle temporal gyrus** | -0.003 | 0.054 | [-0.109 - 0.103] | -0.017 | 0.956 | 0.987 | <0.001 | 533 | 1105 |
| **Left superior parietal cortex** | -0.001 | 0.054 | [-0.107 - 0.105] | -0.004 | 0.987 | 0.995 | <0.001 | 534 | 1109 |
| **Left rostral anterior cingulate cortex** | 0.000 | 0.078 | [-0.154 - 0.153] | -0.004 | 0.995 | 0.995 | 41.547 | 535 | 1109 |
| **Left parahippocampal gyrus** | 0.004 | 0.078 | [-0.148 - 0.156] | 0.051 | 0.958 | 0.987 | 40.649 | 534 | 1110 |
| **Left medial orbitofrontal cortex** | 0.004 | 0.054 | [-0.102 - 0.111] | 0.032 | 0.939 | 0.987 | <0.001 | 532 | 1104 |
| **Left paracentral lobule** | 0.005 | 0.054 | [-0.101 - 0.111] | 0.028 | 0.928 | 0.987 | <0.001 | 534 | 1110 |
| **Left caudal middle frontal gyrus** | 0.005 | 0.058 | [-0.108 - 0.118] | 0.025 | 0.931 | 0.987 | 6.875 | 534 | 1109 |
| **Left inferior temporal gyrus** | 0.006 | 0.107 | [-0.203 - 0.214] | 0.031 | 0.958 | 0.987 | 68.163 | 533 | 1110 |
| **Left cuneus** | 0.007 | 0.067 | [-0.124 - 0.139] | 0.046 | 0.912 | 0.987 | 24.794 | 534 | 1111 |
| **Left pars triangularis** | 0.008 | 0.063 | [-0.116 - 0.132] | 0.050 | 0.897 | 0.987 | 17.537 | 535 | 1109 |
| **Left lateral occipital cortex** | 0.009 | 0.054 | [-0.097 - 0.115] | 0.052 | 0.868 | 0.987 | <0.001 | 535 | 1110 |
| **Right inferior temporal gyrus** | 0.012 | 0.085 | [-0.154 - 0.178] | 0.067 | 0.884 | 0.987 | 49.622 | 534 | 1110 |
| **Right entorhinal cortex** | 0.013 | 0.054 | [-0.093 - 0.119] | 0.154 | 0.811 | 0.987 | <0.001 | 534 | 1110 |
| **Right inferior parietal cortex** | 0.018 | 0.054 | [-0.089 - 0.124] | 0.100 | 0.744 | 0.987 | <0.001 | 534 | 1107 |
| **Left pericalcarine cortex** | 0.018 | 0.054 | [-0.089 - 0.124] | 0.125 | 0.743 | 0.987 | <0.001 | 534 | 1108 |
| **Left pars orbitalis** | 0.020 | 0.055 | [-0.089 - 0.128] | 0.144 | 0.722 | 0.987 | 2.141 | 535 | 1111 |
| **Left middle temporal gyrus** | 0.021 | 0.059 | [-0.096 - 0.137] | 0.122 | 0.729 | 0.987 | 8.534 | 523 | 1072 |
| **Left rostral middle frontal gyrus** | 0.028 | 0.071 | [-0.112 - 0.168] | 0.136 | 0.694 | 0.987 | 31.444 | 534 | 1109 |
| **Left supramarginal gyrus** | 0.029 | 0.054 | [-0.077 - 0.136] | 0.158 | 0.590 | 0.987 | <0.001 | 530 | 1099 |
| **Right banks superior temporal sulcus** | 0.030 | 0.055 | [-0.079 - 0.138] | 0.211 | 0.592 | 0.987 | <0.001 | 520 | 1062 |
| **Right superior parietal cortex** | 0.030 | 0.054 | [-0.076 - 0.137] | 0.152 | 0.575 | 0.987 | <0.001 | 535 | 1111 |
| **Right precentral gyrus** | 0.031 | 0.054 | [-0.076 - 0.137] | 0.182 | 0.572 | 0.987 | <0.001 | 535 | 1108 |
| **Right pars triangularis** | 0.031 | 0.054 | [-0.075 - 0.138] | 0.181 | 0.563 | 0.987 | <0.001 | 535 | 1108 |
| **Left pars opercularis** | 0.033 | 0.054 | [-0.074 - 0.139] | 0.156 | 0.546 | 0.987 | <0.001 | 533 | 1111 |
| **Left superior temporal gyrus** | 0.033 | 0.063 | [-0.09 - 0.156] | 0.203 | 0.598 | 0.987 | 15.592 | 522 | 1079 |
| **Right fusiform gyrus** | 0.036 | 0.054 | [-0.071 - 0.142] | 0.174 | 0.511 | 0.987 | <0.001 | 535 | 1111 |
| **Right superior frontal gyrus** | 0.037 | 0.054 | [-0.07 - 0.143] | 0.177 | 0.500 | 0.987 | <0.001 | 535 | 1110 |
| **Right rostral middle frontal gyrus** | 0.037 | 0.054 | [-0.07 - 0.143] | 0.177 | 0.498 | 0.987 | <0.001 | 533 | 1110 |
| **Right caudal middle frontal gyrus** | 0.039 | 0.086 | [-0.129 - 0.207] | 0.205 | 0.649 | 0.987 | 50.638 | 534 | 1111 |
| **Left hemisphere average thickness** | 0.041 | 0.054 | [-0.065 - 0.147] | 0.151 | 0.448 | 0.987 | <0.001 | 535 | 1111 |
| **Right precuneus** | 0.042 | 0.065 | [-0.085 - 0.169] | 0.210 | 0.514 | 0.987 | 20.342 | 533 | 1109 |
| **Left fusiform gyrus** | 0.046 | 0.054 | [-0.061 - 0.152] | 0.224 | 0.401 | 0.987 | <0.001 | 535 | 1109 |
| **Right transverse temporal gyrus** | 0.048 | 0.054 | [-0.058 - 0.155] | 0.389 | 0.373 | 0.987 | <0.001 | 532 | 1107 |
| **Left entorhinal cortex** | 0.050 | 0.068 | [-0.085 - 0.184] | 0.570 | 0.469 | 0.987 | 26.841 | 535 | 1108 |
| **Right lateral occipital cortex** | 0.050 | 0.091 | [-0.129 - 0.229] | 0.292 | 0.585 | 0.987 | 56.560 | 535 | 1109 |
| **Left postcentral gyrus** | 0.051 | 0.054 | [-0.055 - 0.158] | 0.283 | 0.345 | 0.987 | <0.001 | 535 | 1106 |
| **Left lateral orbitofrontal cortex** | 0.052 | 0.054 | [-0.055 - 0.158] | 0.313 | 0.339 | 0.987 | <0.001 | 534 | 1108 |
| **Right hemisphere average thickness** | 0.059 | 0.054 | [-0.047 - 0.165] | 0.216 | 0.275 | 0.987 | <0.001 | 535 | 1111 |
| **Right superior temporal gyrus** | 0.059 | 0.065 | [-0.068 - 0.186] | 0.355 | 0.359 | 0.987 | 19.239 | 521 | 1090 |
| **Right postcentral gyrus** | 0.060 | 0.061 | [-0.06 - 0.179] | 0.338 | 0.327 | 0.987 | 13.054 | 533 | 1109 |
| **Right posterior cingulate cortex** | 0.062 | 0.061 | [-0.057 - 0.181] | 0.415 | 0.309 | 0.987 | 13.086 | 535 | 1111 |
| **Left caudal anterior cingulate cortex** | 0.064 | 0.075 | [-0.082 - 0.21] | 0.729 | 0.389 | 0.987 | 36.431 | 534 | 1111 |
| **Right pars orbitalis** | 0.067 | 0.054 | [-0.039 - 0.174] | 0.485 | 0.214 | 0.987 | <0.001 | 535 | 1111 |
| **Right rostral anterior cingulate cortex** | 0.068 | 0.079 | [-0.086 - 0.223] | 0.692 | 0.387 | 0.987 | 42.481 | 535 | 1111 |
| **Right lateral orbitofrontal cortex** | 0.075 | 0.054 | [-0.031 - 0.181] | 0.441 | 0.167 | 0.948 | <0.001 | 535 | 1111 |
| **Right caudal anterior cingulate cortex** | 0.078 | 0.054 | [-0.029 - 0.184] | 0.865 | 0.152 | 0.948 | <0.001 | 535 | 1111 |
| **Left superior frontal gyrus** | 0.078 | 0.054 | [-0.028 - 0.185] | 0.396 | 0.149 | 0.948 | <0.001 | 534 | 1109 |
| **Right isthmus cingulate cortex** | 0.079 | 0.054 | [-0.027 - 0.185] | 0.626 | 0.145 | 0.948 | <0.001 | 534 | 1110 |
| **Left banks superior temporal sulcus** | 0.080 | 0.056 | [-0.029 - 0.189] | 0.587 | 0.150 | 0.948 | <0.001 | 512 | 1046 |
| **Left transverse temporal gyrus** | 0.087 | 0.068 | [-0.047 - 0.221] | 0.687 | 0.202 | 0.987 | 26.534 | 531 | 1109 |
| **Right pars opercularis** | 0.094 | 0.057 | [-0.018 - 0.205] | 0.470 | 0.099 | 0.948 | 5.158 | 534 | 1109 |
| **Left insula** | 0.098 | 0.054 | [-0.009 - 0.204] | 0.510 | 0.072 | 0.948 | <0.001 | 535 | 1111 |
| **Right insula** | 0.102 | 0.054 | [-0.005 - 0.208] | 0.554 | 0.061 | 0.948 | <0.001 | 535 | 1111 |
| **Left lingual gyrus** | 0.104 | 0.088 | [-0.068 - 0.276] | 0.603 | 0.235 | 0.987 | 52.913 | 530 | 1110 |
| **Right lingual gyrus** | 0.111 | 0.069 | [-0.023 - 0.246] | 0.645 | 0.105 | 0.948 | 26.926 | 532 | 1108 |
| **Right supramarginal gyrus** | 0.112 | 0.083 | [-0.05 - 0.275] | 0.576 | 0.176 | 0.948 | 46.853 | 527 | 1099 |
| **Right paracentral lobule** | 0.115 | 0.054 | [0.008 - 0.221] | 0.652 | 0.034 | 0.948 | <0.001 | 534 | 1111 |
| **Left temporal pole** | 0.127 | 0.124 | [-0.115 - 0.369] | 1.285 | 0.304 | 0.987 | 76.690 | 534 | 1108 |
| **Right frontal pole** | 0.163 | 0.118 | [-0.068 - 0.395] | 1.705 | 0.166 | 0.948 | 74.268 | 533 | 1110 |
| **Left frontal pole** | 0.220 | 0.091 | [0.041 - 0.4] | 2.321 | 0.016 | 0.948 | 56.316 | 534 | 1110 |

**^a^** Included Samples: CLING, Imaging Genetics Dublin, Clinical Depression Dublin, Houston, MMDP 3T, MPIP, Muenster Cohort, NESDA, DepOx, SHIP, SHIP-trend, Sydney, Rotterdam study.

MDD: Major Depressive Disorder.

**Supplementary Table S10**: Full meta-analytic results for thickness of each structure for the association with number of episodes in recurrent episode MDD patients controlling for age, sex and scan center. Adjusted Cohen's d is reported.

|  | **Pearson's r ^a^** | **Std. Err.** | **95% CI** | **% Difference** | **P-value** | **FDR P-value** | **I^2^** | **# Patients** |
| --- | --- | --- | --- | --- | --- | --- | --- | --- |
|  | **(#Episodes in Recurrent MDD)** |  |  |  |  |  |  |  |
| **Right superior temporal gyrus** | -0.079 | 0.046 | [-0.169 - 0.011] | -0.951 | 0.084 | 0.989 | <0.001 | 473 |
| **Right isthmus cingulate cortex** | -0.063 | 0.055 | [-0.17 - 0.044] | -0.998 | 0.249 | 0.989 | 19.924 | 496 |
| **Left caudal middle frontal gyrus** | -0.057 | 0.085 | [-0.225 - 0.11] | -0.585 | 0.502 | 0.989 | 66.746 | 496 |
| **Right superior frontal gyrus** | -0.044 | 0.045 | [-0.132 - 0.043] | -0.430 | 0.321 | 0.989 | <0.001 | 496 |
| **Left supramarginal gyrus** | -0.044 | 0.078 | [-0.197 - 0.108] | -0.475 | 0.571 | 0.989 | 56.410 | 483 |
| **Left transverse temporal gyrus** | -0.041 | 0.065 | [-0.169 - 0.087] | -0.646 | 0.531 | 0.989 | 40.535 | 496 |
| **Left superior parietal cortex** | -0.036 | 0.056 | [-0.145 - 0.073] | -0.357 | 0.513 | 0.989 | 22.652 | 496 |
| **Right paracentral gyrus** | -0.032 | 0.045 | [-0.12 - 0.056] | -0.365 | 0.474 | 0.989 | <0.001 | 496 |
| **Right precentral gyrus** | -0.031 | 0.045 | [-0.118 - 0.057] | -0.364 | 0.494 | 0.989 | <0.001 | 495 |
| **Left precentral gyrus** | -0.027 | 0.045 | [-0.115 - 0.061] | -0.305 | 0.554 | 0.989 | <0.001 | 496 |
| **Left inferior parietal cortex** | -0.020 | 0.097 | [-0.21 - 0.171] | -0.223 | 0.840 | 0.992 | 74.787 | 495 |
| **Right medial orbitofrontal cortex** | -0.020 | 0.057 | [-0.13 - 0.091] | -0.329 | 0.729 | 0.989 | 25.645 | 495 |
| **Right parahippocampal gyrus** | -0.019 | 0.110 | [-0.234 - 0.197] | -0.405 | 0.866 | 0.992 | 81.045 | 493 |
| **Right superior parietal cortex** | -0.018 | 0.073 | [-0.161 - 0.124] | -0.184 | 0.800 | 0.992 | 50.444 | 492 |
| **Right rostral middle frontal gyrus** | -0.018 | 0.056 | [-0.129 - 0.092] | -0.177 | 0.744 | 0.989 | 24.230 | 496 |
| **Right caudal anterior cingulate cortex** | -0.014 | 0.045 | [-0.102 - 0.074] | -0.312 | 0.755 | 0.989 | <0.001 | 494 |
| **Right caudal middle frontal gyrus** | -0.014 | 0.045 | [-0.102 - 0.074] | -0.145 | 0.759 | 0.989 | <0.001 | 496 |
| **Left pars triangularis** | -0.013 | 0.091 | [-0.191 - 0.164] | -0.164 | 0.882 | 0.992 | 70.499 | 494 |
| **Left superior temporal gyrus** | -0.007 | 0.105 | [-0.212 - 0.198] | -0.088 | 0.945 | 0.992 | 75.628 | 465 |
| **Right lateral orbitofrontal cortex** | -0.006 | 0.064 | [-0.132 - 0.12] | -0.070 | 0.926 | 0.992 | 38.761 | 496 |
| **Right supramarginal gyrus** | -0.003 | 0.045 | [-0.091 - 0.085] | -0.030 | 0.948 | 0.992 | <0.001 | 493 |
| **Left postcentral gyrus** | -0.001 | 0.071 | [-0.141 - 0.139] | -0.010 | 0.990 | 0.994 | 49.562 | 494 |
| **Left pars opercularis** | 4.19E-04 | 0.058 | [-0.114 - 0.115] | 0.004 | 0.994 | 0.994 | 26.750 | 494 |
| **Right frontal pole** | 9.52E-04 | 0.078 | [-0.152 - 0.154] | 0.020 | 0.990 | 0.994 | 59.425 | 496 |
| **Right lateral occipital cortex** | 0.003 | 0.045 | [-0.085 - 0.091] | 0.033 | 0.949 | 0.992 | <0.001 | 496 |
| **Left paracentral gyrus** | 0.004 | 0.065 | [-0.124 - 0.133] | 0.048 | 0.948 | 0.992 | 40.531 | 496 |
| **Right pars triangularis** | 0.005 | 0.073 | [-0.137 - 0.148] | 0.061 | 0.942 | 0.992 | 52.003 | 495 |
| **Left parahippocampal gyrus** | 0.008 | 0.056 | [-0.101 - 0.118] | 0.205 | 0.883 | 0.992 | 21.960 | 495 |
| **Right lingual gyrus** | 0.012 | 0.045 | [-0.076 - 0.1] | 0.137 | 0.792 | 0.992 | <0.001 | 494 |
| **Right hemisphere average thickness** | 0.013 | 0.054 | [-0.094 - 0.12] | 0.095 | 0.810 | 0.992 | 19.910 | 496 |
| **Right insula** | 0.016 | 0.053 | [-0.087 - 0.119] | 0.178 | 0.755 | 0.989 | 15.863 | 493 |
| **Right inferior parietal cortex** | 0.020 | 0.048 | [-0.074 - 0.113] | 0.221 | 0.681 | 0.989 | 5.663 | 496 |
| **Left superior frontal gyrus** | 0.020 | 0.045 | [-0.067 - 0.108] | 0.204 | 0.652 | 0.989 | <0.001 | 496 |
| **Left lingual gyrus** | 0.021 | 0.056 | [-0.088 - 0.13] | 0.244 | 0.705 | 0.989 | 22.420 | 496 |
| **Right pars opercularis** | 0.023 | 0.045 | [-0.065 - 0.112] | 0.235 | 0.603 | 0.989 | <0.001 | 495 |
| **Right pericalcarine cortex** | 0.023 | 0.078 | [-0.129 - 0.176] | 0.342 | 0.763 | 0.989 | 57.927 | 493 |
| **Left banks superior temporal sulcus** | 0.024 | 0.051 | [-0.077 - 0.124] | 0.347 | 0.645 | 0.989 | 8.983 | 457 |
| **Right posterior cingulate cortex** | 0.026 | 0.044 | [-0.061 - 0.113] | 0.346 | 0.562 | 0.989 | <0.001 | 496 |
| **Left lateral occipital cortex** | 0.032 | 0.048 | [-0.062 - 0.126] | 0.372 | 0.501 | 0.989 | 6.445 | 495 |
| **Right precuneus** | 0.038 | 0.047 | [-0.054 - 0.129] | 0.374 | 0.420 | 0.989 | 3.987 | 494 |
| **Left insula** | 0.038 | 0.048 | [-0.057 - 0.133] | 0.400 | 0.429 | 0.989 | 7.150 | 494 |
| **Left pars orbitalis** | 0.040 | 0.095 | [-0.146 - 0.227] | 0.592 | 0.671 | 0.989 | 73.830 | 496 |
| **Right fusiform gyrus** | 0.041 | 0.045 | [-0.047 - 0.128] | 0.396 | 0.365 | 0.989 | <0.001 | 493 |
| **Left medial orbitofrontal cortex** | 0.041 | 0.093 | [-0.142 - 0.224] | 0.640 | 0.659 | 0.989 | 72.412 | 494 |
| **Right postcentral gyrus** | 0.042 | 0.076 | [-0.107 - 0.191] | 0.473 | 0.582 | 0.989 | 56.580 | 496 |
| **Left rostral middle frontal gyrus** | 0.046 | 0.045 | [-0.042 - 0.134] | 0.449 | 0.303 | 0.989 | <0.001 | 496 |
| **Left hempisphere average thickness** | 0.047 | 0.072 | [-0.095 - 0.189] | 0.347 | 0.515 | 0.989 | 51.282 | 496 |
| **Right banks superior temporal sulcus** | 0.047 | 0.086 | [-0.122 - 0.216] | 0.672 | 0.585 | 0.989 | 66.359 | 489 |
| **Left precuneus** | 0.049 | 0.045 | [-0.039 - 0.137] | 0.470 | 0.272 | 0.989 | <0.001 | 495 |
| **Right pars orbitalis** | 0.052 | 0.080 | [-0.105 - 0.208] | 0.747 | 0.517 | 0.989 | 60.371 | 495 |
| **Left rostral anterior cingulate cortex** | 0.053 | 0.086 | [-0.116 - 0.221] | 1.028 | 0.540 | 0.989 | 66.181 | 493 |
| **Left inferior temporal gyrus** | 0.059 | 0.076 | [-0.091 - 0.209] | 0.661 | 0.439 | 0.989 | 53.502 | 478 |
| **Left frontal pole** | 0.062 | 0.057 | [-0.05 - 0.174] | 1.313 | 0.277 | 0.989 | 26.298 | 496 |
| **Right inferior temporal gyrus** | 0.064 | 0.078 | [-0.088 - 0.217] | 0.697 | 0.410 | 0.989 | 57.199 | 487 |
| **Left lateral orbitofrontal cortex** | 0.066 | 0.088 | [-0.107 - 0.239] | 0.801 | 0.453 | 0.989 | 69.508 | 496 |
| **Right entorhinal cortex** | 0.068 | 0.045 | [-0.021 - 0.157] | 1.627 | 0.132 | 0.989 | <0.001 | 472 |
| **Left temporal pole** | 0.070 | 0.076 | [-0.078 - 0.218] | 1.418 | 0.354 | 0.989 | 54.661 | 485 |
| **Left caudal anterior cingulate cortex** | 0.070 | 0.083 | [-0.093 - 0.234] | 1.595 | 0.401 | 0.989 | 64.984 | 495 |
| **Left posterior cingulate cortex** | 0.072 | 0.066 | [-0.058 - 0.202] | 0.901 | 0.278 | 0.989 | 42.602 | 495 |
| **Right cuneus** | 0.072 | 0.045 | [-0.016 - 0.16] | 0.888 | 0.107 | 0.989 | <0.001 | 494 |
| **Left cuneus** | 0.075 | 0.045 | [-0.014 - 0.163] | 0.924 | 0.097 | 0.989 | <0.001 | 494 |
| **Left isthmus cingulate cortex** | 0.077 | 0.080 | [-0.079 - 0.234] | 1.194 | 0.332 | 0.989 | 61.704 | 494 |
| **Left middle temporal gyrus** | 0.089 | 0.112 | [-0.131 - 0.308] | 1.056 | 0.428 | 0.989 | 79.548 | 460 |
| **Left fusiform gyrus** | 0.093 | 0.055 | [-0.014 - 0.2] | 0.919 | 0.088 | 0.989 | 21.706 | 493 |
| **Left pericalcarine cortex** | 0.101 | 0.077 | [-0.05 - 0.252] | 1.423 | 0.192 | 0.989 | 58.002 | 492 |
| **Right transverse temporal gyrus** | 0.110 | 0.077 | [-0.042 - 0.262] | 1.783 | 0.155 | 0.989 | 60.137 | 496 |
| **Left entorhinal cortex** | 0.131 | 0.101 | [-0.067 - 0.329] | 3.034 | 0.196 | 0.989 | 76.576 | 475 |
| **Right temporal pole** | 0.145 | 0.111 | [-0.072 - 0.362] | 3.223 | 0.190 | 0.989 | 82.240 | 483 |
| **Right rostral anterior cingulate cortex** | 0.155 | 0.102 | [-0.045 - 0.355] | 3.174 | 0.130 | 0.989 | 80.180 | 494 |
| **Right middle temporal gyrus** | 0.158 | 0.084 | [-0.005 - 0.322] | 1.788 | 0.058 | 0.989 | 65.816 | 489 |

**^a^** Included Samples: CLING, Houston, Sexpect, Muenster Cohort, NESDA, Novosibirsk, DepOx, Stanford, Sydney.

MDD: Major Depressive Disorder.

**Supplementary Table S11**: Full meta-analytic results for thickness of each structure for MDD patients with an adult age of onset (>21) versus Controls comparison controlling for age, sex and scan center. Adjusted Cohen's d is reported.

|  | **Cohen's d ^a^** | **Std. Err.** | **95% CI** | **% Difference** | **P-value** | **FDR P-value** | **I^2^** | **# Controls** | **# Patients** |
| --- | --- | --- | --- | --- | --- | --- | --- | --- | --- |
|  | **(Adult-onset MDD vs CTL)** |  |  |  |  |  |  |  |  |
| **Right insula** | -0.182 | 0.044 | [-0.269 - -0.095] | -0.991 | 4.01E-05 | 0.002 | 19.366 | 3323 | 1210 |
| **Left rostral anterior cingulate cortex** | -0.180 | 0.055 | [-0.288 - -0.071] | -1.749 | 0.001 | 0.012 | 42.557 | 3327 | 1210 |
| **Left insula** | -0.158 | 0.042 | [-0.24 - -0.076] | -0.825 | 1.51E-04 | 0.003 | 13.815 | 3324 | 1213 |
| **Right fusiform gyrus** | -0.149 | 0.056 | [-0.259 - -0.039] | -0.726 | 0.008 | 0.041 | 43.929 | 3320 | 1213 |
| **Left medial orbitofrontal cortex** | -0.147 | 0.044 | [-0.233 - -0.062] | -1.142 | 7.18E-04 | 0.008 | 17.518 | 3283 | 1204 |
| **Left isthmus cingulate cortex** | -0.144 | 0.054 | [-0.249 - -0.039] | -1.109 | 0.007 | 0.039 | 39.369 | 3326 | 1211 |
| **Right posterior cingulate cortex** | -0.140 | 0.035 | [-0.209 - -0.07] | -0.934 | 8.00E-05 | 0.002 | 0.004 | 3326 | 1214 |
| **Left fusiform gyrus** | -0.138 | 0.035 | [-0.207 - -0.069] | -0.678 | 9.75E-05 | 0.002 | <0.001 | 3317 | 1212 |
| **Right medial orbitofrontal cortex** | -0.134 | 0.043 | [-0.218 - -0.05] | -1.125 | 0.002 | 0.013 | 15.938 | 3299 | 1210 |
| **Right inferior temporal gyrus** | -0.124 | 0.043 | [-0.207 - -0.04] | -0.670 | 0.004 | 0.021 | 15.364 | 3311 | 1208 |
| **Right isthmus cingulate cortex** | -0.123 | 0.035 | [-0.193 - -0.054] | -0.976 | 5.04E-04 | 0.007 | 0.027 | 3324 | 1211 |
| **Left pars orbitalis** | -0.119 | 0.058 | [-0.233 - -0.005] | -0.870 | 0.042 | 0.147 | 47.756 | 3325 | 1214 |
| **Right rostral anterior cingulate cortex** | -0.112 | 0.037 | [-0.185 - -0.039] | -1.137 | 0.003 | 0.017 | 4.023 | 3322 | 1213 |
| **Left posterior cingulate cortex** | -0.112 | 0.035 | [-0.181 - -0.042] | -0.699 | 0.002 | 0.013 | 0.003 | 3326 | 1213 |
| **Right lateral orbitofrontal cortex** | -0.111 | 0.045 | [-0.199 - -0.023] | -0.652 | 0.013 | 0.062 | 20.643 | 3316 | 1214 |
| **Right caudal anterior cingulate cortex** | -0.108 | 0.035 | [-0.178 - -0.039] | -1.207 | 0.002 | 0.015 | <0.001 | 3327 | 1213 |
| **Right supramarginal gyrus** | -0.094 | 0.054 | [-0.199 - 0.012] | -0.481 | 0.082 | 0.200 | 39.066 | 3306 | 1197 |
| **Right superior frontal gyrus** | -0.090 | 0.040 | [-0.168 - -0.012] | -0.437 | 0.023 | 0.102 | 9.396 | 3320 | 1213 |
| **Right middle temporal gyrus** | -0.090 | 0.044 | [-0.176 - -0.003] | -0.500 | 0.042 | 0.147 | 18.868 | 3312 | 1206 |
| **Right pars orbitalis** | -0.088 | 0.052 | [-0.189 - 0.013] | -0.638 | 0.086 | 0.200 | 35.213 | 3326 | 1214 |
| **Left superior frontal gyrus** | -0.088 | 0.042 | [-0.171 - -0.006] | -0.447 | 0.037 | 0.147 | 14.990 | 3323 | 1211 |
| **Left lateral orbitofrontal cortex** | -0.085 | 0.052 | [-0.186 - 0.016] | -0.512 | 0.100 | 0.226 | 35.150 | 3309 | 1210 |
| **Left frontal pole** | -0.083 | 0.048 | [-0.178 - 0.012] | -0.876 | 0.085 | 0.200 | 28.428 | 3327 | 1212 |
| **Left middle temporal gyrus** | -0.083 | 0.041 | [-0.163 - -0.003] | -0.493 | 0.041 | 0.147 | 9.933 | 3265 | 1168 |
| **Left inferior parietal cortex** | -0.079 | 0.067 | [-0.21 - 0.051] | -0.451 | 0.233 | 0.355 | 59.675 | 3309 | 1210 |
| **Right pars triangularis** | -0.078 | 0.056 | [-0.187 - 0.032] | -0.447 | 0.166 | 0.291 | 43.567 | 3316 | 1211 |
| **Right inferior parietal cortex** | -0.077 | 0.063 | [-0.201 - 0.047] | -0.436 | 0.224 | 0.349 | 55.322 | 3312 | 1210 |
| **Left hemisphere average thickness** | -0.076 | 0.043 | [-0.159 - 0.008] | -0.278 | 0.077 | 0.198 | 16.070 | 3329 | 1214 |
| **Right banks superior temporal sulcus** | -0.074 | 0.041 | [-0.154 - 0.007] | -0.524 | 0.074 | 0.198 | 11.684 | 3288 | 1163 |
| **Left caudal anterior cingulate cortex** | -0.073 | 0.040 | [-0.152 - 0.006] | -0.824 | 0.072 | 0.198 | 10.662 | 3321 | 1212 |
| **Left pars opercularis** | -0.069 | 0.035 | [-0.139 - 0] | -0.330 | 0.050 | 0.168 | 0.015 | 3326 | 1210 |
| **Left banks superior temporal sulcus** | -0.069 | 0.036 | [-0.14 - 0.002] | -0.507 | 0.056 | 0.177 | <0.001 | 3246 | 1139 |
| **Right hemisphere average thickness** | -0.069 | 0.043 | [-0.154 - 0.016] | -0.252 | 0.110 | 0.240 | 17.395 | 3329 | 1214 |
| **Left inferior temporal gyrus** | -0.062 | 0.040 | [-0.139 - 0.016] | -0.346 | 0.118 | 0.243 | 8.389 | 3301 | 1198 |
| **Right pars opercularis** | -0.062 | 0.041 | [-0.141 - 0.018] | -0.309 | 0.128 | 0.257 | 11.046 | 3323 | 1212 |
| **Left supramarginal gyrus** | -0.057 | 0.041 | [-0.137 - 0.024] | -0.307 | 0.165 | 0.291 | 11.400 | 3282 | 1189 |
| **Left parahippocampal gyrus** | -0.056 | 0.035 | [-0.125 - 0.013] | -0.698 | 0.113 | 0.240 | 0.001 | 3322 | 1212 |
| **Right transverse temporal gyrus** | -0.055 | 0.039 | [-0.133 - 0.022] | -0.446 | 0.159 | 0.291 | 8.500 | 3293 | 1207 |
| **Left lateral occipital cortex** | -0.052 | 0.055 | [-0.161 - 0.056] | -0.302 | 0.343 | 0.490 | 42.327 | 3316 | 1211 |
| **Right parahippocampal gyrus** | -0.052 | 0.035 | [-0.121 - 0.017] | -0.566 | 0.143 | 0.277 | 0.030 | 3325 | 1211 |
| **Left precuneus** | -0.051 | 0.058 | [-0.165 - 0.063] | -0.242 | 0.384 | 0.538 | 47.374 | 3320 | 1210 |
| **Left transverse temporal gyrus** | -0.048 | 0.035 | [-0.118 - 0.021] | -0.379 | 0.174 | 0.297 | <0.001 | 3307 | 1209 |
| **Right frontal pole** | -0.043 | 0.035 | [-0.112 - 0.026] | -0.449 | 0.224 | 0.349 | 0.001 | 3329 | 1211 |
| **Right rostral middle frontal gyrus** | -0.043 | 0.051 | [-0.143 - 0.057] | -0.205 | 0.404 | 0.555 | 34.158 | 3321 | 1212 |
| **Right entorhinal cortex** | -0.040 | 0.038 | [-0.113 - 0.034] | -0.470 | 0.292 | 0.426 | 4.115 | 3275 | 1193 |
| **Right superior temporal gyrus** | -0.039 | 0.036 | [-0.11 - 0.031] | -0.235 | 0.273 | 0.407 | <0.001 | 3265 | 1170 |
| **Left caudal middle frontal gyrus** | -0.034 | 0.048 | [-0.127 - 0.06] | -0.172 | 0.480 | 0.646 | 27.405 | 3318 | 1212 |
| **Left entorhinal cortex** | -0.030 | 0.052 | [-0.131 - 0.071] | -0.351 | 0.554 | 0.691 | 34.871 | 3278 | 1194 |
| **Left pars triangularis** | -0.024 | 0.035 | [-0.093 - 0.045] | -0.146 | 0.498 | 0.658 | 0.007 | 3322 | 1210 |
| **Left rostral middle frontal gyrus** | -0.022 | 0.035 | [-0.091 - 0.048] | -0.106 | 0.537 | 0.691 | <0.001 | 3325 | 1211 |
| **Right paracentral lobule** | -0.020 | 0.035 | [-0.09 - 0.049] | -0.116 | 0.563 | 0.691 | <0.001 | 3323 | 1213 |
| **Right caudal middle frontal gyrus** | -0.019 | 0.043 | [-0.104 - 0.066] | -0.102 | 0.654 | 0.789 | 17.707 | 3321 | 1213 |
| **Right precentral gyrus** | -0.012 | 0.042 | [-0.095 - 0.07] | -0.074 | 0.769 | 0.883 | 15.181 | 3315 | 1212 |
| **Right precuneus** | -0.007 | 0.047 | [-0.1 - 0.085] | -0.036 | 0.877 | 0.945 | 26.061 | 3320 | 1210 |
| **Left superior temporal gyrus** | -0.006 | 0.036 | [-0.077 - 0.065] | -0.038 | 0.864 | 0.945 | 0.011 | 3225 | 1164 |
| **Right lateral occipital cortex** | -0.005 | 0.052 | [-0.106 - 0.096] | -0.029 | 0.923 | 0.969 | 35.481 | 3321 | 1212 |
| **Left paracentral lobule** | -0.003 | 0.045 | [-0.092 - 0.085] | -0.020 | 0.939 | 0.969 | 21.874 | 3321 | 1212 |
| **Left temporal pole** | -0.002 | 0.042 | [-0.085 - 0.08] | -0.024 | 0.955 | 0.969 | 14.124 | 3285 | 1203 |
| **Left precentral gyrus** | -0.002 | 0.035 | [-0.072 - 0.067] | -0.012 | 0.951 | 0.969 | <0.001 | 3309 | 1212 |
| **Right postcentral gyrus** | -0.002 | 0.051 | [-0.101 - 0.098] | -0.009 | 0.976 | 0.976 | 34.194 | 3313 | 1211 |
| **Right lingual gyrus** | 0.007 | 0.035 | [-0.063 - 0.076] | 0.038 | 0.851 | 0.945 | <0.001 | 3314 | 1209 |
| **Right temporal pole** | 0.011 | 0.048 | [-0.082 - 0.104] | 0.124 | 0.812 | 0.917 | 26.232 | 3303 | 1204 |
| **Right superior parietal cortex** | 0.020 | 0.060 | [-0.098 - 0.138] | 0.099 | 0.743 | 0.867 | 50.725 | 3317 | 1212 |
| **Left lingual gyrus** | 0.021 | 0.035 | [-0.048 - 0.091] | 0.122 | 0.552 | 0.691 | 0.003 | 3311 | 1209 |
| **Left superior parietal cortex** | 0.023 | 0.054 | [-0.083 - 0.13] | 0.114 | 0.670 | 0.795 | 40.851 | 3317 | 1211 |
| **Left postcentral gyrus** | 0.049 | 0.035 | [-0.02 - 0.119] | 0.271 | 0.166 | 0.291 | <0.001 | 3301 | 1208 |
| **Right cuneus** | 0.065 | 0.035 | [-0.005 - 0.134] | 0.398 | 0.067 | 0.196 | <0.001 | 3327 | 1210 |
| **Left cuneus** | 0.066 | 0.035 | [-0.003 - 0.136] | 0.409 | 0.061 | 0.186 | <0.001 | 3323 | 1210 |
| **Left pericalcarine cortex** | 0.084 | 0.065 | [-0.044 - 0.211] | 0.587 | 0.199 | 0.324 | 57.625 | 3316 | 1208 |
| **Right pericalcarine cortex** | 0.105 | 0.080 | [-0.053 - 0.262] | 0.764 | 0.192 | 0.320 | 72.943 | 3308 | 1210 |

**^a^** Included Samples: CLING, Imaging Genetics Dublin, Clinical Depression Dublin, Bipolar Family Study, Houston, Sexpect, MMDP 3T, MPIP, Muenster Cohort, NESDA, Novosibirsk, DepOx, QTIM, SHIP, SHIP-trend, Sydney, Stanford.

MDD: Major Depressive Disorder; CTL: Controls.

**Supplementary Table S12**: Full meta-analytic results for thickness of each structure for MDD patients with an adolescent age of onset (≤21) versus Controls comparison controlling for age, sex and scan center. Adjusted Cohen's d is reported.

|  | **Cohen's d ^a^** | **Std. Err.** | **95% CI** | **% Difference** | **P-value** | **FDR P-value** | **I^2^** | **# Controls** | **# Patients** |
| --- | --- | --- | --- | --- | --- | --- | --- | --- | --- |
|  | **(adolescent-onset MDD vs CTL)** |  |  |  |  |  |  |  |  |
| **Right inferior temporal gyrus** | -0.184 | 0.057 | [-0.295 - -0.073] | -0.998 | 0.001 | 0.080 | 7.018 | 2867 | 463 |
| **Right medial orbitofrontal cortex** | -0.148 | 0.089 | [-0.323 - 0.026] | -1.246 | 0.095 | 0.426 | 59.529 | 2859 | 470 |
| **Right pars orbitalis** | -0.145 | 0.079 | [-0.299 - 0.009] | -1.043 | 0.065 | 0.402 | 48.880 | 2884 | 472 |
| **Left pars orbitalis** | -0.142 | 0.053 | [-0.247 - -0.037] | -1.038 | 0.008 | 0.277 | <0.001 | 2880 | 472 |
| **Left rostral middle frontal gyrus** | -0.134 | 0.064 | [-0.259 - -0.008] | -0.648 | 0.036 | 0.331 | 24.948 | 2881 | 472 |
| **Left pars triangularis** | -0.124 | 0.053 | [-0.228 - -0.019] | -0.753 | 0.020 | 0.331 | <0.001 | 2879 | 472 |
| **Right banks superior temporal sulcus** | -0.120 | 0.055 | [-0.227 - -0.014] | -0.857 | 0.027 | 0.331 | 0.009 | 2847 | 452 |
| **Right inferior parietal cortex** | -0.116 | 0.053 | [-0.221 - -0.012] | -0.660 | 0.029 | 0.331 | <0.001 | 2870 | 471 |
| **Right fusiform gyrus** | -0.112 | 0.054 | [-0.217 - -0.007] | -0.548 | 0.036 | 0.331 | <0.001 | 2876 | 469 |
| **Right middle temporal gyrus** | -0.112 | 0.054 | [-0.217 - -0.006] | -0.623 | 0.038 | 0.331 | 0.004 | 2869 | 464 |
| **Right frontal pole** | -0.111 | 0.084 | [-0.275 - 0.054] | -1.153 | 0.187 | 0.563 | 54.937 | 2885 | 472 |
| **Left superior frontal gyrus** | -0.107 | 0.053 | [-0.211 - -0.002] | -0.539 | 0.046 | 0.358 | 0.008 | 2879 | 472 |
| **Left medial orbitofrontal cortex** | -0.103 | 0.062 | [-0.224 - 0.019] | -0.796 | 0.097 | 0.426 | 20.537 | 2842 | 470 |
| **Left middle temporal gyrus** | -0.102 | 0.054 | [-0.208 - 0.005] | -0.604 | 0.062 | 0.402 | <0.001 | 2833 | 450 |
| **Right rostral middle frontal gyrus** | -0.099 | 0.097 | [-0.29 - 0.092] | -0.476 | 0.312 | 0.650 | 66.614 | 2877 | 471 |
| **Left fusiform gyrus** | -0.098 | 0.054 | [-0.203 - 0.008] | -0.479 | 0.069 | 0.402 | <0.001 | 2873 | 468 |
| **Left inferior parietal cortex** | -0.094 | 0.053 | [-0.199 - 0.01] | -0.535 | 0.078 | 0.419 | <0.001 | 2867 | 471 |
| **Right superior frontal gyrus** | -0.094 | 0.066 | [-0.223 - 0.035] | -0.457 | 0.153 | 0.528 | 29.010 | 2876 | 472 |
| **Right lateral orbitofrontal cortex** | -0.094 | 0.066 | [-0.224 - 0.037] | -0.551 | 0.158 | 0.528 | 29.987 | 2872 | 472 |
| **Right supramarginal gyrus** | -0.092 | 0.055 | [-0.199 - 0.015] | -0.474 | 0.091 | 0.426 | 1.878 | 2864 | 464 |
| **Left isthmus cingulate cortex** | -0.090 | 0.089 | [-0.265 - 0.085] | -0.693 | 0.312 | 0.650 | 60.295 | 2882 | 472 |
| **Right precentral gyrus** | -0.090 | 0.056 | [-0.2 - 0.02] | -0.535 | 0.108 | 0.435 | 6.252 | 2871 | 469 |
| **Left pars opercularis** | -0.087 | 0.060 | [-0.204 - 0.03] | -0.416 | 0.144 | 0.528 | 15.804 | 2882 | 472 |
| **Left hemisphere average thickness** | -0.085 | 0.054 | [-0.19 - 0.02] | -0.313 | 0.112 | 0.435 | 0.594 | 2885 | 472 |
| **Right lateral occipital cortex** | -0.083 | 0.065 | [-0.211 - 0.045] | -0.486 | 0.205 | 0.573 | 28.107 | 2877 | 470 |
| **Left precentral gyrus** | -0.078 | 0.079 | [-0.232 - 0.076] | -0.447 | 0.322 | 0.650 | 48.737 | 2867 | 470 |
| **Right lingual gyrus** | -0.075 | 0.057 | [-0.187 - 0.038] | -0.432 | 0.193 | 0.563 | 9.808 | 2869 | 470 |
| **Left rostral anterior cingulate cortex** | -0.074 | 0.065 | [-0.201 - 0.053] | -0.718 | 0.255 | 0.650 | 27.006 | 2883 | 472 |
| **Right hemisphere average thickness** | -0.070 | 0.053 | [-0.175 - 0.034] | -0.256 | 0.189 | 0.563 | 0.002 | 2885 | 472 |
| **Left lateral occipital cortex** | -0.064 | 0.070 | [-0.2 - 0.073] | -0.366 | 0.362 | 0.650 | 36.049 | 2872 | 472 |
| **Left posterior cingulate cortex** | -0.063 | 0.061 | [-0.183 - 0.057] | -0.393 | 0.305 | 0.650 | 19.052 | 2882 | 471 |
| **Left superior parietal cortex** | -0.062 | 0.053 | [-0.167 - 0.043] | -0.304 | 0.244 | 0.650 | <0.001 | 2872 | 471 |
| **Left parahippocampal gyrus** | -0.061 | 0.075 | [-0.208 - 0.087] | -0.754 | 0.420 | 0.718 | 43.994 | 2879 | 471 |
| **Left lateral orbitofrontal cortex** | -0.060 | 0.081 | [-0.219 - 0.1] | -0.361 | 0.463 | 0.736 | 52.210 | 2865 | 472 |
| **Left precuneus** | -0.060 | 0.053 | [-0.164 - 0.045] | -0.285 | 0.264 | 0.650 | <0.001 | 2876 | 470 |
| **Right caudal anterior cingulate cortex** | -0.056 | 0.053 | [-0.161 - 0.049] | -0.624 | 0.294 | 0.650 | <0.001 | 2884 | 471 |
| **Left insula** | -0.052 | 0.053 | [-0.157 - 0.052] | -0.273 | 0.327 | 0.650 | <0.001 | 2883 | 472 |
| **Right precuneus** | -0.051 | 0.053 | [-0.156 - 0.053] | -0.254 | 0.338 | 0.650 | <0.001 | 2874 | 472 |
| **Left banks superior temporal sulcus** | -0.051 | 0.055 | [-0.158 - 0.057] | -0.372 | 0.355 | 0.650 | <0.001 | 2814 | 442 |
| **Right insula** | -0.049 | 0.053 | [-0.154 - 0.056] | -0.266 | 0.360 | 0.650 | 0.008 | 2884 | 472 |
| **Left supramarginal gyrus** | -0.048 | 0.063 | [-0.171 - 0.076] | -0.257 | 0.450 | 0.732 | 22.121 | 2845 | 467 |
| **Left inferior temporal gyrus** | -0.047 | 0.075 | [-0.193 - 0.099] | -0.262 | 0.530 | 0.757 | 42.184 | 2863 | 462 |
| **Left lingual gyrus** | -0.046 | 0.065 | [-0.172 - 0.081] | -0.264 | 0.480 | 0.739 | 26.273 | 2867 | 470 |
| **Left paracentral lobule** | -0.042 | 0.053 | [-0.146 - 0.063] | -0.238 | 0.433 | 0.722 | <0.001 | 2877 | 472 |
| **Right parahippocampal gyrus** | -0.037 | 0.053 | [-0.141 - 0.068] | -0.398 | 0.494 | 0.739 | <0.001 | 2882 | 471 |
| **Right entorhinal cortex** | -0.028 | 0.054 | [-0.133 - 0.078] | -0.328 | 0.609 | 0.813 | <0.001 | 2849 | 461 |
| **Right rostral anterior cingulate cortex** | -0.025 | 0.077 | [-0.176 - 0.127] | -0.249 | 0.750 | 0.893 | 47.134 | 2882 | 472 |
| **Left transverse temporal gyrus** | -0.024 | 0.053 | [-0.129 - 0.081] | -0.190 | 0.651 | 0.829 | <0.001 | 2863 | 471 |
| **Left postcentral gyrus** | -0.021 | 0.053 | [-0.126 - 0.084] | -0.117 | 0.691 | 0.858 | <0.001 | 2858 | 471 |
| **Right superior temporal gyrus** | -0.020 | 0.065 | [-0.148 - 0.108] | -0.119 | 0.761 | 0.893 | 23.212 | 2824 | 439 |
| **Left caudal middle frontal gyrus** | -0.014 | 0.077 | [-0.165 - 0.137] | -0.071 | 0.856 | 0.909 | 46.781 | 2874 | 471 |
| **Right pericalcarine cortex** | -0.013 | 0.066 | [-0.142 - 0.117] | -0.091 | 0.850 | 0.909 | 29.269 | 2863 | 471 |
| **Right isthmus cingulate cortex** | -0.012 | 0.067 | [-0.143 - 0.119] | -0.094 | 0.859 | 0.909 | 30.564 | 2881 | 472 |
| **Left entorhinal cortex** | -0.009 | 0.054 | [-0.115 - 0.096] | -0.106 | 0.864 | 0.909 | <0.001 | 2853 | 464 |
| **Right transverse temporal gyrus** | -0.009 | 0.061 | [-0.128 - 0.11] | -0.073 | 0.882 | 0.909 | 18.730 | 2849 | 472 |
| **Right superior parietal cortex** | -0.006 | 0.053 | [-0.111 - 0.099] | -0.030 | 0.911 | 0.911 | <0.001 | 2872 | 471 |
| **Right posterior cingulate cortex** | 7.64E-03 | 0.053 | [-0.097 - 0.112] | 0.051 | 0.886 | 0.909 | <0.001 | 2881 | 471 |
| **Right caudal middle frontal gyrus** | 0.010 | 0.075 | [-0.136 - 0.156] | 0.051 | 0.896 | 0.909 | 43.554 | 2877 | 472 |
| **Right pars opercularis** | 0.011 | 0.060 | [-0.108 - 0.129] | 0.055 | 0.856 | 0.909 | 17.358 | 2881 | 470 |
| **Right pars triangularis** | 0.011 | 0.068 | [-0.122 - 0.145] | 0.066 | 0.867 | 0.909 | 33.154 | 2876 | 471 |
| **Right paracentral lobule** | 0.016 | 0.055 | [-0.092 - 0.125] | 0.093 | 0.765 | 0.893 | 4.710 | 2880 | 472 |
| **Left caudal anterior cingulate cortex** | 0.025 | 0.053 | [-0.08 - 0.129] | 0.279 | 0.645 | 0.829 | <0.001 | 2878 | 472 |
| **Left cuneus** | 0.027 | 0.053 | [-0.078 - 0.131] | 0.165 | 0.616 | 0.813 | 0.001 | 2883 | 472 |
| **Right postcentral gyrus** | 0.027 | 0.053 | [-0.078 - 0.132] | 0.153 | 0.614 | 0.813 | <0.001 | 2869 | 471 |
| **Right cuneus** | 0.029 | 0.053 | [-0.076 - 0.134] | 0.178 | 0.587 | 0.813 | <0.001 | 2883 | 470 |
| **Left frontal pole** | 0.029 | 0.075 | [-0.118 - 0.176] | 0.306 | 0.699 | 0.858 | 44.220 | 2883 | 472 |
| **Right temporal pole** | 0.044 | 0.065 | [-0.083 - 0.172] | 0.487 | 0.496 | 0.739 | 25.279 | 2859 | 456 |
| **Left temporal pole** | 0.051 | 0.079 | [-0.105 - 0.206] | 0.513 | 0.523 | 0.757 | 48.370 | 2842 | 456 |
| **Left superior temporal gyrus** | 0.071 | 0.081 | [-0.087 - 0.23] | 0.436 | 0.378 | 0.661 | 48.488 | 2793 | 444 |
| **Left pericalcarine cortex** | 0.086 | 0.079 | [-0.068 - 0.241] | 0.608 | 0.273 | 0.650 | 49.119 | 2872 | 471 |

**^a^** Included Samples: Imaging Genetics Dublin, Houston, MMDP 3T, Melbourne, MPIP, Muenster Cohort, NESDA, QTIM, SHIP, SHIP-trend, Sydney, Stanford.

MDD: Major Depressive Disorder; CTL: Controls.

**Supplementary Table S13**: Full meta-analytic results for thickness of each structure for MDD patients with an adolescent age of onset (≤21) versus MDD patients with an adult age of onset (>21) comparison controlling for age, sex and scan center. Adjusted Cohen's d is reported.

|  | **Cohen's d ^a^** | **Std. Err.** | **95% CI** | **% Difference** | **P-value** | **FDR P-value** | **I^2^** | **# Adolescent-onset MDD** | **# Adult-onset MDD** |
| --- | --- | --- | --- | --- | --- | --- | --- | --- | --- |
|  | **(adolescent-onset MDD vs adult-onset MDD)** |  |  |  |  |  |  |  |  |
| **Right rostral anterior cingulate cortex** | -0.099 | 0.061 | [-0.219 - 0.021] | -1.004 | 0.106 | 0.826 | 3.801 | 462 | 1088 |
| **Right posterior cingulate cortex** | -0.080 | 0.063 | [-0.204 - 0.043] | -0.537 | 0.204 | 0.827 | 7.606 | 462 | 1088 |
| **Left caudal anterior cingulate cortex** | -0.077 | 0.060 | [-0.193 - 0.04] | -0.869 | 0.199 | 0.827 | <0.001 | 462 | 1087 |
| **Right caudal anterior cingulate cortex** | -0.062 | 0.059 | [-0.179 - 0.054] | -0.694 | 0.295 | 0.827 | <0.001 | 462 | 1088 |
| **Right insula** | -0.062 | 0.059 | [-0.179 - 0.054] | -0.339 | 0.295 | 0.827 | <0.001 | 462 | 1088 |
| **Left rostral anterior cingulate cortex** | -0.059 | 0.075 | [-0.205 - 0.087] | -0.572 | 0.431 | 0.839 | 29.133 | 462 | 1086 |
| **Left frontal pole** | -0.051 | 0.070 | [-0.189 - 0.087] | -0.539 | 0.467 | 0.839 | 22.092 | 462 | 1086 |
| **Left insula** | -0.033 | 0.059 | [-0.149 - 0.084] | -0.171 | 0.582 | 0.863 | <0.001 | 462 | 1088 |
| **Left temporal pole** | -0.029 | 0.081 | [-0.187 - 0.129] | -0.293 | 0.720 | 0.887 | 36.462 | 446 | 1077 |
| **Right pars opercularis** | -0.026 | 0.060 | [-0.143 - 0.091] | -0.130 | 0.663 | 0.876 | <0.001 | 460 | 1086 |
| **Right entorhinal cortex** | -0.021 | 0.060 | [-0.139 - 0.096] | -0.253 | 0.722 | 0.887 | <0.001 | 452 | 1080 |
| **Right paracentral lobule** | -0.017 | 0.062 | [-0.139 - 0.104] | -0.099 | 0.779 | 0.926 | 5.928 | 462 | 1087 |
| **Left lateral orbitofrontal cortex** | -0.010 | 0.090 | [-0.188 - 0.167] | -0.062 | 0.910 | 0.946 | 50.099 | 462 | 1084 |
| **Right pars triangularis** | -0.009 | 0.060 | [-0.125 - 0.108] | -0.051 | 0.882 | 0.946 | <0.001 | 461 | 1086 |
| **Right lateral orbitofrontal cortex** | -0.006 | 0.059 | [-0.123 - 0.11] | -0.036 | 0.919 | 0.946 | <0.001 | 462 | 1088 |
| **Left entorhinal cortex** | -0.002 | 0.061 | [-0.121 - 0.116] | -0.027 | 0.969 | 0.969 | 1.684 | 455 | 1081 |
| **Right isthmus cingulate cortex** | 0.004 | 0.059 | [-0.113 - 0.12] | 0.029 | 0.950 | 0.964 | <0.001 | 462 | 1086 |
| **Left banks superior temporal sulcus** | 0.008 | 0.061 | [-0.112 - 0.128] | 0.058 | 0.896 | 0.946 | <0.001 | 432 | 1026 |
| **Left pericalcarine cortex** | 0.008 | 0.070 | [-0.129 - 0.146] | 0.058 | 0.907 | 0.946 | 21.261 | 461 | 1085 |
| **Right temporal pole** | 0.010 | 0.068 | [-0.123 - 0.143] | 0.111 | 0.882 | 0.946 | 15.678 | 446 | 1078 |
| **Left lateral occipital cortex** | 0.011 | 0.094 | [-0.173 - 0.195] | 0.061 | 0.910 | 0.946 | 53.436 | 462 | 1086 |
| **Left supramarginal gyrus** | 0.011 | 0.060 | [-0.107 - 0.128] | 0.058 | 0.858 | 0.946 | <0.001 | 457 | 1072 |
| **Left posterior cingulate cortex** | 0.013 | 0.059 | [-0.103 - 0.13] | 0.084 | 0.822 | 0.946 | <0.001 | 462 | 1088 |
| **Right parahippocampal gyrus** | 0.017 | 0.059 | [-0.1 - 0.133] | 0.180 | 0.781 | 0.926 | <0.001 | 461 | 1087 |
| **Left pars opercularis** | 0.022 | 0.060 | [-0.094 - 0.139] | 0.107 | 0.706 | 0.887 | 0.010 | 462 | 1086 |
| **Right supramarginal gyrus** | 0.026 | 0.060 | [-0.091 - 0.143] | 0.134 | 0.663 | 0.876 | <0.001 | 455 | 1072 |
| **Right transverse temporal gyrus** | 0.029 | 0.074 | [-0.115 - 0.174] | 0.236 | 0.691 | 0.887 | 27.988 | 462 | 1081 |
| **Left caudal middle frontal gyrus** | 0.030 | 0.060 | [-0.087 - 0.146] | 0.151 | 0.620 | 0.868 | 0.005 | 461 | 1086 |
| **Left transverse temporal gyrus** | 0.031 | 0.060 | [-0.086 - 0.148] | 0.243 | 0.604 | 0.863 | <0.001 | 461 | 1083 |
| **Right postcentral gyrus** | 0.032 | 0.060 | [-0.084 - 0.149] | 0.182 | 0.588 | 0.863 | <0.001 | 461 | 1085 |
| **Left medial orbitofrontal cortex** | 0.035 | 0.060 | [-0.082 - 0.151] | 0.269 | 0.560 | 0.863 | <0.001 | 460 | 1079 |
| **Right fusiform gyrus** | 0.039 | 0.060 | [-0.078 - 0.156] | 0.190 | 0.513 | 0.863 | <0.001 | 459 | 1087 |
| **Left inferior parietal cortex** | 0.042 | 0.061 | [-0.078 - 0.161] | 0.237 | 0.493 | 0.863 | 3.000 | 461 | 1084 |
| **Left parahippocampal gyrus** | 0.043 | 0.073 | [-0.099 - 0.185] | 0.534 | 0.555 | 0.863 | 25.793 | 462 | 1086 |
| **Left isthmus cingulate cortex** | 0.043 | 0.081 | [-0.116 - 0.203] | 0.332 | 0.596 | 0.863 | 39.347 | 462 | 1087 |
| **Left cuneus** | 0.045 | 0.093 | [-0.138 - 0.228] | 0.276 | 0.632 | 0.868 | 53.124 | 462 | 1087 |
| **Left fusiform gyrus** | 0.046 | 0.060 | [-0.071 - 0.162] | 0.224 | 0.445 | 0.839 | <0.001 | 458 | 1086 |
| **Right superior frontal gyrus** | 0.046 | 0.059 | [-0.07 - 0.163] | 0.224 | 0.438 | 0.839 | <0.001 | 462 | 1087 |
| **Right banks superior temporal sulcus** | 0.050 | 0.061 | [-0.069 - 0.169] | 0.359 | 0.407 | 0.839 | 0.007 | 444 | 1041 |
| **Left superior frontal gyrus** | 0.053 | 0.059 | [-0.063 - 0.17] | 0.269 | 0.371 | 0.839 | 0.011 | 462 | 1086 |
| **Right middle temporal gyrus** | 0.054 | 0.060 | [-0.063 - 0.171] | 0.302 | 0.366 | 0.839 | 0.002 | 455 | 1081 |
| **Right caudal middle frontal gyrus** | 0.055 | 0.059 | [-0.062 - 0.171] | 0.288 | 0.358 | 0.839 | <0.001 | 462 | 1087 |
| **Left inferior temporal gyrus** | 0.055 | 0.075 | [-0.092 - 0.203] | 0.309 | 0.462 | 0.839 | 28.627 | 452 | 1078 |
| **Right lingual gyrus** | 0.056 | 0.075 | [-0.092 - 0.203] | 0.324 | 0.457 | 0.839 | 30.034 | 460 | 1084 |
| **Right pericalcarine cortex** | 0.057 | 0.102 | [-0.143 - 0.256] | 0.413 | 0.577 | 0.863 | 60.219 | 461 | 1087 |
| **Left paracentral lobule** | 0.057 | 0.059 | [-0.06 - 0.173] | 0.323 | 0.340 | 0.839 | <0.001 | 462 | 1086 |
| **Left superior temporal gyrus** | 0.057 | 0.089 | [-0.118 - 0.232] | 0.348 | 0.523 | 0.863 | 45.791 | 434 | 1047 |
| **Right superior parietal cortex** | 0.065 | 0.078 | [-0.089 - 0.219] | 0.324 | 0.408 | 0.839 | 35.128 | 461 | 1088 |
| **Right medial orbitofrontal cortex** | 0.069 | 0.062 | [-0.052 - 0.189] | 0.577 | 0.264 | 0.827 | 4.342 | 460 | 1085 |
| **Left precuneus** | 0.070 | 0.060 | [-0.046 - 0.187] | 0.336 | 0.237 | 0.827 | 0.005 | 461 | 1084 |
| **Right frontal pole** | 0.071 | 0.088 | [-0.1 - 0.243] | 0.745 | 0.415 | 0.839 | 47.145 | 462 | 1085 |
| **Right hemisphere average thickness** | 0.072 | 0.066 | [-0.057 - 0.2] | 0.262 | 0.274 | 0.827 | 12.936 | 462 | 1088 |
| **Right superior temporal gyrus** | 0.072 | 0.061 | [-0.048 - 0.192] | 0.433 | 0.237 | 0.827 | 0.020 | 430 | 1048 |
| **Right pars orbitalis** | 0.072 | 0.063 | [-0.051 - 0.196] | 0.521 | 0.251 | 0.827 | 7.718 | 462 | 1088 |
| **Left middle temporal gyrus** | 0.078 | 0.061 | [-0.041 - 0.197] | 0.463 | 0.199 | 0.827 | <0.001 | 440 | 1054 |
| **Right lateral occipital cortex** | 0.078 | 0.094 | [-0.106 - 0.263] | 0.458 | 0.406 | 0.839 | 53.857 | 460 | 1086 |
| **Right cuneus** | 0.079 | 0.059 | [-0.037 - 0.196] | 0.487 | 0.182 | 0.827 | <0.001 | 461 | 1087 |
| **Left pars orbitalis** | 0.088 | 0.059 | [-0.029 - 0.204] | 0.640 | 0.141 | 0.827 | 0.019 | 462 | 1088 |
| **Left hemisphere average thickness** | 0.090 | 0.061 | [-0.029 - 0.209] | 0.331 | 0.137 | 0.827 | 2.275 | 462 | 1088 |
| **Right precuneus** | 0.090 | 0.065 | [-0.038 - 0.218] | 0.448 | 0.167 | 0.827 | 11.931 | 462 | 1084 |
| **Right inferior temporal gyrus** | 0.091 | 0.082 | [-0.069 - 0.252] | 0.496 | 0.265 | 0.827 | 39.018 | 453 | 1082 |
| **Right inferior parietal cortex** | 0.095 | 0.068 | [-0.039 - 0.229] | 0.540 | 0.164 | 0.827 | 18.162 | 461 | 1084 |
| **Left lingual gyrus** | 0.101 | 0.060 | [-0.015 - 0.218] | 0.587 | 0.089 | 0.775 | 0.009 | 461 | 1083 |
| **Right precentral gyrus** | 0.103 | 0.060 | [-0.014 - 0.22] | 0.613 | 0.083 | 0.775 | <0.001 | 460 | 1087 |
| **Left postcentral gyrus** | 0.104 | 0.060 | [-0.013 - 0.221] | 0.574 | 0.081 | 0.775 | 0.002 | 461 | 1084 |
| **Left pars triangularis** | 0.125 | 0.060 | [0.008 - 0.241] | 0.758 | 0.036 | 0.611 | <0.001 | 462 | 1086 |
| **Left rostral middle frontal gyrus** | 0.132 | 0.060 | [0.016 - 0.249] | 0.641 | 0.026 | 0.611 | <0.001 | 462 | 1085 |
| **Left superior parietal cortex** | 0.141 | 0.060 | [0.024 - 0.258] | 0.692 | 0.018 | 0.611 | 0.011 | 461 | 1086 |
| **Left precentral gyrus** | 0.148 | 0.060 | [0.031 - 0.265] | 0.850 | 0.013 | 0.611 | 0.003 | 461 | 1086 |
| **Right rostral middle frontal gyrus** | 0.161 | 0.080 | [0.005 - 0.318] | 0.778 | 0.044 | 0.611 | 36.955 | 461 | 1086 |

**^a^** Included Samples: Imaging Genetics Dublin, Houston, MMDP 3T, MPIP, Muenster Cohort, NESDA, QTIM, SHIP, SHIP-trend, Sydney, Stanford.

MDD: Major Depressive Disorder; CTL: Controls.

**Supplementary Table S14**: Full meta-analytic results for thickness of each structure for MDD patients taking antidepressants at time of scanning versus Controls comparison controlling for age, sex and scan center. Adjusted Cohen's d is reported.

|  | **Cohen's d ^a^** | **Std. Err.** | **95% CI** | **% Difference** | **P-value** | **FDR P-value** | **I^2^** | **# Controls** | **# Patients** |
| --- | --- | --- | --- | --- | --- | --- | --- | --- | --- |
|  | **(AD MDD vs CTL)** |  |  |  |  |  |  |  |  |
| **Right medial orbitofrontal cortex** | -0.261 | 0.064 | [-0.387 - -0.135] | -2.191 | 4.66E-05 | 9.73E-04 | 45.245 | 7203 | 948 |
| **Left pars opercularis** | -0.224 | 0.086 | [-0.393 - -0.056] | -1.070 | 0.009 | 0.028 | 70.422 | 7221 | 950 |
| **Left parahippocampal gyrus** | -0.222 | 0.097 | [-0.413 - -0.032] | -2.765 | 0.022 | 0.058 | 77.126 | 7217 | 950 |
| **Right posterior cingulate cortex** | -0.219 | 0.069 | [-0.354 - -0.085] | -1.467 | 0.001 | 0.009 | 52.825 | 7220 | 951 |
| **Right rostral anterior cingulate cortex** | -0.219 | 0.068 | [-0.352 - -0.086] | -2.221 | 0.001 | 0.009 | 50.976 | 7216 | 949 |
| **Left rostral anterior cingulate cortex** | -0.216 | 0.050 | [-0.314 - -0.118] | -2.104 | 1.71E-05 | 5.99E-04 | 18.831 | 7221 | 947 |
| **Left insula** | -0.214 | 0.068 | [-0.348 - -0.081] | -1.119 | 0.002 | 0.010 | 51.990 | 7218 | 949 |
| **Right lateral orbitofrontal cortex** | -0.203 | 0.090 | [-0.379 - -0.026] | -1.192 | 0.024 | 0.061 | 73.054 | 7210 | 951 |
| **Left fusiform gyrus** | -0.201 | 0.050 | [-0.299 - -0.103] | -0.989 | 5.56E-05 | 9.73E-04 | 18.330 | 7212 | 950 |
| **Left posterior cingulate cortex** | -0.197 | 0.076 | [-0.346 - -0.049] | -1.236 | 0.009 | 0.028 | 61.335 | 7220 | 951 |
| **Right isthmus cingulate cortex** | -0.193 | 0.068 | [-0.326 - -0.059] | -1.526 | 0.005 | 0.017 | 51.812 | 7218 | 949 |
| **Left isthmus cingulate cortex** | -0.191 | 0.110 | [-0.407 - 0.025] | -1.467 | 0.084 | 0.146 | 82.590 | 7221 | 948 |
| **Right insula** | -0.185 | 0.041 | [-0.266 - -0.104] | -1.010 | 0.000 | 4.98E-04 | <0.001 | 7217 | 946 |
| **Right inferior temporal gyrus** | -0.162 | 0.048 | [-0.256 - -0.067] | -0.878 | 8.16E-04 | 0.007 | 14.538 | 7206 | 947 |
| **Left caudal anterior cingulate cortex** | -0.155 | 0.086 | [-0.324 - 0.014] | -1.760 | 0.073 | 0.137 | 70.625 | 7215 | 950 |
| **Right fusiform gyrus** | -0.150 | 0.048 | [-0.245 - -0.055] | -0.730 | 0.002 | 0.011 | 15.069 | 7215 | 950 |
| **Right superior frontal gyrus** | -0.149 | 0.041 | [-0.229 - -0.068] | -0.720 | 3.11E-04 | 0.004 | <0.001 | 7214 | 951 |
| **Right parahippocampal gyrus** | -0.147 | 0.079 | [-0.301 - 0.007] | -1.602 | 0.061 | 0.126 | 64.115 | 7219 | 947 |
| **Left medial orbitofrontal cortex** | -0.146 | 0.041 | [-0.227 - -0.065] | -1.131 | 4.25E-04 | 0.005 | <0.001 | 7187 | 944 |
| **Right caudal anterior cingulate cortex** | -0.144 | 0.041 | [-0.225 - -0.063] | -1.603 | 4.77E-04 | 0.005 | <0.001 | 7221 | 949 |
| **Right supramarginal gyrus** | -0.140 | 0.049 | [-0.237 - -0.044] | -0.721 | 0.004 | 0.016 | 15.785 | 7202 | 931 |
| **Left pars orbitalis** | -0.134 | 0.056 | [-0.244 - -0.024] | -0.981 | 0.017 | 0.048 | 31.742 | 7219 | 951 |
| **Left middle temporal gyrus** | -0.134 | 0.042 | [-0.216 - -0.052] | -0.796 | 0.001 | 0.009 | <0.001 | 7166 | 908 |
| **Left pars triangularis** | -0.129 | 0.049 | [-0.226 - -0.033] | -0.788 | 0.009 | 0.028 | 17.365 | 7216 | 949 |
| **Left rostral middle frontal gyrus** | -0.125 | 0.071 | [-0.265 - 0.014] | -0.607 | 0.078 | 0.143 | 55.929 | 7220 | 950 |
| **Left superior frontal gyrus** | -0.125 | 0.041 | [-0.205 - -0.044] | -0.630 | 0.002 | 0.012 | <0.001 | 7217 | 950 |
| **Right pars orbitalis** | -0.122 | 0.041 | [-0.202 - -0.041] | -0.877 | 0.003 | 0.014 | <0.001 | 7220 | 950 |
| **Right middle temporal gyrus** | -0.121 | 0.041 | [-0.202 - -0.041] | -0.677 | 0.003 | 0.014 | <0.001 | 7206 | 946 |
| **Right banks superior temporal sulcus** | -0.121 | 0.042 | [-0.204 - -0.039] | -0.864 | 0.004 | 0.016 | <0.001 | 7184 | 889 |
| **Left hemisphere average thickness** | -0.114 | 0.041 | [-0.195 - -0.033] | -0.419 | 0.006 | 0.020 | <0.001 | 7223 | 951 |
| **Right rostral middle frontal gyrus** | -0.104 | 0.086 | [-0.274 - 0.065] | -0.502 | 0.228 | 0.302 | 70.819 | 7215 | 951 |
| **Right pars opercularis** | -0.103 | 0.054 | [-0.208 - 0.003] | -0.515 | 0.056 | 0.119 | 26.869 | 7217 | 951 |
| **Left inferior temporal gyrus** | -0.102 | 0.057 | [-0.214 - 0.009] | -0.571 | 0.072 | 0.137 | 31.669 | 7195 | 938 |
| **Right hemisphere average thickness** | -0.102 | 0.041 | [-0.183 - -0.022] | -0.374 | 0.013 | 0.038 | <0.001 | 7223 | 951 |
| **Right pars triangularis** | -0.099 | 0.070 | [-0.237 - 0.039] | -0.569 | 0.161 | 0.230 | 54.725 | 7210 | 949 |
| **Left lateral orbitofrontal cortex** | -0.095 | 0.041 | [-0.176 - -0.014] | -0.572 | 0.021 | 0.058 | <0.001 | 7203 | 949 |
| **Left superior temporal gyrus** | -0.094 | 0.061 | [-0.214 - 0.027] | -0.572 | 0.128 | 0.199 | 37.584 | 7128 | 900 |
| **Right inferior parietal cortex** | -0.094 | 0.064 | [-0.22 - 0.033] | -0.530 | 0.147 | 0.218 | 46.292 | 7206 | 947 |
| **Left supramarginal gyrus** | -0.089 | 0.042 | [-0.171 - -0.008] | -0.481 | 0.032 | 0.077 | <0.001 | 7176 | 927 |
| **Left banks superior temporal sulcus** | -0.085 | 0.042 | [-0.168 - -0.002] | -0.621 | 0.046 | 0.103 | <0.001 | 7154 | 873 |
| **Right frontal pole** | -0.085 | 0.041 | [-0.165 - -0.004] | -0.882 | 0.040 | 0.093 | <0.001 | 7223 | 951 |
| **Right superior temporal gyrus** | -0.082 | 0.042 | [-0.164 - 0] | -0.491 | 0.050 | 0.110 | <0.001 | 7162 | 907 |
| **Right transverse temporal gyrus** | -0.074 | 0.041 | [-0.155 - 0.007] | -0.597 | 0.072 | 0.137 | <0.001 | 7187 | 949 |
| **Left frontal pole** | -0.067 | 0.041 | [-0.148 - 0.014] | -0.705 | 0.104 | 0.174 | <0.001 | 7221 | 951 |
| **Right caudal middle frontal gyrus** | -0.067 | 0.041 | [-0.148 - 0.014] | -0.352 | 0.104 | 0.174 | <0.001 | 7215 | 951 |
| **Left inferior parietal cortex** | -0.065 | 0.043 | [-0.149 - 0.019] | -0.368 | 0.130 | 0.199 | 3.157 | 7203 | 949 |
| **Left transverse temporal gyrus** | -0.064 | 0.041 | [-0.145 - 0.016] | -0.507 | 0.118 | 0.188 | <0.001 | 7201 | 950 |
| **Right precentral gyrus** | -0.059 | 0.041 | [-0.14 - 0.021] | -0.353 | 0.150 | 0.219 | <0.001 | 7211 | 950 |
| **Left caudal middle frontal gyrus** | -0.058 | 0.054 | [-0.164 - 0.048] | -0.296 | 0.282 | 0.366 | 27.253 | 7213 | 951 |
| **Left precentral gyrus** | -0.052 | 0.041 | [-0.133 - 0.028] | -0.300 | 0.203 | 0.274 | <0.001 | 7205 | 951 |
| **Left lateral occipital cortex** | -0.048 | 0.051 | [-0.149 - 0.052] | -0.278 | 0.346 | 0.432 | 21.096 | 7210 | 949 |
| **Left precuneus** | -0.046 | 0.050 | [-0.145 - 0.052] | -0.221 | 0.357 | 0.438 | 19.165 | 7214 | 949 |
| **Left entorhinal cortex** | -0.043 | 0.055 | [-0.15 - 0.064] | -0.495 | 0.432 | 0.509 | 27.726 | 7175 | 927 |
| **Right entorhinal cortex** | -0.043 | 0.042 | [-0.125 - 0.039] | -0.511 | 0.303 | 0.386 | <0.001 | 7171 | 923 |
| **Left paracentral lobule** | -0.030 | 0.041 | [-0.111 - 0.051] | -0.170 | 0.468 | 0.537 | <0.001 | 7216 | 950 |
| **Right paracentral lobule** | -0.026 | 0.041 | [-0.107 - 0.054] | -0.149 | 0.525 | 0.583 | <0.001 | 7217 | 950 |
| **Right precuneus** | -0.016 | 0.043 | [-0.101 - 0.069] | -0.081 | 0.709 | 0.752 | 4.475 | 7214 | 948 |
| **Left temporal pole** | -0.013 | 0.059 | [-0.129 - 0.103] | -0.131 | 0.827 | 0.851 | 36.349 | 7182 | 940 |
| **Right cuneus** | -0.012 | 0.087 | [-0.182 - 0.158] | -0.072 | 0.892 | 0.905 | 70.711 | 7221 | 946 |
| **Left superior parietal cortex** | -0.002 | 0.068 | [-0.134 - 0.131] | -0.008 | 0.982 | 0.982 | 51.357 | 7211 | 950 |
| **Left lingual gyrus** | 0.010 | 0.041 | [-0.071 - 0.091] | 0.058 | 0.808 | 0.844 | <0.001 | 7205 | 951 |
| **Right lateral occipital cortex** | 0.018 | 0.041 | [-0.063 - 0.099] | 0.104 | 0.665 | 0.716 | <0.001 | 7215 | 949 |
| **Right lingual gyrus** | 0.022 | 0.041 | [-0.059 - 0.103] | 0.127 | 0.595 | 0.651 | <0.001 | 7208 | 949 |
| **Right superior parietal cortex** | 0.032 | 0.041 | [-0.049 - 0.113] | 0.160 | 0.436 | 0.509 | <0.001 | 7211 | 946 |
| **Left postcentral gyrus** | 0.034 | 0.041 | [-0.047 - 0.115] | 0.188 | 0.408 | 0.493 | <0.001 | 7196 | 949 |
| **Right temporal pole** | 0.056 | 0.041 | [-0.025 - 0.137] | 0.615 | 0.177 | 0.243 | <0.001 | 7197 | 939 |
| **Left cuneus** | 0.057 | 0.041 | [-0.024 - 0.137] | 0.349 | 0.170 | 0.238 | <0.001 | 7217 | 947 |
| **Right postcentral gyrus** | 0.066 | 0.041 | [-0.015 - 0.146] | 0.371 | 0.111 | 0.181 | <0.001 | 7209 | 951 |
| **Left pericalcarine cortex** | 0.069 | 0.098 | [-0.123 - 0.262] | 0.487 | 0.480 | 0.542 | 77.600 | 7210 | 946 |
| **Right pericalcarine cortex** | 0.133 | 0.077 | [-0.018 - 0.284] | 0.971 | 0.084 | 0.146 | 62.397 | 7202 | 947 |

**^a^** Included Samples: CLING, Imaging Genetics Dublin, Clinical Depression Dublin, Sexpect, MMDP 3T, MPIP, Muenster Cohort, NESDA, Novosibirsk, SHIP, SHIP-trend, Sydney, Stanford, Rotterdam study.

AD: antidepressant using; MDD: Major Depressive Disorder; CTL: Controls.

**Supplementary Table S15**: Full meta-analytic results for thickness of each structure for MDD patients not taking antidepressants at time of scanning versus Controls comparison controlling for age, sex and scan center. Adjusted Cohen's d is reported.

|  | **Cohen's d ^a^** | **Std. Err.** | **95% CI** | **% Difference** | **P-value** | **FDR P-value** | **I^2^** | **# Controls** | **# Patients** |
| --- | --- | --- | --- | --- | --- | --- | --- | --- | --- |
|  | **(noAD MDD vs CTL)** |  |  |  |  |  |  |  |  |
| **Left medial orbitofrontal cortex** | -0.144 | 0.040 | [-0.222 - -0.066] | -1.116 | 3.06E-04 | 0.021 | <0.001 | 7205 | 909 |
| **Right fusiform gyrus** | -0.091 | 0.040 | [-0.169 - -0.013] | -0.444 | 0.022 | 0.743 | <0.001 | 7240 | 914 |
| **Left fusiform gyrus** | -0.083 | 0.051 | [-0.184 - 0.017] | -0.410 | 0.105 | 0.743 | 25.891 | 7236 | 912 |
| **Right inferior temporal gyrus** | -0.083 | 0.040 | [-0.161 - -0.005] | -0.451 | 0.037 | 0.743 | <0.001 | 7231 | 905 |
| **Right middle temporal gyrus** | -0.080 | 0.040 | [-0.158 - -0.002] | -0.446 | 0.045 | 0.743 | <0.001 | 7232 | 906 |
| **Left insula** | -0.073 | 0.049 | [-0.168 - 0.022] | -0.382 | 0.133 | 0.743 | 19.945 | 7247 | 914 |
| **Left rostral anterior cingulate cortex** | -0.073 | 0.045 | [-0.16 - 0.014] | -0.710 | 0.102 | 0.743 | 10.374 | 7247 | 914 |
| **Right medial orbitofrontal cortex** | -0.071 | 0.090 | [-0.247 - 0.105] | -0.596 | 0.428 | 0.933 | 75.229 | 7223 | 913 |
| **Left middle temporal gyrus** | -0.067 | 0.053 | [-0.171 - 0.038] | -0.397 | 0.210 | 0.743 | 28.146 | 7192 | 883 |
| **Right banks superior temporal sulcus** | -0.065 | 0.049 | [-0.161 - 0.03] | -0.466 | 0.179 | 0.743 | 18.934 | 7207 | 903 |
| **Left superior frontal gyrus** | -0.064 | 0.057 | [-0.177 - 0.048] | -0.324 | 0.264 | 0.804 | 38.899 | 7243 | 914 |
| **Right rostral anterior cingulate cortex** | -0.062 | 0.050 | [-0.16 - 0.036] | -0.632 | 0.212 | 0.743 | 22.904 | 7246 | 915 |
| **Left lateral orbitofrontal cortex** | -0.061 | 0.052 | [-0.162 - 0.041] | -0.366 | 0.242 | 0.804 | 26.952 | 7229 | 914 |
| **Right lingual gyrus** | -0.059 | 0.040 | [-0.137 - 0.019] | -0.341 | 0.139 | 0.743 | <0.001 | 7232 | 910 |
| **Left inferior parietal cortex** | -0.059 | 0.040 | [-0.136 - 0.019] | -0.333 | 0.141 | 0.743 | <0.001 | 7231 | 910 |
| **Right entorhinal cortex** | -0.058 | 0.040 | [-0.136 - 0.02] | -0.693 | 0.143 | 0.743 | <0.001 | 7213 | 908 |
| **Left isthmus cingulate cortex** | -0.056 | 0.040 | [-0.135 - 0.023] | -0.429 | 0.165 | 0.743 | 1.061 | 7246 | 914 |
| **Right transverse temporal gyrus** | -0.053 | 0.040 | [-0.131 - 0.025] | -0.428 | 0.182 | 0.743 | <0.001 | 7213 | 910 |
| **Right lateral orbitofrontal cortex** | -0.051 | 0.040 | [-0.129 - 0.027] | -0.301 | 0.197 | 0.743 | <0.001 | 7235 | 916 |
| **Left banks superior temporal sulcus** | -0.050 | 0.040 | [-0.13 - 0.029] | -0.370 | 0.211 | 0.743 | <0.001 | 7172 | 876 |
| **Right pars orbitalis** | -0.050 | 0.056 | [-0.159 - 0.059] | -0.360 | 0.370 | 0.933 | 35.464 | 7248 | 915 |
| **Left pars orbitalis** | -0.047 | 0.064 | [-0.173 - 0.079] | -0.344 | 0.465 | 0.933 | 50.749 | 7244 | 914 |
| **Left rostral middle frontal gyrus** | -0.045 | 0.056 | [-0.155 - 0.066] | -0.216 | 0.428 | 0.933 | 36.811 | 7244 | 914 |
| **Right posterior cingulate cortex** | -0.045 | 0.040 | [-0.122 - 0.033] | -0.298 | 0.261 | 0.804 | <0.001 | 7245 | 914 |
| **Right insula** | -0.044 | 0.055 | [-0.152 - 0.063] | -0.242 | 0.417 | 0.933 | 33.627 | 7247 | 914 |
| **Right frontal pole** | -0.041 | 0.040 | [-0.119 - 0.037] | -0.425 | 0.305 | 0.844 | <0.001 | 7248 | 913 |
| **Right caudal anterior cingulate cortex** | -0.040 | 0.040 | [-0.118 - 0.037] | -0.449 | 0.309 | 0.844 | <0.001 | 7247 | 914 |
| **Right superior frontal gyrus** | -0.040 | 0.090 | [-0.217 - 0.136] | -0.195 | 0.656 | 0.933 | 75.568 | 7240 | 914 |
| **Left posterior cingulate cortex** | -0.040 | 0.040 | [-0.118 - 0.038] | -0.250 | 0.313 | 0.844 | <0.001 | 7245 | 915 |
| **Left pars opercularis** | -0.039 | 0.061 | [-0.158 - 0.08] | -0.186 | 0.522 | 0.933 | 45.216 | 7246 | 913 |
| **Right isthmus cingulate cortex** | -0.036 | 0.064 | [-0.161 - 0.09] | -0.281 | 0.579 | 0.933 | 50.273 | 7244 | 913 |
| **Left lingual gyrus** | -0.029 | 0.054 | [-0.136 - 0.077] | -0.171 | 0.587 | 0.933 | 32.560 | 7231 | 909 |
| **Right lateral occipital cortex** | -0.029 | 0.049 | [-0.125 - 0.066] | -0.172 | 0.546 | 0.933 | 19.682 | 7241 | 914 |
| **Left entorhinal cortex** | -0.029 | 0.040 | [-0.107 - 0.049] | -0.335 | 0.465 | 0.933 | <0.001 | 7216 | 908 |
| **Left inferior temporal gyrus** | -0.029 | 0.067 | [-0.16 - 0.102] | -0.161 | 0.666 | 0.933 | 53.593 | 7227 | 903 |
| **Left hemisphere average thickness** | -0.027 | 0.040 | [-0.104 - 0.051] | -0.098 | 0.500 | 0.933 | <0.001 | 7249 | 916 |
| **Left transverse temporal gyrus** | -0.025 | 0.040 | [-0.103 - 0.052] | -0.200 | 0.522 | 0.933 | <0.001 | 7226 | 910 |
| **Right parahippocampal gyrus** | -0.025 | 0.040 | [-0.103 - 0.052] | -0.276 | 0.523 | 0.933 | <0.001 | 7246 | 915 |
| **Right precentral gyrus** | -0.025 | 0.066 | [-0.154 - 0.104] | -0.147 | 0.708 | 0.961 | 52.652 | 7234 | 909 |
| **Right supramarginal gyrus** | -0.019 | 0.072 | [-0.16 - 0.123] | -0.097 | 0.793 | 0.968 | 60.488 | 7226 | 909 |
| **Left precentral gyrus** | -0.018 | 0.064 | [-0.143 - 0.107] | -0.104 | 0.777 | 0.968 | 49.859 | 7230 | 909 |
| **Right hemisphere average thickness** | -0.018 | 0.040 | [-0.096 - 0.06] | -0.065 | 0.653 | 0.933 | <0.001 | 7249 | 916 |
| **Right temporal pole** | -0.015 | 0.049 | [-0.11 - 0.08] | -0.163 | 0.760 | 0.968 | 18.841 | 7222 | 900 |
| **Right inferior parietal cortex** | -0.015 | 0.040 | [-0.092 - 0.063] | -0.082 | 0.714 | 0.961 | <0.001 | 7234 | 915 |
| **Left frontal pole** | -0.013 | 0.083 | [-0.177 - 0.15] | -0.138 | 0.875 | 0.973 | 71.121 | 7247 | 913 |
| **Right rostral middle frontal gyrus** | -0.013 | 0.082 | [-0.174 - 0.148] | -0.062 | 0.876 | 0.973 | 70.110 | 7241 | 913 |
| **Left parahippocampal gyrus** | -0.012 | 0.046 | [-0.102 - 0.078] | -0.153 | 0.789 | 0.968 | 13.481 | 7241 | 911 |
| **Left temporal pole** | -0.010 | 0.040 | [-0.089 - 0.068] | -0.106 | 0.793 | 0.968 | <0.001 | 7206 | 900 |
| **Left superior parietal cortex** | -0.009 | 0.040 | [-0.087 - 0.068] | -0.046 | 0.813 | 0.968 | <0.001 | 7236 | 911 |
| **Right paracentral lobule** | -0.007 | 0.040 | [-0.085 - 0.071] | -0.040 | 0.858 | 0.973 | <0.001 | 7243 | 916 |
| **Left supramarginal gyrus** | -0.004 | 0.040 | [-0.082 - 0.074] | -0.021 | 0.924 | 0.996 | <0.001 | 7207 | 903 |
| **Right pericalcarine cortex** | -0.003 | 0.040 | [-0.081 - 0.075] | -0.022 | 0.940 | 0.996 | <0.001 | 7226 | 914 |
| **Right postcentral gyrus** | -0.001 | 0.040 | [-0.079 - 0.077] | -0.007 | 0.973 | 0.996 | <0.001 | 7233 | 911 |
| **Left pars triangularis** | 0.000 | 0.056 | [-0.11 - 0.11] | -0.002 | 0.996 | 0.996 | 36.241 | 7243 | 913 |
| **Right superior temporal gyrus** | 2.48E-04 | 0.047 | [-0.092 - 0.092] | 0.001 | 0.996 | 0.996 | 13.434 | 7182 | 884 |
| **Left caudal middle frontal gyrus** | 9.42E-04 | 0.077 | [-0.15 - 0.152] | 0.005 | 0.990 | 0.996 | 65.852 | 7238 | 913 |
| **Left precuneus** | 0.003 | 0.040 | [-0.075 - 0.081] | 0.016 | 0.934 | 0.996 | <0.001 | 7240 | 909 |
| **Left paracentral lobule** | 0.009 | 0.040 | [-0.069 - 0.086] | 0.048 | 0.830 | 0.968 | <0.001 | 7241 | 914 |
| **Left cuneus** | 0.013 | 0.060 | [-0.104 - 0.131] | 0.081 | 0.827 | 0.968 | 43.565 | 7247 | 915 |
| **Right superior parietal cortex** | 0.018 | 0.040 | [-0.06 - 0.096] | 0.089 | 0.652 | 0.933 | <0.001 | 7236 | 914 |
| **Right precuneus** | 0.018 | 0.040 | [-0.06 - 0.096] | 0.090 | 0.650 | 0.933 | <0.001 | 7237 | 911 |
| **Left lateral occipital cortex** | 0.022 | 0.040 | [-0.056 - 0.1] | 0.127 | 0.578 | 0.933 | <0.001 | 7236 | 914 |
| **Left caudal anterior cingulate cortex** | 0.025 | 0.046 | [-0.066 - 0.115] | 0.279 | 0.596 | 0.933 | 14.372 | 7242 | 915 |
| **Left postcentral gyrus** | 0.027 | 0.040 | [-0.051 - 0.105] | 0.149 | 0.498 | 0.933 | <0.001 | 7222 | 910 |
| **Right pars opercularis** | 0.028 | 0.049 | [-0.068 - 0.124] | 0.141 | 0.565 | 0.933 | 20.499 | 7244 | 910 |
| **Right pars triangularis** | 0.029 | 0.061 | [-0.09 - 0.148] | 0.168 | 0.632 | 0.933 | 45.083 | 7240 | 913 |
| **Right caudal middle frontal gyrus** | 0.050 | 0.068 | [-0.084 - 0.184] | 0.264 | 0.464 | 0.933 | 56.463 | 7241 | 915 |
| **Right cuneus** | 0.060 | 0.040 | [-0.018 - 0.138] | 0.369 | 0.130 | 0.743 | <0.001 | 7246 | 915 |
| **Left pericalcarine cortex** | 0.064 | 0.040 | [-0.013 - 0.142] | 0.453 | 0.105 | 0.743 | <0.001 | 7236 | 913 |
| **Left superior temporal gyrus** | 0.065 | 0.040 | [-0.014 - 0.144] | 0.396 | 0.109 | 0.743 | <0.001 | 7153 | 874 |

**^a^** Included Samples: CODE, Imaging Genetics Dublin, Bipolar Family Study, Houston, MMDP 3T, Melbourne, MPIP, Muenster Cohort, NESDA, DepOx, QTIM, SHIP, SHIP-trend, Sydney, Stanford, Rotterdam study.

noAD: antidepressant free; MDD: Major Depressive Disorder; CTL: Controls.

**Supplementary Table S16**: Full meta-analytic results for thickness of each structure for MDD patients taking antidepressants MDD patients not taking antidepressants at time of scanning comparison controlling for age, sex and scan center. Adjusted Cohen's d is reported.

|  | **Cohen's d ^a^** | **Std. Err.** | **95% CI** | **% Difference** | **P-value** | **FDR P-value** | **I^2^** | **# AD MDD** | **# noAD MDD** |
| --- | --- | --- | --- | --- | --- | --- | --- | --- | --- |
|  | **(AD MDD vs noAD MDD)** |  |  |  |  |  |  |  |  |
| **Right insula** | -0.177 | 0.066 | [-0.306 - -0.048] | -0.963 | 0.007 | 0.402 | <0.001 | 853 | 673 |
| **Right rostral anterior cingulate cortex** | -0.168 | 0.095 | [-0.355 - 0.019] | -1.703 | 0.078 | 0.979 | 47.128 | 853 | 673 |
| **Left rostral anterior cingulate cortex** | -0.166 | 0.066 | [-0.295 - -0.037] | -1.619 | 0.011 | 0.402 | <0.001 | 853 | 671 |
| **Left superior temporal gyrus** | -0.147 | 0.121 | [-0.385 - 0.09] | -0.900 | 0.224 | 0.979 | 64.539 | 812 | 648 |
| **Left parahippocampal gyrus** | -0.136 | 0.072 | [-0.278 - 0.006] | -1.696 | 0.060 | 0.979 | 13.666 | 853 | 671 |
| **Left pars triangularis** | -0.134 | 0.083 | [-0.296 - 0.028] | -0.815 | 0.106 | 0.979 | 31.426 | 853 | 671 |
| **Right medial orbitofrontal cortex** | -0.131 | 0.078 | [-0.284 - 0.022] | -1.099 | 0.093 | 0.979 | 23.408 | 851 | 671 |
| **Left caudal anterior cingulate cortex** | -0.130 | 0.088 | [-0.302 - 0.042] | -1.478 | 0.137 | 0.979 | 38.182 | 853 | 672 |
| **Right lateral orbitofrontal cortex** | -0.113 | 0.066 | [-0.242 - 0.016] | -0.664 | 0.086 | 0.979 | <0.001 | 853 | 673 |
| **Left pars opercularis** | -0.104 | 0.149 | [-0.396 - 0.188] | -0.496 | 0.485 | 0.979 | 78.453 | 853 | 671 |
| **Left fusiform gyrus** | -0.101 | 0.066 | [-0.23 - 0.028] | -0.497 | 0.125 | 0.979 | <0.001 | 852 | 669 |
| **Right caudal anterior cingulate cortex** | -0.101 | 0.098 | [-0.294 - 0.092] | -1.120 | 0.307 | 0.979 | 50.530 | 853 | 673 |
| **Left insula** | -0.099 | 0.099 | [-0.294 - 0.096] | -0.515 | 0.322 | 0.979 | 51.484 | 853 | 673 |
| **Right parahippocampal gyrus** | -0.090 | 0.105 | [-0.295 - 0.116] | -0.977 | 0.393 | 0.979 | 56.190 | 851 | 673 |
| **Right pars triangularis** | -0.086 | 0.082 | [-0.246 - 0.074] | -0.496 | 0.293 | 0.979 | 29.847 | 852 | 671 |
| **Left inferior temporal gyrus** | -0.077 | 0.106 | [-0.285 - 0.131] | -0.431 | 0.467 | 0.979 | 55.913 | 845 | 663 |
| **Right pars orbitalis** | -0.075 | 0.072 | [-0.216 - 0.065] | -0.544 | 0.292 | 0.979 | 12.081 | 853 | 673 |
| **Left cuneus** | -0.073 | 0.111 | [-0.291 - 0.145] | -0.450 | 0.511 | 0.979 | 60.972 | 853 | 672 |
| **Right cuneus** | -0.072 | 0.084 | [-0.236 - 0.093] | -0.439 | 0.393 | 0.979 | 32.428 | 851 | 673 |
| **Right precuneus** | -0.071 | 0.073 | [-0.214 - 0.072] | -0.355 | 0.328 | 0.979 | 14.537 | 852 | 670 |
| **Left pericalcarine cortex** | -0.067 | 0.071 | [-0.206 - 0.072] | -0.471 | 0.347 | 0.979 | 11.087 | 852 | 670 |
| **Left superior frontal gyrus** | -0.066 | 0.066 | [-0.195 - 0.063] | -0.333 | 0.318 | 0.979 | 0.455 | 853 | 671 |
| **Right superior frontal gyrus** | -0.062 | 0.113 | [-0.283 - 0.159] | -0.302 | 0.581 | 0.979 | 62.137 | 853 | 672 |
| **Right posterior cingulate cortex** | -0.061 | 0.066 | [-0.189 - 0.068] | -0.406 | 0.356 | 0.979 | <0.001 | 853 | 673 |
| **Left transverse temporal gyrus** | -0.058 | 0.074 | [-0.203 - 0.087] | -0.455 | 0.434 | 0.979 | 16.362 | 852 | 668 |
| **Left hemisphere average thickness** | -0.058 | 0.074 | [-0.203 - 0.088] | -0.212 | 0.437 | 0.979 | 17.107 | 853 | 673 |
| **Left pars orbitalis** | -0.054 | 0.083 | [-0.217 - 0.108] | -0.398 | 0.512 | 0.979 | 31.782 | 853 | 673 |
| **Right isthmus cingulate cortex** | -0.052 | 0.107 | [-0.261 - 0.158] | -0.410 | 0.628 | 0.979 | 57.865 | 852 | 672 |
| **Left posterior cingulate cortex** | -0.047 | 0.066 | [-0.176 - 0.081] | -0.297 | 0.470 | 0.979 | <0.001 | 853 | 673 |
| **Right fusiform gyrus** | -0.046 | 0.066 | [-0.174 - 0.083] | -0.222 | 0.488 | 0.979 | <0.001 | 852 | 671 |
| **Right hemisphere average thickness** | -0.045 | 0.081 | [-0.205 - 0.114] | -0.166 | 0.577 | 0.979 | 29.365 | 853 | 673 |
| **Left lateral occipital cortex** | -0.033 | 0.079 | [-0.189 - 0.123] | -0.190 | 0.678 | 0.979 | 25.803 | 852 | 671 |
| **Left supramarginal gyrus** | -0.032 | 0.072 | [-0.174 - 0.11] | -0.174 | 0.656 | 0.979 | 13.480 | 840 | 666 |
| **Left temporal pole** | -0.031 | 0.146 | [-0.317 - 0.254] | -0.317 | 0.830 | 0.979 | 76.422 | 842 | 660 |
| **Right superior temporal gyrus** | -0.031 | 0.134 | [-0.294 - 0.231] | -0.187 | 0.816 | 0.979 | 71.086 | 813 | 648 |
| **Left lateral orbitofrontal cortex** | -0.031 | 0.075 | [-0.177 - 0.116] | -0.185 | 0.682 | 0.979 | 17.523 | 851 | 671 |
| **Left middle temporal gyrus** | -0.029 | 0.114 | [-0.253 - 0.194] | -0.174 | 0.797 | 0.979 | 61.147 | 819 | 652 |
| **Right pars opercularis** | -0.029 | 0.066 | [-0.158 - 0.099] | -0.147 | 0.656 | 0.979 | <0.001 | 853 | 669 |
| **Right inferior temporal gyrus** | -0.027 | 0.066 | [-0.157 - 0.102] | -0.148 | 0.679 | 0.979 | <0.001 | 849 | 665 |
| **Right supramarginal gyrus** | -0.019 | 0.120 | [-0.255 - 0.216] | -0.099 | 0.873 | 0.979 | 66.219 | 834 | 668 |
| **Right pericalcarine cortex** | -0.017 | 0.090 | [-0.192 - 0.159] | -0.121 | 0.853 | 0.979 | 40.802 | 853 | 671 |
| **Right transverse temporal gyrus** | -0.016 | 0.066 | [-0.145 - 0.113] | -0.129 | 0.807 | 0.979 | <0.001 | 851 | 668 |
| **Left postcentral gyrus** | -0.011 | 0.078 | [-0.165 - 0.142] | -0.063 | 0.885 | 0.979 | 24.592 | 853 | 668 |
| **Right entorhinal cortex** | -0.009 | 0.066 | [-0.138 - 0.121] | -0.102 | 0.896 | 0.979 | <0.001 | 844 | 667 |
| **Right caudal middle frontal gyrus** | -0.008 | 0.111 | [-0.225 - 0.21] | -0.041 | 0.944 | 0.979 | 60.912 | 853 | 672 |
| **Right inferior parietal cortex** | -0.007 | 0.067 | [-0.138 - 0.123] | -0.042 | 0.911 | 0.979 | 1.978 | 849 | 672 |
| **Left medial orbitofrontal cortex** | -0.007 | 0.066 | [-0.137 - 0.123] | -0.054 | 0.916 | 0.979 | <0.001 | 849 | 667 |
| **Left paracentral lobule** | 9.70E-04 | 0.066 | [-0.128 - 0.13] | 0.006 | 0.988 | 0.988 | <0.001 | 852 | 672 |
| **Left precuneus** | 0.001 | 0.070 | [-0.137 - 0.139] | 0.005 | 0.988 | 0.988 | 9.251 | 852 | 669 |
| **Left inferior parietal cortex** | 0.005 | 0.076 | [-0.144 - 0.153] | 0.027 | 0.951 | 0.979 | 19.680 | 851 | 670 |
| **Right frontal pole** | 0.006 | 0.066 | [-0.123 - 0.135] | 0.064 | 0.925 | 0.979 | <0.001 | 853 | 670 |
| **Right banks superior temporal sulcus** | 0.010 | 0.074 | [-0.134 - 0.154] | 0.070 | 0.894 | 0.979 | 14.438 | 795 | 666 |
| **Right precentral gyrus** | 0.012 | 0.066 | [-0.117 - 0.141] | 0.072 | 0.853 | 0.979 | <0.001 | 853 | 670 |
| **Left banks superior temporal sulcus** | 0.013 | 0.068 | [-0.12 - 0.146] | 0.094 | 0.850 | 0.979 | <0.001 | 785 | 650 |
| **Left isthmus cingulate cortex** | 0.017 | 0.066 | [-0.112 - 0.146] | 0.130 | 0.796 | 0.979 | <0.001 | 852 | 673 |
| **Right middle temporal gyrus** | 0.019 | 0.066 | [-0.11 - 0.149] | 0.109 | 0.768 | 0.979 | <0.001 | 849 | 665 |
| **Left caudal middle frontal gyrus** | 0.020 | 0.069 | [-0.116 - 0.156] | 0.103 | 0.772 | 0.979 | 8.008 | 853 | 670 |
| **Left precentral gyrus** | 0.021 | 0.080 | [-0.135 - 0.177] | 0.118 | 0.797 | 0.979 | 26.542 | 853 | 670 |
| **Right temporal pole** | 0.021 | 0.119 | [-0.213 - 0.254] | 0.227 | 0.862 | 0.979 | 64.350 | 841 | 661 |
| **Left rostral middle frontal gyrus** | 0.022 | 0.066 | [-0.107 - 0.151] | 0.108 | 0.735 | 0.979 | <0.001 | 852 | 671 |
| **Left lingual gyrus** | 0.026 | 0.104 | [-0.177 - 0.23] | 0.153 | 0.799 | 0.979 | 55.649 | 853 | 667 |
| **Right postcentral gyrus** | 0.028 | 0.066 | [-0.1 - 0.157] | 0.161 | 0.666 | 0.979 | <0.001 | 853 | 669 |
| **Right lateral occipital cortex** | 0.031 | 0.066 | [-0.098 - 0.16] | 0.182 | 0.637 | 0.979 | <0.001 | 851 | 671 |
| **Left entorhinal cortex** | 0.034 | 0.066 | [-0.096 - 0.163] | 0.387 | 0.611 | 0.979 | <0.001 | 848 | 668 |
| **Right rostral middle frontal gyrus** | 0.038 | 0.093 | [-0.144 - 0.22] | 0.184 | 0.680 | 0.979 | 44.460 | 853 | 670 |
| **Right lingual gyrus** | 0.041 | 0.066 | [-0.088 - 0.17] | 0.237 | 0.534 | 0.979 | <0.001 | 853 | 667 |
| **Right paracentral lobule** | 0.043 | 0.066 | [-0.085 - 0.172] | 0.246 | 0.509 | 0.979 | <0.001 | 852 | 673 |
| **Left superior parietal cortex** | 0.044 | 0.087 | [-0.126 - 0.214] | 0.217 | 0.609 | 0.979 | 36.967 | 853 | 670 |
| **Right superior parietal cortex** | 0.044 | 0.066 | [-0.084 - 0.173] | 0.222 | 0.499 | 0.979 | <0.001 | 852 | 673 |
| **Left frontal pole** | 0.114 | 0.129 | [-0.138 - 0.367] | 1.205 | 0.375 | 0.979 | 71.096 | 853 | 671 |

**^a^** Included Samples: Imaging Genetics Dublin, MMDP 3T, MPIP, Muenster Cohort, NESDA, SHIP, SHIP-trend, Sydney, Stanford, Rotterdam study.

AD: antidepressant using; noAD: antidepressant free; MDD: Major Depressive Disorder.

**Supplementary Table S17**: Full meta-analytic results for thickness of each structure associated with severity of symptoms at study inclusion measured by the HDRS-17 controlling for age, sex and scan center. Adjusted Cohen's d is reported.

|  | **Pearsons' r ^a^** | **Std. Err.** | **95% CI** | **% Difference** | **P-value** | **FDR P-value** | **I^2^** | **# Patients** |
| --- | --- | --- | --- | --- | --- | --- | --- | --- |
|  | **(HDRS-17)** |  |  |  |  |  |  |  |
| **Right pars triangularis** | -0.102 | 0.081 | [-0.261 - 0.058] | -1.178 | 0.211 | 0.884 | 79.261 | 775 |
| **Right pars opercularis** | -0.094 | 0.087 | [-0.265 - 0.077] | -0.946 | 0.281 | 0.884 | 81.492 | 776 |
| **Right precentral gyrus** | -0.092 | 0.091 | [-0.27 - 0.086] | -1.097 | 0.312 | 0.884 | 83.492 | 775 |
| **Right insula** | -0.087 | 0.054 | [-0.192 - 0.018] | -0.954 | 0.103 | 0.884 | 44.645 | 771 |
| **Left entorhinal cortex** | -0.087 | 0.044 | [-0.172 - -0.001] | -2.001 | 0.047 | 0.884 | 21.607 | 757 |
| **Right banks superior temporal sulcus** | -0.084 | 0.102 | [-0.284 - 0.117] | -1.197 | 0.412 | 0.884 | 87.625 | 741 |
| **Right supramarginal gyrus** | -0.081 | 0.066 | [-0.21 - 0.049] | -0.831 | 0.224 | 0.884 | 64.645 | 764 |
| **Left lateral orbitofrontal cortex** | -0.079 | 0.077 | [-0.23 - 0.071] | -0.961 | 0.301 | 0.884 | 76.238 | 776 |
| **Left lateral occipital cortex** | -0.078 | 0.064 | [-0.204 - 0.047] | -0.906 | 0.220 | 0.884 | 62.486 | 775 |
| **Right postcentral gyrus** | -0.078 | 0.074 | [-0.223 - 0.067] | -0.889 | 0.291 | 0.884 | 72.955 | 776 |
| **Left pars triangularis** | -0.076 | 0.070 | [-0.214 - 0.061] | -0.933 | 0.275 | 0.884 | 69.992 | 774 |
| **Left superior temporal gyrus** | -0.075 | 0.091 | [-0.253 - 0.102] | -0.923 | 0.405 | 0.884 | 81.813 | 741 |
| **Right parahippocampal gyrus** | -0.070 | 0.067 | [-0.202 - 0.062] | -1.529 | 0.298 | 0.884 | 65.955 | 773 |
| **Right hemisphere average thickness** | -0.070 | 0.088 | [-0.242 - 0.102] | -0.510 | 0.428 | 0.884 | 81.672 | 776 |
| **Left middle temporal gyrus** | -0.062 | 0.085 | [-0.229 - 0.104] | -0.741 | 0.464 | 0.884 | 79.379 | 738 |
| **Right temporal pole** | -0.061 | 0.064 | [-0.187 - 0.065] | -1.343 | 0.344 | 0.884 | 62.721 | 776 |
| **Left hemisphere average thickness** | -0.060 | 0.075 | [-0.207 - 0.087] | -0.441 | 0.425 | 0.884 | 73.645 | 776 |
| **Right frontal pole** | -0.059 | 0.059 | [-0.174 - 0.057] | -1.224 | 0.321 | 0.884 | 55.465 | 776 |
| **Right superior parietal cortex** | -0.057 | 0.053 | [-0.161 - 0.046] | -0.574 | 0.278 | 0.884 | 43.539 | 772 |
| **Left pars orbitalis** | -0.057 | 0.083 | [-0.219 - 0.105] | -0.839 | 0.488 | 0.884 | 79.444 | 776 |
| **Right isthmus cingulate cortex** | -0.055 | 0.071 | [-0.194 - 0.084] | -0.876 | 0.435 | 0.884 | 69.943 | 775 |
| **Right fusiform gyrus** | -0.054 | 0.090 | [-0.231 - 0.122] | -0.532 | 0.545 | 0.884 | 83.084 | 776 |
| **Right inferior parietal cortex** | -0.054 | 0.063 | [-0.178 - 0.07] | -0.613 | 0.392 | 0.884 | 60.777 | 775 |
| **Left parahippocampal gyrus** | -0.051 | 0.036 | [-0.121 - 0.019] | -1.274 | 0.153 | 0.884 | <0.001 | 775 |
| **Left precentral gyrus** | -0.050 | 0.064 | [-0.175 - 0.075] | -0.574 | 0.432 | 0.884 | 61.100 | 776 |
| **Left caudal middle frontal gyrus** | -0.050 | 0.036 | [-0.12 - 0.021] | -0.508 | 0.166 | 0.884 | <0.001 | 776 |
| **Left fusiform gyrus** | -0.049 | 0.077 | [-0.2 - 0.103] | -0.478 | 0.531 | 0.884 | 76.318 | 776 |
| **Left precuneus** | -0.049 | 0.093 | [-0.23 - 0.133] | -0.464 | 0.600 | 0.884 | 83.654 | 775 |
| **Left rostral middle frontal gyrus** | -0.048 | 0.071 | [-0.186 - 0.091] | -0.462 | 0.501 | 0.884 | 70.119 | 776 |
| **Right lingual gyrus** | -0.047 | 0.036 | [-0.117 - 0.023] | -0.548 | 0.185 | 0.884 | <0.001 | 774 |
| **Left lingual gyrus** | -0.046 | 0.052 | [-0.148 - 0.055] | -0.536 | 0.372 | 0.884 | 42.171 | 776 |
| **Left rostral anterior cingulate cortex** | -0.046 | 0.059 | [-0.162 - 0.07] | -0.900 | 0.436 | 0.884 | 54.645 | 772 |
| **Right paracentral lobule** | -0.044 | 0.071 | [-0.184 - 0.096] | -0.503 | 0.536 | 0.884 | 69.992 | 776 |
| **Right caudal middle frontal gyrus** | -0.042 | 0.046 | [-0.132 - 0.048] | -0.438 | 0.365 | 0.884 | 27.770 | 776 |
| **Right middle temporal gyrus** | -0.036 | 0.071 | [-0.176 - 0.103] | -0.406 | 0.610 | 0.884 | 70.956 | 772 |
| **Left pars opercularis** | -0.036 | 0.069 | [-0.17 - 0.099] | -0.343 | 0.600 | 0.884 | 67.865 | 775 |
| **Left frontal pole** | -0.036 | 0.036 | [-0.106 - 0.034] | -0.755 | 0.318 | 0.884 | <0.001 | 776 |
| **Left superior frontal gyrus** | -0.035 | 0.056 | [-0.145 - 0.076] | -0.352 | 0.537 | 0.884 | 50.174 | 775 |
| **Left isthmus cingulate cortex** | -0.033 | 0.070 | [-0.17 - 0.104] | -0.506 | 0.639 | 0.884 | 69.241 | 774 |
| **Right precuneus** | -0.030 | 0.058 | [-0.143 - 0.083] | -0.298 | 0.604 | 0.884 | 52.065 | 774 |
| **Left inferior parietal cortex** | -0.029 | 0.059 | [-0.145 - 0.086] | -0.333 | 0.619 | 0.884 | 54.195 | 774 |
| **Right lateral occipital cortex** | -0.027 | 0.064 | [-0.152 - 0.099] | -0.311 | 0.678 | 0.884 | 62.479 | 776 |
| **Left inferior temporal gyrus** | -0.024 | 0.060 | [-0.141 - 0.092] | -0.272 | 0.682 | 0.884 | 55.609 | 773 |
| **Left postcentral gyrus** | -0.024 | 0.062 | [-0.146 - 0.097] | -0.267 | 0.697 | 0.887 | 58.777 | 774 |
| **Left superior parietal cortex** | -0.022 | 0.049 | [-0.118 - 0.075] | -0.212 | 0.660 | 0.884 | 35.303 | 775 |
| **Left medial orbitofrontal cortex** | -0.019 | 0.068 | [-0.152 - 0.114] | -0.299 | 0.777 | 0.937 | 66.219 | 773 |
| **Right inferior temporal gyrus** | -0.019 | 0.065 | [-0.147 - 0.109] | -0.204 | 0.773 | 0.937 | 63.412 | 776 |
| **Left banks superior temporal sulcus** | -0.018 | 0.067 | [-0.149 - 0.114] | -0.259 | 0.793 | 0.940 | 62.402 | 712 |
| **Right superior frontal gyrus** | -0.012 | 0.066 | [-0.141 - 0.117] | -0.119 | 0.852 | 0.961 | 63.832 | 776 |
| **Left cuneus** | -0.012 | 0.075 | [-0.158 - 0.134] | -0.147 | 0.873 | 0.961 | 72.857 | 772 |
| **Right posterior cingulate cortex** | -0.008 | 0.036 | [-0.078 - 0.063] | -0.104 | 0.829 | 0.961 | <0.001 | 776 |
| **Left posterior cingulate cortex** | -0.007 | 0.071 | [-0.147 - 0.132] | -0.093 | 0.917 | 0.961 | 70.651 | 776 |
| **Left paracentral lobule** | -0.004 | 0.049 | [-0.1 - 0.092] | -0.046 | 0.935 | 0.961 | 34.140 | 776 |
| **Right rostral middle frontal gyrus** | -0.004 | 0.053 | [-0.107 - 0.1] | -0.034 | 0.947 | 0.961 | 43.969 | 776 |
| **Left pericalcarine cortex** | -0.002 | 0.036 | [-0.072 - 0.068] | -0.030 | 0.952 | 0.961 | <0.001 | 772 |
| **Right entorhinal cortex** | -0.002 | 0.039 | [-0.079 - 0.075] | -0.046 | 0.961 | 0.961 | 8.646 | 756 |
| **Left supramarginal gyrus** | 0.002 | 0.036 | [-0.069 - 0.073] | 0.020 | 0.958 | 0.961 | 0.870 | 760 |
| **Right pars orbitalis** | 0.005 | 0.040 | [-0.074 - 0.083] | 0.066 | 0.909 | 0.961 | 12.364 | 775 |
| **Right superior temporal gyrus** | 0.006 | 0.073 | [-0.137 - 0.15] | 0.076 | 0.931 | 0.961 | 70.840 | 760 |
| **Left transverse temporal gyrus** | 0.021 | 0.036 | [-0.049 - 0.091] | 0.331 | 0.557 | 0.884 | <0.001 | 776 |
| **Right medial orbitofrontal cortex** | 0.024 | 0.036 | [-0.046 - 0.094] | 0.403 | 0.502 | 0.884 | <0.001 | 775 |
| **Left temporal pole** | 0.026 | 0.061 | [-0.094 - 0.145] | 0.516 | 0.674 | 0.884 | 57.189 | 776 |
| **Right cuneus** | 0.030 | 0.082 | [-0.13 - 0.19] | 0.370 | 0.712 | 0.890 | 77.857 | 773 |
| **Right caudal anterior cingulate cortex** | 0.036 | 0.055 | [-0.071 - 0.143] | 0.802 | 0.509 | 0.884 | 45.992 | 774 |
| **Left insula** | 0.039 | 0.080 | [-0.118 - 0.197] | 0.411 | 0.624 | 0.884 | 76.896 | 774 |
| **Right lateral orbitofrontal cortex** | 0.040 | 0.050 | [-0.059 - 0.139] | 0.472 | 0.426 | 0.884 | 38.411 | 776 |
| **Right pericalcarine cortex** | 0.047 | 0.068 | [-0.087 - 0.18] | 0.680 | 0.495 | 0.884 | 66.571 | 772 |
| **Right transverse temporal gyrus** | 0.049 | 0.036 | [-0.021 - 0.119] | 0.791 | 0.170 | 0.884 | <0.001 | 776 |
| **Left caudal anterior cingulate cortex** | 0.087 | 0.106 | [-0.122 - 0.295] | 1.975 | 0.415 | 0.884 | 88.971 | 775 |
| **Right rostral anterior cingulate cortex** | 0.127 | 0.082 | [-0.034 - 0.287] | 2.595 | 0.121 | 0.884 | 79.544 | 774 |

**^a^** Included Samples: CLING, Imaging Genetics Dublin, Clinical Depression Dublin, Bipolar Family Study, Houston, Sexpect, MMDP 3T, MPIP, Muenster Cohort, DepOx, Sydney.

HDRS-17: Hamilton Depression Rating Scale with 17 items.

**Supplementary Table S18**: Full meta-analytic results for thickness of each structure associated with severity of symptoms at study inclusion measured by the BDI-II controlling for age, sex and scan center. Adjusted Cohen's d is reported.

|  | **Pearson's r ^a^** | **Std. Err.** | **95% CI** | **% Difference** | **P-value** | **FDR P-value** | **I^2^** | **# Patients** |
| --- | --- | --- | --- | --- | --- | --- | --- | --- |
|  | **(BDI-II)** |  |  |  |  |  |  |  |
| **Left caudal middle frontal gyrus** | -0.111 | 0.052 | [-0.214 - -0.008] | -1.136 | 0.035 | 0.513 | 50.294 | 940 |
| **Left postcentral gyrus** | -0.111 | 0.032 | [-0.174 - -0.048] | -1.229 | 5.76E-04 | 0.040 | <0.001 | 937 |
| **Right pars triangularis** | -0.089 | 0.058 | [-0.203 - 0.025] | -1.028 | 0.127 | 0.513 | 58.577 | 939 |
| **Left insula** | -0.075 | 0.032 | [-0.139 - -0.012] | -0.788 | 0.020 | 0.513 | <0.001 | 942 |
| **Right caudal middle frontal gyrus** | -0.062 | 0.032 | [-0.126 - 0.002] | -0.654 | 0.056 | 0.513 | <0.001 | 942 |
| **Left pars opercularis** | -0.062 | 0.033 | [-0.125 - 0.002] | -0.589 | 0.058 | 0.513 | <0.001 | 940 |
| **Right banks superior temporal sulcus** | -0.061 | 0.033 | [-0.126 - 0.003] | -0.877 | 0.062 | 0.513 | <0.001 | 918 |
| **Left rostral middle frontal gyrus** | -0.058 | 0.033 | [-0.122 - 0.005] | -0.567 | 0.072 | 0.513 | <0.001 | 940 |
| **Left precuneus** | -0.058 | 0.032 | [-0.121 - 0.006] | -0.551 | 0.076 | 0.513 | <0.001 | 938 |
| **Right postcentral gyrus** | -0.057 | 0.033 | [-0.121 - 0.006] | -0.651 | 0.077 | 0.513 | <0.001 | 939 |
| **Right superior parietal cortex** | -0.057 | 0.043 | [-0.142 - 0.027] | -0.572 | 0.185 | 0.540 | 30.073 | 939 |
| **Right precuneus** | -0.056 | 0.032 | [-0.12 - 0.007] | -0.562 | 0.082 | 0.513 | <0.001 | 937 |
| **Left hemisphere average thickness** | -0.056 | 0.032 | [-0.12 - 0.007] | -0.414 | 0.083 | 0.513 | <0.001 | 943 |
| **Left inferior parietal cortex** | -0.054 | 0.032 | [-0.117 - 0.01] | -0.611 | 0.096 | 0.513 | <0.001 | 941 |
| **Left paracentral lobule** | -0.053 | 0.032 | [-0.116 - 0.011] | -0.601 | 0.104 | 0.513 | <0.001 | 941 |
| **Right superior frontal gyrus** | -0.052 | 0.032 | [-0.116 - 0.012] | -0.506 | 0.109 | 0.513 | <0.001 | 942 |
| **Left superior temporal gyrus** | -0.051 | 0.033 | [-0.115 - 0.014] | -0.620 | 0.125 | 0.513 | <0.001 | 907 |
| **Left precentral gyrus** | -0.047 | 0.032 | [-0.111 - 0.017] | -0.541 | 0.147 | 0.513 | <0.001 | 940 |
| **Right inferior parietal cortex** | -0.047 | 0.032 | [-0.111 - 0.017] | -0.533 | 0.148 | 0.513 | <0.001 | 939 |
| **Left supramarginal gyrus** | -0.046 | 0.033 | [-0.11 - 0.018] | -0.498 | 0.159 | 0.513 | <0.001 | 926 |
| **Right hemisphere average thickness** | -0.046 | 0.032 | [-0.109 - 0.018] | -0.336 | 0.157 | 0.513 | <0.001 | 943 |
| **Left pars triangularis** | -0.046 | 0.032 | [-0.109 - 0.018] | -0.557 | 0.159 | 0.513 | <0.001 | 939 |
| **Right precentral gyrus** | -0.046 | 0.032 | [-0.109 - 0.018] | -0.542 | 0.161 | 0.513 | <0.001 | 940 |
| **Right rostral middle frontal gyrus** | -0.041 | 0.032 | [-0.105 - 0.022] | -0.400 | 0.202 | 0.554 | <0.001 | 940 |
| **Right frontal pole** | -0.041 | 0.033 | [-0.104 - 0.023] | -0.847 | 0.213 | 0.554 | <0.001 | 940 |
| **Left lingual gyrus** | -0.040 | 0.056 | [-0.151 - 0.07] | -0.468 | 0.474 | 0.851 | 55.811 | 937 |
| **Right pars opercularis** | -0.040 | 0.033 | [-0.104 - 0.024] | -0.405 | 0.217 | 0.554 | <0.001 | 939 |
| **Left frontal pole** | -0.040 | 0.032 | [-0.103 - 0.024] | -0.837 | 0.222 | 0.554 | <0.001 | 941 |
| **Left superior frontal gyrus** | -0.039 | 0.032 | [-0.102 - 0.025] | -0.390 | 0.235 | 0.561 | <0.001 | 941 |
| **Right lateral occipital cortex** | -0.033 | 0.032 | [-0.096 - 0.031] | -0.381 | 0.315 | 0.689 | <0.001 | 939 |
| **Left lateral orbitofrontal cortex** | -0.032 | 0.033 | [-0.096 - 0.032] | -0.385 | 0.328 | 0.695 | <0.001 | 939 |
| **Left posterior cingulate cortex** | -0.030 | 0.032 | [-0.094 - 0.033] | -0.381 | 0.349 | 0.697 | <0.001 | 943 |
| **Right paracentral lobule** | -0.028 | 0.033 | [-0.092 - 0.035] | -0.322 | 0.383 | 0.725 | <0.001 | 942 |
| **Right medial orbitofrontal cortex** | -0.028 | 0.052 | [-0.13 - 0.075] | -0.465 | 0.598 | 0.881 | 48.871 | 938 |
| **Left superior parietal cortex** | -0.021 | 0.032 | [-0.085 - 0.042] | -0.208 | 0.511 | 0.875 | <0.001 | 939 |
| **Right isthmus cingulate cortex** | -0.021 | 0.032 | [-0.085 - 0.042] | -0.337 | 0.513 | 0.875 | <0.001 | 940 |
| **Right insula** | -0.019 | 0.047 | [-0.111 - 0.073] | -0.207 | 0.686 | 0.905 | 37.936 | 939 |
| **Right lateral orbitofrontal cortex** | -0.019 | 0.032 | [-0.082 - 0.045] | -0.220 | 0.563 | 0.881 | <0.001 | 943 |
| **Right transverse temporal gyrus** | -0.018 | 0.039 | [-0.093 - 0.058] | -0.284 | 0.647 | 0.889 | 17.518 | 936 |
| **Left transverse temporal gyrus** | -0.017 | 0.033 | [-0.081 - 0.046] | -0.275 | 0.593 | 0.881 | <0.001 | 937 |
| **Right inferior temporal gyrus** | -0.017 | 0.053 | [-0.122 - 0.088] | -0.183 | 0.752 | 0.929 | 49.606 | 933 |
| **Left fusiform gyrus** | -0.016 | 0.058 | [-0.129 - 0.098] | -0.155 | 0.786 | 0.929 | 57.712 | 940 |
| **Left pars orbitalis** | -0.015 | 0.052 | [-0.116 - 0.086] | -0.221 | 0.769 | 0.929 | 47.894 | 943 |
| **Right cuneus** | -0.014 | 0.032 | [-0.078 - 0.049] | -0.173 | 0.663 | 0.893 | <0.001 | 938 |
| **Right posterior cingulate cortex** | -0.013 | 0.053 | [-0.117 - 0.091] | -0.179 | 0.801 | 0.929 | 50.613 | 943 |
| **Right supramarginal gyrus** | -0.011 | 0.033 | [-0.075 - 0.053] | -0.113 | 0.735 | 0.929 | <0.001 | 935 |
| **Right temporal pole** | -0.009 | 0.033 | [-0.073 - 0.056] | -0.191 | 0.792 | 0.929 | <0.001 | 925 |
| **Right superior temporal gyrus** | -0.008 | 0.033 | [-0.073 - 0.057] | -0.095 | 0.811 | 0.929 | <0.001 | 906 |
| **Right middle temporal gyrus** | -0.007 | 0.032 | [-0.071 - 0.056] | -0.081 | 0.823 | 0.929 | <0.001 | 935 |
| **Right lingual gyrus** | -0.005 | 0.033 | [-0.069 - 0.059] | -0.060 | 0.873 | 0.955 | <0.001 | 935 |
| **Right pars orbitalis** | -0.003 | 0.033 | [-0.066 - 0.061] | -0.040 | 0.932 | 0.966 | <0.001 | 942 |
| **Left rostral anterior cingulate cortex** | -0.003 | 0.032 | [-0.066 - 0.061] | -0.049 | 0.938 | 0.966 | <0.001 | 938 |
| **Right fusiform gyrus** | -1.45E-05 | 0.032 | [-0.064 - 0.064] | -1.41E-04 | 1.000 | 1.000 | <0.001 | 942 |
| **Left isthmus cingulate cortex** | 0.002 | 0.039 | [-0.075 - 0.079] | 0.030 | 0.960 | 0.974 | 19.802 | 941 |
| **Left entorhinal cortex** | 0.003 | 0.033 | [-0.061 - 0.068] | 0.073 | 0.924 | 0.966 | <0.001 | 921 |
| **Left middle temporal gyrus** | 0.008 | 0.033 | [-0.056 - 0.073] | 0.098 | 0.802 | 0.929 | <0.001 | 922 |
| **Left parahippocampal gyrus** | 0.010 | 0.112 | [-0.209 - 0.228] | 0.237 | 0.932 | 0.966 | 90.246 | 941 |
| **Left medial orbitofrontal cortex** | 0.010 | 0.060 | [-0.106 - 0.127] | 0.162 | 0.861 | 0.955 | 59.489 | 930 |
| **Left lateral occipital cortex** | 0.024 | 0.050 | [-0.074 - 0.123] | 0.280 | 0.628 | 0.881 | 45.096 | 940 |
| **Right caudal anterior cingulate cortex** | 0.026 | 0.051 | [-0.075 - 0.126] | 0.572 | 0.617 | 0.881 | 46.933 | 941 |
| **Left banks superior temporal sulcus** | 0.030 | 0.053 | [-0.074 - 0.135] | 0.441 | 0.572 | 0.881 | 49.138 | 915 |
| **Left inferior temporal gyrus** | 0.031 | 0.033 | [-0.033 - 0.095] | 0.348 | 0.341 | 0.697 | <0.001 | 930 |
| **Right entorhinal cortex** | 0.037 | 0.033 | [-0.028 - 0.101] | 0.870 | 0.267 | 0.603 | <0.001 | 917 |
| **Left caudal anterior cingulate cortex** | 0.038 | 0.033 | [-0.026 - 0.102] | 0.868 | 0.241 | 0.561 | <0.001 | 941 |
| **Right pericalcarine cortex** | 0.040 | 0.072 | [-0.102 - 0.181] | 0.578 | 0.584 | 0.881 | 73.146 | 939 |
| **Right parahippocampal gyrus** | 0.042 | 0.086 | [-0.127 - 0.21] | 0.908 | 0.629 | 0.881 | 82.082 | 939 |
| **Left pericalcarine cortex** | 0.047 | 0.094 | [-0.138 - 0.232] | 0.659 | 0.619 | 0.881 | 85.451 | 937 |
| **Left temporal pole** | 0.056 | 0.066 | [-0.074 - 0.185] | 1.131 | 0.398 | 0.733 | 66.592 | 925 |
| **Right rostral anterior cingulate cortex** | 0.101 | 0.076 | [-0.048 - 0.25] | 2.060 | 0.184 | 0.540 | 76.805 | 941 |
| **Left cuneus** | 0.122 | 0.137 | [-0.146 - 0.391] | 1.523 | 0.371 | 0.721 | 96.204 | 939 |

**^a^** Included Samples: CLING, Houston, Sexpect, MPIP, Muenster Cohort, SHIP, SHIP-trend, Stanford.

BDI-II: Beck Depression Inventory.

**Adult meta-analyses results for surface area**

**Supplementary Table S19**: Full meta-analytic results for surface area of each structure for MDD patients versus Controls comparison controlling for age, sex and scan center. Adjusted Cohen's d is reported.

|  | **Cohen's d ^a^** | **Std. Err.** | **95% CI** | **% Difference** | **P-value** | **FDR P-value** | **I^2^** | **# Controls** | **# Patients** |
| --- | --- | --- | --- | --- | --- | --- | --- | --- | --- |
|  | **(MDD vs CTL)** |  |  |  |  |  |  |  |  |
| **Left pars triangularis** | -0.063 | 0.030 | [-0.121 - -0.004] | -0.381 | 0.037 | 0.870 | <0.001 | 7634 | 1894 |
| **Left frontal pole** | -0.049 | 0.030 | [-0.108 - 0.009] | -0.520 | 0.100 | 0.920 | <0.001 | 7657 | 1900 |
| **Right parahippocampal gyrus** | -0.040 | 0.030 | [-0.099 - 0.019] | -0.437 | 0.184 | 0.920 | <0.001 | 7606 | 1881 |
| **Right fusiform gyrus** | -0.034 | 0.041 | [-0.114 - 0.045] | -0.168 | 0.395 | 0.920 | 29.984 | 7523 | 1860 |
| **Left middle temporal gyrus** | -0.034 | 0.051 | [-0.134 - 0.067] | -0.199 | 0.513 | 0.920 | 52.691 | 7526 | 1802 |
| **Right inferior parietal cortex** | -0.024 | 0.050 | [-0.121 - 0.073] | -0.136 | 0.629 | 0.920 | 51.660 | 7584 | 1877 |
| **Left banks superior temporal sulcus** | -0.024 | 0.043 | [-0.109 - 0.061] | -0.175 | 0.582 | 0.920 | 34.538 | 7487 | 1748 |
| **Right transverse temporal gyrus** | -0.021 | 0.044 | [-0.107 - 0.064] | -0.171 | 0.625 | 0.920 | 38.876 | 7656 | 1901 |
| **Left parahippocampal gyrus** | -0.017 | 0.048 | [-0.11 - 0.077] | -0.210 | 0.725 | 0.920 | 47.899 | 7592 | 1879 |
| **Left pars opercularis** | -0.015 | 0.030 | [-0.074 - 0.044] | -0.071 | 0.620 | 0.920 | <0.001 | 7619 | 1889 |
| **Right pars triangularis** | -0.012 | 0.030 | [-0.071 - 0.047] | -0.071 | 0.684 | 0.920 | <0.001 | 7622 | 1892 |
| **Right pars opercularis** | -0.011 | 0.046 | [-0.102 - 0.079] | -0.057 | 0.806 | 0.920 | 45.081 | 7619 | 1886 |
| **Left pericalcarine cortex** | -0.008 | 0.062 | [-0.13 - 0.113] | -0.059 | 0.893 | 0.920 | 69.732 | 7654 | 1895 |
| **Right superior parietal cortex** | -0.008 | 0.037 | [-0.081 - 0.065] | -0.039 | 0.833 | 0.920 | 21.080 | 7610 | 1873 |
| **Left cuneus** | -0.007 | 0.030 | [-0.066 - 0.052] | -0.043 | 0.818 | 0.920 | <0.001 | 7626 | 1889 |
| **Left inferior parietal cortex** | -0.005 | 0.030 | [-0.065 - 0.054] | -0.031 | 0.859 | 0.920 | <0.001 | 7587 | 1876 |
| **Right insula** | -0.004 | 0.051 | [-0.103 - 0.095] | -0.022 | 0.936 | 0.949 | 53.499 | 7641 | 1889 |
| **Left pars orbitalis** | -0.002 | 0.051 | [-0.103 - 0.098] | -0.016 | 0.965 | 0.965 | 55.122 | 7647 | 1899 |
| **Right supramarginal gyrus** | 0.004 | 0.030 | [-0.055 - 0.064] | 0.022 | 0.890 | 0.920 | <0.001 | 7569 | 1855 |
| **Right inferior temporal gyrus** | 0.005 | 0.030 | [-0.054 - 0.064] | 0.025 | 0.876 | 0.920 | <0.001 | 7634 | 1885 |
| **Right precuneus** | 0.006 | 0.034 | [-0.061 - 0.072] | 0.028 | 0.870 | 0.920 | 11.029 | 7635 | 1894 |
| **Left posterior cingulate cortex** | 0.006 | 0.030 | [-0.053 - 0.064] | 0.035 | 0.851 | 0.920 | <0.001 | 7645 | 1896 |
| **Left postcentral gyrus** | 0.006 | 0.035 | [-0.063 - 0.075] | 0.034 | 0.862 | 0.920 | 14.485 | 7571 | 1866 |
| **Right pericalcarine cortex** | 0.007 | 0.034 | [-0.059 - 0.073] | 0.050 | 0.837 | 0.920 | 10.451 | 7650 | 1897 |
| **Right hemisphere total surface area** | 0.007 | 0.042 | [-0.075 - 0.09] | 0.078 | 0.860 | 0.920 | 35.531 | 7658 | 1902 |
| **Left entorhinal cortex** | 0.008 | 0.043 | [-0.077 - 0.093] | 0.090 | 0.856 | 0.920 | 36.689 | 7434 | 1821 |
| **Right precentral gyrus** | 0.009 | 0.057 | [-0.103 - 0.121] | 0.053 | 0.875 | 0.920 | 63.917 | 7589 | 1880 |
| **Right caudal middle frontal gyrus** | 0.009 | 0.030 | [-0.05 - 0.068] | 0.048 | 0.761 | 0.920 | <0.001 | 7620 | 1889 |
| **Right banks superior temporal sulcus** | 0.010 | 0.031 | [-0.051 - 0.07] | 0.070 | 0.751 | 0.920 | 1.282 | 7569 | 1815 |
| **Left precuneus** | 0.011 | 0.043 | [-0.073 - 0.095] | 0.052 | 0.798 | 0.920 | 36.862 | 7636 | 1892 |
| **Left superior temporal gyrus** | 0.011 | 0.050 | [-0.088 - 0.11] | 0.067 | 0.827 | 0.920 | 50.102 | 7510 | 1787 |
| **Right caudal anterior cingulate cortex** | 0.011 | 0.030 | [-0.048 - 0.07] | 0.126 | 0.707 | 0.920 | <0.001 | 7615 | 1883 |
| **Left fusiform gyrus** | 0.012 | 0.030 | [-0.047 - 0.072] | 0.060 | 0.688 | 0.920 | <0.001 | 7503 | 1862 |
| **Left transverse temporal gyrus** | 0.013 | 0.056 | [-0.097 - 0.123] | 0.104 | 0.815 | 0.920 | 62.951 | 7654 | 1899 |
| **Left insula** | 0.014 | 0.049 | [-0.082 - 0.109] | 0.071 | 0.782 | 0.920 | 50.651 | 7634 | 1896 |
| **Right paracentral lobule** | 0.014 | 0.034 | [-0.052 - 0.081] | 0.080 | 0.676 | 0.920 | 10.905 | 7593 | 1881 |
| **Left lateral occipital cortex** | 0.016 | 0.055 | [-0.092 - 0.123] | 0.090 | 0.776 | 0.920 | 60.795 | 7633 | 1892 |
| **Right lingual gyrus** | 0.016 | 0.063 | [-0.107 - 0.138] | 0.092 | 0.799 | 0.920 | 70.312 | 7649 | 1897 |
| **Right posterior cingulate cortex** | 0.016 | 0.061 | [-0.104 - 0.137] | 0.110 | 0.789 | 0.920 | 69.174 | 7648 | 1898 |
| **Left superior parietal cortex** | 0.017 | 0.030 | [-0.042 - 0.076] | 0.083 | 0.575 | 0.920 | <0.001 | 7592 | 1877 |
| **Left paracentral lobule** | 0.018 | 0.068 | [-0.115 - 0.151] | 0.104 | 0.788 | 0.920 | 74.869 | 7547 | 1865 |
| **Left caudal anterior cingulate cortex** | 0.021 | 0.030 | [-0.038 - 0.08] | 0.242 | 0.480 | 0.920 | <0.001 | 7590 | 1883 |
| **Right postcentral gyrus** | 0.022 | 0.030 | [-0.037 - 0.082] | 0.126 | 0.460 | 0.920 | <0.001 | 7596 | 1874 |
| **Right pars orbitalis** | 0.022 | 0.041 | [-0.059 - 0.104] | 0.161 | 0.589 | 0.920 | 33.355 | 7650 | 1897 |
| **Right frontal pole** | 0.024 | 0.044 | [-0.062 - 0.111] | 0.254 | 0.579 | 0.920 | 39.785 | 7652 | 1900 |
| **Left rostral middle frontal gyrus** | 0.025 | 0.030 | [-0.033 - 0.084] | 0.123 | 0.397 | 0.920 | <0.001 | 7634 | 1897 |
| **Left hemisphere total surface area** | 0.026 | 0.044 | [-0.06 - 0.111] | 0.266 | 0.560 | 0.920 | 39.531 | 7658 | 1902 |
| **Right lateral orbitofrontal cortex** | 0.026 | 0.054 | [-0.08 - 0.132] | 0.153 | 0.631 | 0.920 | 60.082 | 7654 | 1902 |
| **Right medial orbitofrontal cortex** | 0.027 | 0.030 | [-0.032 - 0.086] | 0.227 | 0.369 | 0.920 | <0.001 | 7606 | 1883 |
| **Right middle temporal gyrus** | 0.027 | 0.030 | [-0.032 - 0.086] | 0.151 | 0.370 | 0.920 | <0.001 | 7593 | 1867 |
| **Right superior frontal gyrus** | 0.028 | 0.030 | [-0.032 - 0.087] | 0.134 | 0.361 | 0.920 | <0.001 | 7589 | 1877 |
| **Left lingual gyrus** | 0.028 | 0.059 | [-0.087 - 0.144] | 0.163 | 0.632 | 0.920 | 66.292 | 7640 | 1894 |
| **Right superior temporal gyrus** | 0.029 | 0.062 | [-0.092 - 0.15] | 0.173 | 0.640 | 0.920 | 67.516 | 7547 | 1805 |
| **Right rostral anterior cingulate cortex** | 0.029 | 0.030 | [-0.03 - 0.088] | 0.297 | 0.333 | 0.920 | <0.001 | 7595 | 1877 |
| **Left precentral gyrus** | 0.034 | 0.072 | [-0.106 - 0.175] | 0.197 | 0.631 | 0.920 | 77.695 | 7587 | 1878 |
| **Left medial orbitofrontal cortex** | 0.035 | 0.059 | [-0.08 - 0.15] | 0.270 | 0.552 | 0.920 | 65.276 | 7554 | 1869 |
| **Right entorhinal cortex** | 0.036 | 0.031 | [-0.024 - 0.097] | 0.433 | 0.240 | 0.920 | <0.001 | 7409 | 1796 |
| **Left supramarginal gyrus** | 0.040 | 0.043 | [-0.044 - 0.123] | 0.214 | 0.350 | 0.920 | 34.285 | 7512 | 1835 |
| **Left rostral anterior cingulate cortex** | 0.040 | 0.030 | [-0.019 - 0.1] | 0.391 | 0.186 | 0.920 | <0.001 | 7540 | 1861 |
| **Right isthmus cingulate cortex** | 0.040 | 0.047 | [-0.052 - 0.133] | 0.321 | 0.393 | 0.920 | 47.534 | 7642 | 1896 |
| **Left lateral orbitofrontal cortex** | 0.041 | 0.036 | [-0.03 - 0.113] | 0.250 | 0.253 | 0.920 | 18.652 | 7655 | 1901 |
| **Left caudal middle frontal gyrus** | 0.045 | 0.053 | [-0.058 - 0.148] | 0.231 | 0.389 | 0.920 | 57.053 | 7605 | 1885 |
| **Left inferior temporal gyrus** | 0.046 | 0.044 | [-0.04 - 0.133] | 0.258 | 0.294 | 0.920 | 38.790 | 7623 | 1869 |
| **Right temporal pole** | 0.051 | 0.052 | [-0.051 - 0.153] | 0.561 | 0.327 | 0.920 | 55.319 | 7574 | 1856 |
| **Left superior frontal gyrus** | 0.052 | 0.030 | [-0.008 - 0.111] | 0.261 | 0.088 | 0.920 | <0.001 | 7555 | 1879 |
| **Right rostral middle frontal gyrus** | 0.054 | 0.030 | [-0.005 - 0.113] | 0.262 | 0.072 | 0.920 | <0.001 | 7636 | 1890 |
| **Right cuneus** | 0.058 | 0.036 | [-0.013 - 0.13] | 0.359 | 0.107 | 0.920 | 18.350 | 7634 | 1891 |
| **Left isthmus cingulate cortex** | 0.063 | 0.058 | [-0.052 - 0.177] | 0.482 | 0.283 | 0.920 | 65.722 | 7641 | 1893 |
| **Left temporal pole** | 0.066 | 0.030 | [0.007 - 0.125] | 0.665 | 0.029 | 0.870 | <0.001 | 7620 | 1868 |
| **Right lateral occipital cortex** | 0.067 | 0.030 | [0.008 - 0.126] | 0.391 | 0.026 | 0.870 | <0.001 | 7642 | 1897 |

MDD: Major Depressive Disorder; CTL: Controls.

**Supplementary Table S20**: Full meta-analytic results for surface area of each structure for the Diagnosis by Sex interaction controlling for age, sex and scan center. Adjusted Cohen's d is reported.

|  | **Cohen's d ^a^** | **Std. Err.** | **95% CI** | **% Difference** | **P-value** | **FDR P-value** | **I^2^** | **# Controls** | **# Patients** |
| --- | --- | --- | --- | --- | --- | --- | --- | --- | --- |
|  | **(Dx by Sex)** |  |  |  |  |  |  |  |  |
| **Left fusiform gyrus** | -0.085 | 0.030 | [-0.145 - -0.026] | -0.420 | 0.005 | 0.349 | <0.001 | 7503 | 1862 |
| **Right caudal middle frontal gyrus** | -0.078 | 0.034 | [-0.145 - -0.012] | -0.413 | 0.021 | 0.448 | 11.290 | 7620 | 1889 |
| **Left lateral orbitofrontal cortex** | -0.073 | 0.033 | [-0.138 - -0.007] | -0.438 | 0.029 | 0.448 | 9.722 | 7655 | 1901 |
| **Left parahippocampal gyrus** | -0.072 | 0.044 | [-0.158 - 0.013] | -0.902 | 0.096 | 0.448 | 38.204 | 7592 | 1879 |
| **Right precentral gyrus** | -0.070 | 0.039 | [-0.146 - 0.006] | -0.417 | 0.071 | 0.448 | 25.696 | 7589 | 1880 |
| **Left middle temporal gyrus** | -0.068 | 0.035 | [-0.137 - 0.001] | -0.405 | 0.053 | 0.448 | 13.071 | 7526 | 1802 |
| **Right fusiform gyrus** | -0.066 | 0.040 | [-0.145 - 0.013] | -0.324 | 0.100 | 0.448 | 29.514 | 7523 | 1860 |
| **Right isthmus cingulate cortex** | -0.066 | 0.030 | [-0.125 - -0.007] | -0.520 | 0.029 | 0.448 | 0.014 | 7642 | 1896 |
| **Left frontal pole** | -0.062 | 0.030 | [-0.121 - -0.004] | -0.658 | 0.037 | 0.448 | <0.001 | 7657 | 1900 |
| **Left pars orbitalis** | -0.060 | 0.030 | [-0.119 - -0.002] | -0.442 | 0.044 | 0.448 | <0.001 | 7647 | 1899 |
| **Right inferior temporal gyrus** | -0.059 | 0.031 | [-0.12 - 0.002] | -0.319 | 0.060 | 0.448 | 3.147 | 7634 | 1885 |
| **Right lateral occipital cortex** | -0.057 | 0.034 | [-0.123 - 0.009] | -0.334 | 0.088 | 0.448 | 10.239 | 7642 | 1897 |
| **Right precuneus** | -0.055 | 0.040 | [-0.133 - 0.023] | -0.273 | 0.166 | 0.464 | 28.281 | 7635 | 1894 |
| **Left inferior temporal gyrus** | -0.053 | 0.030 | [-0.113 - 0.006] | -0.298 | 0.077 | 0.448 | <0.001 | 7623 | 1869 |
| **Right postcentral gyrus** | -0.052 | 0.033 | [-0.117 - 0.013] | -0.296 | 0.115 | 0.448 | 8.643 | 7596 | 1874 |
| **Right middle temporal gyrus** | -0.052 | 0.033 | [-0.116 - 0.013] | -0.288 | 0.115 | 0.448 | 7.173 | 7593 | 1867 |
| **Right frontal pole** | -0.052 | 0.036 | [-0.122 - 0.019] | -0.538 | 0.150 | 0.464 | 17.042 | 7652 | 1900 |
| **Left superior parietal cortex** | -0.051 | 0.030 | [-0.11 - 0.008] | -0.249 | 0.093 | 0.448 | <0.001 | 7592 | 1877 |
| **Left insula** | -0.049 | 0.030 | [-0.108 - 0.009] | -0.258 | 0.100 | 0.448 | 0.002 | 7634 | 1896 |
| **Left rostral middle frontal gyrus** | -0.048 | 0.039 | [-0.125 - 0.028] | -0.234 | 0.215 | 0.508 | 26.456 | 7634 | 1897 |
| **Left postcentral gyrus** | -0.048 | 0.030 | [-0.108 - 0.011] | -0.266 | 0.112 | 0.448 | <0.001 | 7571 | 1866 |
| **Left rostral anterior cingulate cortex** | -0.047 | 0.033 | [-0.111 - 0.017] | -0.459 | 0.149 | 0.464 | 6.694 | 7540 | 1861 |
| **Right pars opercularis** | -0.046 | 0.030 | [-0.105 - 0.013] | -0.233 | 0.124 | 0.456 | 0.005 | 7619 | 1886 |
| **Right rostral anterior cingulate cortex** | -0.046 | 0.033 | [-0.11 - 0.018] | -0.467 | 0.161 | 0.464 | 7.633 | 7595 | 1877 |
| **Left banks superior temporal sulcus** | -0.046 | 0.043 | [-0.131 - 0.04] | -0.334 | 0.294 | 0.599 | 34.862 | 7487 | 1748 |
| **Right rostral middle frontal gyrus** | -0.045 | 0.030 | [-0.104 - 0.014] | -0.217 | 0.135 | 0.464 | <0.001 | 7636 | 1890 |
| **Right banks superior temporal sulcus** | -0.043 | 0.030 | [-0.103 - 0.016] | -0.309 | 0.154 | 0.464 | <0.001 | 7569 | 1815 |
| **Right superior temporal gyrus** | -0.042 | 0.032 | [-0.105 - 0.021] | -0.253 | 0.187 | 0.503 | 3.723 | 7547 | 1805 |
| **Left caudal middle frontal gyrus** | -0.042 | 0.034 | [-0.109 - 0.025] | -0.215 | 0.218 | 0.508 | 12.230 | 7605 | 1885 |
| **Right parahippocampal gyrus** | -0.042 | 0.040 | [-0.121 - 0.038] | -0.453 | 0.304 | 0.599 | 30.262 | 7606 | 1881 |
| **Left precentral gyrus** | -0.041 | 0.033 | [-0.106 - 0.024] | -0.236 | 0.215 | 0.508 | 8.786 | 7587 | 1878 |
| **Left lateral occipital cortex** | -0.040 | 0.039 | [-0.117 - 0.037] | -0.230 | 0.310 | 0.599 | 27.373 | 7633 | 1892 |
| **Right pars orbitalis** | -0.037 | 0.030 | [-0.096 - 0.022] | -0.267 | 0.217 | 0.508 | 0.019 | 7650 | 1897 |
| **Right insula** | -0.037 | 0.036 | [-0.108 - 0.034] | -0.201 | 0.308 | 0.599 | 18.109 | 7641 | 1889 |
| **Left hemisphere total surface area** | -0.037 | 0.040 | [-0.115 - 0.041] | -0.381 | 0.358 | 0.642 | 28.999 | 7658 | 1902 |
| **Left superior frontal gyrus** | -0.035 | 0.035 | [-0.103 - 0.033] | -0.176 | 0.317 | 0.599 | 13.727 | 7555 | 1879 |
| **Right superior parietal cortex** | -0.035 | 0.030 | [-0.094 - 0.025] | -0.173 | 0.253 | 0.568 | <0.001 | 7610 | 1873 |
| **Left medial orbitofrontal cortex** | -0.034 | 0.040 | [-0.113 - 0.045] | -0.266 | 0.396 | 0.675 | 29.591 | 7554 | 1869 |
| **Right hemisphere total surface area** | -0.034 | 0.030 | [-0.093 - 0.025] | -0.352 | 0.260 | 0.568 | <0.001 | 7658 | 1902 |
| **Right medial orbitofrontal cortex** | -0.032 | 0.038 | [-0.106 - 0.042] | -0.269 | 0.395 | 0.675 | 22.117 | 7606 | 1883 |
| **Left superior temporal gyrus** | -0.029 | 0.051 | [-0.13 - 0.071] | -0.179 | 0.566 | 0.766 | 51.568 | 7510 | 1787 |
| **Left isthmus cingulate cortex** | -0.028 | 0.030 | [-0.087 - 0.03] | -0.218 | 0.345 | 0.635 | <0.001 | 7641 | 1893 |
| **Left precuneus** | -0.027 | 0.047 | [-0.119 - 0.065] | -0.127 | 0.569 | 0.766 | 46.340 | 7636 | 1892 |
| **Right supramarginal gyrus** | -0.025 | 0.037 | [-0.097 - 0.047] | -0.128 | 0.499 | 0.766 | 19.277 | 7569 | 1855 |
| **Left paracentral lobule** | -0.024 | 0.030 | [-0.084 - 0.035] | -0.138 | 0.423 | 0.705 | 0.001 | 7547 | 1865 |
| **Left pars triangularis** | -0.024 | 0.040 | [-0.102 - 0.054] | -0.145 | 0.549 | 0.766 | 28.390 | 7634 | 1894 |
| **Left entorhinal cortex** | -0.023 | 0.043 | [-0.106 - 0.061] | -0.262 | 0.593 | 0.783 | 34.449 | 7434 | 1821 |
| **Right lingual gyrus** | -0.023 | 0.030 | [-0.082 - 0.036] | -0.132 | 0.450 | 0.732 | 0.009 | 7649 | 1897 |
| **Right temporal pole** | -0.020 | 0.030 | [-0.079 - 0.04] | -0.217 | 0.515 | 0.766 | <0.001 | 7574 | 1856 |
| **Right superior frontal gyrus** | -0.019 | 0.042 | [-0.103 - 0.064] | -0.095 | 0.646 | 0.822 | 35.532 | 7589 | 1877 |
| **Left temporal pole** | -0.019 | 0.030 | [-0.078 - 0.04] | -0.193 | 0.527 | 0.766 | <0.001 | 7620 | 1868 |
| **Left inferior parietal cortex** | -0.019 | 0.032 | [-0.082 - 0.044] | -0.108 | 0.552 | 0.766 | 5.317 | 7587 | 1876 |
| **Left transverse temporal gyrus** | -0.018 | 0.030 | [-0.076 - 0.041] | -0.139 | 0.557 | 0.766 | <0.001 | 7654 | 1899 |
| **Right transverse temporal gyrus** | -0.016 | 0.031 | [-0.077 - 0.045] | -0.125 | 0.618 | 0.802 | 3.279 | 7656 | 1901 |
| **Right inferior parietal cortex** | -0.014 | 0.038 | [-0.088 - 0.061] | -0.079 | 0.713 | 0.857 | 23.264 | 7584 | 1877 |
| **Right lateral orbitofrontal cortex** | -0.014 | 0.038 | [-0.089 - 0.062] | -0.080 | 0.722 | 0.857 | 25.012 | 7654 | 1902 |
| **Right paracentral lobule** | -0.013 | 0.046 | [-0.103 - 0.077] | -0.075 | 0.773 | 0.901 | 44.176 | 7593 | 1881 |
| **Right posterior cingulate cortex** | -0.012 | 0.030 | [-0.071 - 0.047] | -0.078 | 0.696 | 0.855 | <0.001 | 7648 | 1898 |
| **Left caudal anterior cingulate cortex** | -0.008 | 0.046 | [-0.098 - 0.081] | -0.095 | 0.854 | 0.949 | 43.323 | 7590 | 1883 |
| **Left posterior cingulate cortex** | -0.006 | 0.030 | [-0.065 - 0.052] | -0.041 | 0.829 | 0.939 | <0.001 | 7645 | 1896 |
| **Right caudal anterior cingulate cortex** | -0.006 | 0.030 | [-0.065 - 0.053] | -0.071 | 0.832 | 0.939 | <0.001 | 7615 | 1883 |
| **Left supramarginal gyrus** | -0.005 | 0.049 | [-0.101 - 0.092] | -0.025 | 0.925 | 0.977 | 49.744 | 7512 | 1835 |
| **Right cuneus** | -0.003 | 0.032 | [-0.066 - 0.061] | -0.016 | 0.935 | 0.977 | 6.364 | 7634 | 1891 |
| **Left cuneus** | 0.000 | 0.032 | [-0.064 - 0.063] | -0.002 | 0.993 | 0.999 | 6.325 | 7626 | 1889 |
| **Left pars opercularis** | 8.23E-05 | 0.053 | [-0.105 - 0.105] | 3.92E-04 | 0.999 | 0.999 | 58.475 | 7619 | 1889 |
| **Right pars triangularis** | 0.002 | 0.039 | [-0.075 - 0.079] | 0.012 | 0.956 | 0.984 | 27.352 | 7622 | 1892 |
| **Left pericalcarine cortex** | 0.004 | 0.043 | [-0.081 - 0.089] | 0.031 | 0.920 | 0.977 | 37.986 | 7654 | 1895 |
| **Right entorhinal cortex** | 0.005 | 0.046 | [-0.086 - 0.096] | 0.060 | 0.913 | 0.977 | 43.377 | 7409 | 1796 |
| **Right pericalcarine cortex** | 0.015 | 0.038 | [-0.059 - 0.089] | 0.112 | 0.685 | 0.855 | 23.073 | 7650 | 1897 |
| **Left lingual gyrus** | 0.029 | 0.043 | [-0.056 - 0.113] | 0.167 | 0.503 | 0.766 | 37.692 | 7640 | 1894 |

Dx: Diagnosis.

**Supplementary Table S21**: Full meta-analytic results for surface area of each structure for the Diagnosis by Age interaction controlling for age, sex and scan center. Adjusted Cohen's d is reported.

|  | **Cohen's d ^a^** | **Std. Err.** | **95% CI** | **% Difference** | **P-value** | **FDR P-value** | **I^2^** | **# Controls** | **# Patients** |
| --- | --- | --- | --- | --- | --- | --- | --- | --- | --- |
|  | **(Dx by Age)** |  |  |  |  |  |  |  |  |
| **Right caudal middle frontal gyrus** | -0.065 | 0.047 | [-0.157 - 0.027] | -0.340 | 0.169 | 0.309 | 46.393 | 7620 | 1889 |
| **Left caudal middle frontal gyrus** | -0.009 | 0.033 | [-0.073 - 0.056] | -0.044 | 0.795 | 0.884 | 8.124 | 7605 | 1885 |
| **Right superior temporal gyrus** | -0.007 | 0.038 | [-0.081 - 0.067] | -0.041 | 0.855 | 0.892 | 20.152 | 7547 | 1805 |
| **Left banks superior temporal sulcus** | -9.32E-04 | 0.091 | [-0.179 - 0.177] | -0.007 | 0.992 | 0.992 | 85.658 | 7487 | 1748 |
| **Left paracentral lobule** | 0.001 | 0.043 | [-0.084 - 0.086] | 0.006 | 0.980 | 0.992 | 37.916 | 7547 | 1865 |
| **Right banks superior temporal sulcus** | 0.005 | 0.032 | [-0.057 - 0.068] | 0.038 | 0.866 | 0.892 | 3.557 | 7569 | 1815 |
| **Left pars triangularis** | 0.006 | 0.030 | [-0.053 - 0.065] | 0.035 | 0.849 | 0.892 | <0.001 | 7634 | 1894 |
| **Right temporal pole** | 0.008 | 0.044 | [-0.078 - 0.094] | 0.089 | 0.854 | 0.892 | 38.526 | 7574 | 1856 |
| **Right frontal pole** | 0.009 | 0.048 | [-0.084 - 0.103] | 0.098 | 0.844 | 0.892 | 48.463 | 7652 | 1900 |
| **Right entorhinal cortex** | 0.010 | 0.031 | [-0.05 - 0.071] | 0.125 | 0.735 | 0.844 | <0.001 | 7409 | 1796 |
| **Left temporal pole** | 0.014 | 0.044 | [-0.073 - 0.101] | 0.138 | 0.758 | 0.855 | 39.643 | 7620 | 1868 |
| **Left entorhinal cortex** | 0.015 | 0.031 | [-0.045 - 0.075] | 0.171 | 0.630 | 0.735 | <0.001 | 7434 | 1821 |
| **Left pars orbitalis** | 0.016 | 0.030 | [-0.043 - 0.074] | 0.114 | 0.604 | 0.721 | <0.001 | 7647 | 1899 |
| **Right fusiform gyrus** | 0.017 | 0.030 | [-0.043 - 0.077] | 0.083 | 0.578 | 0.710 | <0.001 | 7523 | 1860 |
| **Left pars opercularis** | 0.022 | 0.031 | [-0.039 - 0.083] | 0.103 | 0.489 | 0.646 | 2.956 | 7619 | 1889 |
| **Right parahippocampal gyrus** | 0.022 | 0.030 | [-0.037 - 0.081] | 0.240 | 0.465 | 0.626 | <0.001 | 7606 | 1881 |
| **Right paracentral lobule** | 0.023 | 0.030 | [-0.036 - 0.082] | 0.129 | 0.451 | 0.619 | <0.001 | 7593 | 1881 |
| **Left posterior cingulate cortex** | 0.023 | 0.037 | [-0.049 - 0.095] | 0.144 | 0.533 | 0.677 | 20.617 | 7645 | 1896 |
| **Left lateral orbitofrontal cortex** | 0.024 | 0.030 | [-0.034 - 0.083] | 0.147 | 0.416 | 0.595 | <0.001 | 7655 | 1901 |
| **Left parahippocampal gyrus** | 0.027 | 0.030 | [-0.032 - 0.086] | 0.334 | 0.374 | 0.587 | <0.001 | 7592 | 1879 |
| **Left medial orbitofrontal cortex** | 0.030 | 0.058 | [-0.083 - 0.142] | 0.229 | 0.608 | 0.721 | 63.959 | 7554 | 1869 |
| **Left transverse temporal gyrus** | 0.033 | 0.042 | [-0.05 - 0.116] | 0.262 | 0.431 | 0.604 | 35.544 | 7654 | 1899 |
| **Left superior temporal gyrus** | 0.034 | 0.056 | [-0.076 - 0.145] | 0.210 | 0.541 | 0.677 | 60.078 | 7510 | 1787 |
| **Right rostral anterior cingulate cortex** | 0.037 | 0.046 | [-0.053 - 0.127] | 0.379 | 0.416 | 0.595 | 43.726 | 7595 | 1877 |
| **Left superior parietal cortex** | 0.039 | 0.030 | [-0.021 - 0.098] | 0.189 | 0.202 | 0.354 | <0.001 | 7592 | 1877 |
| **Right precentral gyrus** | 0.041 | 0.066 | [-0.088 - 0.17] | 0.244 | 0.535 | 0.677 | 73.352 | 7589 | 1880 |
| **Right transverse temporal gyrus** | 0.041 | 0.030 | [-0.018 - 0.1] | 0.330 | 0.172 | 0.309 | <0.001 | 7656 | 1901 |
| **Right caudal anterior cingulate cortex** | 0.043 | 0.052 | [-0.06 - 0.145] | 0.474 | 0.416 | 0.595 | 56.522 | 7615 | 1883 |
| **Right superior frontal gyrus** | 0.043 | 0.050 | [-0.056 - 0.142] | 0.210 | 0.392 | 0.595 | 53.285 | 7589 | 1877 |
| **Right precuneus** | 0.045 | 0.042 | [-0.037 - 0.128] | 0.226 | 0.280 | 0.467 | 34.725 | 7635 | 1894 |
| **Right isthmus cingulate cortex** | 0.048 | 0.031 | [-0.014 - 0.11] | 0.379 | 0.128 | 0.280 | 4.146 | 7642 | 1896 |
| **Right insula** | 0.048 | 0.042 | [-0.034 - 0.13] | 0.263 | 0.249 | 0.425 | 34.164 | 7641 | 1889 |
| **Right medial orbitofrontal cortex** | 0.049 | 0.030 | [-0.01 - 0.108] | 0.412 | 0.104 | 0.262 | <0.001 | 7606 | 1883 |
| **Left insula** | 0.050 | 0.030 | [-0.009 - 0.109] | 0.259 | 0.098 | 0.262 | <0.001 | 7634 | 1896 |
| **Right pars opercularis** | 0.050 | 0.030 | [-0.009 - 0.109] | 0.253 | 0.094 | 0.262 | <0.001 | 7619 | 1886 |
| **Left frontal pole** | 0.051 | 0.032 | [-0.012 - 0.114] | 0.539 | 0.112 | 0.270 | 6.456 | 7657 | 1900 |
| **Left precuneus** | 0.054 | 0.030 | [-0.005 - 0.113] | 0.258 | 0.072 | 0.241 | <0.001 | 7636 | 1892 |
| **Right superior parietal cortex** | 0.055 | 0.030 | [-0.004 - 0.114] | 0.275 | 0.068 | 0.241 | <0.001 | 7610 | 1873 |
| **Left fusiform gyrus** | 0.056 | 0.038 | [-0.018 - 0.131] | 0.277 | 0.139 | 0.296 | 23.009 | 7503 | 1862 |
| **Left superior frontal gyrus** | 0.058 | 0.057 | [-0.055 - 0.17] | 0.292 | 0.313 | 0.510 | 63.951 | 7555 | 1879 |
| **Right rostral middle frontal gyrus** | 0.058 | 0.066 | [-0.071 - 0.187] | 0.280 | 0.378 | 0.587 | 73.300 | 7636 | 1890 |
| **Left isthmus cingulate cortex** | 0.058 | 0.030 | [-0.001 - 0.117] | 0.448 | 0.052 | 0.229 | <0.001 | 7641 | 1893 |
| **Right lateral occipital cortex** | 0.061 | 0.030 | [0.002 - 0.12] | 0.357 | 0.042 | 0.217 | <0.001 | 7642 | 1897 |
| **Left cuneus** | 0.062 | 0.030 | [0.003 - 0.121] | 0.382 | 0.040 | 0.217 | <0.001 | 7626 | 1889 |
| **Right hemisphere total surface area** | 0.063 | 0.044 | [-0.024 - 0.15] | 0.655 | 0.156 | 0.309 | 40.771 | 7658 | 1902 |
| **Left rostral anterior cingulate cortex** | 0.063 | 0.030 | [0.004 - 0.123] | 0.616 | 0.037 | 0.217 | <0.001 | 7540 | 1861 |
| **Right lateral orbitofrontal cortex** | 0.064 | 0.030 | [0.006 - 0.123] | 0.378 | 0.032 | 0.217 | <0.001 | 7654 | 1902 |
| **Left supramarginal gyrus** | 0.065 | 0.031 | [0.005 - 0.125] | 0.348 | 0.035 | 0.217 | <0.001 | 7512 | 1835 |
| **Right pars orbitalis** | 0.067 | 0.030 | [0.008 - 0.126] | 0.481 | 0.026 | 0.217 | <0.001 | 7650 | 1897 |
| **Right inferior parietal cortex** | 0.068 | 0.044 | [-0.018 - 0.154] | 0.384 | 0.123 | 0.280 | 39.357 | 7584 | 1877 |
| **Right lingual gyrus** | 0.068 | 0.030 | [0.009 - 0.127] | 0.394 | 0.023 | 0.217 | <0.001 | 7649 | 1897 |
| **Right posterior cingulate cortex** | 0.069 | 0.050 | [-0.029 - 0.167] | 0.461 | 0.168 | 0.309 | 52.808 | 7648 | 1898 |
| **Left postcentral gyrus** | 0.070 | 0.041 | [-0.01 - 0.151] | 0.388 | 0.086 | 0.257 | 31.476 | 7571 | 1866 |
| **Right middle temporal gyrus** | 0.071 | 0.036 | [0.001 - 0.142] | 0.398 | 0.048 | 0.223 | 17.274 | 7593 | 1867 |
| **Right inferior temporal gyrus** | 0.072 | 0.047 | [-0.021 - 0.165] | 0.391 | 0.128 | 0.280 | 47.090 | 7634 | 1885 |
| **Right supramarginal gyrus** | 0.072 | 0.031 | [0.011 - 0.133] | 0.372 | 0.020 | 0.217 | 2.336 | 7569 | 1855 |
| **Left rostral middle frontal gyrus** | 0.072 | 0.041 | [-0.009 - 0.153] | 0.351 | 0.080 | 0.253 | 32.899 | 7634 | 1897 |
| **Right postcentral gyrus** | 0.074 | 0.046 | [-0.015 - 0.164] | 0.421 | 0.105 | 0.262 | 43.792 | 7596 | 1874 |
| **Left lingual gyrus** | 0.075 | 0.030 | [0.016 - 0.134] | 0.433 | 0.013 | 0.179 | <0.001 | 7640 | 1894 |
| **Left pericalcarine cortex** | 0.075 | 0.030 | [0.016 - 0.134] | 0.529 | 0.012 | 0.179 | <0.001 | 7654 | 1895 |
| **Right pars triangularis** | 0.076 | 0.040 | [-0.002 - 0.153] | 0.436 | 0.057 | 0.234 | 28.223 | 7622 | 1892 |
| **Left middle temporal gyrus** | 0.076 | 0.044 | [-0.011 - 0.163] | 0.450 | 0.088 | 0.257 | 38.212 | 7526 | 1802 |
| **Left caudal anterior cingulate cortex** | 0.077 | 0.056 | [-0.033 - 0.186] | 0.870 | 0.172 | 0.309 | 62.390 | 7590 | 1883 |
| **Left inferior temporal gyrus** | 0.080 | 0.058 | [-0.034 - 0.195] | 0.447 | 0.170 | 0.309 | 64.717 | 7623 | 1869 |
| **Right pericalcarine cortex** | 0.083 | 0.030 | [0.024 - 0.141] | 0.602 | 0.006 | 0.138 | <0.001 | 7650 | 1897 |
| **Left lateral occipital cortex** | 0.083 | 0.030 | [0.024 - 0.142] | 0.477 | 0.006 | 0.138 | <0.001 | 7633 | 1892 |
| **Left hemisphere total surface area** | 0.086 | 0.043 | [0.003 - 0.169] | 0.893 | 0.043 | 0.217 | 36.300 | 7658 | 1902 |
| **Left precentral gyrus** | 0.087 | 0.048 | [-0.007 - 0.18] | 0.496 | 0.069 | 0.241 | 47.666 | 7587 | 1878 |
| **Right cuneus** | 0.097 | 0.053 | [-0.007 - 0.2] | 0.593 | 0.069 | 0.241 | 57.768 | 7634 | 1891 |
| **Left inferior parietal cortex** | 0.117 | 0.038 | [0.042 - 0.192] | 0.664 | 0.002 | 0.138 | 24.261 | 7587 | 1876 |

Dx: Diagnosis.

**Supplementary Table S22**: Full meta-analytic results for surface area of each structure for first episode MDD patients versus Controls comparison controlling for age, sex and scan center. Adjusted Cohen's d is reported.

|  | **Cohen's d ^a^** | **Std. Err.** | **95% CI** | **% Difference** | **P-value** | **FDR P-value** | **I^2^** | **# Controls** | **# Patients** |
| --- | --- | --- | --- | --- | --- | --- | --- | --- | --- |
|  | **(First episode MDD vs CTL)** |  |  |  |  |  |  |  |  |
| **Right transverse temporal gyrus** | -0.085 | 0.060 | [-0.203 - 0.034] | -0.680 | 0.162 | 0.986 | 26.284 | 7251 | 535 |
| **Left pars triangularis** | -0.068 | 0.079 | [-0.223 - 0.086] | -0.416 | 0.386 | 0.986 | 54.161 | 7230 | 534 |
| **Right parahippocampal gyrus** | -0.065 | 0.070 | [-0.202 - 0.073] | -0.705 | 0.357 | 0.986 | 42.460 | 7203 | 529 |
| **Left pericalcarine cortex** | -0.056 | 0.048 | [-0.15 - 0.039] | -0.391 | 0.249 | 0.986 | <0.001 | 7249 | 534 |
| **Right precuneus** | -0.051 | 0.078 | [-0.203 - 0.101] | -0.252 | 0.513 | 0.986 | 52.547 | 7233 | 532 |
| **Right supramarginal gyrus** | -0.050 | 0.094 | [-0.234 - 0.134] | -0.255 | 0.597 | 0.986 | 67.175 | 7185 | 521 |
| **Right pericalcarine cortex** | -0.041 | 0.048 | [-0.136 - 0.054] | -0.299 | 0.395 | 0.986 | <0.001 | 7249 | 534 |
| **Left posterior cingulate cortex** | -0.040 | 0.048 | [-0.135 - 0.055] | -0.250 | 0.409 | 0.986 | <0.001 | 7243 | 533 |
| **Left transverse temporal gyrus** | -0.040 | 0.093 | [-0.223 - 0.144] | -0.311 | 0.672 | 0.986 | 67.471 | 7251 | 534 |
| **Left caudal anterior cingulate cortex** | -0.039 | 0.049 | [-0.134 - 0.056] | -0.447 | 0.417 | 0.986 | <0.001 | 7186 | 529 |
| **Left banks superior temporal sulcus** | -0.038 | 0.059 | [-0.155 - 0.078] | -0.282 | 0.517 | 0.986 | 20.316 | 7115 | 497 |
| **Left lingual gyrus** | -0.036 | 0.049 | [-0.132 - 0.06] | -0.209 | 0.460 | 0.986 | 1.191 | 7236 | 530 |
| **Right lingual gyrus** | -0.035 | 0.048 | [-0.129 - 0.06] | -0.201 | 0.473 | 0.986 | <0.001 | 7247 | 534 |
| **Right superior parietal cortex** | -0.033 | 0.071 | [-0.173 - 0.106] | -0.167 | 0.638 | 0.986 | 43.951 | 7206 | 529 |
| **Left pars opercularis** | -0.033 | 0.086 | [-0.201 - 0.135] | -0.157 | 0.700 | 0.986 | 60.790 | 7217 | 529 |
| **Right pars triangularis** | -0.023 | 0.053 | [-0.127 - 0.081] | -0.134 | 0.661 | 0.986 | 10.546 | 7221 | 530 |
| **Left paracentral lobule** | -0.020 | 0.091 | [-0.197 - 0.158] | -0.112 | 0.829 | 0.986 | 64.572 | 7143 | 517 |
| **Left precuneus** | -0.019 | 0.065 | [-0.146 - 0.108] | -0.090 | 0.771 | 0.986 | 33.901 | 7231 | 533 |
| **Left middle temporal gyrus** | -0.018 | 0.067 | [-0.149 - 0.112] | -0.108 | 0.784 | 0.986 | 34.849 | 7148 | 513 |
| **Left superior parietal cortex** | -0.017 | 0.072 | [-0.158 - 0.124] | -0.084 | 0.811 | 0.986 | 44.716 | 7188 | 528 |
| **Right caudal anterior cingulate cortex** | -0.017 | 0.050 | [-0.115 - 0.081] | -0.190 | 0.733 | 0.986 | 3.349 | 7212 | 528 |
| **Left supramarginal gyrus** | -0.009 | 0.082 | [-0.17 - 0.152] | -0.048 | 0.913 | 0.986 | 56.457 | 7136 | 515 |
| **Left lateral occipital cortex** | -0.009 | 0.048 | [-0.104 - 0.086] | -0.051 | 0.856 | 0.986 | <0.001 | 7230 | 530 |
| **Right medial orbitofrontal cortex** | -0.008 | 0.121 | [-0.245 - 0.229] | -0.067 | 0.947 | 0.986 | 81.015 | 7215 | 529 |
| **Right pars opercularis** | -0.007 | 0.071 | [-0.146 - 0.133] | -0.033 | 0.926 | 0.986 | 43.941 | 7218 | 528 |
| **Right paracentral lobule** | -0.006 | 0.049 | [-0.101 - 0.089] | -0.034 | 0.901 | 0.986 | <0.001 | 7190 | 526 |
| **Right lateral orbitofrontal cortex** | -0.004 | 0.099 | [-0.199 - 0.191] | -0.025 | 0.967 | 0.986 | 71.487 | 7250 | 535 |
| **Right fusiform gyrus** | -0.004 | 0.055 | [-0.111 - 0.103] | -0.020 | 0.940 | 0.986 | 12.624 | 7124 | 517 |
| **Left rostral anterior cingulate cortex** | -1.85E-04 | 0.058 | [-0.115 - 0.114] | -0.002 | 0.997 | 0.997 | 20.711 | 7136 | 518 |
| **Left frontal pole** | 0.002 | 0.048 | [-0.093 - 0.096] | 0.018 | 0.972 | 0.986 | <0.001 | 7252 | 535 |
| **Left cuneus** | 0.002 | 0.054 | [-0.103 - 0.108] | 0.014 | 0.966 | 0.986 | 12.228 | 7225 | 530 |
| **Left isthmus cingulate cortex** | 0.005 | 0.056 | [-0.105 - 0.115] | 0.036 | 0.933 | 0.986 | 17.584 | 7237 | 534 |
| **Left medial orbitofrontal cortex** | 0.006 | 0.083 | [-0.156 - 0.168] | 0.044 | 0.945 | 0.986 | 57.556 | 7168 | 522 |
| **Left caudal middle frontal gyrus** | 0.008 | 0.088 | [-0.165 - 0.181] | 0.042 | 0.926 | 0.986 | 63.071 | 7201 | 528 |
| **Right frontal pole** | 0.011 | 0.067 | [-0.12 - 0.143] | 0.119 | 0.865 | 0.986 | 38.380 | 7249 | 535 |
| **Right insula** | 0.013 | 0.062 | [-0.108 - 0.134] | 0.069 | 0.837 | 0.986 | 28.508 | 7243 | 533 |
| **Right hemisphere total surface area** | 0.014 | 0.082 | [-0.148 - 0.175] | 0.142 | 0.869 | 0.986 | 57.865 | 7253 | 535 |
| **Left inferior parietal cortex** | 0.014 | 0.057 | [-0.098 - 0.126] | 0.080 | 0.806 | 0.986 | 18.216 | 7187 | 524 |
| **Right rostral anterior cingulate cortex** | 0.014 | 0.049 | [-0.081 - 0.109] | 0.143 | 0.771 | 0.986 | <0.001 | 7194 | 526 |
| **Left superior temporal gyrus** | 0.015 | 0.097 | [-0.174 - 0.204] | 0.092 | 0.876 | 0.986 | 68.574 | 7162 | 513 |
| **Right superior temporal gyrus** | 0.018 | 0.102 | [-0.182 - 0.217] | 0.105 | 0.864 | 0.986 | 72.073 | 7195 | 516 |
| **Left hemisphere total surface area** | 0.020 | 0.078 | [-0.133 - 0.174] | 0.213 | 0.794 | 0.986 | 53.511 | 7253 | 535 |
| **Left entorhinal cortex** | 0.021 | 0.049 | [-0.076 - 0.117] | 0.236 | 0.677 | 0.986 | <0.001 | 7079 | 514 |
| **Left lateral orbitofrontal cortex** | 0.021 | 0.082 | [-0.139 - 0.182] | 0.128 | 0.795 | 0.986 | 57.417 | 7250 | 534 |
| **Right posterior cingulate cortex** | 0.022 | 0.083 | [-0.141 - 0.185] | 0.146 | 0.793 | 0.986 | 58.681 | 7243 | 533 |
| **Right temporal pole** | 0.024 | 0.068 | [-0.11 - 0.157] | 0.261 | 0.728 | 0.986 | 39.282 | 7182 | 527 |
| **Right inferior temporal gyrus** | 0.025 | 0.058 | [-0.088 - 0.138] | 0.137 | 0.662 | 0.986 | 20.684 | 7242 | 534 |
| **Right middle temporal gyrus** | 0.026 | 0.089 | [-0.148 - 0.199] | 0.143 | 0.772 | 0.986 | 63.277 | 7197 | 527 |
| **Right precentral gyrus** | 0.026 | 0.075 | [-0.12 - 0.173] | 0.155 | 0.727 | 0.986 | 48.804 | 7188 | 530 |
| **Right cuneus** | 0.030 | 0.048 | [-0.065 - 0.125] | 0.182 | 0.540 | 0.986 | <0.001 | 7232 | 531 |
| **Right banks superior temporal sulcus** | 0.030 | 0.074 | [-0.115 - 0.176] | 0.215 | 0.684 | 0.986 | 47.065 | 7179 | 514 |
| **Right superior frontal gyrus** | 0.033 | 0.090 | [-0.143 - 0.209] | 0.159 | 0.714 | 0.986 | 64.158 | 7184 | 523 |
| **Left insula** | 0.034 | 0.062 | [-0.087 - 0.156] | 0.180 | 0.579 | 0.986 | 29.205 | 7235 | 534 |
| **Right entorhinal cortex** | 0.035 | 0.051 | [-0.064 - 0.134] | 0.415 | 0.491 | 0.986 | 2.243 | 7051 | 506 |
| **Right caudal middle frontal gyrus** | 0.035 | 0.063 | [-0.089 - 0.159] | 0.186 | 0.577 | 0.986 | 31.116 | 7215 | 529 |
| **Left rostral middle frontal gyrus** | 0.037 | 0.068 | [-0.095 - 0.17] | 0.181 | 0.579 | 0.986 | 38.583 | 7232 | 533 |
| **Left postcentral gyrus** | 0.041 | 0.083 | [-0.122 - 0.203] | 0.224 | 0.624 | 0.986 | 57.944 | 7168 | 523 |
| **Right pars orbitalis** | 0.042 | 0.081 | [-0.117 - 0.2] | 0.300 | 0.606 | 0.986 | 56.281 | 7247 | 534 |
| **Right postcentral gyrus** | 0.043 | 0.071 | [-0.096 - 0.183] | 0.246 | 0.541 | 0.986 | 43.768 | 7193 | 531 |
| **Left fusiform gyrus** | 0.047 | 0.056 | [-0.063 - 0.158] | 0.233 | 0.399 | 0.986 | 16.117 | 7107 | 518 |
| **Right isthmus cingulate cortex** | 0.048 | 0.048 | [-0.046 - 0.143] | 0.383 | 0.316 | 0.986 | <0.001 | 7242 | 535 |
| **Left parahippocampal gyrus** | 0.051 | 0.049 | [-0.044 - 0.146] | 0.632 | 0.296 | 0.986 | <0.001 | 7195 | 530 |
| **Right lateral occipital cortex** | 0.053 | 0.068 | [-0.08 - 0.186] | 0.311 | 0.433 | 0.986 | 38.879 | 7237 | 533 |
| **Right inferior parietal cortex** | 0.060 | 0.069 | [-0.076 - 0.196] | 0.342 | 0.384 | 0.986 | 40.883 | 7182 | 526 |
| **Left pars orbitalis** | 0.060 | 0.069 | [-0.074 - 0.195] | 0.441 | 0.380 | 0.986 | 40.674 | 7243 | 535 |
| **Right rostral middle frontal gyrus** | 0.061 | 0.052 | [-0.041 - 0.163] | 0.292 | 0.245 | 0.986 | 8.219 | 7231 | 530 |
| **Left precentral gyrus** | 0.066 | 0.071 | [-0.074 - 0.206] | 0.380 | 0.354 | 0.986 | 44.115 | 7188 | 525 |
| **Left superior frontal gyrus** | 0.070 | 0.061 | [-0.05 - 0.189] | 0.352 | 0.253 | 0.986 | 26.412 | 7150 | 526 |
| **Left inferior temporal gyrus** | 0.073 | 0.065 | [-0.055 - 0.2] | 0.407 | 0.263 | 0.986 | 34.336 | 7241 | 532 |
| **Left temporal pole** | 0.090 | 0.048 | [-0.005 - 0.185] | 0.907 | 0.064 | 0.986 | <0.001 | 7232 | 530 |

**^a^** Included Samples: CLING, Imaging Genetics Dublin, Clinical Depression Dublin, Houston, MMDP 3T, MPIP, Muenster Cohort, NESDA, DepOx, SHIP, SHIP-trend, Sydney, QTIM, Rotterdam study.

MDD: Major Depressive Disorder; CTL: Controls.

**Supplementary Table S23**: Full meta-analytic results for surface area of each structure for recurrent episode MDD patients versus Controls comparison controlling for age, sex and scan center. Adjusted Cohen's d is reported.

|  | **Cohen's d ^a^** | **Std. Err.** | **95% CI** | **% Difference** | **P-value** | **FDR P-value** | **I^2^** | **# Controls** | **# Patients** |
| --- | --- | --- | --- | --- | --- | --- | --- | --- | --- |
|  | **(Recurrent MDD vs CTL)** |  |  |  |  |  |  |  |  |
| **Left frontal pole** | -0.058 | 0.035 | [-0.126 - 0.011] | -0.609 | 0.098 | 0.516 | <0.001 | 7449 | 1300 |
| **Left pars triangularis** | -0.047 | 0.035 | [-0.115 - 0.022] | -0.284 | 0.182 | 0.631 | <0.001 | 7426 | 1295 |
| **Left banks superior temporal sulcus** | -0.043 | 0.056 | [-0.153 - 0.067] | -0.315 | 0.444 | 0.797 | 46.656 | 7292 | 1189 |
| **Right parahippocampal gyrus** | -0.036 | 0.035 | [-0.105 - 0.033] | -0.391 | 0.307 | 0.716 | <0.001 | 7398 | 1287 |
| **Right fusiform gyrus** | -0.035 | 0.038 | [-0.109 - 0.04] | -0.169 | 0.363 | 0.725 | 7.016 | 7316 | 1278 |
| **Left parahippocampal gyrus** | -0.024 | 0.053 | [-0.129 - 0.08] | -0.305 | 0.647 | 0.888 | 44.457 | 7385 | 1284 |
| **Right transverse temporal gyrus** | -0.010 | 0.059 | [-0.125 - 0.105] | -0.078 | 0.868 | 0.951 | 54.272 | 7448 | 1301 |
| **Right insula** | -0.009 | 0.058 | [-0.123 - 0.106] | -0.047 | 0.883 | 0.951 | 53.288 | 7433 | 1293 |
| **Left entorhinal cortex** | -0.009 | 0.058 | [-0.122 - 0.105] | -0.098 | 0.883 | 0.951 | 51.902 | 7237 | 1249 |
| **Left pericalcarine cortex** | -0.008 | 0.057 | [-0.121 - 0.104] | -0.059 | 0.884 | 0.951 | 51.728 | 7446 | 1296 |
| **Right caudal middle frontal gyrus** | -0.007 | 0.035 | [-0.075 - 0.062] | -0.034 | 0.852 | 0.951 | <0.001 | 7412 | 1295 |
| **Left inferior parietal cortex** | -0.004 | 0.035 | [-0.073 - 0.065] | -0.023 | 0.910 | 0.951 | <0.001 | 7379 | 1287 |
| **Right pars triangularis** | -0.003 | 0.045 | [-0.092 - 0.086] | -0.017 | 0.949 | 0.977 | 26.851 | 7414 | 1297 |
| **Right inferior parietal cortex** | -0.002 | 0.045 | [-0.09 - 0.086] | -0.010 | 0.967 | 0.981 | 25.759 | 7376 | 1286 |
| **Left pars opercularis** | -0.001 | 0.035 | [-0.069 - 0.068] | -0.004 | 0.982 | 0.982 | <0.001 | 7412 | 1295 |
| **Right banks superior temporal sulcus** | 0.004 | 0.036 | [-0.066 - 0.074] | 0.030 | 0.907 | 0.951 | <0.001 | 7364 | 1238 |
| **Right pars opercularis** | 0.005 | 0.035 | [-0.064 - 0.073] | 0.024 | 0.893 | 0.951 | <0.001 | 7411 | 1293 |
| **Right superior parietal cortex** | 0.006 | 0.036 | [-0.064 - 0.077] | 0.031 | 0.861 | 0.951 | 1.840 | 7402 | 1279 |
| **Left fusiform gyrus** | 0.007 | 0.035 | [-0.062 - 0.077] | 0.035 | 0.838 | 0.951 | <0.001 | 7296 | 1279 |
| **Left cuneus** | 0.009 | 0.035 | [-0.059 - 0.078] | 0.057 | 0.792 | 0.951 | <0.001 | 7418 | 1296 |
| **Right supramarginal gyrus** | 0.010 | 0.045 | [-0.079 - 0.099] | 0.050 | 0.830 | 0.951 | 26.053 | 7369 | 1273 |
| **Left pars orbitalis** | 0.010 | 0.052 | [-0.093 - 0.113] | 0.075 | 0.846 | 0.951 | 43.113 | 7439 | 1299 |
| **Left middle temporal gyrus** | 0.015 | 0.039 | [-0.061 - 0.091] | 0.091 | 0.695 | 0.909 | 7.578 | 7322 | 1225 |
| **Left posterior cingulate cortex** | 0.018 | 0.047 | [-0.074 - 0.11] | 0.113 | 0.701 | 0.909 | 30.639 | 7437 | 1298 |
| **Right inferior temporal gyrus** | 0.019 | 0.035 | [-0.05 - 0.087] | 0.101 | 0.596 | 0.852 | <0.001 | 7427 | 1287 |
| **Left postcentral gyrus** | 0.019 | 0.035 | [-0.051 - 0.088] | 0.103 | 0.596 | 0.852 | <0.001 | 7364 | 1278 |
| **Left precuneus** | 0.020 | 0.050 | [-0.079 - 0.119] | 0.095 | 0.693 | 0.909 | 38.949 | 7428 | 1294 |
| **Left transverse temporal gyrus** | 0.022 | 0.035 | [-0.046 - 0.091] | 0.177 | 0.520 | 0.810 | <0.001 | 7446 | 1300 |
| **Right postcentral gyrus** | 0.023 | 0.035 | [-0.046 - 0.092] | 0.129 | 0.519 | 0.810 | <0.001 | 7390 | 1278 |
| **Right pericalcarine cortex** | 0.025 | 0.035 | [-0.044 - 0.093] | 0.182 | 0.475 | 0.809 | <0.001 | 7442 | 1299 |
| **Right entorhinal cortex** | 0.025 | 0.036 | [-0.046 - 0.096] | 0.299 | 0.485 | 0.809 | <0.001 | 7207 | 1230 |
| **Left insula** | 0.026 | 0.043 | [-0.058 - 0.111] | 0.136 | 0.545 | 0.829 | 21.264 | 7426 | 1297 |
| **Right precentral gyrus** | 0.028 | 0.059 | [-0.087 - 0.143] | 0.166 | 0.635 | 0.888 | 54.004 | 7383 | 1285 |
| **Right frontal pole** | 0.031 | 0.053 | [-0.073 - 0.135] | 0.322 | 0.562 | 0.837 | 44.587 | 7444 | 1300 |
| **Right caudal anterior cingulate cortex** | 0.031 | 0.035 | [-0.038 - 0.1] | 0.346 | 0.375 | 0.730 | <0.001 | 7407 | 1290 |
| **Right precuneus** | 0.033 | 0.035 | [-0.035 - 0.102] | 0.166 | 0.339 | 0.719 | <0.001 | 7427 | 1297 |
| **Right medial orbitofrontal cortex** | 0.034 | 0.035 | [-0.035 - 0.103] | 0.284 | 0.336 | 0.719 | <0.001 | 7408 | 1290 |
| **Left superior parietal cortex** | 0.034 | 0.035 | [-0.035 - 0.103] | 0.168 | 0.330 | 0.719 | <0.001 | 7384 | 1285 |
| **Right superior frontal gyrus** | 0.036 | 0.035 | [-0.033 - 0.105] | 0.176 | 0.303 | 0.716 | <0.001 | 7381 | 1289 |
| **Right posterior cingulate cortex** | 0.036 | 0.050 | [-0.061 - 0.134] | 0.244 | 0.463 | 0.809 | 37.390 | 7440 | 1300 |
| **Right isthmus cingulate cortex** | 0.036 | 0.057 | [-0.075 - 0.148] | 0.289 | 0.521 | 0.810 | 51.039 | 7434 | 1297 |
| **Right superior temporal gyrus** | 0.039 | 0.036 | [-0.031 - 0.109] | 0.233 | 0.276 | 0.689 | <0.001 | 7348 | 1236 |
| **Right hemisphere total surface area** | 0.040 | 0.035 | [-0.029 - 0.108] | 0.416 | 0.253 | 0.681 | <0.001 | 7450 | 1302 |
| **Left superior temporal gyrus** | 0.041 | 0.036 | [-0.029 - 0.112] | 0.252 | 0.252 | 0.681 | <0.001 | 7316 | 1215 |
| **Left caudal anterior cingulate cortex** | 0.044 | 0.035 | [-0.025 - 0.113] | 0.498 | 0.212 | 0.645 | <0.001 | 7382 | 1289 |
| **Right middle temporal gyrus** | 0.045 | 0.035 | [-0.024 - 0.114] | 0.253 | 0.198 | 0.631 | <0.001 | 7385 | 1277 |
| **Left rostral middle frontal gyrus** | 0.046 | 0.035 | [-0.023 - 0.114] | 0.221 | 0.192 | 0.631 | <0.001 | 7427 | 1299 |
| **Right paracentral lobule** | 0.046 | 0.035 | [-0.023 - 0.115] | 0.262 | 0.189 | 0.631 | <0.001 | 7385 | 1290 |
| **Right lingual gyrus** | 0.047 | 0.035 | [-0.021 - 0.116] | 0.274 | 0.176 | 0.631 | <0.001 | 7441 | 1298 |
| **Right lateral orbitofrontal cortex** | 0.048 | 0.052 | [-0.054 - 0.151] | 0.284 | 0.355 | 0.725 | 42.745 | 7446 | 1302 |
| **Right rostral anterior cingulate cortex** | 0.050 | 0.035 | [-0.019 - 0.119] | 0.508 | 0.154 | 0.631 | <0.001 | 7387 | 1286 |
| **Left caudal middle frontal gyrus** | 0.053 | 0.044 | [-0.033 - 0.14] | 0.272 | 0.227 | 0.661 | 23.837 | 7398 | 1292 |
| **Right pars orbitalis** | 0.056 | 0.035 | [-0.013 - 0.124] | 0.402 | 0.111 | 0.516 | <0.001 | 7442 | 1299 |
| **Left lingual gyrus** | 0.058 | 0.072 | [-0.082 - 0.199] | 0.337 | 0.416 | 0.787 | 69.734 | 7432 | 1299 |
| **Left lateral orbitofrontal cortex** | 0.059 | 0.035 | [-0.009 - 0.128] | 0.359 | 0.089 | 0.516 | <0.001 | 7447 | 1302 |
| **Left hemisphere total surface area** | 0.060 | 0.035 | [-0.009 - 0.128] | 0.622 | 0.087 | 0.516 | <0.001 | 7450 | 1302 |
| **Left rostral anterior cingulate cortex** | 0.060 | 0.035 | [-0.009 - 0.129] | 0.584 | 0.089 | 0.516 | <0.001 | 7332 | 1279 |
| **Left superior frontal gyrus** | 0.060 | 0.035 | [-0.008 - 0.129] | 0.306 | 0.086 | 0.516 | <0.001 | 7347 | 1288 |
| **Left precentral gyrus** | 0.061 | 0.078 | [-0.092 - 0.213] | 0.348 | 0.437 | 0.797 | 74.453 | 7381 | 1288 |
| **Left temporal pole** | 0.061 | 0.035 | [-0.008 - 0.13] | 0.614 | 0.084 | 0.516 | <0.001 | 7415 | 1276 |
| **Left inferior temporal gyrus** | 0.064 | 0.039 | [-0.011 - 0.14] | 0.360 | 0.096 | 0.516 | 8.832 | 7415 | 1274 |
| **Right cuneus** | 0.066 | 0.035 | [-0.003 - 0.134] | 0.403 | 0.061 | 0.516 | <0.001 | 7426 | 1296 |
| **Left paracentral lobule** | 0.069 | 0.043 | [-0.016 - 0.154] | 0.394 | 0.111 | 0.516 | 21.568 | 7340 | 1283 |
| **Right rostral middle frontal gyrus** | 0.074 | 0.035 | [0.005 - 0.142] | 0.356 | 0.035 | 0.516 | <0.001 | 7428 | 1295 |
| **Left supramarginal gyrus** | 0.075 | 0.051 | [-0.025 - 0.175] | 0.401 | 0.144 | 0.630 | 38.090 | 7308 | 1257 |
| **Left medial orbitofrontal cortex** | 0.081 | 0.073 | [-0.061 - 0.223] | 0.628 | 0.264 | 0.684 | 70.002 | 7356 | 1284 |
| **Left lateral occipital cortex** | 0.085 | 0.035 | [0.017 - 0.154] | 0.491 | 0.015 | 0.516 | <0.001 | 7425 | 1297 |
| **Right temporal pole** | 0.094 | 0.042 | [0.011 - 0.177] | 1.030 | 0.027 | 0.516 | 18.209 | 7366 | 1268 |
| **Right lateral occipital cortex** | 0.108 | 0.035 | [0.039 - 0.176] | 0.629 | 0.002 | 0.147 | <0.001 | 7434 | 1299 |
| **Left isthmus cingulate cortex** | 0.108 | 0.052 | [0.006 - 0.21] | 0.832 | 0.038 | 0.516 | 42.251 | 7434 | 1294 |

**^a^** Included Samples: CODE, CLING, Imaging Genetics Dublin, Clinical Depression Dublin, Houston, Sexpect, MMDP 3T, Melbourne, MPIP, Muenster Cohort, NESDA, Novosibirsk, DepOx, SHIP, SHIP-trend, Sydney, Stanford, Rotterdam study.

MDD: Major Depressive Disorder; CTL: Controls.

**Supplementary Table S24**: Full meta-analytic results for surface area of each structure for first episode MDD versus recurrent episode MDD patients comparison controlling for age, sex and scan center. Adjusted Cohen's d is reported.

|  | **Cohen's d ^a^** | **Std. Err.** | **95% CI** | **% Difference** | **P-value** | **FDR P-value** | **I^2^** | **# First episode** | **# Recurrent episode** |
| --- | --- | --- | --- | --- | --- | --- | --- | --- | --- |
|  | **(First episode MDD vs Recurrent MDD)** |  |  |  |  |  |  |  |  |
| **Left parahippocampal gyrus** | -0.116 | 0.078 | [-0.269 - 0.037] | -1.443 | 0.137 | 0.987 | 41.006 | 530 | 1097 |
| **Left frontal pole** | -0.114 | 0.091 | [-0.292 - 0.064] | -1.198 | 0.210 | 0.987 | 55.976 | 535 | 1110 |
| **Right inferior parietal cortex** | -0.084 | 0.083 | [-0.246 - 0.079] | -0.474 | 0.314 | 0.987 | 47.165 | 526 | 1095 |
| **Right frontal pole** | -0.069 | 0.102 | [-0.27 - 0.131] | -0.721 | 0.499 | 0.987 | 65.553 | 535 | 1109 |
| **Left inferior parietal cortex** | -0.064 | 0.095 | [-0.251 - 0.122] | -0.366 | 0.498 | 0.987 | 59.280 | 524 | 1100 |
| **Right caudal middle frontal gyrus** | -0.057 | 0.054 | [-0.164 - 0.05] | -0.301 | 0.295 | 0.987 | <0.001 | 529 | 1105 |
| **Right fusiform gyrus** | -0.046 | 0.055 | [-0.154 - 0.062] | -0.225 | 0.401 | 0.987 | <0.001 | 517 | 1091 |
| **Left cuneus** | -0.045 | 0.097 | [-0.234 - 0.145] | -0.276 | 0.644 | 0.987 | 61.100 | 530 | 1107 |
| **Right isthmus cingulate cortex** | -0.043 | 0.078 | [-0.196 - 0.11] | -0.339 | 0.584 | 0.987 | 41.491 | 535 | 1108 |
| **Right postcentral gyrus** | -0.038 | 0.098 | [-0.231 - 0.154] | -0.218 | 0.694 | 0.987 | 61.955 | 531 | 1088 |
| **Left fusiform gyrus** | -0.036 | 0.063 | [-0.16 - 0.087] | -0.178 | 0.565 | 0.987 | 15.884 | 518 | 1092 |
| **Right insula** | -0.032 | 0.054 | [-0.139 - 0.074] | -0.175 | 0.555 | 0.987 | <0.001 | 533 | 1107 |
| **Right rostral middle frontal gyrus** | -0.029 | 0.091 | [-0.208 - 0.15] | -0.138 | 0.753 | 0.987 | 56.375 | 530 | 1104 |
| **Right banks superior temporal sulcus** | -0.027 | 0.055 | [-0.136 - 0.081] | -0.196 | 0.620 | 0.987 | <0.001 | 514 | 1056 |
| **Right parahippocampal gyrus** | -0.018 | 0.055 | [-0.125 - 0.089] | -0.200 | 0.737 | 0.987 | <0.001 | 529 | 1100 |
| **Left entorhinal cortex** | -0.016 | 0.069 | [-0.15 - 0.119] | -0.180 | 0.821 | 0.987 | 26.064 | 514 | 1085 |
| **Right posterior cingulate cortex** | -0.013 | 0.090 | [-0.19 - 0.164] | -0.088 | 0.884 | 0.987 | 55.469 | 533 | 1111 |
| **Left pericalcarine cortex** | -0.012 | 0.078 | [-0.166 - 0.141] | -0.087 | 0.874 | 0.987 | 41.683 | 534 | 1110 |
| **Left inferior temporal gyrus** | -0.011 | 0.054 | [-0.118 - 0.096] | -0.061 | 0.840 | 0.987 | <0.001 | 532 | 1108 |
| **Left rostral middle frontal gyrus** | -0.010 | 0.086 | [-0.179 - 0.158] | -0.051 | 0.904 | 0.987 | 51.226 | 533 | 1108 |
| **Right cuneus** | -0.008 | 0.081 | [-0.168 - 0.151] | -0.051 | 0.918 | 0.987 | 45.537 | 531 | 1110 |
| **Right lateral orbitofrontal cortex** | -0.008 | 0.054 | [-0.114 - 0.098] | -0.048 | 0.881 | 0.987 | <0.001 | 535 | 1111 |
| **Left insula** | -0.006 | 0.054 | [-0.112 - 0.101] | -0.029 | 0.917 | 0.987 | <0.001 | 534 | 1110 |
| **Right superior temporal gyrus** | -0.005 | 0.093 | [-0.187 - 0.178] | -0.028 | 0.960 | 0.987 | 57.311 | 516 | 1084 |
| **Right middle temporal gyrus** | -0.002 | 0.109 | [-0.215 - 0.211] | -0.010 | 0.987 | 0.987 | 69.253 | 527 | 1092 |
| **Right hemisphere total surface area** | 0.001 | 0.079 | [-0.153 - 0.156] | 0.013 | 0.987 | 0.987 | 42.724 | 535 | 1111 |
| **Right entorhinal cortex** | 0.004 | 0.070 | [-0.134 - 0.142] | 0.051 | 0.952 | 0.987 | 28.204 | 506 | 1071 |
| **Left pars triangularis** | 0.005 | 0.084 | [-0.161 - 0.17] | 0.028 | 0.956 | 0.987 | 49.259 | 534 | 1107 |
| **Left temporal pole** | 0.005 | 0.054 | [-0.102 - 0.111] | 0.048 | 0.930 | 0.987 | <0.001 | 530 | 1109 |
| **Right superior frontal gyrus** | 0.005 | 0.087 | [-0.166 - 0.176] | 0.024 | 0.954 | 0.987 | 52.157 | 523 | 1099 |
| **Left lingual gyrus** | 0.007 | 0.082 | [-0.153 - 0.167] | 0.039 | 0.934 | 0.987 | 45.750 | 530 | 1109 |
| **Right medial orbitofrontal cortex** | 0.007 | 0.118 | [-0.225 - 0.239] | 0.062 | 0.951 | 0.987 | 74.419 | 529 | 1100 |
| **Right pars orbitalis** | 0.011 | 0.102 | [-0.189 - 0.211] | 0.078 | 0.916 | 0.987 | 65.320 | 534 | 1110 |
| **Left pars orbitalis** | 0.012 | 0.054 | [-0.094 - 0.118] | 0.090 | 0.821 | 0.987 | <0.001 | 535 | 1110 |
| **Left lateral orbitofrontal cortex** | 0.012 | 0.075 | [-0.135 - 0.16] | 0.074 | 0.871 | 0.987 | 37.798 | 534 | 1111 |
| **Right pars opercularis** | 0.013 | 0.054 | [-0.093 - 0.12] | 0.067 | 0.807 | 0.987 | <0.001 | 528 | 1105 |
| **Left superior temporal gyrus** | 0.017 | 0.089 | [-0.159 - 0.192] | 0.102 | 0.853 | 0.987 | 53.450 | 513 | 1070 |
| **Left superior frontal gyrus** | 0.017 | 0.060 | [-0.1 - 0.134] | 0.086 | 0.775 | 0.987 | 9.929 | 526 | 1097 |
| **Right caudal anterior cingulate cortex** | 0.019 | 0.085 | [-0.148 - 0.185] | 0.207 | 0.827 | 0.987 | 49.794 | 528 | 1103 |
| **Left banks superior temporal sulcus** | 0.020 | 0.056 | [-0.091 - 0.13] | 0.144 | 0.727 | 0.987 | <0.001 | 497 | 1030 |
| **Left middle temporal gyrus** | 0.021 | 0.075 | [-0.126 - 0.169] | 0.125 | 0.779 | 0.987 | 35.198 | 513 | 1062 |
| **Right pericalcarine cortex** | 0.022 | 0.054 | [-0.084 - 0.128] | 0.160 | 0.686 | 0.987 | <0.001 | 534 | 1111 |
| **Right pars triangularis** | 0.026 | 0.079 | [-0.128 - 0.18] | 0.150 | 0.741 | 0.987 | 41.886 | 530 | 1108 |
| **Left postcentral gyrus** | 0.027 | 0.064 | [-0.097 - 0.152] | 0.151 | 0.667 | 0.987 | 17.012 | 523 | 1090 |
| **Right inferior temporal gyrus** | 0.028 | 0.054 | [-0.079 - 0.134] | 0.149 | 0.612 | 0.987 | <0.001 | 534 | 1110 |
| **Right supramarginal gyrus** | 0.028 | 0.092 | [-0.152 - 0.209] | 0.146 | 0.758 | 0.987 | 56.422 | 521 | 1091 |
| **Left posterior cingulate cortex** | 0.029 | 0.091 | [-0.15 - 0.207] | 0.181 | 0.751 | 0.987 | 56.250 | 533 | 1108 |
| **Left transverse temporal gyrus** | 0.033 | 0.103 | [-0.169 - 0.235] | 0.259 | 0.749 | 0.987 | 65.868 | 534 | 1110 |
| **Left hemisphere total surface area** | 0.034 | 0.073 | [-0.11 - 0.178] | 0.353 | 0.643 | 0.987 | 34.870 | 535 | 1111 |
| **Left precentral gyrus** | 0.037 | 0.069 | [-0.097 - 0.172] | 0.213 | 0.589 | 0.987 | 26.491 | 525 | 1101 |
| **Left supramarginal gyrus** | 0.037 | 0.098 | [-0.155 - 0.23] | 0.200 | 0.704 | 0.987 | 61.629 | 515 | 1085 |
| **Right rostral anterior cingulate cortex** | 0.037 | 0.080 | [-0.119 - 0.194] | 0.379 | 0.639 | 0.987 | 43.034 | 526 | 1098 |
| **Left pars opercularis** | 0.038 | 0.079 | [-0.116 - 0.193] | 0.183 | 0.626 | 0.987 | 41.902 | 529 | 1106 |
| **Right superior parietal cortex** | 0.043 | 0.055 | [-0.064 - 0.15] | 0.213 | 0.435 | 0.987 | <0.001 | 529 | 1095 |
| **Left precuneus** | 0.044 | 0.054 | [-0.063 - 0.15] | 0.208 | 0.422 | 0.987 | <0.001 | 533 | 1107 |
| **Right precentral gyrus** | 0.049 | 0.055 | [-0.058 - 0.156] | 0.289 | 0.372 | 0.987 | <0.001 | 530 | 1099 |
| **Right transverse temporal gyrus** | 0.050 | 0.098 | [-0.142 - 0.241] | 0.401 | 0.610 | 0.987 | 62.112 | 535 | 1111 |
| **Left medial orbitofrontal cortex** | 0.051 | 0.062 | [-0.071 - 0.172] | 0.393 | 0.413 | 0.987 | 13.902 | 522 | 1095 |
| **Right lateral occipital cortex** | 0.054 | 0.086 | [-0.116 - 0.223] | 0.316 | 0.533 | 0.987 | 51.625 | 533 | 1110 |
| **Right lingual gyrus** | 0.055 | 0.054 | [-0.052 - 0.161] | 0.318 | 0.312 | 0.987 | <0.001 | 534 | 1109 |
| **Right temporal pole** | 0.056 | 0.055 | [-0.051 - 0.163] | 0.611 | 0.309 | 0.987 | <0.001 | 527 | 1100 |
| **Left superior parietal cortex** | 0.059 | 0.071 | [-0.079 - 0.198] | 0.289 | 0.403 | 0.987 | 29.821 | 528 | 1096 |
| **Right precuneus** | 0.060 | 0.054 | [-0.046 - 0.167] | 0.301 | 0.265 | 0.987 | <0.001 | 532 | 1110 |
| **Left lateral occipital cortex** | 0.061 | 0.075 | [-0.086 - 0.208] | 0.349 | 0.419 | 0.987 | 37.097 | 530 | 1109 |
| **Left caudal middle frontal gyrus** | 0.066 | 0.080 | [-0.092 - 0.223] | 0.334 | 0.414 | 0.987 | 43.969 | 528 | 1101 |
| **Left rostral anterior cingulate cortex** | 0.071 | 0.055 | [-0.036 - 0.179] | 0.696 | 0.194 | 0.987 | <0.001 | 518 | 1091 |
| **Right paracentral lobule** | 0.075 | 0.055 | [-0.032 - 0.182] | 0.425 | 0.170 | 0.987 | <0.001 | 526 | 1099 |
| **Left caudal anterior cingulate cortex** | 0.078 | 0.055 | [-0.028 - 0.185] | 0.891 | 0.150 | 0.987 | <0.001 | 529 | 1099 |
| **Left isthmus cingulate cortex** | 0.142 | 0.054 | [0.035 - 0.248] | 1.089 | 0.009 | 0.642 | <0.001 | 534 | 1107 |
| **Left paracentral lobule** | 0.148 | 0.063 | [0.025 - 0.271] | 0.840 | 0.019 | 0.655 | 15.203 | 517 | 1093 |

**^a^** Included Samples: CLING, Imaging Genetics Dublin, Clinical Depression Dublin, Houston, MMDP 3T, MPIP, Muenster Cohort, NESDA, DepOx, SHIP, SHIP-trend, Sydney, Rotterdam study.

MDD: Major Depressive Disorder.

**Supplementary Table S25**: Full meta-analytic results for thickness of each structure for the association with number of episodes in recurrent episode MDD patients controlling for age, sex and scan center. Adjusted Cohen's d is reported.

|  | **Pearson's r ^a^** | **Std. Err.** | **95% CI** | **% Difference** | **P-value** | **FDR P-value** | **I^2^** | **# Patients** |
| --- | --- | --- | --- | --- | --- | --- | --- | --- |
|  | **(#Episodes in Recurrent MDD)** |  |  |  |  |  |  |  |
| **Left isthmus cingulate cortex** | -0.078 | 0.095 | [-0.264 - 0.107] | -2.648 | 0.410 | 0.879 | 73.921 | 494 |
| **Right entorhinal cortex** | -0.063 | 0.046 | [-0.153 - 0.027] | -2.765 | 0.173 | 0.879 | <0.001 | 472 |
| **Right pars opercularis** | -0.053 | 0.045 | [-0.141 - 0.034] | -1.717 | 0.230 | 0.879 | <0.001 | 495 |
| **Right temporal pole** | -0.036 | 0.117 | [-0.265 - 0.193] | -1.167 | 0.759 | 0.948 | 83.692 | 483 |
| **Right precentral gyrus** | -0.036 | 0.045 | [-0.123 - 0.052] | -0.816 | 0.421 | 0.879 | <0.001 | 495 |
| **Left rostral middle frontal gyrus** | -0.035 | 0.045 | [-0.123 - 0.052] | -0.995 | 0.430 | 0.879 | <0.001 | 496 |
| **Right medial orbitofrontal cortex** | -0.033 | 0.080 | [-0.191 - 0.125] | -1.019 | 0.681 | 0.948 | 61.063 | 495 |
| **Right pars orbitalis** | -0.032 | 0.068 | [-0.165 - 0.101] | -0.954 | 0.641 | 0.948 | 44.260 | 495 |
| **Left transverse temporal gyrus** | -0.025 | 0.094 | [-0.209 - 0.16] | -0.820 | 0.792 | 0.951 | 72.694 | 496 |
| **Right banks superior temporal sulcus** | -0.018 | 0.045 | [-0.106 - 0.07] | -0.565 | 0.689 | 0.948 | <0.001 | 489 |
| **Left pars triangularis** | -0.018 | 0.045 | [-0.106 - 0.071] | -0.542 | 0.696 | 0.948 | <0.001 | 494 |
| **Left superior parietal cortex** | -0.014 | 0.066 | [-0.144 - 0.115] | -0.343 | 0.829 | 0.951 | 42.156 | 496 |
| **Right inferior temporal gyrus** | -0.014 | 0.075 | [-0.161 - 0.133] | -0.477 | 0.851 | 0.961 | 53.274 | 487 |
| **Left precentral gyrus** | -0.002 | 0.045 | [-0.091 - 0.086] | -0.050 | 0.960 | 0.988 | <0.001 | 496 |
| **Right isthmus cingulate cortex** | 1.74E-04 | 0.076 | [-0.148 - 0.148] | 0.006 | 0.998 | 0.998 | 55.634 | 496 |
| **Left paracentral gyrus** | 0.001 | 0.053 | [-0.102 - 0.104] | 0.031 | 0.983 | 0.997 | 16.641 | 496 |
| **Right posterior cingulate cortex** | 0.003 | 0.044 | [-0.084 - 0.09] | 0.093 | 0.946 | 0.988 | <0.001 | 496 |
| **Right superior parietal cortex** | 0.006 | 0.065 | [-0.121 - 0.133] | 0.132 | 0.931 | 0.987 | 39.146 | 492 |
| **Right pericalcarine cortex** | 0.007 | 0.045 | [-0.081 - 0.094] | 0.220 | 0.883 | 0.981 | <0.001 | 493 |
| **Right rostral middle frontal gyrus** | 0.008 | 0.090 | [-0.169 - 0.185] | 0.229 | 0.928 | 0.987 | 69.892 | 496 |
| **Right paracentral gyrus** | 0.009 | 0.089 | [-0.165 - 0.183] | 0.252 | 0.919 | 0.987 | 69.053 | 496 |
| **Left lingual gyrus** | 0.010 | 0.045 | [-0.078 - 0.098] | 0.290 | 0.818 | 0.951 | <0.001 | 496 |
| **Left pericalcarine cortex** | 0.012 | 0.057 | [-0.099 - 0.124] | 0.425 | 0.829 | 0.951 | 24.074 | 491 |
| **Left postcentral gyrus** | 0.015 | 0.045 | [-0.073 - 0.103] | 0.347 | 0.740 | 0.948 | <0.001 | 494 |
| **Left medial orbitofrontal cortex** | 0.016 | 0.045 | [-0.072 - 0.105] | 0.508 | 0.719 | 0.948 | <0.001 | 494 |
| **Left entorhinal cortex** | 0.020 | 0.046 | [-0.07 - 0.11] | 0.879 | 0.667 | 0.948 | <0.001 | 475 |
| **Right rostral anterior cingulate cortex** | 0.027 | 0.045 | [-0.062 - 0.115] | 1.212 | 0.555 | 0.925 | <0.001 | 494 |
| **Left precuneus** | 0.029 | 0.081 | [-0.129 - 0.188] | 0.751 | 0.715 | 0.948 | 61.283 | 495 |
| **Right insula** | 0.031 | 0.114 | [-0.193 - 0.255] | 0.853 | 0.785 | 0.951 | 82.717 | 493 |
| **Right pars triangularis** | 0.031 | 0.046 | [-0.058 - 0.121] | 1.019 | 0.493 | 0.879 | 3.185 | 495 |
| **Left superior temporal gyrus** | 0.032 | 0.100 | [-0.165 - 0.228] | 0.789 | 0.752 | 0.948 | 73.566 | 465 |
| **Right lateral occipital cortex** | 0.035 | 0.045 | [-0.053 - 0.123] | 0.925 | 0.437 | 0.879 | <0.001 | 496 |
| **Right superior temporal gyrus** | 0.037 | 0.096 | [-0.152 - 0.225] | 0.868 | 0.701 | 0.948 | 71.275 | 473 |
| **Left posterior cingulate cortex** | 0.039 | 0.079 | [-0.115 - 0.194] | 1.164 | 0.616 | 0.948 | 59.567 | 496 |
| **Right caudal anterior cingulate cortex** | 0.040 | 0.061 | [-0.079 - 0.159] | 1.551 | 0.511 | 0.879 | 32.267 | 494 |
| **Left frontal pole** | 0.042 | 0.045 | [-0.046 - 0.13] | 1.305 | 0.352 | 0.879 | <0.001 | 496 |
| **Left pars orbitalis** | 0.043 | 0.045 | [-0.045 - 0.131] | 1.296 | 0.339 | 0.879 | <0.001 | 496 |
| **Left rostral anterior cingulate cortex** | 0.044 | 0.093 | [-0.139 - 0.227] | 1.946 | 0.636 | 0.948 | 72.224 | 493 |
| **Right parahippocampal gyrus** | 0.050 | 0.055 | [-0.058 - 0.157] | 1.626 | 0.367 | 0.879 | 21.408 | 493 |
| **Right superior frontal gyrus** | 0.051 | 0.094 | [-0.134 - 0.235] | 1.301 | 0.590 | 0.948 | 73.003 | 496 |
| **Left hemisphere total surface area** | 0.052 | 0.074 | [-0.093 - 0.198] | 1.089 | 0.481 | 0.879 | 53.855 | 496 |
| **Left supramarginal gyrus** | 0.054 | 0.084 | [-0.109 - 0.218] | 1.591 | 0.515 | 0.879 | 62.541 | 483 |
| **Right inferior parietal cortex** | 0.055 | 0.053 | [-0.05 - 0.159] | 1.513 | 0.305 | 0.879 | 17.481 | 496 |
| **Right supramarginal gyrus** | 0.055 | 0.045 | [-0.033 - 0.143] | 1.563 | 0.224 | 0.879 | <0.001 | 492 |
| **Right hemisphere total surface area** | 0.055 | 0.076 | [-0.094 - 0.204] | 1.150 | 0.469 | 0.879 | 56.263 | 496 |
| **Right lingual gyrus** | 0.057 | 0.045 | [-0.031 - 0.144] | 1.577 | 0.205 | 0.879 | <0.001 | 494 |
| **Right transverse temporal gyrus** | 0.057 | 0.068 | [-0.075 - 0.19] | 1.899 | 0.397 | 0.879 | 44.880 | 496 |
| **Left superior frontal gyrus** | 0.060 | 0.083 | [-0.103 - 0.224] | 1.484 | 0.468 | 0.879 | 64.593 | 496 |
| **Right precuneus** | 0.061 | 0.055 | [-0.047 - 0.168] | 1.572 | 0.271 | 0.879 | 20.711 | 494 |
| **Left insula** | 0.062 | 0.085 | [-0.105 - 0.229] | 1.403 | 0.466 | 0.879 | 65.825 | 494 |
| **Right postcentral gyrus** | 0.072 | 0.064 | [-0.053 - 0.197] | 1.723 | 0.259 | 0.879 | 38.529 | 496 |
| **Left inferior parietal cortex** | 0.077 | 0.074 | [-0.068 - 0.221] | 2.204 | 0.299 | 0.879 | 54.429 | 495 |
| **Left lateral occipital cortex** | 0.078 | 0.045 | [-0.009 - 0.166] | 2.027 | 0.079 | 0.786 | <0.001 | 495 |
| **Left lateral orbitofrontal cortex** | 0.078 | 0.074 | [-0.067 - 0.224] | 2.344 | 0.290 | 0.879 | 54.587 | 496 |
| **Right middle temporal gyrus** | 0.079 | 0.088 | [-0.094 - 0.252] | 2.251 | 0.372 | 0.879 | 68.343 | 489 |
| **Left middle temporal gyrus** | 0.081 | 0.110 | [-0.136 - 0.297] | 2.412 | 0.464 | 0.879 | 78.724 | 460 |
| **Right frontal pole** | 0.082 | 0.046 | [-0.009 - 0.172] | 2.518 | 0.076 | 0.786 | 3.131 | 496 |
| **Left parahippocampal gyrus** | 0.087 | 0.045 | [-0.001 - 0.175] | 3.721 | 0.052 | 0.786 | 0.713 | 495 |
| **Left fusiform gyrus** | 0.088 | 0.063 | [-0.036 - 0.213] | 2.578 | 0.163 | 0.879 | 37.707 | 493 |
| **Right lateral orbitofrontal cortex** | 0.089 | 0.084 | [-0.075 - 0.253] | 2.523 | 0.286 | 0.879 | 65.077 | 496 |
| **Left banks superior temporal sulcus** | 0.094 | 0.085 | [-0.071 - 0.26] | 3.273 | 0.265 | 0.879 | 60.020 | 456 |
| **Left inferior temporal gyrus** | 0.099 | 0.092 | [-0.082 - 0.28] | 3.282 | 0.284 | 0.879 | 71.424 | 478 |
| **Left caudal anterior cingulate cortex** | 0.100 | 0.076 | [-0.049 - 0.249] | 3.788 | 0.189 | 0.879 | 57.152 | 495 |
| **Left pars opercularis** | 0.104 | 0.084 | [-0.06 - 0.269] | 3.383 | 0.212 | 0.879 | 65.195 | 495 |
| **Left cuneus** | 0.107 | 0.080 | [-0.05 - 0.263] | 3.212 | 0.182 | 0.879 | 62.022 | 494 |
| **Right cuneus** | 0.125 | 0.070 | [-0.011 - 0.261] | 3.605 | 0.072 | 0.786 | 49.170 | 494 |
| **Right fusiform gyrus** | 0.141 | 0.063 | [0.018 - 0.265] | 4.265 | 0.024 | 0.786 | 38.814 | 493 |
| **Right caudal middle frontal gyrus** | 0.149 | 0.082 | [-0.012 - 0.31] | 5.431 | 0.070 | 0.786 | 65.302 | 495 |
| **Left caudal middle frontal gyrus** | 0.162 | 0.106 | [-0.046 - 0.37] | 5.406 | 0.127 | 0.879 | 82.457 | 496 |
| **Left temporal pole** | 0.206 | 0.074 | [0.06 - 0.352] | 6.712 | 0.006 | 0.397 | 58.096 | 485 |

**^a^** Included Samples: CLING, Houston, Sexpect, Muenster Cohort, NESDA, Novosibirsk, DepOx, Stanford, Sydney.

MDD: Major Depressive Disorder.

**Supplementary Table S26**: Full meta-analytic results for surface area of each structure for MDD patients with an adult age of onset (>21) versus Controls comparison controlling for age, sex and scan center. Adjusted Cohen's d is reported.

|  | **Cohen's d ^a^** | **Std. Err.** | **95% CI** | **% Difference** | **P-value** | **FDR P-value** | **I^2^** | **# Controls** | **# Patients** |
| --- | --- | --- | --- | --- | --- | --- | --- | --- | --- |
|  | **(adult-onset MDD vs CTL)** |  |  |  |  |  |  |  |  |
| **Left pars triangularis** | -0.054 | 0.037 | [-0.127 - 0.019] | -0.329 | 0.145 | 0.535 | 3.454 | 3305 | 1207 |
| **Right parahippocampal gyrus** | -0.041 | 0.036 | [-0.11 - 0.029] | -0.442 | 0.255 | 0.682 | <0.001 | 3277 | 1198 |
| **Left frontal pole** | -0.027 | 0.035 | [-0.096 - 0.042] | -0.284 | 0.445 | 0.807 | <0.001 | 3328 | 1213 |
| **Right pars triangularis** | -0.022 | 0.035 | [-0.091 - 0.048] | -0.126 | 0.539 | 0.819 | <0.001 | 3293 | 1206 |
| **Right fusiform gyrus** | -0.022 | 0.036 | [-0.092 - 0.049] | -0.106 | 0.547 | 0.819 | 0.003 | 3194 | 1182 |
| **Left pars opercularis** | -0.014 | 0.038 | [-0.088 - 0.059] | -0.068 | 0.705 | 0.907 | 4.425 | 3290 | 1203 |
| **Right transverse temporal gyrus** | -0.007 | 0.036 | [-0.078 - 0.064] | -0.059 | 0.839 | 0.993 | 1.983 | 3327 | 1214 |
| **Left banks superior temporal sulcus** | -0.007 | 0.042 | [-0.089 - 0.075] | -0.052 | 0.866 | 0.993 | 10.555 | 3162 | 1110 |
| **Left posterior cingulate cortex** | -0.005 | 0.035 | [-0.074 - 0.065] | -0.030 | 0.893 | 0.993 | <0.001 | 3318 | 1210 |
| **Left pericalcarine cortex** | -0.003 | 0.035 | [-0.073 - 0.066] | -0.022 | 0.930 | 0.993 | 0.013 | 3325 | 1208 |
| **Left inferior parietal cortex** | -0.003 | 0.036 | [-0.072 - 0.067] | -0.015 | 0.940 | 0.993 | <0.001 | 3258 | 1198 |
| **Left cuneus** | -0.002 | 0.036 | [-0.071 - 0.068] | -0.011 | 0.959 | 0.993 | <0.001 | 3297 | 1202 |
| **Left transverse temporal gyrus** | -3.05E-04 | 0.035 | [-0.07 - 0.069] | -0.002 | 0.993 | 0.993 | 0.007 | 3326 | 1212 |
| **Right supramarginal gyrus** | 4.43E-04 | 0.036 | [-0.07 - 0.071] | 0.002 | 0.990 | 0.993 | <0.001 | 3242 | 1182 |
| **Right banks superior temporal sulcus** | 0.001 | 0.036 | [-0.069 - 0.072] | 0.010 | 0.969 | 0.993 | 0.002 | 3244 | 1152 |
| **Left middle temporal gyrus** | 0.002 | 0.036 | [-0.069 - 0.073] | 0.012 | 0.957 | 0.993 | <0.001 | 3201 | 1150 |
| **Right pars opercularis** | 0.003 | 0.046 | [-0.086 - 0.093] | 0.018 | 0.939 | 0.993 | 22.531 | 3291 | 1205 |
| **Right inferior parietal cortex** | 0.006 | 0.045 | [-0.081 - 0.094] | 0.036 | 0.887 | 0.993 | 19.805 | 3255 | 1196 |
| **Right superior parietal cortex** | 0.011 | 0.042 | [-0.071 - 0.092] | 0.054 | 0.793 | 0.957 | 12.890 | 3282 | 1194 |
| **Left caudal middle frontal gyrus** | 0.012 | 0.044 | [-0.075 - 0.098] | 0.059 | 0.793 | 0.957 | 18.974 | 3276 | 1200 |
| **Right caudal middle frontal gyrus** | 0.013 | 0.041 | [-0.067 - 0.093] | 0.066 | 0.757 | 0.946 | 11.490 | 3291 | 1203 |
| **Right inferior temporal gyrus** | 0.013 | 0.035 | [-0.056 - 0.083] | 0.071 | 0.713 | 0.907 | 0.002 | 3305 | 1207 |
| **Left parahippocampal gyrus** | 0.015 | 0.036 | [-0.055 - 0.084] | 0.181 | 0.683 | 0.907 | <0.001 | 3266 | 1200 |
| **Right caudal anterior cingulate cortex** | 0.016 | 0.036 | [-0.054 - 0.086] | 0.180 | 0.650 | 0.892 | <0.001 | 3287 | 1200 |
| **Right superior temporal gyrus** | 0.017 | 0.036 | [-0.054 - 0.088] | 0.102 | 0.636 | 0.890 | 0.010 | 3225 | 1159 |
| **Right precuneus** | 0.019 | 0.052 | [-0.083 - 0.121] | 0.095 | 0.712 | 0.907 | 36.000 | 3309 | 1210 |
| **Left paracentral lobule** | 0.020 | 0.036 | [-0.051 - 0.09] | 0.112 | 0.583 | 0.838 | 0.023 | 3218 | 1179 |
| **Right precentral gyrus** | 0.021 | 0.038 | [-0.054 - 0.095] | 0.123 | 0.587 | 0.838 | 5.265 | 3261 | 1201 |
| **Left lingual gyrus** | 0.022 | 0.036 | [-0.048 - 0.092] | 0.130 | 0.530 | 0.819 | 0.514 | 3311 | 1207 |
| **Left entorhinal cortex** | 0.024 | 0.036 | [-0.048 - 0.095] | 0.272 | 0.516 | 0.819 | 0.004 | 3107 | 1159 |
| **Right frontal pole** | 0.024 | 0.040 | [-0.054 - 0.101] | 0.247 | 0.550 | 0.819 | 8.991 | 3324 | 1212 |
| **Left caudal anterior cingulate cortex** | 0.024 | 0.036 | [-0.045 - 0.094] | 0.277 | 0.494 | 0.819 | <0.001 | 3261 | 1201 |
| **Left superior parietal cortex** | 0.026 | 0.041 | [-0.055 - 0.108] | 0.129 | 0.523 | 0.819 | 12.586 | 3264 | 1196 |
| **Right medial orbitofrontal cortex** | 0.027 | 0.036 | [-0.043 - 0.096] | 0.223 | 0.455 | 0.807 | 0.013 | 3277 | 1199 |
| **Left fusiform gyrus** | 0.027 | 0.036 | [-0.044 - 0.097] | 0.131 | 0.458 | 0.807 | 0.007 | 3175 | 1185 |
| **Right insula** | 0.030 | 0.036 | [-0.04 - 0.099] | 0.162 | 0.403 | 0.783 | 0.006 | 3313 | 1204 |
| **Right lateral orbitofrontal cortex** | 0.031 | 0.042 | [-0.052 - 0.114] | 0.184 | 0.461 | 0.807 | 15.437 | 3326 | 1214 |
| **Right pericalcarine cortex** | 0.033 | 0.035 | [-0.037 - 0.102] | 0.238 | 0.356 | 0.712 | <0.001 | 3323 | 1210 |
| **Right postcentral gyrus** | 0.034 | 0.036 | [-0.036 - 0.104] | 0.192 | 0.341 | 0.702 | 0.015 | 3267 | 1192 |
| **Right paracentral lobule** | 0.035 | 0.036 | [-0.035 - 0.105] | 0.198 | 0.328 | 0.697 | 0.003 | 3265 | 1195 |
| **Left precuneus** | 0.035 | 0.036 | [-0.035 - 0.105] | 0.167 | 0.324 | 0.697 | 0.128 | 3307 | 1209 |
| **Right superior frontal gyrus** | 0.036 | 0.036 | [-0.034 - 0.106] | 0.173 | 0.319 | 0.697 | 0.011 | 3260 | 1192 |
| **Left pars orbitalis** | 0.036 | 0.035 | [-0.034 - 0.105] | 0.261 | 0.313 | 0.697 | <0.001 | 3319 | 1213 |
| **Left rostral middle frontal gyrus** | 0.039 | 0.035 | [-0.031 - 0.108] | 0.188 | 0.273 | 0.682 | 0.029 | 3306 | 1210 |
| **Left superior temporal gyrus** | 0.039 | 0.037 | [-0.033 - 0.111] | 0.238 | 0.289 | 0.696 | 0.783 | 3184 | 1148 |
| **Right hemisphere total surface area** | 0.039 | 0.035 | [-0.03 - 0.108] | 0.408 | 0.269 | 0.682 | <0.001 | 3329 | 1214 |
| **Left postcentral gyrus** | 0.039 | 0.036 | [-0.031 - 0.109] | 0.217 | 0.273 | 0.682 | 0.006 | 3242 | 1186 |
| **Right middle temporal gyrus** | 0.041 | 0.036 | [-0.029 - 0.111] | 0.230 | 0.247 | 0.682 | 0.010 | 3265 | 1193 |
| **Right rostral anterior cingulate cortex** | 0.044 | 0.036 | [-0.026 - 0.114] | 0.445 | 0.219 | 0.682 | <0.001 | 3266 | 1195 |
| **Left hemisphere total surface area** | 0.047 | 0.035 | [-0.022 - 0.117] | 0.494 | 0.180 | 0.599 | 0.016 | 3329 | 1214 |
| **Right lingual gyrus** | 0.052 | 0.035 | [-0.017 - 0.122] | 0.304 | 0.139 | 0.535 | <0.001 | 3322 | 1210 |
| **Left insula** | 0.056 | 0.035 | [-0.013 - 0.126] | 0.294 | 0.111 | 0.457 | 0.017 | 3306 | 1213 |
| **Left lateral occipital cortex** | 0.059 | 0.035 | [-0.011 - 0.128] | 0.339 | 0.097 | 0.425 | 0.002 | 3304 | 1206 |
| **Left rostral anterior cingulate cortex** | 0.059 | 0.036 | [-0.011 - 0.13] | 0.579 | 0.097 | 0.425 | 0.003 | 3211 | 1187 |
| **Right posterior cingulate cortex** | 0.060 | 0.035 | [-0.01 - 0.129] | 0.400 | 0.092 | 0.425 | 0.003 | 3319 | 1212 |
| **Left superior frontal gyrus** | 0.060 | 0.036 | [-0.01 - 0.13] | 0.304 | 0.093 | 0.425 | <0.001 | 3226 | 1197 |
| **Right rostral middle frontal gyrus** | 0.061 | 0.036 | [-0.009 - 0.131] | 0.294 | 0.086 | 0.425 | 0.019 | 3307 | 1204 |
| **Left inferior temporal gyrus** | 0.062 | 0.036 | [-0.008 - 0.132] | 0.347 | 0.081 | 0.425 | 0.002 | 3294 | 1196 |
| **Right pars orbitalis** | 0.064 | 0.035 | [-0.005 - 0.133] | 0.460 | 0.071 | 0.425 | <0.001 | 3321 | 1213 |
| **Left medial orbitofrontal cortex** | 0.064 | 0.046 | [-0.027 - 0.155] | 0.499 | 0.165 | 0.578 | 23.479 | 3228 | 1193 |
| **Left supramarginal gyrus** | 0.068 | 0.036 | [-0.003 - 0.139] | 0.365 | 0.061 | 0.425 | 0.001 | 3185 | 1167 |
| **Left lateral orbitofrontal cortex** | 0.072 | 0.059 | [-0.044 - 0.188] | 0.433 | 0.225 | 0.682 | 49.036 | 3326 | 1213 |
| **Left temporal pole** | 0.073 | 0.036 | [0.003 - 0.142] | 0.734 | 0.041 | 0.361 | <0.001 | 3291 | 1200 |
| **Right isthmus cingulate cortex** | 0.073 | 0.035 | [0.004 - 0.142] | 0.579 | 0.039 | 0.361 | <0.001 | 3316 | 1212 |
| **Right entorhinal cortex** | 0.084 | 0.037 | [0.013 - 0.156] | 1.003 | 0.021 | 0.361 | 0.003 | 3082 | 1140 |
| **Left precentral gyrus** | 0.085 | 0.042 | [0.004 - 0.167] | 0.490 | 0.040 | 0.361 | 13.139 | 3259 | 1198 |
| **Right temporal pole** | 0.088 | 0.036 | [0.018 - 0.158] | 0.970 | 0.014 | 0.361 | 0.001 | 3246 | 1194 |
| **Right cuneus** | 0.099 | 0.047 | [0.007 - 0.19] | 0.605 | 0.034 | 0.361 | 24.472 | 3307 | 1206 |
| **Right lateral occipital cortex** | 0.105 | 0.035 | [0.035 - 0.174] | 0.613 | 0.003 | 0.219 | <0.001 | 3313 | 1210 |
| **Left isthmus cingulate cortex** | 0.111 | 0.046 | [0.02 - 0.201] | 0.849 | 0.017 | 0.361 | 24.080 | 3312 | 1208 |

**^a^** Included Samples: CLING, Imaging Genetics Dublin, Clinical Depression Dublin, Bipolar Family Study, Houston, Sexpect, MMDP 3T, MPIP, Muenster Cohort, NESDA, Novosibirsk, DepOx, QTIM, SHIP, SHIP-trend, Sydney, Stanford.

MDD: Major Depressive Disorder; CTL: Controls.

**Supplementary Table S27**: Full meta-analytic results for surface area of each structure for MDD patients with an adolescent age of onset (≤21) versus Controls comparison controlling for age, sex and scan center. Adjusted Cohen's d is reported.

|  | **Cohen's d ^a^** | **Std. Err.** | **95% CI** | **% Difference** | **P-value** | **FDR P-value** | **I^2^** | **# Controls** | **# Patients** |
| --- | --- | --- | --- | --- | --- | --- | --- | --- | --- |
|  | **(adolescent-onset MDD vs CTL)** |  |  |  |  |  |  |  |  |
| **Left frontal pole** | -0.062 | 0.072 | [-0.204 - 0.08] | -0.650 | 0.394 | 0.936 | 40.472 | 2884 | 472 |
| **Left pars triangularis** | -0.059 | 0.053 | [-0.163 - 0.046] | -0.358 | 0.270 | 0.936 | 0.001 | 2862 | 472 |
| **Right entorhinal cortex** | -0.052 | 0.055 | [-0.16 - 0.056] | -0.616 | 0.346 | 0.936 | <0.001 | 2660 | 448 |
| **Left parahippocampal gyrus** | -0.046 | 0.082 | [-0.206 - 0.114] | -0.576 | 0.571 | 0.943 | 51.914 | 2823 | 466 |
| **Right fusiform gyrus** | -0.042 | 0.059 | [-0.157 - 0.074] | -0.203 | 0.480 | 0.936 | 12.618 | 2750 | 462 |
| **Right parahippocampal gyrus** | -0.034 | 0.054 | [-0.139 - 0.071] | -0.366 | 0.531 | 0.943 | 0.007 | 2834 | 468 |
| **Left pericalcarine cortex** | -0.024 | 0.053 | [-0.129 - 0.08] | -0.172 | 0.646 | 0.943 | <0.001 | 2881 | 472 |
| **Left banks superior temporal sulcus** | -0.023 | 0.055 | [-0.131 - 0.085] | -0.168 | 0.677 | 0.943 | <0.001 | 2734 | 439 |
| **Left insula** | -0.023 | 0.072 | [-0.165 - 0.119] | -0.120 | 0.751 | 0.943 | 39.917 | 2865 | 470 |
| **Right lingual gyrus** | -0.022 | 0.053 | [-0.127 - 0.083] | -0.128 | 0.680 | 0.943 | <0.001 | 2877 | 472 |
| **Left rostral anterior cingulate cortex** | -0.022 | 0.054 | [-0.128 - 0.085] | -0.210 | 0.690 | 0.943 | <0.001 | 2768 | 460 |
| **Right transverse temporal gyrus** | -0.018 | 0.053 | [-0.123 - 0.086] | -0.147 | 0.731 | 0.943 | <0.001 | 2883 | 472 |
| **Left entorhinal cortex** | -0.015 | 0.055 | [-0.122 - 0.092] | -0.168 | 0.789 | 0.943 | <0.001 | 2692 | 454 |
| **Right superior parietal cortex** | -0.013 | 0.060 | [-0.131 - 0.105] | -0.063 | 0.833 | 0.943 | 16.482 | 2837 | 467 |
| **Left pars orbitalis** | -0.004 | 0.069 | [-0.139 - 0.131] | -0.031 | 0.951 | 0.986 | 34.552 | 2874 | 472 |
| **Left postcentral gyrus** | -0.002 | 0.054 | [-0.107 - 0.104] | -0.009 | 0.977 | 0.986 | <0.001 | 2799 | 465 |
| **Right pars orbitalis** | -0.001 | 0.055 | [-0.109 - 0.106] | -0.010 | 0.979 | 0.986 | 3.927 | 2879 | 471 |
| **Left lingual gyrus** | 0.003 | 0.053 | [-0.102 - 0.107] | 0.015 | 0.961 | 0.986 | 0.001 | 2867 | 471 |
| **Right precentral gyrus** | 0.003 | 0.054 | [-0.102 - 0.108] | 0.017 | 0.958 | 0.986 | 0.004 | 2817 | 466 |
| **Right pericalcarine cortex** | 0.007 | 0.053 | [-0.098 - 0.111] | 0.048 | 0.901 | 0.986 | <0.001 | 2880 | 472 |
| **Left transverse temporal gyrus** | 0.010 | 0.053 | [-0.094 - 0.115] | 0.081 | 0.847 | 0.943 | <0.001 | 2883 | 472 |
| **Left fusiform gyrus** | 0.010 | 0.054 | [-0.096 - 0.116] | 0.051 | 0.849 | 0.943 | <0.001 | 2731 | 461 |
| **Right posterior cingulate cortex** | 0.011 | 0.053 | [-0.094 - 0.115] | 0.072 | 0.841 | 0.943 | <0.001 | 2875 | 471 |
| **Right lateral orbitofrontal cortex** | 0.011 | 0.053 | [-0.093 - 0.116] | 0.066 | 0.832 | 0.943 | <0.001 | 2882 | 472 |
| **Left pars opercularis** | 0.016 | 0.072 | [-0.124 - 0.156] | 0.076 | 0.823 | 0.943 | 38.832 | 2847 | 471 |
| **Right caudal anterior cingulate cortex** | 0.017 | 0.064 | [-0.108 - 0.141] | 0.186 | 0.792 | 0.943 | 24.104 | 2844 | 469 |
| **Right precuneus** | 0.018 | 0.053 | [-0.087 - 0.122] | 0.087 | 0.742 | 0.943 | 0.003 | 2863 | 472 |
| **Right hemisphere total surface area** | 0.022 | 0.053 | [-0.082 - 0.127] | 0.233 | 0.675 | 0.943 | <0.001 | 2885 | 472 |
| **Right cuneus** | 0.023 | 0.054 | [-0.082 - 0.128] | 0.141 | 0.669 | 0.943 | 0.545 | 2863 | 469 |
| **Right pars opercularis** | 0.023 | 0.061 | [-0.097 - 0.143] | 0.118 | 0.701 | 0.943 | 18.607 | 2849 | 467 |
| **Right inferior parietal cortex** | 0.025 | 0.062 | [-0.097 - 0.148] | 0.143 | 0.686 | 0.943 | 20.950 | 2813 | 465 |
| **Left superior parietal cortex** | 0.026 | 0.054 | [-0.079 - 0.131] | 0.127 | 0.629 | 0.943 | <0.001 | 2819 | 467 |
| **Left superior temporal gyrus** | 0.027 | 0.055 | [-0.081 - 0.135] | 0.163 | 0.629 | 0.943 | <0.001 | 2759 | 441 |
| **Right caudal middle frontal gyrus** | 0.027 | 0.053 | [-0.078 - 0.132] | 0.142 | 0.613 | 0.943 | 0.012 | 2847 | 471 |
| **Right frontal pole** | 0.030 | 0.056 | [-0.081 - 0.14] | 0.308 | 0.599 | 0.943 | 7.557 | 2881 | 472 |
| **Left cuneus** | 0.034 | 0.057 | [-0.078 - 0.146] | 0.210 | 0.550 | 0.943 | 9.563 | 2857 | 472 |
| **Right inferior temporal gyrus** | 0.038 | 0.054 | [-0.068 - 0.143] | 0.205 | 0.482 | 0.936 | 0.003 | 2861 | 464 |
| **Right rostral anterior cingulate cortex** | 0.039 | 0.054 | [-0.066 - 0.144] | 0.396 | 0.467 | 0.936 | 0.021 | 2826 | 468 |
| **Right superior frontal gyrus** | 0.041 | 0.053 | [-0.064 - 0.145] | 0.197 | 0.447 | 0.936 | <0.001 | 2816 | 470 |
| **Left caudal anterior cingulate cortex** | 0.041 | 0.054 | [-0.065 - 0.146] | 0.463 | 0.448 | 0.936 | <0.001 | 2818 | 466 |
| **Right insula** | 0.042 | 0.053 | [-0.062 - 0.147] | 0.231 | 0.427 | 0.936 | 0.005 | 2874 | 472 |
| **Right medial orbitofrontal cortex** | 0.044 | 0.062 | [-0.077 - 0.164] | 0.366 | 0.478 | 0.936 | 19.328 | 2837 | 468 |
| **Right postcentral gyrus** | 0.044 | 0.054 | [-0.061 - 0.149] | 0.248 | 0.415 | 0.936 | 0.007 | 2823 | 467 |
| **Right banks superior temporal sulcus** | 0.046 | 0.077 | [-0.105 - 0.196] | 0.327 | 0.550 | 0.943 | 44.709 | 2804 | 452 |
| **Left precentral gyrus** | 0.048 | 0.054 | [-0.057 - 0.153] | 0.276 | 0.370 | 0.936 | 0.011 | 2817 | 467 |
| **Left supramarginal gyrus** | 0.048 | 0.063 | [-0.074 - 0.171] | 0.260 | 0.440 | 0.936 | 19.646 | 2752 | 460 |
| **Left lateral orbitofrontal cortex** | 0.051 | 0.061 | [-0.069 - 0.171] | 0.310 | 0.403 | 0.936 | 19.678 | 2882 | 472 |
| **Left rostral middle frontal gyrus** | 0.053 | 0.053 | [-0.052 - 0.158] | 0.257 | 0.322 | 0.936 | 0.002 | 2863 | 471 |
| **Left inferior parietal cortex** | 0.053 | 0.063 | [-0.071 - 0.177] | 0.303 | 0.399 | 0.936 | 22.521 | 2816 | 465 |
| **Left superior frontal gyrus** | 0.054 | 0.054 | [-0.052 - 0.159] | 0.271 | 0.319 | 0.936 | <0.001 | 2782 | 466 |
| **Right pars triangularis** | 0.057 | 0.064 | [-0.067 - 0.182] | 0.331 | 0.368 | 0.936 | 24.392 | 2853 | 471 |
| **Right paracentral lobule** | 0.058 | 0.053 | [-0.047 - 0.163] | 0.330 | 0.277 | 0.936 | <0.001 | 2822 | 470 |
| **Left temporal pole** | 0.059 | 0.070 | [-0.08 - 0.197] | 0.591 | 0.406 | 0.936 | 35.202 | 2848 | 456 |
| **Left hemisphere total surface area** | 0.066 | 0.053 | [-0.039 - 0.171] | 0.686 | 0.216 | 0.936 | 0.013 | 2885 | 472 |
| **Left caudal middle frontal gyrus** | 0.068 | 0.054 | [-0.037 - 0.173] | 0.345 | 0.207 | 0.936 | <0.001 | 2832 | 469 |
| **Left medial orbitofrontal cortex** | 0.068 | 0.057 | [-0.043 - 0.179] | 0.526 | 0.230 | 0.936 | 6.553 | 2788 | 462 |
| **Right isthmus cingulate cortex** | 0.068 | 0.065 | [-0.058 - 0.195] | 0.542 | 0.290 | 0.936 | 26.447 | 2872 | 470 |
| **Right lateral occipital cortex** | 0.070 | 0.072 | [-0.071 - 0.211] | 0.411 | 0.328 | 0.936 | 39.315 | 2869 | 471 |
| **Left lateral occipital cortex** | 0.076 | 0.053 | [-0.029 - 0.181] | 0.437 | 0.156 | 0.936 | 0.008 | 2860 | 471 |
| **Left isthmus cingulate cortex** | 0.076 | 0.053 | [-0.028 - 0.181] | 0.586 | 0.154 | 0.936 | 0.001 | 2868 | 471 |
| **Right temporal pole** | 0.081 | 0.056 | [-0.03 - 0.191] | 0.891 | 0.151 | 0.936 | 4.867 | 2802 | 451 |
| **Left middle temporal gyrus** | 0.081 | 0.055 | [-0.026 - 0.188] | 0.482 | 0.137 | 0.936 | 0.018 | 2768 | 448 |
| **Right supramarginal gyrus** | 0.084 | 0.066 | [-0.046 - 0.214] | 0.431 | 0.206 | 0.936 | 28.599 | 2806 | 462 |
| **Right middle temporal gyrus** | 0.086 | 0.071 | [-0.053 - 0.225] | 0.480 | 0.226 | 0.936 | 36.720 | 2823 | 458 |
| **Left posterior cingulate cortex** | 0.091 | 0.053 | [-0.014 - 0.196] | 0.569 | 0.089 | 0.936 | <0.001 | 2873 | 470 |
| **Right superior temporal gyrus** | 0.093 | 0.055 | [-0.015 - 0.201] | 0.558 | 0.091 | 0.936 | 0.005 | 2790 | 436 |
| **Left paracentral lobule** | 0.105 | 0.053 | [0 - 0.21] | 0.596 | 0.050 | 0.936 | 0.003 | 2774 | 471 |
| **Right rostral middle frontal gyrus** | 0.109 | 0.054 | [0.004 - 0.213] | 0.524 | 0.042 | 0.936 | 0.005 | 2863 | 470 |
| **Left inferior temporal gyrus** | 0.138 | 0.054 | [0.032 - 0.244] | 0.771 | 0.010 | 0.734 | <0.001 | 2856 | 461 |
| **Left precuneus** | <0.001 | 0.056 | [-0.109 - 0.111] | 0.005 | 0.986 | 0.986 | 6.854 | 2863 | 470 |

**^a^** Included Samples: Imaging Genetics Dublin, Houston, MMDP 3T, Melbourne, MPIP, Muenster Cohort, NESDA, QTIM, SHIP, SHIP-trend, Sydney, Stanford.

MDD: Major Depressive Disorder; CTL: Controls.

**Supplementary Table S28**: Full meta-analytic results for surface area of each structure for MDD patients with an adolescent age of onset (≤21) versus MDD patients with an adult age of onset (>21) comparison controlling for age, sex and scan center. Adjusted Cohen's d is reported.

|  | **Cohen's d ^a^** | **Std. Err.** | **95% CI** | **% Difference** | **P-value** | **FDR P-value** | **I^2^** | **# adolescent-onset MDD** | **# adult-onset MDD** |
| --- | --- | --- | --- | --- | --- | --- | --- | --- | --- |
|  | **(adolescent-onset MDD vs adult-onset MDD)** |  |  |  |  |  |  |  |  |
| **Right supramarginal gyrus** | -0.167 | 0.060 | [-0.285 - -0.049] | -0.856 | 0.006 | 0.271 | 0.009 | 453 | 1060 |
| **Left inferior parietal cortex** | -0.160 | 0.060 | [-0.278 - -0.042] | -0.908 | 0.008 | 0.271 | <0.001 | 455 | 1072 |
| **Right superior temporal gyrus** | -0.144 | 0.117 | [-0.374 - 0.086] | -0.861 | 0.220 | 0.961 | 66.976 | 427 | 1040 |
| **Left inferior temporal gyrus** | -0.134 | 0.060 | [-0.251 - -0.016] | -0.746 | 0.027 | 0.464 | 0.003 | 451 | 1076 |
| **Left middle temporal gyrus** | -0.125 | 0.061 | [-0.244 - -0.006] | -0.742 | 0.040 | 0.521 | 0.001 | 438 | 1036 |
| **Right middle temporal gyrus** | -0.123 | 0.096 | [-0.312 - 0.066] | -0.684 | 0.203 | 0.961 | 54.708 | 449 | 1068 |
| **Left caudal anterior cingulate cortex** | -0.120 | 0.060 | [-0.238 - -0.003] | -1.368 | 0.045 | 0.521 | <0.001 | 456 | 1076 |
| **Right pars triangularis** | -0.112 | 0.060 | [-0.229 - 0.004] | -0.647 | 0.059 | 0.593 | <0.001 | 461 | 1081 |
| **Left posterior cingulate cortex** | -0.100 | 0.060 | [-0.217 - 0.016] | -0.629 | 0.092 | 0.802 | <0.001 | 461 | 1084 |
| **Right precuneus** | -0.092 | 0.060 | [-0.208 - 0.025] | -0.455 | 0.124 | 0.961 | 0.001 | 462 | 1084 |
| **Right transverse temporal gyrus** | -0.089 | 0.093 | [-0.271 - 0.092] | -0.719 | 0.335 | 0.961 | 52.500 | 462 | 1088 |
| **Right inferior temporal gyrus** | -0.088 | 0.060 | [-0.205 - 0.03] | -0.476 | 0.142 | 0.961 | 0.002 | 454 | 1081 |
| **Right insula** | -0.087 | 0.082 | [-0.248 - 0.074] | -0.474 | 0.290 | 0.961 | 40.463 | 462 | 1082 |
| **Right postcentral gyrus** | -0.081 | 0.060 | [-0.198 - 0.037] | -0.457 | 0.178 | 0.961 | 0.004 | 457 | 1066 |
| **Right banks superior temporal sulcus** | -0.077 | 0.061 | [-0.196 - 0.042] | -0.547 | 0.206 | 0.961 | 0.007 | 443 | 1030 |
| **Left hemisphere total surface area** | -0.076 | 0.060 | [-0.194 - 0.042] | -0.793 | 0.205 | 0.961 | 1.473 | 462 | 1088 |
| **Left paracentral lobule** | -0.068 | 0.060 | [-0.185 - 0.049] | -0.388 | 0.253 | 0.961 | <0.001 | 461 | 1053 |
| **Right hemisphere total surface area** | -0.067 | 0.068 | [-0.2 - 0.066] | -0.697 | 0.324 | 0.961 | 17.056 | 462 | 1088 |
| **Right inferior parietal cortex** | -0.067 | 0.060 | [-0.184 - 0.051] | -0.378 | 0.267 | 0.961 | 0.001 | 455 | 1070 |
| **Left lateral occipital cortex** | -0.066 | 0.060 | [-0.183 - 0.051] | -0.379 | 0.269 | 0.961 | 0.010 | 461 | 1081 |
| **Left medial orbitofrontal cortex** | -0.062 | 0.060 | [-0.18 - 0.056] | -0.479 | 0.305 | 0.961 | <0.001 | 452 | 1068 |
| **Left superior frontal gyrus** | -0.062 | 0.070 | [-0.199 - 0.075] | -0.312 | 0.378 | 0.961 | 20.224 | 456 | 1072 |
| **Left isthmus cingulate cortex** | -0.054 | 0.060 | [-0.171 - 0.063] | -0.416 | 0.364 | 0.961 | 0.007 | 461 | 1084 |
| **Right isthmus cingulate cortex** | -0.050 | 0.077 | [-0.201 - 0.101] | -0.395 | 0.517 | 0.961 | 32.628 | 460 | 1087 |
| **Left cuneus** | -0.048 | 0.070 | [-0.185 - 0.089] | -0.296 | 0.494 | 0.961 | 21.482 | 462 | 1079 |
| **Left superior parietal cortex** | -0.047 | 0.060 | [-0.164 - 0.07] | -0.230 | 0.433 | 0.961 | <0.001 | 457 | 1071 |
| **Right rostral middle frontal gyrus** | -0.043 | 0.079 | [-0.198 - 0.111] | -0.210 | 0.581 | 0.961 | 35.382 | 460 | 1078 |
| **Left lateral orbitofrontal cortex** | -0.039 | 0.087 | [-0.209 - 0.131] | -0.234 | 0.655 | 0.961 | 46.116 | 462 | 1087 |
| **Right lateral orbitofrontal cortex** | -0.036 | 0.059 | [-0.153 - 0.08] | -0.214 | 0.542 | 0.961 | 0.001 | 462 | 1088 |
| **Left rostral middle frontal gyrus** | -0.035 | 0.060 | [-0.152 - 0.082] | -0.170 | 0.556 | 0.961 | 0.003 | 461 | 1084 |
| **Right medial orbitofrontal cortex** | -0.034 | 0.073 | [-0.177 - 0.109] | -0.284 | 0.643 | 0.961 | 25.571 | 458 | 1074 |
| **Right paracentral lobule** | -0.034 | 0.060 | [-0.151 - 0.083] | -0.191 | 0.573 | 0.961 | 0.010 | 460 | 1069 |
| **Left fusiform gyrus** | -0.033 | 0.060 | [-0.151 - 0.085] | -0.163 | 0.582 | 0.961 | <0.001 | 451 | 1059 |
| **Right precentral gyrus** | -0.033 | 0.060 | [-0.15 - 0.084] | -0.195 | 0.583 | 0.961 | 0.003 | 457 | 1076 |
| **Left caudal middle frontal gyrus** | -0.033 | 0.060 | [-0.15 - 0.084] | -0.166 | 0.585 | 0.961 | <0.001 | 459 | 1074 |
| **Left supramarginal gyrus** | -0.031 | 0.077 | [-0.181 - 0.12] | -0.165 | 0.690 | 0.961 | 30.315 | 450 | 1050 |
| **Right superior frontal gyrus** | -0.030 | 0.060 | [-0.147 - 0.087] | -0.146 | 0.613 | 0.961 | <0.001 | 460 | 1066 |
| **Left precentral gyrus** | -0.027 | 0.060 | [-0.144 - 0.091] | -0.153 | 0.656 | 0.961 | <0.001 | 458 | 1072 |
| **Left pars orbitalis** | -0.026 | 0.059 | [-0.143 - 0.09] | -0.193 | 0.657 | 0.961 | 0.001 | 462 | 1087 |
| **Right caudal anterior cingulate cortex** | -0.021 | 0.060 | [-0.138 - 0.096] | -0.231 | 0.728 | 0.961 | 0.007 | 460 | 1075 |
| **Right rostral anterior cingulate cortex** | -0.019 | 0.060 | [-0.137 - 0.098] | -0.197 | 0.745 | 0.961 | <0.001 | 458 | 1070 |
| **Left pars opercularis** | -0.018 | 0.115 | [-0.243 - 0.208] | -0.084 | 0.878 | 0.977 | 69.078 | 461 | 1078 |
| **Right posterior cingulate cortex** | -0.017 | 0.069 | [-0.153 - 0.118] | -0.117 | 0.800 | 0.961 | 19.476 | 462 | 1086 |
| **Left banks superior temporal sulcus** | -0.017 | 0.062 | [-0.138 - 0.104] | -0.125 | 0.781 | 0.961 | 0.007 | 429 | 998 |
| **Left lingual gyrus** | -0.015 | 0.059 | [-0.132 - 0.101] | -0.089 | 0.795 | 0.961 | <0.001 | 462 | 1081 |
| **Left frontal pole** | -0.015 | 0.101 | [-0.213 - 0.183] | -0.161 | 0.880 | 0.977 | 59.859 | 462 | 1087 |
| **Right superior parietal cortex** | -0.013 | 0.060 | [-0.13 - 0.104] | -0.065 | 0.827 | 0.961 | 0.018 | 457 | 1070 |
| **Left pars triangularis** | -0.006 | 0.061 | [-0.126 - 0.114] | -0.037 | 0.920 | 0.978 | 4.130 | 462 | 1083 |
| **Left postcentral gyrus** | -0.006 | 0.060 | [-0.124 - 0.112] | -0.034 | 0.919 | 0.978 | <0.001 | 455 | 1062 |
| **Left superior temporal gyrus** | -0.005 | 0.065 | [-0.132 - 0.123] | -0.028 | 0.944 | 0.978 | 7.919 | 431 | 1032 |
| **Right caudal middle frontal gyrus** | -0.004 | 0.060 | [-0.12 - 0.113] | -0.020 | 0.950 | 0.978 | <0.001 | 461 | 1077 |
| **Right pars orbitalis** | -0.002 | 0.060 | [-0.118 - 0.115] | -0.011 | 0.979 | 0.987 | <0.001 | 461 | 1087 |
| **Left insula** | -0.001 | 0.073 | [-0.144 - 0.141] | -0.006 | 0.987 | 0.987 | 26.008 | 460 | 1088 |
| **Right cuneus** | 0.006 | 0.098 | [-0.185 - 0.198] | 0.040 | 0.947 | 0.978 | 57.116 | 461 | 1083 |
| **Left pericalcarine cortex** | 0.015 | 0.060 | [-0.101 - 0.132] | 0.108 | 0.797 | 0.961 | 0.020 | 462 | 1086 |
| **Left precuneus** | 0.018 | 0.089 | [-0.157 - 0.193] | 0.087 | 0.838 | 0.961 | 48.703 | 461 | 1083 |
| **Left transverse temporal gyrus** | 0.019 | 0.070 | [-0.119 - 0.157] | 0.148 | 0.790 | 0.961 | 22.177 | 462 | 1086 |
| **Right pericalcarine cortex** | 0.020 | 0.059 | [-0.097 - 0.136] | 0.143 | 0.742 | 0.961 | <0.001 | 462 | 1087 |
| **Right fusiform gyrus** | 0.020 | 0.085 | [-0.147 - 0.186] | 0.097 | 0.815 | 0.961 | 42.821 | 452 | 1056 |
| **Right parahippocampal gyrus** | 0.021 | 0.070 | [-0.116 - 0.159] | 0.232 | 0.762 | 0.961 | 21.102 | 458 | 1074 |
| **Right pars opercularis** | 0.023 | 0.061 | [-0.096 - 0.141] | 0.113 | 0.710 | 0.961 | 1.811 | 457 | 1079 |
| **Right frontal pole** | 0.026 | 0.059 | [-0.091 - 0.142] | 0.270 | 0.664 | 0.961 | <0.001 | 462 | 1086 |
| **Left entorhinal cortex** | 0.026 | 0.080 | [-0.131 - 0.183] | 0.301 | 0.744 | 0.961 | 35.099 | 445 | 1047 |
| **Left temporal pole** | 0.027 | 0.072 | [-0.115 - 0.169] | 0.271 | 0.712 | 0.961 | 23.855 | 446 | 1074 |
| **Right lateral occipital cortex** | 0.033 | 0.060 | [-0.084 - 0.15] | 0.193 | 0.580 | 0.961 | <0.001 | 461 | 1084 |
| **Right temporal pole** | 0.036 | 0.087 | [-0.135 - 0.207] | 0.399 | 0.678 | 0.961 | 43.903 | 441 | 1068 |
| **Left parahippocampal gyrus** | 0.046 | 0.060 | [-0.071 - 0.164] | 0.578 | 0.439 | 0.961 | 0.003 | 457 | 1074 |
| **Left rostral anterior cingulate cortex** | 0.049 | 0.060 | [-0.069 - 0.167] | 0.477 | 0.418 | 0.961 | <0.001 | 450 | 1063 |
| **Right lingual gyrus** | 0.059 | 0.059 | [-0.058 - 0.175] | 0.341 | 0.323 | 0.961 | <0.001 | 462 | 1085 |
| **Right entorhinal cortex** | 0.140 | 0.061 | [0.02 - 0.26] | 1.664 | 0.022 | 0.464 | <0.001 | 438 | 1027 |

**^a^** Included Samples: Imaging Genetics Dublin, Houston, MMDP 3T, MPIP, Muenster Cohort, NESDA, QTIM, SHIP, SHIP-trend, Sydney, Stanford.

MDD: Major Depressive Disorder; CTL: Controls.

**Supplementary Table S29**: Full meta-analytic results for surface area of each structure for MDD patients taking antidepressants at time of scanning versus Controls comparison controlling for age, sex and scan center. Adjusted Cohen's d is reported.

|  | **Cohen's d ^a^** | **Std. Err.** | **95% CI** | **% Difference** | **P-value** | **FDR P-value** | **I^2^** | **# Controls** | **# Patients** |
| --- | --- | --- | --- | --- | --- | --- | --- | --- | --- |
|  | **(AD MDD vs CTL)** |  |  |  |  |  |  |  |  |
| **Right transverse temporal gyrus** | -0.045 | 0.041 | [-0.126 - 0.035] | -0.752 | 0.270 | 0.681 | <0.001 | 7221 | 951 |
| **Left pars triangularis** | -0.044 | 0.041 | [-0.124 - 0.037] | -0.671 | 0.290 | 0.681 | <0.001 | 7199 | 949 |
| **Right fusiform gyrus** | -0.037 | 0.041 | [-0.118 - 0.044] | -0.555 | 0.368 | 0.681 | <0.001 | 7089 | 945 |
| **Right parahippocampal gyrus** | -0.033 | 0.044 | [-0.119 - 0.053] | -0.544 | 0.448 | 0.681 | 4.975 | 7171 | 944 |
| **Left frontal pole** | -0.032 | 0.041 | [-0.113 - 0.049] | -0.499 | 0.435 | 0.681 | <0.001 | 7222 | 950 |
| **Left pericalcarine cortex** | -0.030 | 0.062 | [-0.151 - 0.091] | -0.519 | 0.626 | 0.780 | 41.759 | 7219 | 947 |
| **Left inferior parietal cortex** | -0.027 | 0.049 | [-0.123 - 0.07] | -0.384 | 0.586 | 0.759 | 16.220 | 7152 | 946 |
| **Left banks superior temporal sulcus** | -0.020 | 0.043 | [-0.104 - 0.063] | -0.351 | 0.633 | 0.780 | <0.001 | 7073 | 869 |
| **Right banks superior temporal sulcus** | -0.020 | 0.042 | [-0.102 - 0.062] | -0.312 | 0.635 | 0.780 | <0.001 | 7141 | 888 |
| **Right superior parietal cortex** | -0.011 | 0.041 | [-0.092 - 0.07] | -0.128 | 0.791 | 0.912 | <0.001 | 7176 | 938 |
| **Right pars triangularis** | -0.008 | 0.041 | [-0.089 - 0.072] | -0.133 | 0.842 | 0.912 | <0.001 | 7187 | 950 |
| **Right caudal anterior cingulate cortex** | -0.003 | 0.041 | [-0.084 - 0.078] | -0.064 | 0.936 | 0.964 | <0.001 | 7181 | 946 |
| **Right inferior parietal cortex** | -0.001 | 0.083 | [-0.164 - 0.162] | -0.018 | 0.987 | 0.987 | 68.026 | 7149 | 944 |
| **Left fusiform gyrus** | 0.002 | 0.041 | [-0.08 - 0.083] | 0.022 | 0.970 | 0.984 | <0.001 | 7070 | 944 |
| **Left cuneus** | 0.004 | 0.041 | [-0.077 - 0.085] | 0.058 | 0.925 | 0.964 | <0.001 | 7191 | 947 |
| **Left parahippocampal gyrus** | 0.007 | 0.041 | [-0.074 - 0.088] | 0.155 | 0.860 | 0.912 | <0.001 | 7161 | 945 |
| **Right superior temporal gyrus** | 0.009 | 0.042 | [-0.073 - 0.091] | 0.108 | 0.827 | 0.912 | <0.001 | 7128 | 905 |
| **Right pericalcarine cortex** | 0.010 | 0.041 | [-0.07 - 0.091] | 0.173 | 0.801 | 0.912 | <0.001 | 7217 | 947 |
| **Right pars opercularis** | 0.012 | 0.041 | [-0.069 - 0.093] | 0.194 | 0.769 | 0.912 | <0.001 | 7185 | 951 |
| **Left pars opercularis** | 0.016 | 0.086 | [-0.152 - 0.185] | 0.262 | 0.850 | 0.912 | 70.494 | 7185 | 949 |
| **Left rostral anterior cingulate cortex** | 0.019 | 0.041 | [-0.062 - 0.1] | 0.411 | 0.652 | 0.787 | <0.001 | 7105 | 942 |
| **Left entorhinal cortex** | 0.019 | 0.082 | [-0.141 - 0.178] | 0.416 | 0.819 | 0.912 | 65.612 | 7013 | 919 |
| **Left caudal anterior cingulate cortex** | 0.026 | 0.041 | [-0.055 - 0.107] | 0.484 | 0.534 | 0.719 | <0.001 | 7155 | 945 |
| **Right inferior temporal gyrus** | 0.028 | 0.041 | [-0.053 - 0.109] | 0.471 | 0.499 | 0.698 | <0.001 | 7200 | 947 |
| **Left transverse temporal gyrus** | 0.031 | 0.055 | [-0.077 - 0.14] | 0.515 | 0.574 | 0.758 | 30.169 | 7220 | 951 |
| **Right medial orbitofrontal cortex** | 0.034 | 0.041 | [-0.047 - 0.114] | 0.518 | 0.414 | 0.681 | <0.001 | 7181 | 950 |
| **Right rostral anterior cingulate cortex** | 0.034 | 0.041 | [-0.047 - 0.114] | 0.766 | 0.414 | 0.681 | <0.001 | 7160 | 949 |
| **Left middle temporal gyrus** | 0.034 | 0.042 | [-0.048 - 0.116] | 0.507 | 0.416 | 0.681 | <0.001 | 7102 | 908 |
| **Left superior parietal cortex** | 0.035 | 0.041 | [-0.046 - 0.116] | 0.423 | 0.395 | 0.681 | <0.001 | 7158 | 947 |
| **Right insula** | 0.036 | 0.041 | [-0.045 - 0.117] | 0.490 | 0.385 | 0.681 | <0.001 | 7207 | 946 |
| **Right caudal middle frontal gyrus** | 0.039 | 0.041 | [-0.042 - 0.119] | 0.695 | 0.349 | 0.681 | <0.001 | 7185 | 948 |
| **Right superior frontal gyrus** | 0.040 | 0.041 | [-0.041 - 0.121] | 0.511 | 0.335 | 0.681 | <0.001 | 7154 | 947 |
| **Right supramarginal gyrus** | 0.040 | 0.056 | [-0.069 - 0.149] | 0.570 | 0.473 | 0.684 | 28.772 | 7144 | 924 |
| **Right postcentral gyrus** | 0.040 | 0.041 | [-0.041 - 0.121] | 0.479 | 0.333 | 0.681 | <0.001 | 7163 | 943 |
| **Right entorhinal cortex** | 0.041 | 0.042 | [-0.042 - 0.124] | 0.902 | 0.331 | 0.681 | <0.001 | 6982 | 911 |
| **Left postcentral gyrus** | 0.042 | 0.041 | [-0.039 - 0.123] | 0.489 | 0.310 | 0.681 | <0.001 | 7137 | 944 |
| **Right precuneus** | 0.043 | 0.041 | [-0.037 - 0.124] | 0.562 | 0.292 | 0.681 | <0.001 | 7203 | 949 |
| **Right lingual gyrus** | 0.044 | 0.062 | [-0.078 - 0.166] | 0.614 | 0.478 | 0.684 | 43.093 | 7216 | 949 |
| **Right paracentral lobule** | 0.044 | 0.041 | [-0.036 - 0.125] | 0.624 | 0.281 | 0.681 | <0.001 | 7159 | 948 |
| **Left insula** | 0.046 | 0.041 | [-0.034 - 0.127] | 0.524 | 0.260 | 0.681 | <0.001 | 7200 | 949 |
| **Left superior temporal gyrus** | 0.049 | 0.042 | [-0.034 - 0.132] | 0.613 | 0.245 | 0.681 | <0.001 | 7094 | 898 |
| **Left caudal middle frontal gyrus** | 0.049 | 0.060 | [-0.068 - 0.166] | 0.809 | 0.412 | 0.681 | 38.579 | 7171 | 949 |
| **Left precuneus** | 0.049 | 0.064 | [-0.077 - 0.175] | 0.625 | 0.445 | 0.681 | 46.201 | 7201 | 948 |
| **Left paracentral lobule** | 0.049 | 0.041 | [-0.032 - 0.13] | 0.690 | 0.234 | 0.681 | <0.001 | 7113 | 945 |
| **Right posterior cingulate cortex** | 0.051 | 0.080 | [-0.106 - 0.207] | 0.781 | 0.526 | 0.719 | 65.581 | 7213 | 951 |
| **Right middle temporal gyrus** | 0.053 | 0.041 | [-0.028 - 0.134] | 0.752 | 0.200 | 0.681 | <0.001 | 7159 | 943 |
| **Left pars orbitalis** | 0.055 | 0.070 | [-0.082 - 0.193] | 0.836 | 0.429 | 0.681 | 54.614 | 7213 | 951 |
| **Right precentral gyrus** | 0.058 | 0.041 | [-0.023 - 0.139] | 0.656 | 0.162 | 0.681 | <0.001 | 7157 | 948 |
| **Left superior frontal gyrus** | 0.059 | 0.041 | [-0.022 - 0.14] | 0.721 | 0.154 | 0.681 | <0.001 | 7120 | 946 |
| **Right hemisphere total surface area** | 0.059 | 0.041 | [-0.021 - 0.14] | 0.619 | 0.149 | 0.681 | <0.001 | 7223 | 951 |
| **Right pars orbitalis** | 0.060 | 0.041 | [-0.02 - 0.141] | 0.910 | 0.143 | 0.681 | <0.001 | 7215 | 950 |
| **Left lingual gyrus** | 0.061 | 0.085 | [-0.106 - 0.228] | 0.859 | 0.473 | 0.684 | 69.765 | 7205 | 951 |
| **Left rostral middle frontal gyrus** | 0.064 | 0.041 | [-0.016 - 0.145] | 0.905 | 0.118 | 0.681 | <0.001 | 7201 | 951 |
| **Left lateral orbitofrontal cortex** | 0.067 | 0.067 | [-0.065 - 0.199] | 0.994 | 0.322 | 0.681 | 51.099 | 7220 | 951 |
| **Left medial orbitofrontal cortex** | 0.069 | 0.050 | [-0.028 - 0.166] | 1.076 | 0.166 | 0.681 | 17.165 | 7132 | 945 |
| **Left posterior cingulate cortex** | 0.071 | 0.050 | [-0.027 - 0.169] | 1.048 | 0.155 | 0.681 | 18.585 | 7212 | 949 |
| **Right cuneus** | 0.071 | 0.041 | [-0.009 - 0.152] | 1.022 | 0.083 | 0.646 | <0.001 | 7201 | 946 |
| **Right rostral middle frontal gyrus** | 0.075 | 0.044 | [-0.012 - 0.162] | 1.058 | 0.092 | 0.646 | 6.513 | 7201 | 950 |
| **Left hemisphere total surface area** | 0.077 | 0.041 | [-0.004 - 0.157] | 0.799 | 0.062 | 0.542 | <0.001 | 7223 | 951 |
| **Right isthmus cingulate cortex** | 0.079 | 0.081 | [-0.081 - 0.238] | 1.268 | 0.335 | 0.681 | 66.834 | 7210 | 950 |
| **Left lateral occipital cortex** | 0.088 | 0.041 | [0.007 - 0.168] | 1.130 | 0.034 | 0.336 | <0.001 | 7198 | 948 |
| **Right lateral orbitofrontal cortex** | 0.089 | 0.072 | [-0.053 - 0.231] | 1.255 | 0.218 | 0.681 | 57.629 | 7220 | 951 |
| **Left temporal pole** | 0.093 | 0.041 | [0.011 - 0.174] | 1.477 | 0.025 | 0.296 | <0.001 | 7188 | 941 |
| **Right lateral occipital cortex** | 0.093 | 0.041 | [0.012 - 0.173] | 1.226 | 0.024 | 0.296 | <0.001 | 7207 | 949 |
| **Right frontal pole** | 0.094 | 0.080 | [-0.063 - 0.25] | 1.438 | 0.241 | 0.681 | 65.267 | 7218 | 951 |
| **Left precentral gyrus** | 0.095 | 0.120 | [-0.139 - 0.33] | 1.050 | 0.426 | 0.681 | 85.296 | 7155 | 947 |
| **Left supramarginal gyrus** | 0.097 | 0.042 | [0.015 - 0.179] | 1.414 | 0.020 | 0.296 | <0.001 | 7083 | 921 |
| **Left inferior temporal gyrus** | 0.099 | 0.041 | [0.017 - 0.18] | 1.629 | 0.017 | 0.296 | <0.001 | 7188 | 938 |
| **Right temporal pole** | 0.107 | 0.042 | [0.026 - 0.189] | 1.740 | 0.010 | 0.296 | <0.001 | 7140 | 935 |
| **Left isthmus cingulate cortex** | 0.153 | 0.068 | [0.02 - 0.286] | 2.585 | 0.024 | 0.296 | 51.379 | 7207 | 946 |

**^a^** Included Samples: CLING, Imaging Genetics Dublin, Clinical Depression Dublin, Sexpect, MMDP 3T, MPIP, Muenster Cohort, NESDA, Novosibirsk, SHIP, SHIP-trend, Sydney, Stanford, Rotterdam study.

AD: antidepressant using; MDD: Major Depressive Disorder; CTL: Controls.

**Supplementary Table S30**: Full meta-analytic results for surface area of each structure for MDD patients not taking antidepressants at time of scanning versus Controls comparison controlling for age, sex and scan center. Adjusted Cohen's d is reported.

|  | **Cohen's d ^a^** | **Std. Err.** | **95% CI** | **% Difference** | **P-value** | **FDR P-value** | **I^2^** | **# Controls** | **# Patients** |
| --- | --- | --- | --- | --- | --- | --- | --- | --- | --- |
|  | **(noAD MDD vs CTL)** |  |  |  |  |  |  |  |  |
| **Left pars triangularis** | -0.090 | 0.059 | [-0.206 - 0.026] | -0.548 | 0.128 | 0.997 | 41.796 | 7226 | 910 |
| **Left pars orbitalis** | -0.073 | 0.066 | [-0.202 - 0.056] | -0.534 | 0.266 | 0.997 | 52.630 | 7238 | 913 |
| **Left middle temporal gyrus** | -0.061 | 0.084 | [-0.226 - 0.104] | -0.363 | 0.467 | 0.997 | 70.028 | 7127 | 863 |
| **Left posterior cingulate cortex** | -0.055 | 0.040 | [-0.133 - 0.023] | -0.343 | 0.167 | 0.997 | <0.001 | 7236 | 912 |
| **Left frontal pole** | -0.054 | 0.040 | [-0.131 - 0.024] | -0.565 | 0.176 | 0.997 | <0.001 | 7248 | 915 |
| **Left banks superior temporal sulcus** | -0.051 | 0.074 | [-0.197 - 0.094] | -0.376 | 0.490 | 0.997 | 60.586 | 7089 | 847 |
| **Right inferior parietal cortex** | -0.044 | 0.076 | [-0.194 - 0.106] | -0.248 | 0.568 | 0.997 | 65.056 | 7177 | 898 |
| **Right frontal pole** | -0.043 | 0.040 | [-0.121 - 0.035] | -0.450 | 0.276 | 0.997 | <0.001 | 7244 | 914 |
| **Left pars opercularis** | -0.037 | 0.040 | [-0.116 - 0.041] | -0.178 | 0.348 | 0.997 | <0.001 | 7211 | 905 |
| **Right supramarginal gyrus** | -0.033 | 0.040 | [-0.112 - 0.045] | -0.172 | 0.405 | 0.997 | <0.001 | 7162 | 898 |
| **Right precuneus** | -0.032 | 0.047 | [-0.125 - 0.061] | -0.160 | 0.496 | 0.997 | 16.702 | 7226 | 910 |
| **Right parahippocampal gyrus** | -0.029 | 0.040 | [-0.107 - 0.049] | -0.315 | 0.470 | 0.997 | <0.001 | 7198 | 902 |
| **Left parahippocampal gyrus** | -0.028 | 0.070 | [-0.166 - 0.11] | -0.352 | 0.688 | 0.997 | 58.448 | 7185 | 899 |
| **Right precentral gyrus** | -0.027 | 0.075 | [-0.174 - 0.12] | -0.163 | 0.716 | 0.997 | 63.509 | 7180 | 897 |
| **Right lateral orbitofrontal cortex** | -0.026 | 0.052 | [-0.127 - 0.075] | -0.154 | 0.613 | 0.997 | 26.857 | 7245 | 916 |
| **Right caudal middle frontal gyrus** | -0.025 | 0.040 | [-0.104 - 0.053] | -0.134 | 0.523 | 0.997 | <0.001 | 7211 | 906 |
| **Right insula** | -0.023 | 0.044 | [-0.11 - 0.064] | -0.126 | 0.603 | 0.997 | 9.734 | 7237 | 908 |
| **Left entorhinal cortex** | -0.023 | 0.041 | [-0.103 - 0.057] | -0.261 | 0.579 | 0.997 | <0.001 | 7046 | 871 |
| **Left pericalcarine cortex** | -0.020 | 0.052 | [-0.122 - 0.083] | -0.137 | 0.709 | 0.997 | 28.176 | 7245 | 914 |
| **Left transverse temporal gyrus** | -0.019 | 0.061 | [-0.139 - 0.101] | -0.149 | 0.758 | 0.997 | 45.837 | 7246 | 913 |
| **Right hemisphere total surface area** | -0.018 | 0.051 | [-0.118 - 0.082] | -0.192 | 0.718 | 0.997 | 25.318 | 7249 | 916 |
| **Left precuneus** | -0.018 | 0.040 | [-0.096 - 0.06] | -0.086 | 0.652 | 0.997 | <0.001 | 7227 | 909 |
| **Right superior parietal cortex** | -0.017 | 0.048 | [-0.111 - 0.077] | -0.087 | 0.716 | 0.997 | 17.738 | 7201 | 900 |
| **Left postcentral gyrus** | -0.017 | 0.051 | [-0.117 - 0.084] | -0.092 | 0.746 | 0.997 | 25.082 | 7163 | 887 |
| **Left superior temporal gyrus** | -0.016 | 0.063 | [-0.14 - 0.107] | -0.101 | 0.793 | 0.997 | 44.962 | 7112 | 857 |
| **Left lateral occipital cortex** | -0.016 | 0.058 | [-0.128 - 0.097] | -0.089 | 0.787 | 0.997 | 38.982 | 7224 | 909 |
| **Right fusiform gyrus** | -0.015 | 0.045 | [-0.104 - 0.074] | -0.074 | 0.738 | 0.997 | 10.568 | 7114 | 881 |
| **Left insula** | -0.014 | 0.052 | [-0.115 - 0.087] | -0.074 | 0.782 | 0.997 | 26.406 | 7229 | 912 |
| **Right pars orbitalis** | -0.010 | 0.075 | [-0.157 - 0.137] | -0.073 | 0.893 | 0.997 | 63.948 | 7243 | 913 |
| **Right paracentral lobule** | -0.006 | 0.051 | [-0.106 - 0.095] | -0.031 | 0.914 | 0.997 | 25.115 | 7185 | 898 |
| **Left hemisphere total surface area** | -0.005 | 0.051 | [-0.104 - 0.094] | -0.052 | 0.921 | 0.997 | 24.584 | 7249 | 916 |
| **Right pars opercularis** | -0.003 | 0.060 | [-0.122 - 0.115] | -0.017 | 0.955 | 0.997 | 43.691 | 7212 | 900 |
| **Right inferior temporal gyrus** | -0.002 | 0.040 | [-0.081 - 0.076] | -0.013 | 0.952 | 0.997 | <0.001 | 7225 | 905 |
| **Left caudal middle frontal gyrus** | -1.36E-04 | 0.040 | [-0.079 - 0.078] | -6.91E-04 | 0.997 | 0.997 | <0.001 | 7196 | 901 |
| **Right temporal pole** | 5.09E-04 | 0.067 | [-0.131 - 0.132] | 0.006 | 0.994 | 0.997 | 53.126 | 7165 | 889 |
| **Left cuneus** | 6.16E-04 | 0.040 | [-0.078 - 0.079] | 0.004 | 0.988 | 0.997 | <0.001 | 7221 | 907 |
| **Left supramarginal gyrus** | 0.002 | 0.047 | [-0.09 - 0.093] | 0.009 | 0.970 | 0.997 | 13.364 | 7110 | 880 |
| **Left caudal anterior cingulate cortex** | 0.003 | 0.040 | [-0.076 - 0.081] | 0.032 | 0.944 | 0.997 | <0.001 | 7182 | 903 |
| **Left lateral orbitofrontal cortex** | 0.004 | 0.044 | [-0.083 - 0.091] | 0.023 | 0.933 | 0.997 | 9.826 | 7246 | 915 |
| **Left lingual gyrus** | 0.004 | 0.059 | [-0.111 - 0.119] | 0.023 | 0.947 | 0.997 | 40.898 | 7231 | 908 |
| **Right pars triangularis** | 0.005 | 0.040 | [-0.073 - 0.083] | 0.028 | 0.902 | 0.997 | <0.001 | 7217 | 907 |
| **Right pericalcarine cortex** | 0.006 | 0.049 | [-0.089 - 0.101] | 0.042 | 0.906 | 0.997 | 19.621 | 7243 | 915 |
| **Right posterior cingulate cortex** | 0.006 | 0.049 | [-0.089 - 0.102] | 0.043 | 0.896 | 0.997 | 20.282 | 7239 | 912 |
| **Right lingual gyrus** | 0.007 | 0.066 | [-0.122 - 0.136] | 0.039 | 0.920 | 0.997 | 52.886 | 7240 | 913 |
| **Left superior parietal cortex** | 0.007 | 0.040 | [-0.071 - 0.086] | 0.035 | 0.859 | 0.997 | <0.001 | 7183 | 895 |
| **Left paracentral lobule** | 0.008 | 0.085 | [-0.158 - 0.174] | 0.046 | 0.923 | 0.997 | 71.748 | 7138 | 885 |
| **Left rostral middle frontal gyrus** | 0.008 | 0.040 | [-0.07 - 0.086] | 0.041 | 0.832 | 0.997 | <0.001 | 7226 | 911 |
| **Left inferior parietal cortex** | 0.009 | 0.046 | [-0.082 - 0.099] | 0.050 | 0.849 | 0.997 | 13.084 | 7180 | 895 |
| **Right middle temporal gyrus** | 0.012 | 0.047 | [-0.081 - 0.105] | 0.068 | 0.797 | 0.997 | 15.510 | 7186 | 890 |
| **Right caudal anterior cingulate cortex** | 0.016 | 0.049 | [-0.079 - 0.112] | 0.183 | 0.735 | 0.997 | 19.629 | 7207 | 902 |
| **Right transverse temporal gyrus** | 0.017 | 0.040 | [-0.06 - 0.095] | 0.140 | 0.659 | 0.997 | <0.001 | 7247 | 915 |
| **Right entorhinal cortex** | 0.019 | 0.041 | [-0.062 - 0.1] | 0.222 | 0.650 | 0.997 | <0.001 | 7020 | 854 |
| **Left inferior temporal gyrus** | 0.019 | 0.055 | [-0.088 - 0.127] | 0.108 | 0.724 | 0.997 | 32.618 | 7220 | 900 |
| **Right isthmus cingulate cortex** | 0.020 | 0.040 | [-0.058 - 0.098] | 0.160 | 0.611 | 0.997 | <0.001 | 7235 | 911 |
| **Left isthmus cingulate cortex** | 0.021 | 0.040 | [-0.057 - 0.098] | 0.159 | 0.603 | 0.997 | <0.001 | 7232 | 912 |
| **Left medial orbitofrontal cortex** | 0.021 | 0.045 | [-0.067 - 0.109] | 0.164 | 0.637 | 0.997 | 10.058 | 7151 | 889 |
| **Right cuneus** | 0.024 | 0.040 | [-0.054 - 0.102] | 0.144 | 0.554 | 0.997 | <0.001 | 7226 | 910 |
| **Right banks superior temporal sulcus** | 0.025 | 0.055 | [-0.084 - 0.133] | 0.175 | 0.656 | 0.997 | 33.053 | 7163 | 893 |
| **Right postcentral gyrus** | 0.025 | 0.040 | [-0.053 - 0.104] | 0.143 | 0.528 | 0.997 | <0.001 | 7187 | 896 |
| **Right medial orbitofrontal cortex** | 0.031 | 0.044 | [-0.054 - 0.117] | 0.262 | 0.475 | 0.997 | 7.582 | 7201 | 898 |
| **Left fusiform gyrus** | 0.033 | 0.057 | [-0.079 - 0.144] | 0.160 | 0.567 | 0.997 | 36.583 | 7094 | 884 |
| **Right superior frontal gyrus** | 0.033 | 0.040 | [-0.046 - 0.112] | 0.160 | 0.412 | 0.997 | 0.299 | 7180 | 895 |
| **Left precentral gyrus** | 0.034 | 0.062 | [-0.088 - 0.155] | 0.194 | 0.586 | 0.997 | 46.484 | 7180 | 896 |
| **Right rostral anterior cingulate cortex** | 0.035 | 0.040 | [-0.044 - 0.114] | 0.356 | 0.383 | 0.997 | <0.001 | 7190 | 893 |
| **Left temporal pole** | 0.037 | 0.040 | [-0.042 - 0.115] | 0.369 | 0.361 | 0.997 | <0.001 | 7212 | 896 |
| **Left rostral anterior cingulate cortex** | 0.060 | 0.040 | [-0.019 - 0.139] | 0.587 | 0.136 | 0.997 | <0.001 | 7132 | 884 |
| **Right rostral middle frontal gyrus** | 0.061 | 0.040 | [-0.017 - 0.139] | 0.296 | 0.124 | 0.997 | <0.001 | 7227 | 905 |
| **Right lateral occipital cortex** | 0.062 | 0.046 | [-0.029 - 0.153] | 0.365 | 0.179 | 0.997 | 14.644 | 7233 | 913 |
| **Left superior frontal gyrus** | 0.064 | 0.040 | [-0.015 - 0.142] | 0.322 | 0.113 | 0.997 | <0.001 | 7146 | 898 |
| **Right superior temporal gyrus** | 0.064 | 0.077 | [-0.087 - 0.215] | 0.382 | 0.408 | 0.997 | 63.293 | 7142 | 872 |

**^a^** Included Samples: CODE, Imaging Genetics Dublin, Bipolar Family Study, Houston, MMDP 3T, Melbourne, MPIP, Muenster Cohort, NESDA, DepOx, QTIM, SHIP, SHIP-trend, Sydney, Stanford, Rotterdam study.

noAD: antidepressant free; MDD: Major Depressive Disorder; CTL: Controls.

**Supplementary Table S31**: Full meta-analytic results for surface area of each structure for MDD patients taking antidepressants MDD patients not taking antidepressants at time of scanning comparison controlling for age, sex and scan center. Adjusted Cohen's d is reported.

|  | **Cohen's d ^a^** | **<0.001** | **95% CI** | **% Difference** | **P-value** | **FDR P-value** | **I^2^** | **# AD MDD** | **# noAD MDD** |
| --- | --- | --- | --- | --- | --- | --- | --- | --- | --- |
|  | **(AD MDD vs no AD MDD)** |  |  |  |  |  |  |  |  |
| **Right rostral anterior cingulate cortex** | -0.130 | 0.066 | [-0.26 - -0.001] | -2.961 | 0.049 | 0.980 | 0.633 | 853 | 651 |
| **Right superior temporal gyrus** | -0.126 | 0.103 | [-0.328 - 0.076] | -1.481 | 0.221 | 0.980 | 50.939 | 811 | 639 |
| **Right posterior cingulate cortex** | -0.118 | 0.066 | [-0.246 - 0.011] | -1.814 | 0.073 | 0.980 | <0.001 | 853 | 671 |
| **Right transverse temporal gyrus** | -0.118 | 0.066 | [-0.246 - 0.011] | -1.950 | 0.073 | 0.980 | <0.001 | 853 | 673 |
| **Right superior frontal gyrus** | -0.112 | 0.120 | [-0.347 - 0.123] | -1.437 | 0.349 | 0.980 | 65.966 | 849 | 653 |
| **Left inferior parietal cortex** | -0.105 | 0.094 | [-0.288 - 0.079] | -1.501 | 0.263 | 0.980 | 44.595 | 848 | 655 |
| **Right caudal anterior cingulate cortex** | -0.104 | 0.066 | [-0.233 - 0.026] | -2.009 | 0.117 | 0.980 | <0.001 | 850 | 661 |
| **Left paracentral lobule** | -0.093 | 0.066 | [-0.223 - 0.037] | -1.304 | 0.161 | 0.980 | <0.001 | 847 | 643 |
| **Left frontal pole** | -0.089 | 0.096 | [-0.278 - 0.1] | -1.383 | 0.357 | 0.980 | 48.406 | 852 | 673 |
| **Left fusiform gyrus** | -0.084 | 0.094 | [-0.268 - 0.1] | -1.219 | 0.370 | 0.980 | 44.374 | 846 | 641 |
| **Right inferior parietal cortex** | -0.080 | 0.146 | [-0.365 - 0.206] | -1.106 | 0.583 | 0.980 | 77.135 | 846 | 655 |
| **Right banks superior temporal sulcus** | -0.075 | 0.083 | [-0.238 - 0.088] | -1.176 | 0.368 | 0.980 | 30.563 | 794 | 655 |
| **Left superior frontal gyrus** | -0.073 | 0.066 | [-0.202 - 0.057] | -0.892 | 0.271 | 0.980 | <0.001 | 849 | 655 |
| **Left parahippocampal gyrus** | -0.064 | 0.101 | [-0.262 - 0.135] | -1.359 | 0.529 | 0.980 | 52.702 | 848 | 659 |
| **Left pericalcarine cortex** | -0.061 | 0.108 | [-0.273 - 0.15] | -1.062 | 0.570 | 0.980 | 58.763 | 853 | 671 |
| **Left precentral gyrus** | -0.059 | 0.096 | [-0.246 - 0.128] | -0.648 | 0.538 | 0.980 | 46.835 | 849 | 657 |
| **Left superior parietal cortex** | -0.059 | 0.066 | [-0.188 - 0.071] | -0.708 | 0.373 | 0.980 | <0.001 | 850 | 654 |
| **Left rostral anterior cingulate cortex** | -0.054 | 0.066 | [-0.184 - 0.076] | -1.192 | 0.414 | 0.980 | <0.001 | 848 | 641 |
| **Right superior parietal cortex** | -0.053 | 0.067 | [-0.183 - 0.078] | -0.616 | 0.428 | 0.980 | <0.001 | 844 | 659 |
| **Right postcentral gyrus** | -0.051 | 0.091 | [-0.229 - 0.127] | -0.610 | 0.575 | 0.980 | 41.107 | 845 | 654 |
| **Left medial orbitofrontal cortex** | -0.051 | 0.066 | [-0.18 - 0.078] | -0.799 | 0.440 | 0.980 | <0.001 | 850 | 647 |
| **Right entorhinal cortex** | -0.051 | 0.074 | [-0.196 - 0.094] | -1.117 | 0.493 | 0.980 | 12.721 | 832 | 612 |
| **Right fusiform gyrus** | -0.049 | 0.079 | [-0.203 - 0.105] | -0.725 | 0.536 | 0.980 | 23.533 | 847 | 638 |
| **Left lateral orbitofrontal cortex** | -0.046 | 0.066 | [-0.174 - 0.083] | -0.681 | 0.486 | 0.980 | <0.001 | 853 | 672 |
| **Right rostral middle frontal gyrus** | -0.042 | 0.066 | [-0.171 - 0.087] | -0.600 | 0.520 | 0.980 | <0.001 | 852 | 662 |
| **Left entorhinal cortex** | -0.040 | 0.067 | [-0.172 - 0.091] | -0.898 | 0.547 | 0.980 | <0.001 | 840 | 632 |
| **Right medial orbitofrontal cortex** | -0.040 | 0.066 | [-0.169 - 0.089] | -0.615 | 0.543 | 0.980 | <0.001 | 853 | 656 |
| **Right pericalcarine cortex** | -0.038 | 0.071 | [-0.177 - 0.101] | -0.638 | 0.590 | 0.980 | 10.750 | 853 | 672 |
| **Right paracentral lobule** | -0.033 | 0.066 | [-0.163 - 0.096] | -0.469 | 0.613 | 0.980 | <0.001 | 850 | 655 |
| **Left pars opercularis** | -0.030 | 0.153 | [-0.329 - 0.269] | -0.484 | 0.844 | 0.980 | 79.446 | 852 | 663 |
| **Left middle temporal gyrus** | -0.028 | 0.123 | [-0.269 - 0.213] | -0.417 | 0.820 | 0.980 | 66.528 | 819 | 632 |
| **Left lingual gyrus** | -0.027 | 0.089 | [-0.202 - 0.148] | -0.383 | 0.761 | 0.980 | 40.502 | 853 | 666 |
| **Right caudal middle frontal gyrus** | -0.027 | 0.066 | [-0.156 - 0.102] | -0.484 | 0.683 | 0.980 | <0.001 | 851 | 663 |
| **Left caudal anterior cingulate cortex** | -0.027 | 0.066 | [-0.156 - 0.103] | -0.502 | 0.687 | 0.980 | <0.001 | 848 | 660 |
| **Left cuneus** | -0.025 | 0.138 | [-0.296 - 0.246] | -0.376 | 0.856 | 0.980 | 74.973 | 853 | 664 |
| **Left banks superior temporal sulcus** | -0.020 | 0.088 | [-0.193 - 0.153] | -0.347 | 0.820 | 0.980 | 34.566 | 782 | 622 |
| **Left precuneus** | -0.020 | 0.081 | [-0.179 - 0.139] | -0.253 | 0.806 | 0.980 | 28.242 | 851 | 669 |
| **Left caudal middle frontal gyrus** | -0.019 | 0.066 | [-0.148 - 0.11] | -0.315 | 0.772 | 0.980 | <0.001 | 851 | 658 |
| **Right lingual gyrus** | -0.018 | 0.085 | [-0.184 - 0.148] | -0.254 | 0.829 | 0.980 | 34.130 | 853 | 670 |
| **Right inferior temporal gyrus** | -0.015 | 0.066 | [-0.145 - 0.114] | -0.257 | 0.818 | 0.980 | <0.001 | 849 | 665 |
| **Left postcentral gyrus** | -0.013 | 0.066 | [-0.143 - 0.117] | -0.148 | 0.847 | 0.980 | <0.001 | 848 | 645 |
| **Right pars orbitalis** | -0.012 | 0.087 | [-0.181 - 0.158] | -0.177 | 0.892 | 0.980 | 36.911 | 853 | 671 |
| **Left transverse temporal gyrus** | -0.009 | 0.096 | [-0.197 - 0.179] | -0.149 | 0.925 | 0.980 | 47.891 | 853 | 671 |
| **Left inferior temporal gyrus** | -0.008 | 0.094 | [-0.192 - 0.175] | -0.137 | 0.929 | 0.980 | 43.773 | 845 | 660 |
| **Right lateral occipital cortex** | -0.008 | 0.090 | [-0.184 - 0.168] | -0.101 | 0.932 | 0.980 | 40.669 | 851 | 670 |
| **Left insula** | -0.007 | 0.066 | [-0.136 - 0.121] | -0.085 | 0.909 | 0.980 | <0.001 | 853 | 671 |
| **Left rostral middle frontal gyrus** | -0.002 | 0.066 | [-0.131 - 0.127] | -0.029 | 0.975 | 0.980 | <0.001 | 853 | 668 |
| **Left pars triangularis** | 0.003 | 0.124 | [-0.239 - 0.245] | 0.048 | 0.980 | 0.980 | 68.584 | 853 | 668 |
| **Right middle temporal gyrus** | 0.005 | 0.099 | [-0.189 - 0.199] | 0.071 | 0.960 | 0.980 | 50.174 | 846 | 649 |
| **Right lateral orbitofrontal cortex** | 0.006 | 0.069 | [-0.128 - 0.141] | 0.091 | 0.925 | 0.980 | 6.195 | 853 | 673 |
| **Right cuneus** | 0.007 | 0.109 | [-0.207 - 0.22] | 0.094 | 0.952 | 0.980 | 59.342 | 851 | 669 |
| **Right hemisphere total surface area** | 0.008 | 0.100 | [-0.188 - 0.204] | 0.086 | 0.934 | 0.980 | 51.878 | 853 | 673 |
| **Right isthmus cingulate cortex** | 0.012 | 0.075 | [-0.135 - 0.159] | 0.193 | 0.873 | 0.980 | 18.471 | 853 | 670 |
| **Left hemisphere total surface area** | 0.017 | 0.089 | [-0.157 - 0.192] | 0.179 | 0.847 | 0.980 | 39.989 | 853 | 673 |
| **Right temporal pole** | 0.017 | 0.083 | [-0.146 - 0.18] | 0.280 | 0.836 | 0.980 | 28.966 | 837 | 650 |
| **Left superior temporal gyrus** | 0.019 | 0.089 | [-0.154 - 0.193] | 0.243 | 0.826 | 0.980 | 34.948 | 810 | 632 |
| **Right precuneus** | 0.020 | 0.102 | [-0.18 - 0.22] | 0.260 | 0.844 | 0.980 | 53.802 | 853 | 669 |
| **Left supramarginal gyrus** | 0.024 | 0.097 | [-0.166 - 0.214] | 0.347 | 0.806 | 0.980 | 47.711 | 834 | 643 |
| **Left isthmus cingulate cortex** | 0.025 | 0.066 | [-0.104 - 0.154] | 0.424 | 0.704 | 0.980 | <0.001 | 850 | 671 |
| **Right precentral gyrus** | 0.026 | 0.113 | [-0.196 - 0.248] | 0.296 | 0.818 | 0.980 | 62.209 | 851 | 658 |
| **Right supramarginal gyrus** | 0.027 | 0.067 | [-0.104 - 0.158] | 0.386 | 0.686 | 0.980 | <0.001 | 828 | 660 |
| **Right pars opercularis** | 0.033 | 0.066 | [-0.095 - 0.162] | 0.536 | 0.611 | 0.980 | <0.001 | 853 | 659 |
| **Right pars triangularis** | 0.036 | 0.066 | [-0.093 - 0.164] | 0.576 | 0.589 | 0.980 | <0.001 | 853 | 665 |
| **Right parahippocampal gyrus** | 0.036 | 0.115 | [-0.191 - 0.262] | 0.584 | 0.757 | 0.980 | 63.496 | 848 | 660 |
| **Left pars orbitalis** | 0.045 | 0.066 | [-0.084 - 0.174] | 0.678 | 0.494 | 0.980 | <0.001 | 853 | 672 |
| **Left temporal pole** | 0.047 | 0.081 | [-0.111 - 0.205] | 0.745 | 0.562 | 0.980 | 25.913 | 843 | 656 |
| **Left lateral occipital cortex** | 0.056 | 0.076 | [-0.093 - 0.205] | 0.721 | 0.462 | 0.980 | 20.095 | 851 | 666 |
| **Right insula** | 0.066 | 0.066 | [-0.062 - 0.195] | 0.905 | 0.313 | 0.980 | <0.001 | 853 | 667 |
| **Left posterior cingulate cortex** | 0.081 | 0.110 | [-0.135 - 0.296] | 1.187 | 0.464 | 0.980 | 60.091 | 851 | 670 |
| **Right frontal pole** | 0.100 | 0.104 | [-0.104 - 0.303] | 1.530 | 0.338 | 0.980 | 55.343 | 853 | 671 |

**^a^** Included Samples: Imaging Genetics Dublin, MMDP 3T, MPIP, Muenster Cohort, NESDA, SHIP, SHIP-trend, Sydney, Stanford, Rotterdam study.

AD: antidepressant using; noAD: antidepressant free; MDD: Major Depressive Disorder.

**Supplementary Table S32**: Full meta-analytic results for surface area of each structure associated with severity of symptoms at study inclusion measured by the HDRS-17 controlling for age, sex and scan center. Adjusted Cohen's d is reported.

|  | **Pearson's r ^a^** | **Std. Err.** | **95% CI** | **% Difference** | **P-value** | **FDR P-value** | **I^2^** | **# Patients** |
| --- | --- | --- | --- | --- | --- | --- | --- | --- |
|  | **(HDRS-17)** |  |  |  |  |  |  |  |
| **Left insula** | -0.053 | 0.036 | [-0.123 - 0.017] | -0.551 | 0.139 | 0.972 | <0.001 | 774 |
| **Right pars opercularis** | -0.042 | 0.052 | [-0.144 - 0.06] | -0.423 | 0.419 | 0.972 | 42.883 | 776 |
| **Right caudal middle frontal gyrus** | -0.037 | 0.036 | [-0.107 - 0.033] | -0.388 | 0.302 | 0.972 | <0.001 | 776 |
| **Right paracentral lobule** | -0.035 | 0.036 | [-0.106 - 0.035] | -0.400 | 0.327 | 0.972 | <0.001 | 776 |
| **Right transverse temporal gyrus** | -0.032 | 0.036 | [-0.102 - 0.038] | -0.519 | 0.368 | 0.972 | <0.001 | 776 |
| **Left frontal pole** | -0.024 | 0.036 | [-0.094 - 0.045] | -0.514 | 0.494 | 0.972 | <0.001 | 776 |
| **Right superior temporal gyrus** | -0.022 | 0.076 | [-0.17 - 0.127] | -0.260 | 0.774 | 0.972 | 72.608 | 756 |
| **Left pars orbitalis** | -0.021 | 0.036 | [-0.092 - 0.049] | -0.312 | 0.552 | 0.972 | <0.001 | 776 |
| **Right medial orbitofrontal cortex** | -0.020 | 0.036 | [-0.09 - 0.051] | -0.333 | 0.581 | 0.972 | <0.001 | 775 |
| **Right banks superior temporal sulcus** | -0.018 | 0.037 | [-0.09 - 0.054] | -0.258 | 0.623 | 0.972 | <0.001 | 741 |
| **Right lateral orbitofrontal cortex** | -0.016 | 0.036 | [-0.086 - 0.055] | -0.187 | 0.660 | 0.972 | <0.001 | 776 |
| **Left inferior temporal gyrus** | -0.015 | 0.046 | [-0.104 - 0.074] | -0.169 | 0.740 | 0.972 | 26.588 | 773 |
| **Left posterior cingulate cortex** | -0.005 | 0.058 | [-0.118 - 0.108] | -0.063 | 0.930 | 0.972 | 52.357 | 776 |
| **Right inferior parietal cortex** | -0.005 | 0.048 | [-0.099 - 0.09] | -0.052 | 0.924 | 0.972 | 33.477 | 775 |
| **Right entorhinal cortex** | -0.004 | 0.048 | [-0.098 - 0.089] | -0.106 | 0.926 | 0.972 | 31.380 | 756 |
| **Left inferior parietal cortex** | -0.004 | 0.070 | [-0.142 - 0.133] | -0.049 | 0.951 | 0.972 | 69.019 | 774 |
| **Left pars opercularis** | -0.003 | 0.042 | [-0.085 - 0.079] | -0.033 | 0.934 | 0.972 | 17.687 | 775 |
| **Right posterior cingulate cortex** | -0.003 | 0.036 | [-0.073 - 0.068] | -0.034 | 0.943 | 0.972 | <0.001 | 776 |
| **Right parahippocampal gyrus** | 0.002 | 0.057 | [-0.11 - 0.113] | 0.033 | 0.979 | 0.979 | 51.035 | 773 |
| **Right middle temporal gyrus** | 0.002 | 0.036 | [-0.068 - 0.072] | 0.021 | 0.958 | 0.972 | <0.001 | 772 |
| **Left precentral gyrus** | 0.004 | 0.036 | [-0.066 - 0.074] | 0.045 | 0.913 | 0.972 | <0.001 | 776 |
| **Right superior parietal cortex** | 0.006 | 0.036 | [-0.064 - 0.076] | 0.060 | 0.867 | 0.972 | <0.001 | 772 |
| **Left caudal middle frontal gyrus** | 0.006 | 0.036 | [-0.063 - 0.076] | 0.066 | 0.856 | 0.972 | <0.001 | 776 |
| **Right rostral anterior cingulate cortex** | 0.006 | 0.045 | [-0.081 - 0.094] | 0.131 | 0.885 | 0.972 | 24.214 | 774 |
| **Right fusiform gyrus** | 0.007 | 0.048 | [-0.087 - 0.1] | 0.065 | 0.889 | 0.972 | 32.328 | 776 |
| **Right pericalcarine cortex** | 0.008 | 0.057 | [-0.105 - 0.12] | 0.110 | 0.896 | 0.972 | 52.158 | 772 |
| **Left middle temporal gyrus** | 0.009 | 0.037 | [-0.063 - 0.081] | 0.103 | 0.813 | 0.972 | <0.001 | 738 |
| **Left lateral orbitofrontal cortex** | 0.009 | 0.036 | [-0.062 - 0.079] | 0.106 | 0.807 | 0.972 | <0.001 | 776 |
| **Right postcentral gyrus** | 0.011 | 0.036 | [-0.059 - 0.081] | 0.125 | 0.756 | 0.972 | <0.001 | 776 |
| **Right precentral gyrus** | 0.011 | 0.036 | [-0.059 - 0.082] | 0.133 | 0.756 | 0.972 | <0.001 | 775 |
| **Right supramarginal gyrus** | 0.012 | 0.036 | [-0.059 - 0.083] | 0.121 | 0.745 | 0.972 | <0.001 | 760 |
| **Left medial orbitofrontal cortex** | 0.012 | 0.036 | [-0.058 - 0.083] | 0.188 | 0.735 | 0.972 | <0.001 | 773 |
| **Right pars orbitalis** | 0.012 | 0.051 | [-0.087 - 0.111] | 0.178 | 0.807 | 0.972 | 38.542 | 775 |
| **Right precuneus** | 0.013 | 0.048 | [-0.081 - 0.106] | 0.125 | 0.791 | 0.972 | 30.927 | 774 |
| **Right cuneus** | 0.013 | 0.036 | [-0.057 - 0.082] | 0.155 | 0.723 | 0.972 | <0.001 | 773 |
| **Left isthmus cingulate cortex** | 0.014 | 0.036 | [-0.056 - 0.083] | 0.208 | 0.705 | 0.972 | <0.001 | 774 |
| **Left postcentral gyrus** | 0.017 | 0.038 | [-0.058 - 0.093] | 0.193 | 0.649 | 0.972 | 7.142 | 774 |
| **Right hemisphere total surface area** | 0.018 | 0.036 | [-0.052 - 0.088] | 0.383 | 0.608 | 0.972 | <0.001 | 776 |
| **Left precuneus** | 0.020 | 0.036 | [-0.05 - 0.09] | 0.190 | 0.578 | 0.972 | <0.001 | 775 |
| **Left superior frontal gyrus** | 0.020 | 0.036 | [-0.05 - 0.091] | 0.206 | 0.570 | 0.972 | <0.001 | 775 |
| **Left superior parietal cortex** | 0.021 | 0.036 | [-0.049 - 0.091] | 0.206 | 0.557 | 0.972 | <0.001 | 775 |
| **Right lateral occipital cortex** | 0.022 | 0.043 | [-0.063 - 0.107] | 0.255 | 0.617 | 0.972 | 21.195 | 776 |
| **Left supramarginal gyrus** | 0.024 | 0.036 | [-0.047 - 0.094] | 0.253 | 0.515 | 0.972 | <0.001 | 760 |
| **Left entorhinal cortex** | 0.024 | 0.036 | [-0.047 - 0.095] | 0.552 | 0.508 | 0.972 | <0.001 | 756 |
| **Left parahippocampal gyrus** | 0.027 | 0.068 | [-0.106 - 0.16] | 0.680 | 0.688 | 0.972 | 66.240 | 775 |
| **Right superior frontal gyrus** | 0.028 | 0.036 | [-0.043 - 0.099] | 0.271 | 0.438 | 0.972 | <0.001 | 776 |
| **Left hemisphere total surface area** | 0.029 | 0.036 | [-0.041 - 0.1] | 0.613 | 0.411 | 0.972 | <0.001 | 776 |
| **Left lingual gyrus** | 0.030 | 0.040 | [-0.049 - 0.109] | 0.347 | 0.457 | 0.972 | 12.703 | 776 |
| **Left rostral anterior cingulate cortex** | 0.033 | 0.036 | [-0.038 - 0.103] | 0.639 | 0.361 | 0.972 | <0.001 | 772 |
| **Right caudal anterior cingulate cortex** | 0.033 | 0.056 | [-0.077 - 0.143] | 0.735 | 0.557 | 0.972 | 49.523 | 774 |
| **Left paracentral lobule** | 0.035 | 0.036 | [-0.036 - 0.105] | 0.393 | 0.336 | 0.972 | <0.001 | 776 |
| **Left pars triangularis** | 0.037 | 0.040 | [-0.041 - 0.114] | 0.449 | 0.351 | 0.972 | 11.421 | 774 |
| **Left banks superior temporal sulcus** | 0.039 | 0.037 | [-0.035 - 0.112] | 0.565 | 0.304 | 0.972 | <0.001 | 710 |
| **Left fusiform gyrus** | 0.040 | 0.063 | [-0.084 - 0.164] | 0.392 | 0.529 | 0.972 | 61.309 | 776 |
| **Left superior temporal gyrus** | 0.041 | 0.037 | [-0.031 - 0.113] | 0.500 | 0.266 | 0.972 | <0.001 | 740 |
| **Right insula** | 0.041 | 0.036 | [-0.029 - 0.111] | 0.446 | 0.254 | 0.972 | <0.001 | 771 |
| **Left transverse temporal gyrus** | 0.043 | 0.044 | [-0.043 - 0.129] | 0.680 | 0.324 | 0.972 | 22.011 | 776 |
| **Right lingual gyrus** | 0.043 | 0.038 | [-0.032 - 0.119] | 0.503 | 0.259 | 0.972 | 7.648 | 774 |
| **Left pericalcarine cortex** | 0.046 | 0.036 | [-0.024 - 0.116] | 0.642 | 0.201 | 0.972 | <0.001 | 772 |
| **Left rostral middle frontal gyrus** | 0.050 | 0.047 | [-0.042 - 0.142] | 0.488 | 0.284 | 0.972 | 30.866 | 776 |
| **Right inferior temporal gyrus** | 0.051 | 0.062 | [-0.071 - 0.173] | 0.558 | 0.409 | 0.972 | 60.133 | 776 |
| **Right frontal pole** | 0.051 | 0.067 | [-0.079 - 0.182] | 1.076 | 0.439 | 0.972 | 64.742 | 776 |
| **Left lateral occipital cortex** | 0.052 | 0.070 | [-0.085 - 0.189] | 0.598 | 0.458 | 0.972 | 68.435 | 775 |
| **Left caudal anterior cingulate cortex** | 0.062 | 0.061 | [-0.057 - 0.181] | 1.410 | 0.307 | 0.972 | 57.104 | 775 |
| **Left cuneus** | 0.079 | 0.083 | [-0.083 - 0.241] | 0.982 | 0.338 | 0.972 | 78.980 | 772 |
| **Right isthmus cingulate cortex** | 0.080 | 0.059 | [-0.035 - 0.196] | 1.275 | 0.173 | 0.972 | 55.195 | 775 |
| **Right rostral middle frontal gyrus** | 0.082 | 0.067 | [-0.049 - 0.213] | 0.793 | 0.220 | 0.972 | 65.742 | 776 |
| **Right pars triangularis** | 0.087 | 0.078 | [-0.066 - 0.24] | 1.004 | 0.267 | 0.972 | 75.660 | 775 |
| **Left temporal pole** | 0.088 | 0.036 | [0.018 - 0.158] | 1.787 | 0.013 | 0.933 | <0.001 | 776 |
| **Right temporal pole** | 0.113 | 0.064 | [-0.012 - 0.237] | 2.500 | 0.076 | 0.972 | 62.761 | 776 |

**^a^** Included Samples: CLING, Imaging Genetics Dublin, Clinical Depression Dublin, Bipolar Family Study, Houston, Sexpect, MMDP 3T, MPIP, Muenster Cohort, DepOx, Sydney.

HDRS-17: Hamilton Depression Rating Scale with 17 items.

**Supplementary Table S33**: Full meta-analytic results for surface area of each structure associated with severity of symptoms at study inclusion measured by the BDI-II controlling for age, sex and scan center. Adjusted Cohen's d is reported.

|  | **Pearson's r ^a^** | **Std. Err.** | **95% CI** | **% Difference** | **P-value** | **FDR P-value** | **I^2^** | **# Patients** |
| --- | --- | --- | --- | --- | --- | --- | --- | --- |
|  | **(BDI-II)** |  |  |  |  |  |  |  |
| **Left rostral anterior cingulate cortex** | -0.159 | 0.105 | [-0.365 - 0.046] | -3.144 | 0.129 | 0.312 | 89.443 | 903 |
| **Left superior parietal cortex** | -0.159 | 0.081 | [-0.317 - 0] | -1.575 | 0.050 | 0.184 | 80.098 | 920 |
| **Right postcentral gyrus** | -0.155 | 0.068 | [-0.289 - -0.022] | -1.781 | 0.023 | 0.107 | 70.571 | 916 |
| **Left supramarginal gyrus** | -0.125 | 0.092 | [-0.306 - 0.056] | -1.360 | 0.175 | 0.360 | 83.967 | 897 |
| **Left medial orbitofrontal cortex** | -0.125 | 0.044 | [-0.212 - -0.038] | -1.951 | 0.005 | 0.058 | 32.669 | 911 |
| **Left precuneus** | -0.123 | 0.037 | [-0.196 - -0.051] | -1.188 | 8.21E-04 | 0.020 | 14.863 | 937 |
| **Right inferior temporal gyrus** | -0.120 | 0.070 | [-0.258 - 0.017] | -1.314 | 0.087 | 0.233 | 71.729 | 933 |
| **Left frontal pole** | -0.113 | 0.032 | [-0.176 - -0.05] | -2.400 | 4.33E-04 | 0.020 | <0.001 | 942 |
| **Left postcentral gyrus** | -0.106 | 0.033 | [-0.17 - -0.042] | -1.177 | 0.001 | 0.020 | <0.001 | 909 |
| **Right precuneus** | -0.105 | 0.032 | [-0.168 - -0.042] | -1.051 | 0.001 | 0.020 | <0.001 | 937 |
| **Right hemisphere total surface area** | -0.094 | 0.032 | [-0.157 - -0.031] | -1.971 | 0.003 | 0.048 | <0.001 | 943 |
| **Right paracentral lobule** | -0.092 | 0.050 | [-0.19 - 0.005] | -1.050 | 0.064 | 0.204 | 44.599 | 922 |
| **Left fusiform gyrus** | -0.092 | 0.065 | [-0.218 - 0.035] | -0.903 | 0.157 | 0.355 | 65.808 | 906 |
| **Left hemisphere total surface area** | -0.086 | 0.032 | [-0.149 - -0.023] | -1.791 | 0.008 | 0.076 | <0.001 | 943 |
| **Right superior parietal cortex** | -0.084 | 0.033 | [-0.149 - -0.02] | -0.846 | 0.010 | 0.088 | <0.001 | 917 |
| **Left caudal middle frontal gyrus** | -0.083 | 0.054 | [-0.188 - 0.023] | -0.847 | 0.123 | 0.308 | 50.987 | 926 |
| **Right supramarginal gyrus** | -0.082 | 0.033 | [-0.146 - -0.018] | -0.850 | 0.011 | 0.089 | 0.109 | 921 |
| **Right superior frontal gyrus** | -0.079 | 0.033 | [-0.143 - -0.015] | -0.771 | 0.015 | 0.105 | <0.001 | 919 |
| **Right inferior parietal cortex** | -0.078 | 0.033 | [-0.142 - -0.014] | -0.881 | 0.018 | 0.105 | <0.001 | 919 |
| **Left superior frontal gyrus** | -0.077 | 0.033 | [-0.141 - -0.013] | -0.780 | 0.018 | 0.105 | <0.001 | 921 |
| **Left middle temporal gyrus** | -0.076 | 0.063 | [-0.2 - 0.048] | -0.909 | 0.227 | 0.425 | 63.089 | 902 |
| **Left insula** | -0.075 | 0.032 | [-0.139 - -0.012] | -0.789 | 0.020 | 0.107 | <0.001 | 940 |
| **Left inferior temporal gyrus** | -0.075 | 0.088 | [-0.248 - 0.099] | -0.835 | 0.399 | 0.618 | 82.446 | 927 |
| **Right middle temporal gyrus** | -0.075 | 0.033 | [-0.139 - -0.01] | -0.834 | 0.023 | 0.107 | <0.001 | 916 |
| **Right fusiform gyrus** | -0.074 | 0.050 | [-0.171 - 0.023] | -0.722 | 0.137 | 0.319 | 43.711 | 904 |
| **Right rostral middle frontal gyrus** | -0.070 | 0.051 | [-0.17 - 0.03] | -0.675 | 0.171 | 0.360 | 46.691 | 931 |
| **Left rostral middle frontal gyrus** | -0.069 | 0.052 | [-0.17 - 0.032] | -0.670 | 0.182 | 0.365 | 48.690 | 938 |
| **Right superior temporal gyrus** | -0.068 | 0.033 | [-0.134 - -0.003] | -0.822 | 0.039 | 0.171 | <0.001 | 895 |
| **Left precentral gyrus** | -0.068 | 0.055 | [-0.176 - 0.04] | -0.785 | 0.217 | 0.421 | 53.984 | 923 |
| **Right lingual gyrus** | -0.066 | 0.079 | [-0.22 - 0.088] | -0.769 | 0.399 | 0.618 | 77.792 | 938 |
| **Right cuneus** | -0.066 | 0.090 | [-0.243 - 0.111] | -0.810 | 0.466 | 0.645 | 83.660 | 934 |
| **Right precentral gyrus** | -0.065 | 0.033 | [-0.129 - -0.001] | -0.775 | 0.046 | 0.184 | <0.001 | 926 |
| **Left inferior parietal cortex** | -0.064 | 0.033 | [-0.128 - 0] | -0.728 | 0.050 | 0.184 | <0.001 | 923 |
| **Right lateral occipital cortex** | -0.061 | 0.032 | [-0.125 - 0.002] | -0.720 | 0.057 | 0.200 | <0.001 | 938 |
| **Left pars opercularis** | -0.061 | 0.033 | [-0.125 - 0.003] | -0.583 | 0.061 | 0.204 | <0.001 | 931 |
| **Left superior temporal gyrus** | -0.061 | 0.033 | [-0.126 - 0.005] | -0.743 | 0.070 | 0.212 | <0.001 | 889 |
| **Right caudal middle frontal gyrus** | -0.058 | 0.033 | [-0.122 - 0.006] | -0.616 | 0.074 | 0.215 | <0.001 | 931 |
| **Right rostral anterior cingulate cortex** | -0.057 | 0.049 | [-0.154 - 0.039] | -1.166 | 0.244 | 0.436 | 42.037 | 919 |
| **Left transverse temporal gyrus** | -0.056 | 0.032 | [-0.119 - 0.008] | -0.880 | 0.085 | 0.233 | <0.001 | 941 |
| **Left lingual gyrus** | -0.055 | 0.032 | [-0.119 - 0.009] | -0.638 | 0.090 | 0.233 | <0.001 | 936 |
| **Left caudal anterior cingulate cortex** | -0.052 | 0.063 | [-0.175 - 0.071] | -1.183 | 0.406 | 0.618 | 63.154 | 924 |
| **Right temporal pole** | -0.046 | 0.033 | [-0.111 - 0.019] | -1.016 | 0.162 | 0.355 | <0.001 | 910 |
| **Right insula** | -0.045 | 0.070 | [-0.183 - 0.093] | -0.495 | 0.519 | 0.673 | 70.890 | 933 |
| **Right entorhinal cortex** | -0.044 | 0.050 | [-0.142 - 0.055] | -1.039 | 0.386 | 0.618 | 42.845 | 850 |
| **Left parahippocampal gyrus** | -0.043 | 0.056 | [-0.153 - 0.066] | -1.080 | 0.438 | 0.625 | 54.356 | 924 |
| **Left temporal pole** | -0.041 | 0.049 | [-0.137 - 0.055] | -0.831 | 0.401 | 0.618 | 41.187 | 922 |
| **Right pericalcarine cortex** | -0.040 | 0.084 | [-0.204 - 0.124] | -0.582 | 0.634 | 0.745 | 80.589 | 940 |
| **Right banks superior temporal sulcus** | -0.040 | 0.033 | [-0.105 - 0.025] | -0.567 | 0.231 | 0.425 | <0.001 | 906 |
| **Left lateral occipital cortex** | -0.037 | 0.032 | [-0.101 - 0.026] | -0.431 | 0.249 | 0.436 | <0.001 | 934 |
| **Right pars orbitalis** | -0.035 | 0.051 | [-0.136 - 0.066] | -0.508 | 0.494 | 0.652 | 47.520 | 940 |
| **Right medial orbitofrontal cortex** | -0.031 | 0.044 | [-0.118 - 0.055] | -0.525 | 0.479 | 0.645 | 31.199 | 925 |
| **Right parahippocampal gyrus** | -0.031 | 0.053 | [-0.135 - 0.074] | -0.672 | 0.564 | 0.693 | 50.083 | 923 |
| **Right posterior cingulate cortex** | -0.030 | 0.032 | [-0.094 - 0.033] | -0.407 | 0.349 | 0.595 | <0.001 | 941 |
| **Left entorhinal cortex** | -0.029 | 0.053 | [-0.133 - 0.074] | -0.670 | 0.581 | 0.701 | 49.196 | 877 |
| **Left lateral orbitofrontal cortex** | -0.027 | 0.058 | [-0.14 - 0.086] | -0.326 | 0.639 | 0.745 | 57.679 | 942 |
| **Left paracentral lobule** | -0.027 | 0.033 | [-0.092 - 0.039] | -0.303 | 0.423 | 0.625 | <0.001 | 907 |
| **Left posterior cingulate cortex** | -0.026 | 0.033 | [-0.09 - 0.038] | -0.323 | 0.429 | 0.625 | <0.001 | 938 |
| **Left pericalcarine cortex** | -0.025 | 0.042 | [-0.107 - 0.057] | -0.352 | 0.549 | 0.693 | 25.959 | 939 |
| **Left banks superior temporal sulcus** | -0.025 | 0.111 | [-0.242 - 0.193] | -0.362 | 0.824 | 0.901 | 89.329 | 883 |
| **Right frontal pole** | -0.023 | 0.033 | [-0.087 - 0.041] | -0.483 | 0.478 | 0.645 | <0.001 | 941 |
| **Right transverse temporal gyrus** | -0.019 | 0.032 | [-0.083 - 0.045] | -0.307 | 0.557 | 0.693 | <0.001 | 943 |
| **Left cuneus** | -0.019 | 0.101 | [-0.216 - 0.179] | -0.231 | 0.853 | 0.914 | 87.237 | 931 |
| **Right lateral orbitofrontal cortex** | -0.018 | 0.043 | [-0.102 - 0.065] | -0.215 | 0.668 | 0.754 | 29.083 | 943 |
| **Right pars triangularis** | -0.014 | 0.033 | [-0.078 - 0.05] | -0.166 | 0.659 | 0.754 | <0.001 | 934 |
| **Left pars triangularis** | -0.012 | 0.045 | [-0.099 - 0.075] | -0.147 | 0.786 | 0.873 | 32.183 | 936 |
| **Left isthmus cingulate cortex** | -0.005 | 0.056 | [-0.115 - 0.104] | -0.083 | 0.923 | 0.941 | 55.272 | 937 |
| **Right caudal anterior cingulate cortex** | -0.005 | 0.057 | [-0.118 - 0.107] | -0.117 | 0.927 | 0.941 | 56.594 | 926 |
| **Left pars orbitalis** | -0.005 | 0.033 | [-0.069 - 0.059] | -0.075 | 0.875 | 0.914 | <0.001 | 942 |
| **Right pars opercularis** | 0.007 | 0.094 | [-0.178 - 0.191] | 0.066 | 0.945 | 0.945 | 85.349 | 929 |
| **Right isthmus cingulate cortex** | 0.008 | 0.047 | [-0.085 - 0.1] | 0.123 | 0.869 | 0.914 | 39.306 | 939 |

**^a^** Included Samples: CLING, Houston, Sexpect, MPIP, Muenster Cohort, SHIP, SHIP-trend, Stanford.

BDI-II: Beck Depression Inventory.

**Adolescent meta-analyses results for cortical thickness**

**Supplementary Table S34**: Full meta-analytic results for thickness of each structure for MDD patients versus Controls comparison controlling for age, sex and scan center. Adjusted Cohen's d is reported.

|  | **Cohen's d ^a^** | **Std. Err.** | **95% CI** | **% Difference** | **P-value** | **FDR P-value** | **I^2^** | **# Controls** | **# Patients** |
| --- | --- | --- | --- | --- | --- | --- | --- | --- | --- |
|  | **(MDD vs CTL)** |  |  |  |  |  |  |  |  |
| **Left lateral orbitofrontal cortex** | -0.311 | 0.107 | [-0.522 - -0.101] | -1.880 | 0.004 | 0.258 | <0.001 | 294 | 213 |
| **Right lateral orbitofrontal cortex** | -0.227 | 0.107 | [-0.437 - -0.016] | -1.334 | 0.035 | 0.691 | <0.001 | 294 | 211 |
| **Right medial orbitofrontal cortex** | -0.223 | 0.108 | [-0.436 - -0.011] | -1.875 | 0.039 | 0.691 | <0.001 | 282 | 211 |
| **Left isthmus cingulate cortex** | -0.222 | 0.107 | [-0.433 - -0.012] | -1.709 | 0.038 | 0.691 | <0.001 | 293 | 212 |
| **Left rostral anterior cingulate cortex** | -0.216 | 0.152 | [-0.515 - 0.082] | -2.105 | 0.156 | 0.979 | 44.717 | 294 | 213 |
| **Left frontal pole** | -0.182 | 0.240 | [-0.653 - 0.288] | -1.922 | 0.447 | 0.979 | 77.559 | 294 | 213 |
| **Left precentral gyrus** | -0.168 | 0.108 | [-0.379 - 0.043] | -0.963 | 0.119 | 0.979 | <0.001 | 291 | 212 |
| **Left medial orbitofrontal cortex** | -0.165 | 0.132 | [-0.423 - 0.093] | -1.278 | 0.211 | 0.979 | 26.370 | 283 | 210 |
| **Right rostral anterior cingulate cortex** | -0.155 | 0.107 | [-0.365 - 0.055] | -1.571 | 0.149 | 0.979 | <0.001 | 293 | 212 |
| **Left insula** | -0.152 | 0.134 | [-0.415 - 0.11] | -0.795 | 0.255 | 0.979 | 30.078 | 291 | 213 |
| **Left parahippocampal gyrus** | -0.151 | 0.107 | [-0.361 - 0.06] | -1.877 | 0.160 | 0.979 | <0.001 | 294 | 211 |
| **Left caudal anterior cingulate cortex** | -0.140 | 0.120 | [-0.376 - 0.096] | -1.593 | 0.244 | 0.979 | 16.397 | 291 | 213 |
| **Right pars opercularis** | -0.137 | 0.113 | [-0.359 - 0.085] | -0.686 | 0.227 | 0.979 | 7.860 | 292 | 213 |
| **Left paracentral gyrus** | -0.131 | 0.107 | [-0.341 - 0.079] | -0.746 | 0.221 | 0.979 | <0.001 | 294 | 212 |
| **Right isthmus cingulate cortex** | -0.129 | 0.107 | [-0.339 - 0.081] | -1.021 | 0.229 | 0.979 | <0.001 | 293 | 213 |
| **Right entorhinal cortex** | -0.117 | 0.109 | [-0.33 - 0.096] | -1.388 | 0.283 | 0.979 | <0.001 | 291 | 210 |
| **Right precentral gyrus** | -0.100 | 0.107 | [-0.31 - 0.11] | -0.594 | 0.351 | 0.979 | <0.001 | 290 | 213 |
| **Right inferior temporal gyrus** | -0.082 | 0.107 | [-0.291 - 0.128] | -0.444 | 0.444 | 0.979 | <0.001 | 291 | 213 |
| **Right middle temporal gyrus** | -0.066 | 0.137 | [-0.334 - 0.202] | -0.369 | 0.629 | 0.979 | 32.909 | 293 | 206 |
| **Left fusiform gyrus** | -0.061 | 0.107 | [-0.271 - 0.149] | -0.301 | 0.568 | 0.979 | <0.001 | 293 | 213 |
| **Right pars orbitalis** | -0.053 | 0.164 | [-0.375 - 0.269] | -0.382 | 0.747 | 0.979 | 52.081 | 294 | 210 |
| **Left posterior cingulate cortex** | -0.051 | 0.107 | [-0.261 - 0.159] | -0.319 | 0.634 | 0.979 | <0.001 | 293 | 213 |
| **Right superior frontal gyrus** | -0.046 | 0.121 | [-0.284 - 0.192] | -0.222 | 0.706 | 0.979 | 17.763 | 294 | 213 |
| **Right fusiform gyrus** | -0.036 | 0.167 | [-0.364 - 0.292] | -0.177 | 0.828 | 0.979 | 53.884 | 294 | 211 |
| **Left banks superior temporal sulcus** | -0.035 | 0.185 | [-0.397 - 0.328] | -0.255 | 0.851 | 0.979 | 59.448 | 279 | 190 |
| **Right superior temporal gyrus** | -0.033 | 0.107 | [-0.243 - 0.177] | -0.198 | 0.757 | 0.979 | <0.001 | 292 | 208 |
| **Right hemisphere average thickness** | -0.031 | 0.132 | [-0.29 - 0.228] | -0.114 | 0.813 | 0.979 | 28.685 | 294 | 213 |
| **Right postcentral gyrus** | -0.025 | 0.107 | [-0.235 - 0.186] | -0.140 | 0.818 | 0.979 | <0.001 | 289 | 212 |
| **Left pericalcarine cortex** | -0.023 | 0.107 | [-0.233 - 0.186] | -0.163 | 0.829 | 0.979 | <0.001 | 294 | 213 |
| **Left pars orbitalis** | -0.023 | 0.191 | [-0.397 - 0.35] | -0.169 | 0.903 | 0.979 | 64.240 | 292 | 213 |
| **Left lateral occipital cortex** | -0.018 | 0.187 | [-0.385 - 0.35] | -0.102 | 0.925 | 0.979 | 62.766 | 294 | 212 |
| **Right insula** | -0.014 | 0.107 | [-0.225 - 0.196] | -0.079 | 0.893 | 0.979 | <0.001 | 291 | 213 |
| **Left hempisphere average thickness** | -0.011 | 0.114 | [-0.235 - 0.214] | -0.039 | 0.926 | 0.979 | 9.648 | 294 | 213 |
| **Right cuneus** | -0.009 | 0.107 | [-0.22 - 0.201] | -0.057 | 0.932 | 0.979 | 0.001 | 292 | 213 |
| **Right inferior parietal cortex** | -0.009 | 0.157 | [-0.317 - 0.299] | -0.051 | 0.954 | 0.979 | 48.150 | 293 | 213 |
| **Left rostral middle frontal gyrus** | -0.007 | 0.107 | [-0.216 - 0.202] | -0.034 | 0.948 | 0.979 | <0.001 | 294 | 213 |
| **Right frontal pole** | -0.007 | 0.121 | [-0.244 - 0.231] | -0.071 | 0.955 | 0.979 | 17.542 | 294 | 213 |
| **Right paracentral gyrus** | -0.003 | 0.107 | [-0.213 - 0.208] | -0.016 | 0.979 | 0.979 | <0.001 | 291 | 213 |
| **Left superior frontal gyrus** | 0.003 | 0.107 | [-0.207 - 0.213] | 0.015 | 0.977 | 0.979 | <0.001 | 293 | 212 |
| **Left caudal middle frontal gyrus** | 0.004 | 0.107 | [-0.205 - 0.214] | 0.022 | 0.967 | 0.979 | <0.001 | 294 | 213 |
| **Left superior temporal gyrus** | 0.006 | 0.109 | [-0.208 - 0.22] | 0.035 | 0.958 | 0.979 | 0.001 | 283 | 198 |
| **Right parahippocampal gyrus** | 0.007 | 0.113 | [-0.214 - 0.228] | 0.075 | 0.952 | 0.979 | 7.035 | 294 | 212 |
| **Right superior parietal cortex** | 0.009 | 0.136 | [-0.258 - 0.276] | 0.046 | 0.946 | 0.979 | 32.178 | 293 | 213 |
| **Left inferior parietal cortex** | 0.010 | 0.107 | [-0.2 - 0.221] | 0.059 | 0.922 | 0.979 | 0.298 | 294 | 213 |
| **Left postcentral gyrus** | 0.011 | 0.107 | [-0.199 - 0.221] | 0.061 | 0.918 | 0.979 | <0.001 | 294 | 212 |
| **Right precuneus** | 0.022 | 0.157 | [-0.285 - 0.328] | 0.108 | 0.890 | 0.979 | 47.669 | 294 | 213 |
| **Left precuneus** | 0.022 | 0.210 | [-0.389 - 0.433] | 0.106 | 0.916 | 0.979 | 70.419 | 294 | 213 |
| **Left supramarginal gyrus** | 0.025 | 0.107 | [-0.186 - 0.235] | 0.133 | 0.817 | 0.979 | <0.001 | 291 | 213 |
| **Right supramarginal gyrus** | 0.032 | 0.107 | [-0.179 - 0.242] | 0.163 | 0.768 | 0.979 | <0.001 | 288 | 211 |
| **Right lateral occipital cortex** | 0.033 | 0.140 | [-0.242 - 0.308] | 0.193 | 0.814 | 0.979 | 36.094 | 294 | 213 |
| **Right rostral middle frontal gyrus** | 0.034 | 0.140 | [-0.24 - 0.308] | 0.165 | 0.806 | 0.979 | 35.371 | 292 | 213 |
| **Left middle temporal gyrus** | 0.034 | 0.110 | [-0.181 - 0.249] | 0.204 | 0.754 | 0.979 | <0.001 | 284 | 196 |
| **Left lingual gyrus** | 0.037 | 0.107 | [-0.173 - 0.246] | 0.213 | 0.731 | 0.979 | <0.001 | 294 | 213 |
| **Left temporal pole** | 0.044 | 0.107 | [-0.166 - 0.254] | 0.448 | 0.679 | 0.979 | 0.002 | 294 | 213 |
| **Left superior parietal cortex** | 0.050 | 0.107 | [-0.159 - 0.26] | 0.247 | 0.637 | 0.979 | 0.006 | 294 | 213 |
| **Right banks superior temporal sulcus** | 0.054 | 0.140 | [-0.22 - 0.327] | 0.381 | 0.701 | 0.979 | 34.767 | 293 | 203 |
| **Right pericalcarine cortex** | 0.063 | 0.107 | [-0.146 - 0.273] | 0.463 | 0.554 | 0.979 | <0.001 | 294 | 212 |
| **Left cuneus** | 0.072 | 0.197 | [-0.315 - 0.458] | 0.442 | 0.716 | 0.979 | 66.245 | 293 | 213 |
| **Right transverse temporal gyrus** | 0.073 | 0.107 | [-0.136 - 0.283] | 0.591 | 0.492 | 0.979 | <0.001 | 294 | 213 |
| **Left transverse temporal gyrus** | 0.075 | 0.118 | [-0.156 - 0.307] | 0.593 | 0.524 | 0.979 | 13.790 | 294 | 213 |
| **Left pars triangularis** | 0.077 | 0.121 | [-0.161 - 0.314] | 0.466 | 0.527 | 0.979 | 17.231 | 293 | 213 |
| **Right posterior cingulate cortex** | 0.083 | 0.114 | [-0.14 - 0.306] | 0.555 | 0.466 | 0.979 | 8.675 | 293 | 213 |
| **Right pars triangularis** | 0.087 | 0.107 | [-0.123 - 0.297] | 0.501 | 0.417 | 0.979 | <0.001 | 293 | 213 |
| **Right caudal anterior cingulate cortex** | 0.087 | 0.131 | [-0.17 - 0.344] | 0.971 | 0.506 | 0.979 | 27.776 | 293 | 213 |
| **Left inferior temporal gyrus** | 0.106 | 0.108 | [-0.105 - 0.317] | 0.594 | 0.323 | 0.979 | <0.001 | 291 | 212 |
| **Right lingual gyrus** | 0.119 | 0.107 | [-0.091 - 0.328] | 0.687 | 0.267 | 0.979 | <0.001 | 294 | 213 |
| **Right caudal middle frontal gyrus** | 0.145 | 0.132 | [-0.114 - 0.403] | 0.761 | 0.274 | 0.979 | 28.073 | 294 | 211 |
| **Left pars opercularis** | 0.150 | 0.107 | [-0.061 - 0.361] | 0.715 | 0.163 | 0.979 | <0.001 | 293 | 211 |
| **Left entorhinal cortex** | 0.160 | 0.110 | [-0.055 - 0.375] | 1.843 | 0.144 | 0.979 | 2.664 | 292 | 210 |
| **Right temporal pole** | 0.210 | 0.174 | [-0.131 - 0.551] | 2.312 | 0.227 | 0.979 | 56.849 | 294 | 213 |

**^a^** Included Samples: MMDP 3T, Melbourne, Muenster Cohort, QTIM, Sydney.

MDD: Major Depressive Disorder; CTL: Controls.

**Supplementary Table S35**: Full meta-analytic results for thickness of each structure for the Diagnosis by Sex interaction controlling for age, sex and scan center. Adjusted Cohen's d is reported.

|  | **Cohen's d ^a^** | **Std. Err.** | **95% CI** | **% Difference** | **P-value** | **FDR P-value** | **I^2^** | **# Controls** | **# Patients** |
| --- | --- | --- | --- | --- | --- | --- | --- | --- | --- |
|  | **(Dx by Sex)** |  |  |  |  |  |  |  |  |
| **Right pars triangularis** | -0.190 | 0.180 | [-0.544 - 0.164] | -1.095 | 0.292 | 0.978 | 60.064 | 293 | 213 |
| **Left insula** | -0.186 | 0.120 | [-0.422 - 0.05] | -0.971 | 0.122 | 0.978 | 16.175 | 291 | 213 |
| **Right supramarginal gyrus** | -0.177 | 0.113 | [-0.399 - 0.046] | -0.908 | 0.119 | 0.978 | 7.187 | 288 | 211 |
| **Left superior frontal gyrus** | -0.171 | 0.107 | [-0.382 - 0.039] | -0.866 | 0.111 | 0.978 | <0.001 | 293 | 212 |
| **Left medial orbitofrontal cortex** | -0.153 | 0.108 | [-0.365 - 0.06] | -1.183 | 0.159 | 0.978 | <0.001 | 283 | 210 |
| **Left entorhinal cortex** | -0.147 | 0.107 | [-0.358 - 0.063] | -1.695 | 0.170 | 0.978 | <0.001 | 292 | 210 |
| **Left superior parietal cortex** | -0.140 | 0.107 | [-0.35 - 0.069] | -0.688 | 0.189 | 0.978 | <0.001 | 294 | 213 |
| **Right posterior cingulate cortex** | -0.131 | 0.194 | [-0.511 - 0.249] | -0.875 | 0.500 | 0.978 | 65.427 | 293 | 213 |
| **Right paracentral gyrus** | -0.127 | 0.152 | [-0.425 - 0.172] | -0.718 | 0.407 | 0.978 | 44.646 | 291 | 213 |
| **Right pars orbitalis** | -0.124 | 0.144 | [-0.407 - 0.159] | -0.893 | 0.391 | 0.978 | 38.880 | 294 | 210 |
| **Right precentral gyrus** | -0.115 | 0.145 | [-0.399 - 0.168] | -0.686 | 0.426 | 0.978 | 39.205 | 290 | 213 |
| **Left frontal pole** | -0.106 | 0.107 | [-0.315 - 0.104] | -1.112 | 0.323 | 0.978 | <0.001 | 294 | 213 |
| **Right superior frontal gyrus** | -0.101 | 0.122 | [-0.339 - 0.138] | -0.488 | 0.409 | 0.978 | 17.746 | 294 | 213 |
| **Right insula** | -0.087 | 0.141 | [-0.364 - 0.19] | -0.476 | 0.537 | 0.978 | 36.559 | 291 | 213 |
| **Right parahippocampal gyrus** | -0.083 | 0.108 | [-0.295 - 0.129] | -0.905 | 0.443 | 0.978 | 1.046 | 294 | 212 |
| **Right lateral orbitofrontal cortex** | -0.079 | 0.107 | [-0.289 - 0.131] | -0.466 | 0.460 | 0.978 | <0.001 | 294 | 211 |
| **Right postcentral gyrus** | -0.078 | 0.157 | [-0.386 - 0.229] | -0.444 | 0.617 | 0.978 | 47.475 | 289 | 212 |
| **Left precentral gyrus** | -0.076 | 0.147 | [-0.364 - 0.211] | -0.438 | 0.603 | 0.978 | 40.600 | 291 | 212 |
| **Right cuneus** | -0.072 | 0.107 | [-0.282 - 0.138] | -0.443 | 0.501 | 0.978 | <0.001 | 292 | 213 |
| **Right lateral occipital cortex** | -0.070 | 0.107 | [-0.28 - 0.139] | -0.412 | 0.510 | 0.978 | <0.001 | 294 | 213 |
| **Left lateral orbitofrontal cortex** | -0.069 | 0.107 | [-0.278 - 0.141] | -0.414 | 0.521 | 0.978 | <0.001 | 294 | 213 |
| **Right entorhinal cortex** | -0.067 | 0.109 | [-0.281 - 0.146] | -0.801 | 0.536 | 0.978 | 0.001 | 291 | 210 |
| **Left rostral anterior cingulate cortex** | -0.065 | 0.107 | [-0.275 - 0.145] | -0.632 | 0.544 | 0.978 | 0.010 | 294 | 213 |
| **Left rostral middle frontal gyrus** | -0.061 | 0.132 | [-0.32 - 0.198] | -0.294 | 0.646 | 0.978 | 28.606 | 294 | 213 |
| **Right inferior temporal gyrus** | -0.060 | 0.166 | [-0.384 - 0.265] | -0.324 | 0.718 | 0.978 | 53.049 | 291 | 213 |
| **Right superior parietal cortex** | -0.058 | 0.121 | [-0.294 - 0.179] | -0.288 | 0.633 | 0.978 | 16.750 | 293 | 213 |
| **Left pericalcarine cortex** | -0.052 | 0.107 | [-0.262 - 0.157] | -0.369 | 0.624 | 0.978 | <0.001 | 294 | 213 |
| **Left isthmus cingulate cortex** | -0.050 | 0.107 | [-0.26 - 0.16] | -0.386 | 0.639 | 0.978 | <0.001 | 293 | 212 |
| **Right pars opercularis** | -0.045 | 0.146 | [-0.331 - 0.241] | -0.227 | 0.757 | 0.978 | 40.310 | 292 | 213 |
| **Right isthmus cingulate cortex** | -0.038 | 0.107 | [-0.248 - 0.172] | -0.302 | 0.722 | 0.978 | <0.001 | 293 | 213 |
| **Left pars triangularis** | -0.038 | 0.163 | [-0.356 - 0.281] | -0.229 | 0.817 | 0.978 | 51.275 | 293 | 213 |
| **Left transverse temporal gyrus** | -0.036 | 0.107 | [-0.246 - 0.173] | -0.287 | 0.733 | 0.978 | <0.001 | 294 | 213 |
| **Right hemisphere average thickness** | -0.035 | 0.123 | [-0.276 - 0.206] | -0.128 | 0.775 | 0.978 | 19.426 | 294 | 213 |
| **Left supramarginal gyrus** | -0.032 | 0.107 | [-0.243 - 0.179] | -0.172 | 0.766 | 0.978 | <0.001 | 291 | 213 |
| **Right pericalcarine cortex** | -0.032 | 0.107 | [-0.241 - 0.178] | -0.230 | 0.769 | 0.978 | <0.001 | 294 | 212 |
| **Right inferior parietal cortex** | -0.014 | 0.107 | [-0.223 - 0.196] | -0.077 | 0.899 | 0.978 | <0.001 | 293 | 213 |
| **Left caudal middle frontal gyrus** | -0.013 | 0.107 | [-0.223 - 0.197] | -0.066 | 0.904 | 0.978 | <0.001 | 294 | 213 |
| **Right fusiform gyrus** | -0.011 | 0.154 | [-0.312 - 0.291] | -0.053 | 0.944 | 0.978 | 45.908 | 294 | 211 |
| **Right rostral anterior cingulate cortex** | -0.009 | 0.107 | [-0.219 - 0.201] | -0.095 | 0.930 | 0.978 | <0.001 | 293 | 212 |
| **Left parahippocampal gyrus** | -0.005 | 0.107 | [-0.215 - 0.205] | -0.065 | 0.961 | 0.978 | <0.001 | 294 | 211 |
| **Right temporal pole** | -0.001 | 0.107 | [-0.211 - 0.209] | -0.011 | 0.992 | 0.992 | 0.004 | 294 | 213 |
| **Left hempisphere average thickness** | 0.005 | 0.107 | [-0.205 - 0.214] | 0.018 | 0.964 | 0.978 | <0.001 | 294 | 213 |
| **Left inferior temporal gyrus** | 0.007 | 0.120 | [-0.227 - 0.242] | 0.041 | 0.951 | 0.978 | 14.926 | 291 | 212 |
| **Left pars orbitalis** | 0.009 | 0.133 | [-0.251 - 0.27] | 0.069 | 0.944 | 0.978 | 29.382 | 292 | 213 |
| **Right caudal middle frontal gyrus** | 0.011 | 0.117 | [-0.219 - 0.24] | 0.056 | 0.928 | 0.978 | 12.347 | 294 | 211 |
| **Left pars opercularis** | 0.012 | 0.198 | [-0.375 - 0.4] | 0.058 | 0.951 | 0.978 | 66.539 | 293 | 211 |
| **Right medial orbitofrontal cortex** | 0.012 | 0.129 | [-0.24 - 0.265] | 0.103 | 0.924 | 0.978 | 24.176 | 282 | 211 |
| **Right precuneus** | 0.016 | 0.107 | [-0.194 - 0.226] | 0.079 | 0.881 | 0.978 | <0.001 | 294 | 213 |
| **Right banks superior temporal sulcus** | 0.017 | 0.107 | [-0.193 - 0.228] | 0.124 | 0.871 | 0.978 | <0.001 | 293 | 203 |
| **Right frontal pole** | 0.020 | 0.107 | [-0.189 - 0.23] | 0.213 | 0.849 | 0.978 | <0.001 | 294 | 213 |
| **Right superior temporal gyrus** | 0.020 | 0.139 | [-0.253 - 0.293] | 0.122 | 0.883 | 0.978 | 34.897 | 292 | 208 |
| **Left middle temporal gyrus** | 0.021 | 0.110 | [-0.194 - 0.236] | 0.126 | 0.846 | 0.978 | <0.001 | 284 | 196 |
| **Left precuneus** | 0.028 | 0.107 | [-0.182 - 0.238] | 0.133 | 0.794 | 0.978 | <0.001 | 294 | 213 |
| **Left paracentral gyrus** | 0.031 | 0.140 | [-0.243 - 0.305] | 0.178 | 0.823 | 0.978 | 35.529 | 294 | 212 |
| **Left superior temporal gyrus** | 0.042 | 0.109 | [-0.171 - 0.256] | 0.258 | 0.698 | 0.978 | <0.001 | 283 | 198 |
| **Right transverse temporal gyrus** | 0.046 | 0.107 | [-0.164 - 0.255] | 0.369 | 0.668 | 0.978 | <0.001 | 294 | 213 |
| **Right caudal anterior cingulate cortex** | 0.046 | 0.109 | [-0.167 - 0.259] | 0.513 | 0.672 | 0.978 | 2.279 | 293 | 213 |
| **Right middle temporal gyrus** | 0.060 | 0.122 | [-0.179 - 0.299] | 0.335 | 0.622 | 0.978 | 18.100 | 293 | 206 |
| **Left lateral occipital cortex** | 0.074 | 0.107 | [-0.136 - 0.284] | 0.427 | 0.489 | 0.978 | <0.001 | 294 | 212 |
| **Left inferior parietal cortex** | 0.074 | 0.107 | [-0.135 - 0.284] | 0.421 | 0.488 | 0.978 | <0.001 | 294 | 213 |
| **Right rostral middle frontal gyrus** | 0.074 | 0.107 | [-0.136 - 0.284] | 0.358 | 0.489 | 0.978 | 0.005 | 292 | 213 |
| **Left temporal pole** | 0.085 | 0.107 | [-0.124 - 0.295] | 0.864 | 0.425 | 0.978 | 0.003 | 294 | 213 |
| **Left postcentral gyrus** | 0.086 | 0.107 | [-0.124 - 0.296] | 0.474 | 0.423 | 0.978 | <0.001 | 294 | 212 |
| **Left posterior cingulate cortex** | 0.089 | 0.107 | [-0.121 - 0.299] | 0.559 | 0.405 | 0.978 | <0.001 | 293 | 213 |
| **Left caudal anterior cingulate cortex** | 0.093 | 0.138 | [-0.177 - 0.363] | 1.057 | 0.500 | 0.978 | 33.332 | 291 | 213 |
| **Left cuneus** | 0.116 | 0.107 | [-0.094 - 0.326] | 0.715 | 0.280 | 0.978 | <0.001 | 293 | 213 |
| **Left banks superior temporal sulcus** | 0.116 | 0.111 | [-0.101 - 0.333] | 0.851 | 0.293 | 0.978 | <0.001 | 279 | 190 |
| **Left fusiform gyrus** | 0.130 | 0.107 | [-0.08 - 0.34] | 0.640 | 0.224 | 0.978 | <0.001 | 293 | 213 |
| **Right lingual gyrus** | 0.153 | 0.107 | [-0.056 - 0.363] | 0.887 | 0.152 | 0.978 | <0.001 | 294 | 213 |
| **Left lingual gyrus** | 0.162 | 0.107 | [-0.048 - 0.372] | 0.937 | 0.130 | 0.978 | <0.001 | 294 | 213 |

**^a^** Included Samples: MMDP 3T, Melbourne, Muenster Cohort, QTIM, Sydney.

Dx: Diagnosis.

**Supplementary Table S36**: Full meta-analytic results for thickness of each structure for the Diagnosis by Age interaction controlling for age, sex and scan center. Adjusted Cohen's d is reported.

|  | **Cohen's d ^a^** | **Std. Err.** | **95% CI** | **% Difference** | **P-value** | **FDR P-value** | **I^2^** | **# Controls** | **# Patients** |
| --- | --- | --- | --- | --- | --- | --- | --- | --- | --- |
|  | **(Dx by Age)** |  |  |  |  |  |  |  |  |
| **Right pericalcarine cortex** | -0.157 | 0.109 | [-0.371 - 0.058] | -1.142 | 0.152 | 0.820 | 2.807 | 294 | 212 |
| **Left superior parietal cortex** | -0.098 | 0.107 | [-0.308 - 0.112] | -0.479 | 0.360 | 0.971 | <0.001 | 294 | 213 |
| **Right postcentral gyrus** | -0.097 | 0.107 | [-0.308 - 0.113] | -0.551 | 0.365 | 0.971 | <0.001 | 289 | 212 |
| **Left pars opercularis** | -0.096 | 0.107 | [-0.306 - 0.115] | -0.456 | 0.373 | 0.971 | <0.001 | 293 | 211 |
| **Left pericalcarine cortex** | -0.073 | 0.107 | [-0.282 - 0.137] | -0.511 | 0.497 | 0.971 | <0.001 | 294 | 213 |
| **Left superior frontal gyrus** | -0.063 | 0.107 | [-0.273 - 0.147] | -0.321 | 0.554 | 0.971 | <0.001 | 293 | 212 |
| **Right paracentral gyrus** | -0.062 | 0.107 | [-0.272 - 0.149] | -0.350 | 0.566 | 0.971 | <0.001 | 291 | 213 |
| **Right superior parietal cortex** | -0.059 | 0.107 | [-0.269 - 0.15] | -0.297 | 0.579 | 0.971 | <0.001 | 293 | 213 |
| **Right pars triangularis** | -0.055 | 0.155 | [-0.359 - 0.249] | -0.317 | 0.723 | 0.971 | 46.515 | 293 | 213 |
| **Left precentral gyrus** | -0.051 | 0.108 | [-0.262 - 0.16] | -0.293 | 0.635 | 0.971 | <0.001 | 291 | 212 |
| **Right medial orbitofrontal cortex** | -0.049 | 0.146 | [-0.336 - 0.237] | -0.415 | 0.735 | 0.971 | 39.323 | 282 | 211 |
| **Right lateral orbitofrontal cortex** | -0.040 | 0.174 | [-0.382 - 0.302] | -0.235 | 0.819 | 0.971 | 57.078 | 294 | 211 |
| **Left rostral middle frontal gyrus** | -0.034 | 0.107 | [-0.243 - 0.176] | -0.163 | 0.754 | 0.971 | <0.001 | 294 | 213 |
| **Right insula** | -0.031 | 0.107 | [-0.241 - 0.18] | -0.167 | 0.775 | 0.971 | 0.008 | 291 | 213 |
| **Left frontal pole** | -0.027 | 0.118 | [-0.258 - 0.203] | -0.289 | 0.816 | 0.971 | 13.423 | 294 | 213 |
| **Right caudal anterior cingulate cortex** | -0.027 | 0.189 | [-0.397 - 0.343] | -0.297 | 0.888 | 0.971 | 63.371 | 293 | 213 |
| **Right rostral middle frontal gyrus** | -0.027 | 0.107 | [-0.237 - 0.183] | -0.129 | 0.803 | 0.971 | 0.003 | 292 | 213 |
| **Left lateral orbitofrontal cortex** | -0.027 | 0.107 | [-0.237 - 0.183] | -0.161 | 0.804 | 0.971 | 0.008 | 294 | 213 |
| **Right superior frontal gyrus** | -0.013 | 0.107 | [-0.222 - 0.197] | -0.062 | 0.904 | 0.971 | <0.001 | 294 | 213 |
| **Left pars orbitalis** | -0.012 | 0.108 | [-0.222 - 0.199] | -0.085 | 0.914 | 0.971 | <0.001 | 292 | 213 |
| **Left transverse temporal gyrus** | -0.004 | 0.107 | [-0.214 - 0.206] | -0.031 | 0.971 | 0.971 | 0.003 | 294 | 213 |
| **Left lateral occipital cortex** | 0.005 | 0.107 | [-0.205 - 0.214] | 0.027 | 0.965 | 0.971 | <0.001 | 294 | 212 |
| **Right rostral anterior cingulate cortex** | 0.007 | 0.107 | [-0.203 - 0.217] | 0.069 | 0.949 | 0.971 | <0.001 | 293 | 212 |
| **Right pars opercularis** | 0.007 | 0.122 | [-0.232 - 0.246] | 0.035 | 0.954 | 0.971 | 18.361 | 292 | 213 |
| **Left banks superior temporal sulcus** | 0.010 | 0.110 | [-0.206 - 0.227] | 0.074 | 0.927 | 0.971 | <0.001 | 279 | 190 |
| **Left caudal middle frontal gyrus** | 0.013 | 0.107 | [-0.197 - 0.223] | 0.068 | 0.900 | 0.971 | 0.010 | 294 | 213 |
| **Left postcentral gyrus** | 0.014 | 0.107 | [-0.196 - 0.224] | 0.077 | 0.896 | 0.971 | <0.001 | 294 | 212 |
| **Right lingual gyrus** | 0.024 | 0.131 | [-0.233 - 0.281] | 0.136 | 0.858 | 0.971 | 27.853 | 294 | 213 |
| **Left lingual gyrus** | 0.025 | 0.107 | [-0.185 - 0.234] | 0.142 | 0.818 | 0.971 | <0.001 | 294 | 213 |
| **Right precentral gyrus** | 0.029 | 0.107 | [-0.181 - 0.238] | 0.170 | 0.790 | 0.971 | <0.001 | 290 | 213 |
| **Left pars triangularis** | 0.034 | 0.107 | [-0.176 - 0.244] | 0.206 | 0.752 | 0.971 | <0.001 | 293 | 213 |
| **Right caudal middle frontal gyrus** | 0.036 | 0.107 | [-0.174 - 0.246] | 0.191 | 0.735 | 0.971 | <0.001 | 294 | 211 |
| **Left precuneus** | 0.047 | 0.107 | [-0.162 - 0.256] | 0.224 | 0.660 | 0.971 | <0.001 | 294 | 213 |
| **Right entorhinal cortex** | 0.048 | 0.109 | [-0.165 - 0.261] | 0.570 | 0.659 | 0.971 | <0.001 | 291 | 210 |
| **Left parahippocampal gyrus** | 0.048 | 0.137 | [-0.221 - 0.318] | 0.603 | 0.724 | 0.971 | 33.215 | 294 | 211 |
| **Left insula** | 0.055 | 0.107 | [-0.156 - 0.265] | 0.285 | 0.611 | 0.971 | 0.011 | 291 | 213 |
| **Right posterior cingulate cortex** | 0.056 | 0.107 | [-0.154 - 0.266] | 0.372 | 0.604 | 0.971 | 0.001 | 293 | 213 |
| **Right isthmus cingulate cortex** | 0.056 | 0.127 | [-0.193 - 0.304] | 0.443 | 0.659 | 0.971 | 23.067 | 293 | 213 |
| **Left hempisphere average thickness** | 0.058 | 0.107 | [-0.151 - 0.268] | 0.214 | 0.585 | 0.971 | <0.001 | 294 | 213 |
| **Left inferior parietal cortex** | 0.060 | 0.107 | [-0.15 - 0.27] | 0.341 | 0.574 | 0.971 | <0.001 | 294 | 213 |
| **Right pars orbitalis** | 0.071 | 0.180 | [-0.281 - 0.423] | 0.509 | 0.694 | 0.971 | 59.425 | 294 | 210 |
| **Left rostral anterior cingulate cortex** | 0.075 | 0.107 | [-0.135 - 0.285] | 0.732 | 0.482 | 0.971 | <0.001 | 294 | 213 |
| **Left inferior temporal gyrus** | 0.077 | 0.111 | [-0.141 - 0.295] | 0.431 | 0.488 | 0.971 | 4.960 | 291 | 212 |
| **Right hemisphere average thickness** | 0.086 | 0.107 | [-0.124 - 0.295] | 0.313 | 0.422 | 0.971 | <0.001 | 294 | 213 |
| **Left medial orbitofrontal cortex** | 0.087 | 0.108 | [-0.125 - 0.298] | 0.671 | 0.424 | 0.971 | <0.001 | 283 | 210 |
| **Left paracentral gyrus** | 0.094 | 0.122 | [-0.145 - 0.333] | 0.537 | 0.439 | 0.971 | 18.134 | 294 | 212 |
| **Right cuneus** | 0.095 | 0.107 | [-0.116 - 0.305] | 0.581 | 0.378 | 0.971 | <0.001 | 292 | 213 |
| **Right temporal pole** | 0.096 | 0.107 | [-0.114 - 0.306] | 1.056 | 0.369 | 0.971 | <0.001 | 294 | 213 |
| **Right middle temporal gyrus** | 0.100 | 0.107 | [-0.11 - 0.309] | 0.556 | 0.351 | 0.971 | <0.001 | 293 | 206 |
| **Left cuneus** | 0.102 | 0.159 | [-0.211 - 0.414] | 0.627 | 0.524 | 0.971 | 49.023 | 293 | 213 |
| **Right inferior temporal gyrus** | 0.103 | 0.107 | [-0.107 - 0.313] | 0.557 | 0.338 | 0.971 | <0.001 | 291 | 213 |
| **Left fusiform gyrus** | 0.111 | 0.107 | [-0.098 - 0.321] | 0.548 | 0.298 | 0.971 | <0.001 | 293 | 213 |
| **Right inferior parietal cortex** | 0.114 | 0.107 | [-0.096 - 0.324] | 0.645 | 0.288 | 0.971 | <0.001 | 293 | 213 |
| **Right precuneus** | 0.124 | 0.107 | [-0.086 - 0.333] | 0.615 | 0.247 | 0.971 | <0.001 | 294 | 213 |
| **Right supramarginal gyrus** | 0.132 | 0.108 | [-0.079 - 0.343] | 0.678 | 0.221 | 0.971 | 0.007 | 288 | 211 |
| **Left supramarginal gyrus** | 0.150 | 0.162 | [-0.167 - 0.466] | 0.805 | 0.355 | 0.971 | 50.638 | 291 | 213 |
| **Left posterior cingulate cortex** | 0.157 | 0.107 | [-0.053 - 0.367] | 0.981 | 0.144 | 0.820 | <0.001 | 293 | 213 |
| **Right transverse temporal gyrus** | 0.158 | 0.125 | [-0.087 - 0.403] | 1.271 | 0.206 | 0.971 | 21.480 | 294 | 213 |
| **Left superior temporal gyrus** | 0.165 | 0.109 | [-0.049 - 0.379] | 1.007 | 0.131 | 0.820 | <0.001 | 283 | 198 |
| **Right lateral occipital cortex** | 0.167 | 0.107 | [-0.042 - 0.377] | 0.980 | 0.117 | 0.820 | <0.001 | 294 | 213 |
| **Right fusiform gyrus** | 0.171 | 0.107 | [-0.04 - 0.381] | 0.833 | 0.112 | 0.820 | 0.004 | 294 | 211 |
| **Left middle temporal gyrus** | 0.173 | 0.110 | [-0.042 - 0.388] | 1.027 | 0.115 | 0.820 | <0.001 | 284 | 196 |
| **Left isthmus cingulate cortex** | 0.173 | 0.142 | [-0.105 - 0.452] | 1.333 | 0.223 | 0.971 | 37.015 | 293 | 212 |
| **Right banks superior temporal sulcus** | 0.179 | 0.107 | [-0.031 - 0.39] | 1.275 | 0.095 | 0.820 | <0.001 | 293 | 203 |
| **Left temporal pole** | 0.185 | 0.107 | [-0.024 - 0.395] | 1.871 | 0.083 | 0.820 | <0.001 | 294 | 213 |
| **Right superior temporal gyrus** | 0.197 | 0.107 | [-0.014 - 0.407] | 1.177 | 0.067 | 0.820 | <0.001 | 292 | 208 |
| **Left caudal anterior cingulate cortex** | 0.230 | 0.137 | [-0.038 - 0.498] | 2.608 | 0.093 | 0.820 | 32.428 | 291 | 213 |
| **Left entorhinal cortex** | 0.240 | 0.108 | [0.029 - 0.45] | 2.757 | 0.026 | 0.820 | <0.001 | 292 | 210 |
| **Right parahippocampal gyrus** | 0.275 | 0.141 | [-0.002 - 0.552] | 2.997 | 0.052 | 0.820 | 35.843 | 294 | 212 |
| **Right frontal pole** | 0.288 | 0.107 | [0.077 - 0.498] | 3.000 | 0.007 | 0.523 | 0.002 | 294 | 213 |

**^a^** Included Samples: MMDP 3T, Melbourne, Muenster Cohort, QTIM, Sydney.

Dx: Diagnosis.

**Supplementary Table S37**: Full meta-analytic results for thickness of each structure for first episode MDD patients versus Controls comparison controlling for age, sex and scan center. Adjusted Cohen's d is reported.

|  | **Cohen's d ^a^** | **Std. Err.** | **95% CI** | **% Difference** | **P-value** | **FDR P-value** | **I^2^** | **# Controls** | **# Patients** |
| --- | --- | --- | --- | --- | --- | --- | --- | --- | --- |
|  | **(First episode MDD vs CTL)** |  |  |  |  |  |  |  |  |
| **Left rostral anterior cingulate cortex** | -0.379 | 0.153 | [-0.679 - -0.078] | -3.687 | 0.013 | 0.907 | <0.001 | 154 | 80 |
| **Left fusiform gyrus** | -0.378 | 0.197 | [-0.765 - 0.008] | -1.858 | 0.055 | 0.907 | 33.680 | 153 | 80 |
| **Left lateral occipital cortex** | -0.370 | 0.319 | [-0.996 - 0.256] | -2.131 | 0.246 | 0.907 | 73.485 | 154 | 80 |
| **Right lateral orbitofrontal cortex** | -0.359 | 0.252 | [-0.852 - 0.135] | -2.111 | 0.154 | 0.907 | 58.117 | 154 | 80 |
| **Left medial orbitofrontal cortex** | -0.295 | 0.154 | [-0.597 - 0.008] | -2.284 | 0.056 | 0.907 | 0.002 | 154 | 79 |
| **Right medial orbitofrontal cortex** | -0.276 | 0.153 | [-0.577 - 0.024] | -2.320 | 0.071 | 0.907 | <0.001 | 153 | 80 |
| **Right superior temporal gyrus** | -0.263 | 0.190 | [-0.634 - 0.109] | -1.573 | 0.166 | 0.907 | 29.338 | 154 | 79 |
| **Left insula** | -0.259 | 0.230 | [-0.709 - 0.192] | -1.350 | 0.261 | 0.907 | 50.393 | 152 | 80 |
| **Right insula** | -0.230 | 0.153 | [-0.529 - 0.07] | -1.251 | 0.133 | 0.907 | <0.001 | 151 | 80 |
| **Right fusiform gyrus** | -0.228 | 0.193 | [-0.606 - 0.151] | -1.111 | 0.238 | 0.907 | 31.630 | 154 | 80 |
| **Left precentral gyrus** | -0.210 | 0.153 | [-0.51 - 0.09] | -1.205 | 0.170 | 0.907 | <0.001 | 151 | 80 |
| **Right entorhinal cortex** | -0.202 | 0.155 | [-0.507 - 0.103] | -2.401 | 0.194 | 0.907 | <0.001 | 151 | 79 |
| **Left parahippocampal gyrus** | -0.196 | 0.154 | [-0.498 - 0.105] | -2.445 | 0.201 | 0.907 | 0.003 | 154 | 79 |
| **Right rostral anterior cingulate cortex** | -0.185 | 0.153 | [-0.484 - 0.115] | -1.874 | 0.226 | 0.907 | <0.001 | 153 | 80 |
| **Left caudal anterior cingulate cortex** | -0.177 | 0.153 | [-0.476 - 0.121] | -2.016 | 0.245 | 0.907 | <0.001 | 152 | 80 |
| **Left lateral orbitofrontal cortex** | -0.175 | 0.152 | [-0.473 - 0.124] | -1.054 | 0.251 | 0.907 | <0.001 | 154 | 80 |
| **Right pars orbitalis** | -0.174 | 0.343 | [-0.847 - 0.499] | -1.254 | 0.612 | 0.907 | 77.259 | 154 | 79 |
| **Right cuneus** | -0.171 | 0.245 | [-0.652 - 0.309] | -1.053 | 0.484 | 0.907 | 56.099 | 152 | 80 |
| **Left inferior parietal cortex** | -0.169 | 0.230 | [-0.619 - 0.282] | -0.958 | 0.463 | 0.907 | 50.592 | 154 | 80 |
| **Left hempisphere average thickness** | -0.167 | 0.255 | [-0.666 - 0.332] | -0.614 | 0.512 | 0.907 | 59.558 | 154 | 80 |
| **Left supramarginal gyrus** | -0.166 | 0.203 | [-0.564 - 0.232] | -0.892 | 0.415 | 0.907 | 37.666 | 151 | 80 |
| **Right lateral occipital cortex** | -0.154 | 0.174 | [-0.496 - 0.188] | -0.899 | 0.379 | 0.907 | 18.932 | 154 | 80 |
| **Right hemisphere average thickness** | -0.146 | 0.262 | [-0.66 - 0.367] | -0.535 | 0.576 | 0.907 | 61.812 | 154 | 80 |
| **Left postcentral gyrus** | -0.139 | 0.153 | [-0.44 - 0.161] | -0.769 | 0.363 | 0.907 | <0.001 | 154 | 79 |
| **Right inferior temporal gyrus** | -0.137 | 0.152 | [-0.434 - 0.161] | -0.741 | 0.369 | 0.907 | <0.001 | 154 | 80 |
| **Right precentral gyrus** | -0.132 | 0.202 | [-0.527 - 0.264] | -0.783 | 0.515 | 0.907 | 37.578 | 153 | 80 |
| **Left precuneus** | -0.123 | 0.394 | [-0.895 - 0.648] | -0.588 | 0.754 | 0.907 | 82.683 | 154 | 80 |
| **Right pars opercularis** | -0.114 | 0.186 | [-0.478 - 0.25] | -0.572 | 0.540 | 0.907 | 27.612 | 153 | 80 |
| **Left banks superior temporal sulcus** | -0.112 | 0.310 | [-0.719 - 0.495] | -0.822 | 0.717 | 0.907 | 70.890 | 150 | 74 |
| **Left pars orbitalis** | -0.111 | 0.270 | [-0.641 - 0.419] | -0.812 | 0.681 | 0.907 | 64.017 | 152 | 80 |
| **Left superior parietal cortex** | -0.093 | 0.197 | [-0.479 - 0.294] | -0.454 | 0.639 | 0.907 | 34.675 | 154 | 80 |
| **Left inferior temporal gyrus** | -0.092 | 0.153 | [-0.391 - 0.207] | -0.516 | 0.545 | 0.907 | <0.001 | 153 | 80 |
| **Left middle temporal gyrus** | -0.091 | 0.194 | [-0.472 - 0.289] | -0.543 | 0.638 | 0.907 | 30.447 | 150 | 77 |
| **Right precuneus** | -0.088 | 0.340 | [-0.755 - 0.578] | -0.440 | 0.795 | 0.907 | 77.055 | 154 | 80 |
| **Left posterior cingulate cortex** | -0.085 | 0.206 | [-0.488 - 0.319] | -0.531 | 0.681 | 0.907 | 39.456 | 153 | 80 |
| **Right frontal pole** | -0.083 | 0.152 | [-0.381 - 0.216] | -0.861 | 0.587 | 0.907 | <0.001 | 154 | 80 |
| **Right supramarginal gyrus** | -0.081 | 0.153 | [-0.38 - 0.218] | -0.418 | 0.593 | 0.907 | <0.001 | 151 | 80 |
| **Left frontal pole** | -0.079 | 0.339 | [-0.742 - 0.585] | -0.828 | 0.816 | 0.907 | 76.931 | 154 | 80 |
| **Right inferior parietal cortex** | -0.079 | 0.286 | [-0.64 - 0.483] | -0.445 | 0.784 | 0.907 | 67.897 | 153 | 80 |
| **Left paracentral gyrus** | -0.077 | 0.215 | [-0.498 - 0.344] | -0.438 | 0.720 | 0.907 | 43.895 | 154 | 80 |
| **Left isthmus cingulate cortex** | -0.074 | 0.153 | [-0.374 - 0.227] | -0.566 | 0.631 | 0.907 | <0.001 | 153 | 79 |
| **Left superior temporal gyrus** | -0.072 | 0.173 | [-0.411 - 0.267] | -0.439 | 0.678 | 0.907 | 15.782 | 153 | 76 |
| **Right postcentral gyrus** | -0.067 | 0.154 | [-0.368 - 0.234] | -0.379 | 0.663 | 0.907 | <0.001 | 152 | 79 |
| **Right isthmus cingulate cortex** | -0.055 | 0.152 | [-0.353 - 0.243] | -0.434 | 0.719 | 0.907 | <0.001 | 153 | 80 |
| **Left superior frontal gyrus** | -0.054 | 0.152 | [-0.353 - 0.245] | -0.275 | 0.722 | 0.907 | 0.002 | 153 | 80 |
| **Right superior frontal gyrus** | -0.039 | 0.223 | [-0.475 - 0.398] | -0.188 | 0.862 | 0.928 | 48.079 | 154 | 80 |
| **Right middle temporal gyrus** | -0.036 | 0.155 | [-0.34 - 0.267] | -0.202 | 0.815 | 0.907 | 2.165 | 154 | 79 |
| **Left cuneus** | -0.025 | 0.277 | [-0.567 - 0.517] | -0.153 | 0.928 | 0.966 | 65.489 | 153 | 80 |
| **Left temporal pole** | 0.008 | 0.216 | [-0.415 - 0.432] | 0.085 | 0.969 | 0.969 | 44.233 | 154 | 80 |
| **Right superior parietal cortex** | 0.012 | 0.276 | [-0.528 - 0.553] | 0.062 | 0.964 | 0.969 | 65.542 | 153 | 80 |
| **Right pars triangularis** | 0.018 | 0.235 | [-0.442 - 0.479] | 0.105 | 0.938 | 0.966 | 52.800 | 154 | 80 |
| **Right banks superior temporal sulcus** | 0.021 | 0.269 | [-0.506 - 0.549] | 0.153 | 0.936 | 0.966 | 63.360 | 153 | 77 |
| **Right parahippocampal gyrus** | 0.039 | 0.152 | [-0.259 - 0.337] | 0.427 | 0.797 | 0.907 | <0.001 | 154 | 80 |
| **Right transverse temporal gyrus** | 0.045 | 0.152 | [-0.253 - 0.344] | 0.365 | 0.766 | 0.907 | <0.001 | 154 | 80 |
| **Left rostral middle frontal gyrus** | 0.047 | 0.152 | [-0.252 - 0.346] | 0.228 | 0.758 | 0.907 | 0.001 | 154 | 80 |
| **Right posterior cingulate cortex** | 0.047 | 0.160 | [-0.267 - 0.361] | 0.316 | 0.768 | 0.907 | 7.206 | 154 | 80 |
| **Right rostral middle frontal gyrus** | 0.047 | 0.265 | [-0.472 - 0.566] | 0.228 | 0.858 | 0.928 | 62.702 | 153 | 80 |
| **Right paracentral gyrus** | 0.063 | 0.153 | [-0.236 - 0.362] | 0.356 | 0.681 | 0.907 | <0.001 | 151 | 80 |
| **Left transverse temporal gyrus** | 0.063 | 0.152 | [-0.236 - 0.361] | 0.495 | 0.680 | 0.907 | <0.001 | 154 | 80 |
| **Left lingual gyrus** | 0.066 | 0.152 | [-0.232 - 0.364] | 0.381 | 0.665 | 0.907 | <0.001 | 154 | 80 |
| **Right lingual gyrus** | 0.067 | 0.152 | [-0.231 - 0.365] | 0.388 | 0.660 | 0.907 | <0.001 | 154 | 80 |
| **Right caudal anterior cingulate cortex** | 0.069 | 0.171 | [-0.266 - 0.404] | 0.766 | 0.688 | 0.907 | 16.480 | 154 | 80 |
| **Left pericalcarine cortex** | 0.072 | 0.152 | [-0.227 - 0.37] | 0.504 | 0.638 | 0.907 | <0.001 | 154 | 80 |
| **Right pericalcarine cortex** | 0.090 | 0.152 | [-0.208 - 0.388] | 0.657 | 0.554 | 0.907 | <0.001 | 154 | 80 |
| **Left entorhinal cortex** | 0.103 | 0.153 | [-0.197 - 0.403] | 1.183 | 0.502 | 0.907 | <0.001 | 154 | 79 |
| **Right temporal pole** | 0.154 | 0.346 | [-0.523 - 0.832] | 1.700 | 0.655 | 0.907 | 77.566 | 154 | 80 |
| **Left caudal middle frontal gyrus** | 0.175 | 0.152 | [-0.124 - 0.473] | 0.890 | 0.252 | 0.907 | 0.005 | 154 | 80 |
| **Left pars opercularis** | 0.189 | 0.153 | [-0.112 - 0.49] | 0.902 | 0.217 | 0.907 | <0.001 | 153 | 79 |
| **Left pars triangularis** | 0.249 | 0.153 | [-0.05 - 0.549] | 1.516 | 0.103 | 0.907 | <0.001 | 153 | 80 |
| **Right caudal middle frontal gyrus** | 0.269 | 0.326 | [-0.369 - 0.907] | 1.415 | 0.409 | 0.907 | 74.732 | 154 | 78 |

**^a^** Included Samples: MMDP 3T, Melbourne, Muenster Cohort, QTIM, Sydney.

MDD: Major Depressive Disorder; CTL: Controls.

**Supplementary Table S38**: Full meta-analytic results for thickness of each structure for recurrent episode MDD patients versus Controls comparison controlling for age, sex and scan center. Adjusted Cohen's d is reported.

|  | **Cohen's d ^a^** | **Std. Err.** | **95% CI** | **% Difference** | **P-value** | **FDR P-value** | **I^2^** | **# Controls** | **# Patients** |
| --- | --- | --- | --- | --- | --- | --- | --- | --- | --- |
|  | **(Recurrent MDD vs CTL)** |  |  |  |  |  |  |  |  |
| **Left lateral orbitofrontal cortex** | -0.486 | 0.154 | [-0.788 - -0.183] | -2.932 | 0.002 | 0.116 | <0.001 | 142 | 104 |
| **Left isthmus cingulate cortex** | -0.343 | 0.154 | [-0.645 - -0.042] | -2.637 | 0.026 | 0.901 | <0.001 | 141 | 104 |
| **Left frontal pole** | -0.323 | 0.379 | [-1.065 - 0.419] | -3.403 | 0.393 | 0.989 | 80.949 | 142 | 104 |
| **Right lateral orbitofrontal cortex** | -0.261 | 0.155 | [-0.564 - 0.043] | -1.533 | 0.092 | 0.989 | <0.001 | 142 | 102 |
| **Right pars opercularis** | -0.254 | 0.154 | [-0.555 - 0.048] | -1.271 | 0.099 | 0.989 | 0.002 | 141 | 104 |
| **Left parahippocampal gyrus** | -0.235 | 0.154 | [-0.536 - 0.067] | -2.920 | 0.128 | 0.989 | <0.001 | 142 | 103 |
| **Left medial orbitofrontal cortex** | -0.228 | 0.239 | [-0.697 - 0.241] | -1.767 | 0.341 | 0.989 | 53.737 | 142 | 104 |
| **Left caudal anterior cingulate cortex** | -0.196 | 0.300 | [-0.783 - 0.391] | -2.221 | 0.514 | 0.989 | 69.963 | 140 | 104 |
| **Left paracentral gyrus** | -0.192 | 0.154 | [-0.494 - 0.109] | -1.094 | 0.212 | 0.989 | <0.001 | 142 | 103 |
| **Right isthmus cingulate cortex** | -0.187 | 0.153 | [-0.487 - 0.114] | -1.478 | 0.224 | 0.989 | <0.001 | 141 | 104 |
| **Left rostral anterior cingulate cortex** | -0.185 | 0.320 | [-0.813 - 0.442] | -1.805 | 0.563 | 0.989 | 73.730 | 142 | 104 |
| **Right medial orbitofrontal cortex** | -0.173 | 0.175 | [-0.516 - 0.17] | -1.450 | 0.324 | 0.989 | 18.497 | 141 | 104 |
| **Left pars orbitalis** | -0.160 | 0.334 | [-0.814 - 0.495] | -1.168 | 0.633 | 0.989 | 75.800 | 140 | 104 |
| **Right parahippocampal gyrus** | -0.153 | 0.219 | [-0.582 - 0.276] | -1.664 | 0.485 | 0.989 | 43.811 | 142 | 103 |
| **Left rostral middle frontal gyrus** | -0.140 | 0.153 | [-0.44 - 0.16] | -0.680 | 0.359 | 0.989 | <0.001 | 142 | 104 |
| **Right precentral gyrus** | -0.137 | 0.153 | [-0.438 - 0.163] | -0.817 | 0.370 | 0.989 | <0.001 | 141 | 104 |
| **Left precentral gyrus** | -0.136 | 0.154 | [-0.439 - 0.166] | -0.782 | 0.377 | 0.989 | <0.001 | 139 | 103 |
| **Right frontal pole** | -0.136 | 0.153 | [-0.435 - 0.164] | -1.415 | 0.375 | 0.989 | <0.001 | 142 | 104 |
| **Left caudal middle frontal gyrus** | -0.115 | 0.153 | [-0.415 - 0.185] | -0.587 | 0.452 | 0.989 | 0.002 | 142 | 104 |
| **Left superior frontal gyrus** | -0.098 | 0.205 | [-0.5 - 0.303] | -0.497 | 0.631 | 0.989 | 37.799 | 141 | 103 |
| **Right postcentral gyrus** | -0.095 | 0.224 | [-0.533 - 0.344] | -0.536 | 0.672 | 0.989 | 47.397 | 140 | 104 |
| **Right inferior temporal gyrus** | -0.090 | 0.209 | [-0.499 - 0.319] | -0.491 | 0.665 | 0.989 | 40.259 | 142 | 104 |
| **Left superior temporal gyrus** | -0.090 | 0.219 | [-0.518 - 0.339] | -0.549 | 0.681 | 0.989 | 43.565 | 141 | 97 |
| **Right inferior parietal cortex** | -0.089 | 0.225 | [-0.531 - 0.352] | -0.507 | 0.691 | 0.989 | 48.117 | 141 | 104 |
| **Right fusiform gyrus** | -0.089 | 0.349 | [-0.772 - 0.595] | -0.432 | 0.799 | 0.989 | 77.583 | 142 | 102 |
| **Right cuneus** | -0.082 | 0.153 | [-0.382 - 0.219] | -0.502 | 0.594 | 0.989 | <0.001 | 140 | 104 |
| **Right rostral anterior cingulate cortex** | -0.069 | 0.154 | [-0.371 - 0.233] | -0.699 | 0.654 | 0.989 | 0.007 | 141 | 103 |
| **Right paracentral gyrus** | -0.057 | 0.154 | [-0.358 - 0.244] | -0.324 | 0.710 | 0.989 | <0.001 | 139 | 104 |
| **Left posterior cingulate cortex** | -0.053 | 0.178 | [-0.402 - 0.295] | -0.335 | 0.764 | 0.989 | 21.033 | 141 | 104 |
| **Right pars triangularis** | -0.051 | 0.178 | [-0.399 - 0.298] | -0.292 | 0.776 | 0.989 | 21.129 | 142 | 104 |
| **Right banks superior temporal sulcus** | -0.049 | 0.154 | [-0.35 - 0.253] | -0.346 | 0.752 | 0.989 | 0.003 | 141 | 97 |
| **Left insula** | -0.048 | 0.244 | [-0.527 - 0.431] | -0.249 | 0.845 | 0.989 | 55.620 | 140 | 104 |
| **Left hempisphere average thickness** | -0.043 | 0.203 | [-0.441 - 0.354] | -0.159 | 0.831 | 0.989 | 37.151 | 142 | 104 |
| **Right entorhinal cortex** | -0.032 | 0.158 | [-0.342 - 0.277] | -0.383 | 0.838 | 0.989 | 0.009 | 139 | 102 |
| **Right hemisphere average thickness** | -0.031 | 0.232 | [-0.487 - 0.425] | -0.113 | 0.894 | 0.989 | 51.247 | 142 | 104 |
| **Left temporal pole** | -0.018 | 0.242 | [-0.493 - 0.456] | -0.187 | 0.939 | 0.989 | 54.999 | 142 | 104 |
| **Left superior parietal cortex** | -0.017 | 0.153 | [-0.317 - 0.282] | -0.084 | 0.911 | 0.989 | <0.001 | 142 | 104 |
| **Right superior parietal cortex** | -0.011 | 0.153 | [-0.311 - 0.29] | -0.054 | 0.944 | 0.989 | <0.001 | 141 | 104 |
| **Right pars orbitalis** | -0.010 | 0.291 | [-0.58 - 0.56] | -0.072 | 0.973 | 0.989 | 68.049 | 142 | 102 |
| **Right middle temporal gyrus** | -0.004 | 0.198 | [-0.393 - 0.384] | -0.025 | 0.982 | 0.989 | 34.028 | 142 | 98 |
| **Left postcentral gyrus** | 0.002 | 0.153 | [-0.298 - 0.302] | 0.011 | 0.989 | 0.989 | 0.001 | 142 | 104 |
| **Right pericalcarine cortex** | 0.007 | 0.154 | [-0.295 - 0.308] | 0.048 | 0.966 | 0.989 | <0.001 | 142 | 103 |
| **Right precuneus** | 0.009 | 0.211 | [-0.405 - 0.423] | 0.044 | 0.966 | 0.989 | 41.787 | 142 | 104 |
| **Left banks superior temporal sulcus** | 0.013 | 0.286 | [-0.548 - 0.574] | 0.094 | 0.964 | 0.989 | 64.775 | 138 | 91 |
| **Right superior temporal gyrus** | 0.015 | 0.214 | [-0.404 - 0.434] | 0.092 | 0.942 | 0.989 | 42.660 | 142 | 100 |
| **Left precuneus** | 0.016 | 0.286 | [-0.545 - 0.577] | 0.077 | 0.955 | 0.989 | 67.356 | 142 | 104 |
| **Left pericalcarine cortex** | 0.028 | 0.163 | [-0.292 - 0.348] | 0.196 | 0.864 | 0.989 | 9.220 | 142 | 104 |
| **Right temporal pole** | 0.037 | 0.194 | [-0.343 - 0.417] | 0.405 | 0.849 | 0.989 | 31.812 | 142 | 104 |
| **Right superior frontal gyrus** | 0.038 | 0.153 | [-0.262 - 0.338] | 0.183 | 0.806 | 0.989 | <0.001 | 142 | 104 |
| **Left supramarginal gyrus** | 0.039 | 0.154 | [-0.262 - 0.34] | 0.210 | 0.800 | 0.989 | <0.001 | 139 | 104 |
| **Left middle temporal gyrus** | 0.039 | 0.209 | [-0.371 - 0.449] | 0.232 | 0.852 | 0.989 | 36.470 | 138 | 93 |
| **Left lingual gyrus** | 0.044 | 0.153 | [-0.256 - 0.343] | 0.253 | 0.775 | 0.989 | <0.001 | 142 | 104 |
| **Right insula** | 0.050 | 0.154 | [-0.251 - 0.351] | 0.272 | 0.745 | 0.989 | <0.001 | 139 | 104 |
| **Left fusiform gyrus** | 0.069 | 0.236 | [-0.393 - 0.531] | 0.338 | 0.770 | 0.989 | 52.458 | 141 | 104 |
| **Left pars opercularis** | 0.071 | 0.154 | [-0.231 - 0.372] | 0.337 | 0.646 | 0.989 | <0.001 | 141 | 103 |
| **Right lingual gyrus** | 0.108 | 0.153 | [-0.192 - 0.408] | 0.625 | 0.481 | 0.989 | <0.001 | 142 | 104 |
| **Right posterior cingulate cortex** | 0.108 | 0.153 | [-0.192 - 0.409] | 0.726 | 0.479 | 0.989 | 0.002 | 142 | 104 |
| **Right rostral middle frontal gyrus** | 0.110 | 0.153 | [-0.19 - 0.411] | 0.532 | 0.472 | 0.989 | <0.001 | 141 | 104 |
| **Left pars triangularis** | 0.116 | 0.153 | [-0.184 - 0.416] | 0.705 | 0.450 | 0.989 | <0.001 | 141 | 104 |
| **Left inferior parietal cortex** | 0.133 | 0.153 | [-0.167 - 0.433] | 0.756 | 0.384 | 0.989 | <0.001 | 142 | 104 |
| **Right caudal middle frontal gyrus** | 0.145 | 0.188 | [-0.223 - 0.513] | 0.763 | 0.441 | 0.989 | 27.960 | 142 | 104 |
| **Left inferior temporal gyrus** | 0.154 | 0.220 | [-0.277 - 0.584] | 0.858 | 0.485 | 0.989 | 45.612 | 141 | 104 |
| **Right caudal anterior cingulate cortex** | 0.158 | 0.218 | [-0.27 - 0.585] | 1.756 | 0.470 | 0.989 | 44.931 | 142 | 104 |
| **Right supramarginal gyrus** | 0.158 | 0.155 | [-0.145 - 0.461] | 0.813 | 0.306 | 0.989 | <0.001 | 139 | 102 |
| **Left transverse temporal gyrus** | 0.160 | 0.174 | [-0.181 - 0.501] | 1.263 | 0.357 | 0.989 | 17.949 | 142 | 104 |
| **Left entorhinal cortex** | 0.168 | 0.249 | [-0.32 - 0.657] | 1.934 | 0.500 | 0.989 | 57.233 | 142 | 103 |
| **Right lateral occipital cortex** | 0.200 | 0.309 | [-0.406 - 0.806] | 1.172 | 0.517 | 0.989 | 71.863 | 142 | 104 |
| **Left lateral occipital cortex** | 0.228 | 0.254 | [-0.271 - 0.726] | 1.310 | 0.371 | 0.989 | 58.532 | 142 | 103 |
| **Right transverse temporal gyrus** | 0.298 | 0.154 | [-0.003 - 0.599] | 2.396 | 0.052 | 0.989 | 0.002 | 142 | 104 |
| **Left cuneus** | 0.321 | 0.228 | [-0.127 - 0.768] | 1.979 | 0.160 | 0.989 | 49.066 | 141 | 104 |

**^a^** Included Samples: Melbourne, Muenster Cohort, Sydney.

MDD: Major Depressive Disorder; CTL: Controls.

**Supplementary Table S39**: Full meta-analytic results for thickness of each structure for first episode MDD versus recurrent episode MDD patients comparison controlling for age, sex and scan center. Adjusted Cohen's d is reported.

|  | **Cohen's d ^a^** | **Std. Err.** | **95% CI** | **% Difference** | **P-value** | **FDR P-value** | **I^2^** | **#**  **First episode** | **# Recurrent episode** |
| --- | --- | --- | --- | --- | --- | --- | --- | --- | --- |
|  | **(First episode MDD vs Recurrent MDD)** |  |  |  |  |  |  |  |  |
| **Left pars triangularis** | -0.412 | 0.160 | [-0.726 - -0.099] | -2.510 | 0.010 | 0.698 | <0.001 | 67 | 102 |
| **Right caudal middle frontal gyrus** | -0.322 | 0.177 | [-0.669 - 0.025] | -1.695 | 0.069 | 0.928 | 12.351 | 65 | 102 |
| **Left frontal pole** | -0.264 | 0.159 | [-0.576 - 0.048] | -2.782 | 0.097 | 0.928 | <0.001 | 67 | 102 |
| **Left rostral middle frontal gyrus** | -0.243 | 0.159 | [-0.555 - 0.069] | -1.178 | 0.126 | 0.928 | <0.001 | 67 | 102 |
| **Right pericalcarine cortex** | -0.242 | 0.160 | [-0.555 - 0.071] | -1.766 | 0.129 | 0.928 | <0.001 | 67 | 101 |
| **Left caudal middle frontal gyrus** | -0.228 | 0.184 | [-0.588 - 0.132] | -1.163 | 0.215 | 0.928 | 18.872 | 67 | 102 |
| **Left paracentral gyrus** | -0.196 | 0.348 | [-0.878 - 0.485] | -1.119 | 0.572 | 0.933 | 74.756 | 67 | 101 |
| **Left pars orbitalis** | -0.192 | 0.251 | [-0.685 - 0.3] | -1.406 | 0.445 | 0.928 | 53.416 | 67 | 102 |
| **Right banks superior temporal sulcus** | -0.157 | 0.163 | [-0.476 - 0.163] | -1.115 | 0.336 | 0.928 | <0.001 | 64 | 95 |
| **Left lateral orbitofrontal cortex** | -0.156 | 0.159 | [-0.468 - 0.155] | -0.943 | 0.325 | 0.928 | 0.004 | 67 | 102 |
| **Left superior frontal gyrus** | -0.144 | 0.159 | [-0.456 - 0.168] | -0.728 | 0.366 | 0.928 | <0.001 | 67 | 101 |
| **Left entorhinal cortex** | -0.135 | 0.159 | [-0.447 - 0.178] | -1.550 | 0.398 | 0.928 | <0.001 | 66 | 101 |
| **Right insula** | -0.134 | 0.158 | [-0.444 - 0.177] | -0.728 | 0.399 | 0.928 | <0.001 | 67 | 102 |
| **Left medial orbitofrontal cortex** | -0.131 | 0.159 | [-0.443 - 0.182] | -1.013 | 0.412 | 0.928 | <0.001 | 66 | 102 |
| **Right postcentral gyrus** | -0.130 | 0.160 | [-0.442 - 0.183] | -0.733 | 0.417 | 0.928 | 0.001 | 66 | 102 |
| **Right inferior parietal cortex** | -0.126 | 0.159 | [-0.436 - 0.185] | -0.712 | 0.428 | 0.928 | <0.001 | 67 | 102 |
| **Left superior temporal gyrus** | -0.122 | 0.164 | [-0.443 - 0.198] | -0.746 | 0.455 | 0.928 | <0.001 | 63 | 95 |
| **Right transverse temporal gyrus** | -0.116 | 0.159 | [-0.428 - 0.195] | -0.937 | 0.464 | 0.928 | <0.001 | 67 | 102 |
| **Left middle temporal gyrus** | -0.108 | 0.164 | [-0.43 - 0.214] | -0.639 | 0.512 | 0.933 | <0.001 | 64 | 91 |
| **Left transverse temporal gyrus** | -0.092 | 0.158 | [-0.403 - 0.218] | -0.726 | 0.561 | 0.933 | <0.001 | 67 | 102 |
| **Right inferior temporal gyrus** | -0.084 | 0.159 | [-0.396 - 0.228] | -0.458 | 0.596 | 0.933 | 0.008 | 67 | 102 |
| **Right superior frontal gyrus** | -0.083 | 0.159 | [-0.394 - 0.228] | -0.403 | 0.600 | 0.933 | <0.001 | 67 | 102 |
| **Left insula** | -0.079 | 0.282 | [-0.631 - 0.473] | -0.412 | 0.780 | 0.970 | 62.727 | 67 | 102 |
| **Right cuneus** | -0.068 | 0.183 | [-0.426 - 0.29] | -0.420 | 0.708 | 0.970 | 19.215 | 67 | 102 |
| **Right superior parietal cortex** | -0.056 | 0.159 | [-0.367 - 0.255] | -0.281 | 0.722 | 0.970 | <0.001 | 67 | 102 |
| **Left lingual gyrus** | -0.054 | 0.158 | [-0.364 - 0.256] | -0.312 | 0.733 | 0.970 | <0.001 | 67 | 102 |
| **Left temporal pole** | -0.044 | 0.159 | [-0.355 - 0.267] | -0.445 | 0.781 | 0.970 | <0.001 | 67 | 102 |
| **Right rostral middle frontal gyrus** | -0.041 | 0.158 | [-0.351 - 0.269] | -0.197 | 0.796 | 0.970 | <0.001 | 67 | 102 |
| **Left pericalcarine cortex** | -0.029 | 0.209 | [-0.44 - 0.381] | -0.207 | 0.888 | 0.970 | 35.965 | 67 | 102 |
| **Left pars opercularis** | -0.029 | 0.160 | [-0.342 - 0.284] | -0.137 | 0.857 | 0.970 | <0.001 | 66 | 101 |
| **Left hempisphere average thickness** | -0.026 | 0.158 | [-0.336 - 0.285] | -0.094 | 0.871 | 0.970 | <0.001 | 67 | 102 |
| **Right frontal pole** | -0.022 | 0.247 | [-0.505 - 0.462] | -0.225 | 0.931 | 0.970 | 52.279 | 67 | 102 |
| **Right supramarginal gyrus** | -0.015 | 0.171 | [-0.349 - 0.319] | -0.077 | 0.930 | 0.970 | 9.731 | 67 | 100 |
| **Right parahippocampal gyrus** | -0.004 | 0.159 | [-0.316 - 0.307] | -0.045 | 0.979 | 0.989 | <0.001 | 67 | 101 |
| **Right pars orbitalis** | 0.003 | 0.190 | [-0.369 - 0.374] | 0.019 | 0.989 | 0.989 | 22.941 | 66 | 100 |
| **Left postcentral gyrus** | 0.012 | 0.172 | [-0.324 - 0.349] | 0.068 | 0.943 | 0.970 | 10.219 | 66 | 102 |
| **Left inferior temporal gyrus** | 0.012 | 0.159 | [-0.298 - 0.323] | 0.070 | 0.937 | 0.970 | <0.001 | 67 | 102 |
| **Left caudal anterior cingulate cortex** | 0.018 | 0.158 | [-0.292 - 0.328] | 0.205 | 0.909 | 0.970 | <0.001 | 67 | 102 |
| **Right precentral gyrus** | 0.020 | 0.159 | [-0.291 - 0.332] | 0.121 | 0.898 | 0.970 | 0.003 | 67 | 102 |
| **Right hemisphere average thickness** | 0.026 | 0.158 | [-0.284 - 0.336] | 0.096 | 0.869 | 0.970 | <0.001 | 67 | 102 |
| **Left banks superior temporal sulcus** | 0.031 | 0.167 | [-0.297 - 0.359] | 0.226 | 0.854 | 0.970 | <0.001 | 61 | 89 |
| **Right paracentral gyrus** | 0.046 | 0.159 | [-0.266 - 0.358] | 0.263 | 0.771 | 0.970 | 0.001 | 67 | 102 |
| **Right lingual gyrus** | 0.050 | 0.159 | [-0.261 - 0.36] | 0.288 | 0.754 | 0.970 | <0.001 | 67 | 102 |
| **Left precentral gyrus** | 0.050 | 0.159 | [-0.261 - 0.361] | 0.287 | 0.753 | 0.970 | <0.001 | 67 | 101 |
| **Right precuneus** | 0.066 | 0.159 | [-0.245 - 0.378] | 0.330 | 0.676 | 0.970 | <0.001 | 67 | 102 |
| **Right medial orbitofrontal cortex** | 0.075 | 0.158 | [-0.236 - 0.385] | 0.628 | 0.637 | 0.948 | <0.001 | 67 | 102 |
| **Right pars triangularis** | 0.093 | 0.159 | [-0.218 - 0.403] | 0.534 | 0.559 | 0.933 | <0.001 | 67 | 102 |
| **Right entorhinal cortex** | 0.098 | 0.160 | [-0.216 - 0.412] | 1.165 | 0.541 | 0.933 | <0.001 | 66 | 100 |
| **Right superior temporal gyrus** | 0.104 | 0.160 | [-0.211 - 0.418] | 0.621 | 0.518 | 0.933 | <0.001 | 66 | 98 |
| **Right lateral orbitofrontal cortex** | 0.109 | 0.159 | [-0.203 - 0.422] | 0.642 | 0.493 | 0.933 | <0.001 | 67 | 100 |
| **Right middle temporal gyrus** | 0.112 | 0.199 | [-0.277 - 0.501] | 0.624 | 0.573 | 0.933 | 28.390 | 66 | 96 |
| **Left lateral occipital cortex** | 0.112 | 0.224 | [-0.327 - 0.551] | 0.645 | 0.617 | 0.939 | 42.328 | 67 | 101 |
| **Left posterior cingulate cortex** | 0.121 | 0.159 | [-0.191 - 0.433] | 0.758 | 0.447 | 0.928 | 0.003 | 67 | 102 |
| **Left inferior parietal cortex** | 0.127 | 0.158 | [-0.184 - 0.437] | 0.718 | 0.425 | 0.928 | <0.001 | 67 | 102 |
| **Left fusiform gyrus** | 0.133 | 0.159 | [-0.179 - 0.444] | 0.651 | 0.404 | 0.928 | 0.007 | 67 | 102 |
| **Left isthmus cingulate cortex** | 0.134 | 0.176 | [-0.211 - 0.479] | 1.030 | 0.446 | 0.928 | 13.454 | 66 | 102 |
| **Right pars opercularis** | 0.139 | 0.159 | [-0.172 - 0.45] | 0.695 | 0.382 | 0.928 | <0.001 | 67 | 102 |
| **Left parahippocampal gyrus** | 0.146 | 0.160 | [-0.167 - 0.459] | 1.815 | 0.361 | 0.928 | <0.001 | 66 | 101 |
| **Left rostral anterior cingulate cortex** | 0.174 | 0.159 | [-0.137 - 0.485] | 1.693 | 0.273 | 0.928 | <0.001 | 67 | 102 |
| **Left precuneus** | 0.183 | 0.159 | [-0.129 - 0.495] | 0.874 | 0.250 | 0.928 | 0.003 | 67 | 102 |
| **Right lateral occipital cortex** | 0.191 | 0.159 | [-0.121 - 0.503] | 1.118 | 0.229 | 0.928 | 0.003 | 67 | 102 |
| **Left cuneus** | 0.192 | 0.159 | [-0.12 - 0.504] | 1.182 | 0.228 | 0.928 | <0.001 | 67 | 102 |
| **Left superior parietal cortex** | 0.195 | 0.159 | [-0.116 - 0.506] | 0.955 | 0.219 | 0.928 | <0.001 | 67 | 102 |
| **Left supramarginal gyrus** | 0.204 | 0.159 | [-0.107 - 0.516] | 1.099 | 0.199 | 0.928 | <0.001 | 67 | 102 |
| **Right temporal pole** | 0.214 | 0.159 | [-0.097 - 0.525] | 2.350 | 0.178 | 0.928 | <0.001 | 67 | 102 |
| **Right fusiform gyrus** | 0.237 | 0.160 | [-0.076 - 0.55] | 1.156 | 0.138 | 0.928 | <0.001 | 67 | 100 |
| **Right rostral anterior cingulate cortex** | 0.240 | 0.170 | [-0.093 - 0.573] | 2.436 | 0.158 | 0.928 | 8.921 | 67 | 101 |
| **Right isthmus cingulate cortex** | 0.252 | 0.182 | [-0.105 - 0.609] | 1.995 | 0.167 | 0.928 | 18.559 | 67 | 102 |
| **Right caudal anterior cingulate cortex** | 0.278 | 0.194 | [-0.103 - 0.658] | 3.091 | 0.153 | 0.928 | 26.408 | 67 | 102 |
| **Right posterior cingulate cortex** | 0.299 | 0.159 | [-0.013 - 0.611] | 1.999 | 0.061 | 0.928 | <0.001 | 67 | 102 |

**^a^** Included Samples: Melbourne, Muenster Cohort, Sydney.

MDD: Major Depressive Disorder.

**Supplementary Table S40**: Full meta-analytic results for thickness of each structure for the association with number of episodes in recurrent episode MDD patients controlling for age, sex and scan center. Adjusted Cohen's d is reported.

|  | **Pearson's r ^a^** | **Std. Err.** | **95% CI** | **% Difference** | **P-value** | **FDR P-value** | **I^2^** | **# Patients** |
| --- | --- | --- | --- | --- | --- | --- | --- | --- |
|  | **(#Episodes in Recurrent MDD)** |  |  |  |  |  |  |  |
| **Left isthmus cingulate cortex** | -0.294 | 0.237 | [0.214 - -0.758] | -4.733 | 0.214 | 0.770 | 82.584 | 70 |
| **Left frontal pole** | -0.258 | 0.225 | [0.251 - -0.698] | -5.622 | 0.251 | 0.770 | 78.277 | 70 |
| **Right isthmus cingulate cortex** | -0.244 | 0.282 | [0.388 - -0.797] | -3.981 | 0.388 | 0.770 | 88.424 | 70 |
| **Right entorhinal cortex** | -0.228 | 0.208 | [0.271 - -0.635] | -5.577 | 0.271 | 0.770 | 70.934 | 69 |
| **Right supramarginal gyrus** | -0.202 | 0.188 | [0.281 - -0.57] | -2.120 | 0.281 | 0.770 | 62.359 | 69 |
| **Right frontal pole** | -0.177 | 0.226 | [0.432 - -0.62] | -3.760 | 0.432 | 0.770 | 75.946 | 70 |
| **Right insula** | -0.177 | 0.199 | [0.375 - -0.568] | -1.959 | 0.375 | 0.770 | 67.442 | 70 |
| **Right temporal pole** | -0.136 | 0.120 | [0.254 - -0.371] | -3.032 | 0.254 | 0.770 | <0.001 | 70 |
| **Right parahippocampal gyrus** | -0.135 | 0.121 | [0.262 - -0.372] | -2.980 | 0.262 | 0.770 | <0.001 | 69 |
| **Left entorhinal cortex** | -0.120 | 0.121 | [0.321 - -0.358] | -2.785 | 0.321 | 0.770 | <0.001 | 69 |
| **Left transverse temporal gyrus** | -0.106 | 0.213 | [0.617 - -0.523] | -1.686 | 0.617 | 0.785 | 70.499 | 70 |
| **Left pars opercularis** | -0.095 | 0.121 | [0.428 - -0.332] | -0.914 | 0.428 | 0.770 | <0.001 | 69 |
| **Right rostral middle frontal gyrus** | -0.087 | 0.118 | [0.458 - -0.318] | -0.845 | 0.458 | 0.770 | 3.153 | 70 |
| **Left inferior parietal cortex** | -0.084 | 0.167 | [0.614 - -0.411] | -0.959 | 0.614 | 0.785 | 47.897 | 70 |
| **Right inferior parietal cortex** | -0.082 | 0.117 | [0.484 - -0.312] | -0.932 | 0.484 | 0.770 | <0.001 | 70 |
| **Right precuneus** | -0.072 | 0.121 | [0.553 - -0.309] | -0.717 | 0.553 | 0.785 | <0.001 | 70 |
| **Right middle temporal gyrus** | -0.069 | 0.124 | [0.579 - -0.313] | -0.773 | 0.579 | 0.785 | <0.001 | 66 |
| **Right postcentral gyrus** | -0.036 | 0.122 | [0.767 - -0.275] | -0.408 | 0.767 | 0.892 | <0.001 | 70 |
| **Left caudal anterior cingulate cortex** | -0.032 | 0.243 | [0.896 - -0.509] | -0.727 | 0.896 | 0.950 | 78.090 | 70 |
| **Left insula** | -0.027 | 0.200 | [0.892 - -0.419] | -0.283 | 0.892 | 0.950 | 64.429 | 70 |
| **Left postcentral gyrus** | -0.019 | 0.121 | [0.875 - -0.257] | -0.210 | 0.875 | 0.950 | <0.001 | 70 |
| **Left pars orbitalis** | -0.019 | 0.126 | [0.882 - -0.266] | -0.274 | 0.882 | 0.950 | 12.495 | 70 |
| **Right lateral orbitofrontal cortex** | -0.012 | 0.121 | [0.924 - -0.25] | -0.137 | 0.924 | 0.965 | <0.001 | 68 |
| **Left parahippocampal gyrus** | -0.008 | 0.130 | [0.95 - -0.264] | -0.203 | 0.950 | 0.968 | 12.633 | 69 |
| **Left middle temporal gyrus** | 0.003 | 0.128 | [0.98 - -0.247] | 0.039 | 0.980 | 0.980 | <0.001 | 64 |
| **Left superior frontal gyrus** | 0.014 | 0.243 | [0.954 - -0.463] | 0.140 | 0.954 | 0.968 | 77.542 | 69 |
| **Left posterior cingulate cortex** | 0.021 | 0.122 | [0.863 - -0.218] | 0.264 | 0.863 | 0.950 | <0.001 | 70 |
| **Right posterior cingulate cortex** | 0.034 | 0.119 | [0.777 - -0.2] | 0.452 | 0.777 | 0.892 | <0.001 | 70 |
| **Left fusiform gyrus** | 0.037 | 0.121 | [0.76 - -0.201] | 0.365 | 0.760 | 0.892 | <0.001 | 70 |
| **Left precuneus** | 0.040 | 0.120 | [0.74 - -0.196] | 0.382 | 0.740 | 0.892 | <0.001 | 70 |
| **Right superior temporal gyrus** | 0.042 | 0.123 | [0.735 - -0.2] | 0.499 | 0.735 | 0.892 | <0.001 | 68 |
| **Right caudal anterior cingulate cortex** | 0.054 | 0.121 | [0.654 - -0.183] | 1.214 | 0.654 | 0.817 | <0.001 | 70 |
| **Right superior parietal cortex** | 0.063 | 0.125 | [0.616 - -0.182] | 0.627 | 0.616 | 0.785 | 11.441 | 70 |
| **Left supramarginal gyrus** | 0.065 | 0.121 | [0.589 - -0.171] | 0.702 | 0.589 | 0.785 | <0.001 | 70 |
| **Left rostral anterior cingulate cortex** | 0.066 | 0.121 | [0.588 - -0.171] | 1.279 | 0.588 | 0.785 | <0.001 | 70 |
| **Right transverse temporal gyrus** | 0.071 | 0.121 | [0.554 - -0.165] | 1.152 | 0.554 | 0.785 | <0.001 | 70 |
| **Left medial orbitofrontal cortex** | 0.072 | 0.121 | [0.552 - -0.165] | 1.117 | 0.552 | 0.785 | <0.001 | 70 |
| **Right lateral occipital cortex** | 0.080 | 0.134 | [0.549 - -0.183] | 0.942 | 0.549 | 0.785 | 22.134 | 70 |
| **Right pars orbitalis** | 0.089 | 0.127 | [0.484 - -0.16] | 1.283 | 0.484 | 0.770 | 8.678 | 68 |
| **Left cuneus** | 0.089 | 0.120 | [0.457 - -0.146] | 1.104 | 0.457 | 0.770 | <0.001 | 70 |
| **Left lingual gyrus** | 0.103 | 0.119 | [0.387 - -0.13] | 1.197 | 0.387 | 0.770 | <0.001 | 70 |
| **Right hemisphere average thickness** | 0.103 | 0.116 | [0.373 - -0.124] | 0.757 | 0.373 | 0.770 | <0.001 | 70 |
| **Left caudal middle frontal gyrus** | 0.103 | 0.119 | [0.385 - -0.13] | 1.061 | 0.385 | 0.770 | <0.001 | 70 |
| **Left banks superior temporal sulcus** | 0.107 | 0.127 | [0.401 - -0.143] | 1.576 | 0.401 | 0.770 | <0.001 | 63 |
| **Right banks superior temporal sulcus** | 0.118 | 0.144 | [0.414 - -0.165] | 1.684 | 0.414 | 0.770 | 26.461 | 66 |
| **Left inferior temporal gyrus** | 0.118 | 0.117 | [0.312 - -0.111] | 1.328 | 0.312 | 0.770 | <0.001 | 70 |
| **Right pericalcarine cortex** | 0.124 | 0.119 | [0.3 - -0.11] | 1.816 | 0.300 | 0.770 | <0.001 | 69 |
| **Right superior frontal gyrus** | 0.124 | 0.147 | [0.399 - -0.164] | 1.209 | 0.399 | 0.770 | 33.403 | 70 |
| **Left pars triangularis** | 0.124 | 0.236 | [0.6 - -0.338] | 1.519 | 0.600 | 0.785 | 77.615 | 70 |
| **Right medial orbitofrontal cortex** | 0.124 | 0.120 | [0.301 - -0.111] | 2.103 | 0.301 | 0.770 | <0.001 | 70 |
| **Left superior parietal cortex** | 0.125 | 0.176 | [0.476 - -0.219] | 1.236 | 0.476 | 0.770 | 54.902 | 70 |
| **Left temporal pole** | 0.134 | 0.120 | [0.265 - -0.101] | 2.726 | 0.265 | 0.770 | 3.098 | 70 |
| **Left hempisphere average thickness** | 0.154 | 0.117 | [0.191 - -0.076] | 1.142 | 0.191 | 0.770 | <0.001 | 70 |
| **Left rostral middle frontal gyrus** | 0.154 | 0.118 | [0.191 - -0.077] | 1.515 | 0.191 | 0.770 | <0.001 | 70 |
| **Left lateral orbitofrontal cortex** | 0.155 | 0.116 | [0.182 - -0.073] | 1.889 | 0.182 | 0.770 | <0.001 | 70 |
| **Left superior temporal gyrus** | 0.156 | 0.121 | [0.2 - -0.082] | 1.926 | 0.200 | 0.770 | <0.001 | 65 |
| **Right paracentral gyrus** | 0.165 | 0.119 | [0.166 - -0.068] | 1.895 | 0.166 | 0.770 | <0.001 | 70 |
| **Right cuneus** | 0.184 | 0.251 | [0.465 - -0.309] | 2.296 | 0.465 | 0.770 | 82.267 | 70 |
| **Right caudal middle frontal gyrus** | 0.185 | 0.148 | [0.211 - -0.105] | 1.983 | 0.211 | 0.770 | 37.705 | 70 |
| **Right pars triangularis** | 0.186 | 0.146 | [0.205 - -0.101] | 2.178 | 0.205 | 0.770 | 36.474 | 70 |
| **Right precentral gyrus** | 0.191 | 0.116 | [0.098 - -0.035] | 2.314 | 0.098 | 0.770 | <0.001 | 70 |
| **Left paracentral gyrus** | 0.192 | 0.116 | [0.098 - -0.036] | 2.231 | 0.098 | 0.770 | <0.001 | 69 |
| **Right fusiform gyrus** | 0.196 | 0.118 | [0.097 - -0.036] | 1.948 | 0.097 | 0.770 | 5.713 | 68 |
| **Left precentral gyrus** | 0.200 | 0.115 | [0.081 - -0.025] | 2.339 | 0.081 | 0.770 | <0.001 | 69 |
| **Left pericalcarine cortex** | 0.201 | 0.115 | [0.081 - -0.025] | 2.883 | 0.081 | 0.770 | <0.001 | 70 |
| **Left lateral occipital cortex** | 0.206 | 0.148 | [0.164 - -0.084] | 2.430 | 0.164 | 0.770 | 39.608 | 70 |
| **Right lingual gyrus** | 0.213 | 0.115 | [0.064 - -0.013] | 2.528 | 0.064 | 0.770 | <0.001 | 70 |
| **Right rostral anterior cingulate cortex** | 0.236 | 0.221 | [0.286 - -0.197] | 4.920 | 0.286 | 0.770 | 76.313 | 69 |
| **Right inferior temporal gyrus** | 0.241 | 0.113 | [0.033 - 0.02] | 2.694 | 0.033 | 0.770 | <0.001 | 70 |
| **Right pars opercularis** | 0.256 | 0.114 | [0.024 - 0.034] | 2.659 | 0.024 | 0.770 | <0.001 | 70 |

**^a^** Included Samples: Melbourne, Muenster Cohort, Sydney.

MDD: Major Depressive Disorder.

**Supplementary Table S41**: Full meta-analytic results for thickness of each structure for MDD patients taking antidepressants at time of scanning versus Controls comparison controlling for age, sex and scan center. Adjusted Cohen's d is reported.

|  | **Cohen's d ^a^** | **Std. Err.** | **95% CI** | **% Difference** | **P-value** | **FDR P-value** | **I^2^** | **# Controls** | **# Patients** |
| --- | --- | --- | --- | --- | --- | --- | --- | --- | --- |
|  | **(AD MDD vs CTL)** |  |  |  |  |  |  |  |  |
| **Left isthmus cingulate cortex** | -0.440 | 0.164 | [-0.762 - -0.118] | -3.385 | 0.007 | 0.429 | <0.001 | 141 | 82 |
| **Left lateral orbitofrontal cortex** | -0.411 | 0.164 | [-0.733 - -0.089] | -2.482 | 0.012 | 0.429 | <0.001 | 142 | 82 |
| **Left rostral anterior cingulate cortex** | -0.356 | 0.277 | [-0.899 - 0.188] | -3.463 | 0.200 | 0.989 | 64.146 | 142 | 82 |
| **Right lateral orbitofrontal cortex** | -0.351 | 0.188 | [-0.719 - 0.018] | -2.063 | 0.062 | 0.891 | 22.000 | 142 | 81 |
| **Left parahippocampal gyrus** | -0.310 | 0.164 | [-0.631 - 0.01] | -3.865 | 0.058 | 0.891 | <0.001 | 142 | 82 |
| **Left medial orbitofrontal cortex** | -0.297 | 0.171 | [-0.632 - 0.037] | -2.303 | 0.082 | 0.954 | 7.628 | 142 | 82 |
| **Right entorhinal cortex** | -0.277 | 0.168 | [-0.606 - 0.052] | -3.294 | 0.099 | 0.989 | <0.001 | 139 | 81 |
| **Left frontal pole** | -0.268 | 0.410 | [-1.072 - 0.536] | -2.825 | 0.513 | 0.989 | 83.438 | 142 | 82 |
| **Left insula** | -0.216 | 0.164 | [-0.537 - 0.105] | -1.128 | 0.187 | 0.989 | <0.001 | 140 | 82 |
| **Left caudal anterior cingulate cortex** | -0.200 | 0.242 | [-0.674 - 0.273] | -2.273 | 0.407 | 0.989 | 53.210 | 140 | 82 |
| **Right rostral anterior cingulate cortex** | -0.195 | 0.163 | [-0.515 - 0.125] | -1.979 | 0.232 | 0.989 | <0.001 | 141 | 82 |
| **Right pars opercularis** | -0.182 | 0.163 | [-0.502 - 0.138] | -0.913 | 0.265 | 0.989 | <0.001 | 141 | 82 |
| **Right medial orbitofrontal cortex** | -0.163 | 0.163 | [-0.483 - 0.157] | -1.369 | 0.319 | 0.989 | <0.001 | 141 | 82 |
| **Left fusiform gyrus** | -0.152 | 0.163 | [-0.472 - 0.168] | -0.746 | 0.353 | 0.989 | <0.001 | 141 | 82 |
| **Right cuneus** | -0.151 | 0.163 | [-0.471 - 0.17] | -0.924 | 0.357 | 0.989 | <0.001 | 140 | 82 |
| **Right parahippocampal gyrus** | -0.150 | 0.236 | [-0.612 - 0.312] | -1.634 | 0.525 | 0.989 | 50.297 | 142 | 81 |
| **Left temporal pole** | -0.145 | 0.203 | [-0.543 - 0.253] | -1.463 | 0.476 | 0.989 | 34.394 | 142 | 82 |
| **Right pericalcarine cortex** | -0.143 | 0.164 | [-0.465 - 0.179] | -1.042 | 0.385 | 0.989 | <0.001 | 142 | 81 |
| **Right inferior temporal gyrus** | -0.127 | 0.163 | [-0.447 - 0.193] | -0.689 | 0.437 | 0.989 | <0.001 | 142 | 82 |
| **Right fusiform gyrus** | -0.125 | 0.385 | [-0.879 - 0.629] | -0.610 | 0.745 | 0.989 | 81.312 | 142 | 82 |
| **Right middle temporal gyrus** | -0.119 | 0.164 | [-0.44 - 0.202] | -0.664 | 0.467 | 0.989 | 0.005 | 142 | 79 |
| **Left precentral gyrus** | -0.118 | 0.196 | [-0.503 - 0.267] | -0.678 | 0.547 | 0.989 | 28.738 | 139 | 81 |
| **Right insula** | -0.116 | 0.163 | [-0.436 - 0.204] | -0.630 | 0.479 | 0.989 | <0.001 | 139 | 82 |
| **Right superior temporal gyrus** | -0.090 | 0.163 | [-0.41 - 0.231] | -0.536 | 0.584 | 0.989 | 0.006 | 142 | 81 |
| **Right inferior parietal cortex** | -0.086 | 0.282 | [-0.639 - 0.468] | -0.485 | 0.762 | 0.989 | 65.696 | 141 | 82 |
| **Right isthmus cingulate cortex** | -0.084 | 0.163 | [-0.404 - 0.235] | -0.668 | 0.605 | 0.989 | <0.001 | 141 | 82 |
| **Left pars orbitalis** | -0.080 | 0.273 | [-0.614 - 0.455] | -0.582 | 0.771 | 0.989 | 63.318 | 140 | 82 |
| **Right precuneus** | -0.077 | 0.333 | [-0.729 - 0.576] | -0.381 | 0.818 | 0.989 | 75.260 | 142 | 82 |
| **Right precentral gyrus** | -0.075 | 0.259 | [-0.582 - 0.433] | -0.443 | 0.773 | 0.989 | 59.307 | 141 | 82 |
| **Right pars triangularis** | -0.065 | 0.267 | [-0.589 - 0.459] | -0.375 | 0.808 | 0.989 | 61.927 | 142 | 82 |
| **Left superior temporal gyrus** | -0.065 | 0.281 | [-0.617 - 0.487] | -0.396 | 0.818 | 0.989 | 64.101 | 141 | 77 |
| **Left transverse temporal gyrus** | -0.064 | 0.163 | [-0.384 - 0.256] | -0.502 | 0.696 | 0.989 | 0.006 | 142 | 82 |
| **Left middle temporal gyrus** | -0.062 | 0.168 | [-0.392 - 0.267] | -0.371 | 0.710 | 0.989 | 0.003 | 138 | 75 |
| **Left posterior cingulate cortex** | -0.059 | 0.195 | [-0.44 - 0.323] | -0.368 | 0.763 | 0.989 | 28.873 | 141 | 82 |
| **Left hempisphere average thickness** | -0.053 | 0.289 | [-0.619 - 0.514] | -0.194 | 0.855 | 0.989 | 67.353 | 142 | 82 |
| **Right hemisphere average thickness** | -0.049 | 0.326 | [-0.687 - 0.589] | -0.178 | 0.881 | 0.989 | 74.141 | 142 | 82 |
| **Right postcentral gyrus** | -0.048 | 0.222 | [-0.483 - 0.386] | -0.273 | 0.828 | 0.989 | 44.099 | 140 | 81 |
| **Right temporal pole** | -0.033 | 0.240 | [-0.504 - 0.439] | -0.358 | 0.892 | 0.989 | 53.039 | 142 | 82 |
| **Right banks superior temporal sulcus** | -0.018 | 0.254 | [-0.515 - 0.48] | -0.125 | 0.945 | 0.989 | 56.861 | 141 | 77 |
| **Right frontal pole** | -0.011 | 0.163 | [-0.33 - 0.308] | -0.113 | 0.947 | 0.989 | <0.001 | 142 | 82 |
| **Left inferior temporal gyrus** | -0.006 | 0.164 | [-0.327 - 0.315] | -0.032 | 0.972 | 0.989 | 0.405 | 141 | 82 |
| **Left precuneus** | 0.002 | 0.444 | [-0.868 - 0.872] | 0.011 | 0.996 | 0.996 | 85.898 | 142 | 82 |
| **Right pars orbitalis** | 0.006 | 0.183 | [-0.352 - 0.364] | 0.044 | 0.973 | 0.989 | 18.206 | 142 | 81 |
| **Left superior frontal gyrus** | 0.009 | 0.273 | [-0.527 - 0.544] | 0.044 | 0.974 | 0.989 | 63.479 | 141 | 82 |
| **Left banks superior temporal sulcus** | 0.012 | 0.354 | [-0.683 - 0.706] | 0.086 | 0.974 | 0.989 | 76.481 | 138 | 74 |
| **Right paracentral gyrus** | 0.025 | 0.163 | [-0.295 - 0.346] | 0.143 | 0.877 | 0.989 | <0.001 | 139 | 82 |
| **Left rostral middle frontal gyrus** | 0.030 | 0.163 | [-0.289 - 0.35] | 0.147 | 0.852 | 0.989 | <0.001 | 142 | 82 |
| **Left supramarginal gyrus** | 0.039 | 0.227 | [-0.406 - 0.485] | 0.212 | 0.862 | 0.989 | 47.474 | 139 | 82 |
| **Left postcentral gyrus** | 0.042 | 0.233 | [-0.414 - 0.498] | 0.230 | 0.857 | 0.989 | 49.959 | 142 | 82 |
| **Left pars triangularis** | 0.045 | 0.163 | [-0.274 - 0.365] | 0.276 | 0.781 | 0.989 | <0.001 | 141 | 82 |
| **Left superior parietal cortex** | 0.048 | 0.182 | [-0.308 - 0.405] | 0.237 | 0.790 | 0.989 | 18.754 | 142 | 82 |
| **Left paracentral gyrus** | 0.056 | 0.163 | [-0.264 - 0.376] | 0.318 | 0.732 | 0.989 | <0.001 | 142 | 82 |
| **Left lingual gyrus** | 0.073 | 0.163 | [-0.247 - 0.392] | 0.421 | 0.655 | 0.989 | <0.001 | 142 | 82 |
| **Left pericalcarine cortex** | 0.089 | 0.163 | [-0.231 - 0.409] | 0.627 | 0.585 | 0.989 | <0.001 | 142 | 82 |
| **Left pars opercularis** | 0.090 | 0.166 | [-0.235 - 0.415] | 0.427 | 0.589 | 0.989 | <0.001 | 141 | 80 |
| **Left lateral occipital cortex** | 0.093 | 0.219 | [-0.335 - 0.522] | 0.536 | 0.670 | 0.989 | 42.526 | 142 | 81 |
| **Right supramarginal gyrus** | 0.095 | 0.163 | [-0.225 - 0.415] | 0.486 | 0.562 | 0.989 | <0.001 | 139 | 82 |
| **Right lateral occipital cortex** | 0.096 | 0.339 | [-0.568 - 0.76] | 0.561 | 0.777 | 0.989 | 76.085 | 142 | 82 |
| **Right lingual gyrus** | 0.103 | 0.163 | [-0.217 - 0.422] | 0.595 | 0.529 | 0.989 | <0.001 | 142 | 82 |
| **Right caudal anterior cingulate cortex** | 0.106 | 0.230 | [-0.345 - 0.557] | 1.177 | 0.646 | 0.989 | 48.823 | 142 | 82 |
| **Left inferior parietal cortex** | 0.106 | 0.221 | [-0.327 - 0.54] | 0.605 | 0.630 | 0.989 | 44.652 | 142 | 82 |
| **Left caudal middle frontal gyrus** | 0.107 | 0.163 | [-0.212 - 0.427] | 0.548 | 0.510 | 0.989 | <0.001 | 142 | 82 |
| **Right superior parietal cortex** | 0.107 | 0.290 | [-0.461 - 0.675] | 0.537 | 0.711 | 0.989 | 67.445 | 141 | 82 |
| **Right posterior cingulate cortex** | 0.109 | 0.194 | [-0.271 - 0.488] | 0.727 | 0.575 | 0.989 | 28.162 | 142 | 82 |
| **Left entorhinal cortex** | 0.137 | 0.227 | [-0.308 - 0.582] | 1.579 | 0.545 | 0.989 | 46.640 | 142 | 80 |
| **Right superior frontal gyrus** | 0.154 | 0.234 | [-0.305 - 0.612] | 0.745 | 0.512 | 0.989 | 50.416 | 142 | 82 |
| **Right caudal middle frontal gyrus** | 0.180 | 0.218 | [-0.248 - 0.608] | 0.948 | 0.410 | 0.989 | 43.126 | 142 | 82 |
| **Left cuneus** | 0.213 | 0.196 | [-0.171 - 0.596] | 1.313 | 0.277 | 0.989 | 29.319 | 141 | 82 |
| **Right rostral middle frontal gyrus** | 0.289 | 0.262 | [-0.224 - 0.801] | 1.392 | 0.270 | 0.989 | 59.971 | 141 | 82 |
| **Right transverse temporal gyrus** | 0.304 | 0.164 | [-0.017 - 0.625] | 2.443 | 0.064 | 0.891 | <0.001 | 142 | 82 |

**^a^** Included Samples: Melbourne, Muenster Cohort, Sydney.

AD: antidepressant using; MDD: Major Depressive Disorder; CTL: Controls.

**Supplementary Table S42**: Full meta-analytic results for thickness of each structure for MDD patients not taking antidepressants at time of scanning versus Controls comparison controlling for age, sex and scan center. Adjusted Cohen's d is reported.

|  | **Cohen's d ^a^** | **Std. Err.** | **95% CI** | **% Difference** | **P-value** | **FDR P-value** | **I^2^** | **# Controls** | **# Patients** |
| --- | --- | --- | --- | --- | --- | --- | --- | --- | --- |
|  | **(noAD MDD vs CTL)** |  |  |  |  |  |  |  |  |
| **Right pars opercularis** | -0.338 | 0.130 | [-0.592 - -0.084] | -1.695 | 0.009 | 0.328 | <0.001 | 232 | 119 |
| **Left paracentral gyrus** | -0.322 | 0.130 | [-0.576 - -0.067] | -1.831 | 0.013 | 0.328 | <0.001 | 234 | 118 |
| **Left lateral orbitofrontal cortex** | -0.317 | 0.129 | [-0.57 - -0.064] | -1.914 | 0.014 | 0.328 | <0.001 | 234 | 119 |
| **Right medial orbitofrontal cortex** | -0.309 | 0.157 | [-0.617 - 0] | -2.593 | 0.050 | 0.580 | 23.459 | 222 | 117 |
| **Left precentral gyrus** | -0.288 | 0.130 | [-0.542 - -0.033] | -1.649 | 0.027 | 0.380 | 0.005 | 231 | 119 |
| **Right lateral orbitofrontal cortex** | -0.286 | 0.130 | [-0.54 - -0.032] | -1.684 | 0.027 | 0.380 | <0.001 | 234 | 118 |
| **Left medial orbitofrontal cortex** | -0.254 | 0.234 | [-0.712 - 0.205] | -1.966 | 0.279 | 0.826 | 62.916 | 223 | 116 |
| **Left frontal pole** | -0.253 | 0.254 | [-0.751 - 0.246] | -2.660 | 0.320 | 0.826 | 69.822 | 234 | 119 |
| **Right isthmus cingulate cortex** | -0.217 | 0.129 | [-0.47 - 0.036] | -1.716 | 0.093 | 0.826 | <0.001 | 233 | 119 |
| **Right rostral anterior cingulate cortex** | -0.216 | 0.134 | [-0.48 - 0.047] | -2.194 | 0.108 | 0.826 | 5.120 | 233 | 118 |
| **Right superior frontal gyrus** | -0.204 | 0.129 | [-0.456 - 0.049] | -0.987 | 0.114 | 0.826 | <0.001 | 234 | 119 |
| **Left rostral anterior cingulate cortex** | -0.190 | 0.170 | [-0.524 - 0.144] | -1.849 | 0.265 | 0.826 | 35.580 | 234 | 119 |
| **Left posterior cingulate cortex** | -0.186 | 0.167 | [-0.515 - 0.142] | -1.167 | 0.265 | 0.826 | 33.633 | 233 | 119 |
| **Right precentral gyrus** | -0.186 | 0.129 | [-0.439 - 0.067] | -1.105 | 0.150 | 0.826 | <0.001 | 230 | 119 |
| **Left caudal anterior cingulate cortex** | -0.182 | 0.144 | [-0.465 - 0.1] | -2.071 | 0.206 | 0.826 | 14.726 | 231 | 119 |
| **Left lateral occipital cortex** | -0.180 | 0.251 | [-0.672 - 0.312] | -1.038 | 0.473 | 0.866 | 68.873 | 234 | 119 |
| **Left banks superior temporal sulcus** | -0.174 | 0.135 | [-0.439 - 0.091] | -1.276 | 0.198 | 0.826 | <0.001 | 219 | 104 |
| **Right fusiform gyrus** | -0.173 | 0.130 | [-0.428 - 0.082] | -0.845 | 0.183 | 0.826 | 0.012 | 234 | 117 |
| **Right inferior parietal cortex** | -0.166 | 0.173 | [-0.504 - 0.173] | -0.938 | 0.338 | 0.826 | 36.975 | 233 | 119 |
| **Left superior frontal gyrus** | -0.162 | 0.130 | [-0.416 - 0.091] | -0.822 | 0.210 | 0.826 | <0.001 | 233 | 118 |
| **Left fusiform gyrus** | -0.157 | 0.165 | [-0.48 - 0.165] | -0.773 | 0.339 | 0.826 | 31.688 | 233 | 119 |
| **Left parahippocampal gyrus** | -0.150 | 0.130 | [-0.405 - 0.104] | -1.873 | 0.247 | 0.826 | 0.004 | 234 | 117 |
| **Right inferior temporal gyrus** | -0.149 | 0.129 | [-0.402 - 0.103] | -0.809 | 0.247 | 0.826 | <0.001 | 231 | 119 |
| **Left hempisphere average thickness** | -0.145 | 0.138 | [-0.416 - 0.126] | -0.534 | 0.293 | 0.826 | 9.454 | 234 | 119 |
| **Right hemisphere average thickness** | -0.140 | 0.129 | [-0.393 - 0.113] | -0.512 | 0.277 | 0.826 | <0.001 | 234 | 119 |
| **Left superior temporal gyrus** | -0.131 | 0.133 | [-0.391 - 0.129] | -0.800 | 0.324 | 0.826 | <0.001 | 223 | 109 |
| **Right superior temporal gyrus** | -0.129 | 0.129 | [-0.383 - 0.124] | -0.774 | 0.318 | 0.826 | 0.002 | 232 | 115 |
| **Left caudal middle frontal gyrus** | -0.118 | 0.129 | [-0.37 - 0.134] | -0.601 | 0.359 | 0.826 | <0.001 | 234 | 119 |
| **Left isthmus cingulate cortex** | -0.118 | 0.129 | [-0.372 - 0.136] | -0.905 | 0.363 | 0.826 | <0.001 | 233 | 118 |
| **Right rostral middle frontal gyrus** | -0.117 | 0.129 | [-0.37 - 0.135] | -0.566 | 0.362 | 0.826 | <0.001 | 232 | 119 |
| **Left precuneus** | -0.098 | 0.266 | [-0.62 - 0.424] | -0.469 | 0.713 | 0.928 | 72.500 | 234 | 119 |
| **Left insula** | -0.098 | 0.177 | [-0.445 - 0.25] | -0.510 | 0.582 | 0.880 | 40.165 | 231 | 119 |
| **Right lateral occipital cortex** | -0.098 | 0.129 | [-0.35 - 0.155] | -0.571 | 0.448 | 0.866 | <0.001 | 234 | 119 |
| **Left rostral middle frontal gyrus** | -0.096 | 0.129 | [-0.349 - 0.156] | -0.466 | 0.455 | 0.866 | 0.004 | 234 | 119 |
| **Right superior parietal cortex** | -0.092 | 0.129 | [-0.345 - 0.161] | -0.459 | 0.476 | 0.866 | <0.001 | 233 | 119 |
| **Left inferior parietal cortex** | -0.091 | 0.129 | [-0.344 - 0.162] | -0.515 | 0.482 | 0.866 | 0.002 | 234 | 119 |
| **Right postcentral gyrus** | -0.081 | 0.129 | [-0.334 - 0.173] | -0.456 | 0.533 | 0.866 | <0.001 | 229 | 119 |
| **Left supramarginal gyrus** | -0.080 | 0.129 | [-0.334 - 0.173] | -0.433 | 0.534 | 0.866 | <0.001 | 231 | 119 |
| **Left pars orbitalis** | -0.079 | 0.243 | [-0.556 - 0.398] | -0.578 | 0.745 | 0.928 | 67.181 | 232 | 119 |
| **Right transverse temporal gyrus** | -0.078 | 0.129 | [-0.331 - 0.174] | -0.631 | 0.542 | 0.866 | <0.001 | 234 | 119 |
| **Right banks superior temporal sulcus** | -0.075 | 0.149 | [-0.368 - 0.218] | -0.532 | 0.617 | 0.882 | 19.136 | 233 | 114 |
| **Right pars orbitalis** | -0.074 | 0.237 | [-0.54 - 0.391] | -0.534 | 0.755 | 0.928 | 65.477 | 234 | 117 |
| **Right supramarginal gyrus** | -0.071 | 0.131 | [-0.328 - 0.187] | -0.363 | 0.591 | 0.880 | 1.567 | 228 | 117 |
| **Left postcentral gyrus** | -0.064 | 0.129 | [-0.318 - 0.189] | -0.356 | 0.618 | 0.882 | <0.001 | 234 | 118 |
| **Right cuneus** | -0.055 | 0.139 | [-0.327 - 0.216] | -0.341 | 0.689 | 0.927 | 9.094 | 232 | 119 |
| **Left cuneus** | -0.050 | 0.222 | [-0.485 - 0.386] | -0.306 | 0.824 | 0.928 | 60.604 | 233 | 119 |
| **Left middle temporal gyrus** | -0.047 | 0.180 | [-0.4 - 0.306] | -0.279 | 0.794 | 0.928 | 37.647 | 224 | 109 |
| **Right precuneus** | -0.040 | 0.195 | [-0.422 - 0.342] | -0.198 | 0.838 | 0.928 | 49.732 | 234 | 119 |
| **Left pars triangularis** | -0.037 | 0.212 | [-0.454 - 0.379] | -0.228 | 0.860 | 0.928 | 57.519 | 233 | 119 |
| **Left pericalcarine cortex** | -0.035 | 0.129 | [-0.287 - 0.218] | -0.244 | 0.787 | 0.928 | <0.001 | 234 | 119 |
| **Right entorhinal cortex** | -0.031 | 0.131 | [-0.288 - 0.226] | -0.373 | 0.811 | 0.928 | <0.001 | 231 | 117 |
| **Right paracentral gyrus** | -0.028 | 0.129 | [-0.281 - 0.226] | -0.157 | 0.830 | 0.928 | <0.001 | 231 | 119 |
| **Left superior parietal cortex** | -0.020 | 0.129 | [-0.272 - 0.232] | -0.098 | 0.877 | 0.928 | <0.001 | 234 | 119 |
| **Right posterior cingulate cortex** | -0.016 | 0.155 | [-0.32 - 0.287] | -0.110 | 0.915 | 0.928 | 24.490 | 233 | 119 |
| **Right insula** | -0.016 | 0.129 | [-0.269 - 0.237] | -0.086 | 0.902 | 0.928 | <0.001 | 231 | 119 |
| **Right middle temporal gyrus** | -0.011 | 0.208 | [-0.418 - 0.396] | -0.063 | 0.957 | 0.957 | 55.348 | 233 | 115 |
| **Left lingual gyrus** | 0.016 | 0.129 | [-0.236 - 0.269] | 0.094 | 0.900 | 0.928 | <0.001 | 234 | 119 |
| **Right frontal pole** | 0.033 | 0.204 | [-0.368 - 0.433] | 0.342 | 0.873 | 0.928 | 54.258 | 234 | 119 |
| **Left inferior temporal gyrus** | 0.035 | 0.130 | [-0.22 - 0.289] | 0.194 | 0.790 | 0.928 | <0.001 | 231 | 118 |
| **Right caudal anterior cingulate cortex** | 0.046 | 0.134 | [-0.218 - 0.309] | 0.510 | 0.734 | 0.928 | 5.832 | 233 | 119 |
| **Right parahippocampal gyrus** | 0.059 | 0.129 | [-0.194 - 0.311] | 0.641 | 0.648 | 0.890 | <0.001 | 234 | 119 |
| **Left pars opercularis** | 0.062 | 0.129 | [-0.191 - 0.315] | 0.295 | 0.632 | 0.884 | <0.001 | 233 | 119 |
| **Right pars triangularis** | 0.076 | 0.129 | [-0.177 - 0.329] | 0.437 | 0.557 | 0.866 | <0.001 | 233 | 119 |
| **Right pericalcarine cortex** | 0.079 | 0.129 | [-0.174 - 0.332] | 0.575 | 0.541 | 0.866 | <0.001 | 234 | 119 |
| **Right caudal middle frontal gyrus** | 0.081 | 0.130 | [-0.174 - 0.335] | 0.425 | 0.535 | 0.866 | 0.002 | 234 | 117 |
| **Left transverse temporal gyrus** | 0.088 | 0.146 | [-0.197 - 0.374] | 0.695 | 0.545 | 0.866 | 16.610 | 234 | 119 |
| **Right lingual gyrus** | 0.112 | 0.129 | [-0.14 - 0.365] | 0.651 | 0.383 | 0.838 | <0.001 | 234 | 119 |
| **Left temporal pole** | 0.117 | 0.129 | [-0.136 - 0.37] | 1.181 | 0.366 | 0.826 | 0.006 | 234 | 119 |
| **Left entorhinal cortex** | 0.121 | 0.129 | [-0.133 - 0.375] | 1.394 | 0.349 | 0.826 | <0.001 | 232 | 118 |
| **Right temporal pole** | 0.163 | 0.209 | [-0.247 - 0.572] | 1.789 | 0.437 | 0.866 | 55.363 | 234 | 119 |

**^a^** Included Samples: MMDP 3T, Melbourne, QTIM, Sydney.

noAD: antidepressant free; MDD: Major Depressive Disorder; CTL: Controls.

**Supplementary Table S43**: Full meta-analytic results for thickness of each structure for MDD patients taking antidepressants MDD patients not taking antidepressants at time of scanning comparison controlling for age, sex and scan center. Adjusted Cohen's d is reported.

|  | **Cohen's d ^a^** | **Std. Err.** | **95% CI** | **% Difference** | **P-value** | **FDR P-value** | **I^2^** | **# AD MDD** | **# noAD MDD** |
| --- | --- | --- | --- | --- | --- | --- | --- | --- | --- |
|  | **(AD MDD vs noAD MDD)** |  |  |  |  |  |  |  |  |
| **Left insula** | -0.292 | 0.168 | [-0.621 - 0.038] | -1.522 | 0.083 | 0.570 | <0.001 | 66 | 88 |
| **Left isthmus cingulate cortex** | -0.259 | 0.177 | [-0.605 - 0.087] | -1.990 | 0.143 | 0.588 | 8.305 | 66 | 87 |
| **Right middle temporal gyrus** | -0.250 | 0.292 | [-0.821 - 0.322] | -1.392 | 0.392 | 0.811 | 63.288 | 63 | 84 |
| **Right pericalcarine cortex** | -0.118 | 0.177 | [-0.465 - 0.229] | -0.863 | 0.504 | 0.818 | 8.027 | 65 | 88 |
| **Right rostral anterior cingulate cortex** | -0.117 | 0.167 | [-0.445 - 0.211] | -1.188 | 0.484 | 0.818 | <0.001 | 66 | 87 |
| **Left transverse temporal gyrus** | -0.111 | 0.167 | [-0.439 - 0.217] | -0.874 | 0.507 | 0.818 | <0.001 | 66 | 88 |
| **Left pars opercularis** | -0.111 | 0.170 | [-0.443 - 0.222] | -0.527 | 0.515 | 0.818 | <0.001 | 64 | 88 |
| **Left middle temporal gyrus** | -0.105 | 0.177 | [-0.452 - 0.242] | -0.625 | 0.552 | 0.818 | <0.001 | 59 | 80 |
| **Left inferior temporal gyrus** | -0.100 | 0.167 | [-0.427 - 0.228] | -0.556 | 0.552 | 0.818 | <0.001 | 66 | 88 |
| **Left temporal pole** | -0.094 | 0.167 | [-0.422 - 0.234] | -0.951 | 0.574 | 0.818 | <0.001 | 66 | 88 |
| **Right cuneus** | -0.076 | 0.167 | [-0.403 - 0.252] | -0.464 | 0.651 | 0.826 | <0.001 | 66 | 88 |
| **Right inferior temporal gyrus** | -0.068 | 0.167 | [-0.396 - 0.26] | -0.369 | 0.685 | 0.826 | <0.001 | 66 | 88 |
| **Left pars triangularis** | -0.034 | 0.173 | [-0.374 - 0.306] | -0.206 | 0.845 | 0.954 | 6.098 | 66 | 88 |
| **Right entorhinal cortex** | -0.023 | 0.173 | [-0.363 - 0.316] | -0.276 | 0.893 | 0.961 | 5.432 | 66 | 86 |
| **Right temporal pole** | -0.022 | 0.167 | [-0.35 - 0.305] | -0.247 | 0.893 | 0.961 | <0.001 | 66 | 88 |
| **Right lingual gyrus** | -0.009 | 0.167 | [-0.336 - 0.319] | -0.050 | 0.959 | 0.987 | <0.001 | 66 | 88 |
| **Left banks superior temporal sulcus** | -0.005 | 0.180 | [-0.358 - 0.347] | -0.038 | 0.977 | 0.989 | <0.001 | 58 | 76 |
| **Right pars triangularis** | -0.003 | 0.212 | [-0.419 - 0.413] | -0.018 | 0.989 | 0.989 | 35.142 | 66 | 88 |
| **Right caudal anterior cingulate cortex** | 0.017 | 0.167 | [-0.311 - 0.344] | 0.187 | 0.920 | 0.961 | <0.001 | 66 | 88 |
| **Right pars orbitalis** | 0.021 | 0.196 | [-0.363 - 0.405] | 0.151 | 0.915 | 0.961 | 23.090 | 65 | 86 |
| **Left cuneus** | 0.021 | 0.167 | [-0.307 - 0.349] | 0.130 | 0.900 | 0.961 | <0.001 | 66 | 88 |
| **Left frontal pole** | 0.046 | 0.167 | [-0.281 - 0.374] | 0.486 | 0.783 | 0.913 | <0.001 | 66 | 88 |
| **Left lateral occipital cortex** | 0.056 | 0.168 | [-0.274 - 0.386] | 0.324 | 0.739 | 0.876 | <0.001 | 65 | 88 |
| **Left supramarginal gyrus** | 0.065 | 0.255 | [-0.434 - 0.564] | 0.351 | 0.798 | 0.916 | 54.207 | 66 | 88 |
| **Left precuneus** | 0.068 | 0.167 | [-0.26 - 0.396] | 0.325 | 0.684 | 0.826 | <0.001 | 66 | 88 |
| **Left pars orbitalis** | 0.069 | 0.167 | [-0.259 - 0.397] | 0.504 | 0.680 | 0.826 | <0.001 | 66 | 88 |
| **Left caudal anterior cingulate cortex** | 0.070 | 0.167 | [-0.258 - 0.398] | 0.796 | 0.675 | 0.826 | <0.001 | 66 | 88 |
| **Left rostral anterior cingulate cortex** | 0.074 | 0.167 | [-0.253 - 0.402] | 0.725 | 0.656 | 0.826 | <0.001 | 66 | 88 |
| **Right insula** | 0.088 | 0.167 | [-0.24 - 0.416] | 0.480 | 0.598 | 0.821 | <0.001 | 66 | 88 |
| **Left fusiform gyrus** | 0.092 | 0.167 | [-0.237 - 0.42] | 0.450 | 0.585 | 0.818 | <0.001 | 66 | 88 |
| **Right precuneus** | 0.094 | 0.167 | [-0.234 - 0.421] | 0.466 | 0.575 | 0.818 | <0.001 | 66 | 88 |
| **Right paracentral gyrus** | 0.100 | 0.167 | [-0.228 - 0.428] | 0.567 | 0.550 | 0.818 | <0.001 | 66 | 88 |
| **Right banks superior temporal sulcus** | 0.104 | 0.174 | [-0.238 - 0.445] | 0.737 | 0.553 | 0.818 | <0.001 | 61 | 83 |
| **Left pericalcarine cortex** | 0.112 | 0.167 | [-0.216 - 0.44] | 0.785 | 0.504 | 0.818 | <0.001 | 66 | 88 |
| **Left precentral gyrus** | 0.120 | 0.169 | [-0.212 - 0.452] | 0.689 | 0.478 | 0.818 | 0.605 | 65 | 88 |
| **Right posterior cingulate cortex** | 0.136 | 0.211 | [-0.277 - 0.549] | 0.911 | 0.519 | 0.818 | 34.264 | 66 | 88 |
| **Right superior temporal gyrus** | 0.139 | 0.171 | [-0.196 - 0.473] | 0.830 | 0.417 | 0.811 | <0.001 | 65 | 84 |
| **Right superior parietal cortex** | 0.139 | 0.167 | [-0.189 - 0.467] | 0.693 | 0.407 | 0.811 | <0.001 | 66 | 88 |
| **Right precentral gyrus** | 0.141 | 0.301 | [-0.45 - 0.732] | 0.837 | 0.641 | 0.826 | 66.902 | 66 | 88 |
| **Right lateral orbitofrontal cortex** | 0.143 | 0.169 | [-0.188 - 0.474] | 0.841 | 0.397 | 0.811 | <0.001 | 65 | 87 |
| **Right lateral occipital cortex** | 0.158 | 0.168 | [-0.17 - 0.487] | 0.926 | 0.345 | 0.797 | <0.001 | 66 | 88 |
| **Left lateral orbitofrontal cortex** | 0.162 | 0.168 | [-0.167 - 0.49] | 0.976 | 0.334 | 0.797 | <0.001 | 66 | 88 |
| **Left superior temporal gyrus** | 0.163 | 0.176 | [-0.181 - 0.508] | 0.998 | 0.353 | 0.797 | <0.001 | 61 | 81 |
| **Left medial orbitofrontal cortex** | 0.170 | 0.174 | [-0.171 - 0.511] | 1.317 | 0.329 | 0.797 | 6.007 | 66 | 87 |
| **Right caudal middle frontal gyrus** | 0.183 | 0.168 | [-0.146 - 0.512] | 0.964 | 0.275 | 0.725 | <0.001 | 66 | 86 |
| **Right frontal pole** | 0.191 | 0.168 | [-0.137 - 0.52] | 1.997 | 0.253 | 0.725 | <0.001 | 66 | 88 |
| **Left superior parietal cortex** | 0.200 | 0.168 | [-0.129 - 0.528] | 0.978 | 0.233 | 0.725 | <0.001 | 66 | 88 |
| **Left inferior parietal cortex** | 0.200 | 0.168 | [-0.129 - 0.528] | 1.134 | 0.233 | 0.725 | <0.001 | 66 | 88 |
| **Right medial orbitofrontal cortex** | 0.209 | 0.168 | [-0.119 - 0.538] | 1.757 | 0.212 | 0.725 | <0.001 | 66 | 88 |
| **Right transverse temporal gyrus** | 0.216 | 0.184 | [-0.144 - 0.577] | 1.741 | 0.240 | 0.725 | 15.236 | 66 | 88 |
| **Left posterior cingulate cortex** | 0.221 | 0.168 | [-0.108 - 0.55] | 1.384 | 0.188 | 0.691 | <0.001 | 66 | 88 |
| **Right isthmus cingulate cortex** | 0.227 | 0.168 | [-0.101 - 0.556] | 1.800 | 0.175 | 0.682 | <0.001 | 66 | 88 |
| **Left postcentral gyrus** | 0.235 | 0.218 | [-0.191 - 0.662] | 1.298 | 0.280 | 0.725 | 37.466 | 66 | 87 |
| **Right supramarginal gyrus** | 0.247 | 0.280 | [-0.301 - 0.795] | 1.267 | 0.378 | 0.811 | 61.328 | 66 | 86 |
| **Left hempisphere average thickness** | 0.248 | 0.168 | [-0.081 - 0.577] | 0.912 | 0.139 | 0.588 | <0.001 | 66 | 88 |
| **Right inferior parietal cortex** | 0.260 | 0.168 | [-0.07 - 0.589] | 1.472 | 0.122 | 0.570 | <0.001 | 66 | 88 |
| **Left entorhinal cortex** | 0.268 | 0.170 | [-0.065 - 0.601] | 3.079 | 0.115 | 0.570 | <0.001 | 64 | 87 |
| **Right hemisphere average thickness** | 0.271 | 0.168 | [-0.058 - 0.6] | 0.990 | 0.107 | 0.570 | <0.001 | 66 | 88 |
| **Left lingual gyrus** | 0.273 | 0.168 | [-0.057 - 0.602] | 1.577 | 0.105 | 0.570 | <0.001 | 66 | 88 |
| **Right fusiform gyrus** | 0.282 | 0.168 | [-0.048 - 0.612] | 1.377 | 0.094 | 0.570 | <0.001 | 66 | 86 |
| **Left caudal middle frontal gyrus** | 0.292 | 0.168 | [-0.038 - 0.621] | 1.487 | 0.083 | 0.570 | <0.001 | 66 | 88 |
| **Left parahippocampal gyrus** | 0.305 | 0.278 | [-0.239 - 0.85] | 3.800 | 0.272 | 0.725 | 60.780 | 66 | 86 |
| **Right parahippocampal gyrus** | 0.313 | 0.168 | [-0.017 - 0.643] | 3.415 | 0.063 | 0.570 | <0.001 | 66 | 88 |
| **Left rostral middle frontal gyrus** | 0.342 | 0.168 | [0.012 - 0.672] | 1.656 | 0.042 | 0.493 | <0.001 | 66 | 88 |
| **Right rostral middle frontal gyrus** | 0.348 | 0.213 | [-0.07 - 0.766] | 1.678 | 0.103 | 0.570 | 34.886 | 66 | 88 |
| **Left superior frontal gyrus** | 0.355 | 0.169 | [0.025 - 0.686] | 1.797 | 0.035 | 0.491 | <0.001 | 66 | 87 |
| **Right pars opercularis** | 0.368 | 0.169 | [0.037 - 0.698] | 1.843 | 0.029 | 0.491 | <0.001 | 66 | 88 |
| **Right superior frontal gyrus** | 0.378 | 0.169 | [0.047 - 0.708] | 1.831 | 0.025 | 0.491 | <0.001 | 66 | 88 |
| **Right postcentral gyrus** | 0.386 | 0.170 | [0.053 - 0.719] | 2.184 | 0.023 | 0.491 | <0.001 | 65 | 88 |
| **Left paracentral gyrus** | 0.433 | 0.169 | [0.101 - 0.765] | 2.466 | 0.010 | 0.491 | <0.001 | 66 | 87 |

**^a^** Included Samples: Melbourne, Sydney.

AD: antidepressant using; noAD: antidepressant free; MDD: Major Depressive Disorder.

**Supplementary Table S44**: Full meta-analytic results for thickness of each structure associated with severity of symptoms at study inclusion measured by the HDRS-17 controlling for age, sex and scan center. Adjusted Cohen's d is reported.

|  | **Pearson's r ^a^** | **Std. Err.** | **95% CI** | **% Difference** | **P-value** | **FDR P-value** | **I^2^** | **# Patients** |
| --- | --- | --- | --- | --- | --- | --- | --- | --- |
|  | **(HDRS-17)** |  |  |  |  |  |  |  |
| **Left parahippocampal gyrus** | -0.282 | 0.139 | [0.042 - -0.554] | -7.320 | 0.042 | 0.543 | 62.161 | 134 |
| **Left posterior cingulate cortex** | -0.276 | 0.165 | [0.094 - -0.599] | -3.593 | 0.094 | 0.550 | 80.961 | 134 |
| **Left fusiform gyrus** | -0.273 | 0.116 | [0.019 - -0.501] | -2.789 | 0.019 | 0.543 | 42.996 | 134 |
| **Left postcentral gyrus** | -0.273 | 0.147 | [0.063 - -0.561] | -3.131 | 0.063 | 0.543 | 68.772 | 134 |
| **Left caudal middle frontal gyrus** | -0.272 | 0.182 | [0.136 - -0.629] | -2.882 | 0.136 | 0.678 | 85.625 | 134 |
| **Right insula** | -0.261 | 0.139 | [0.061 - -0.533] | -2.940 | 0.061 | 0.543 | 63.936 | 134 |
| **Left inferior temporal gyrus** | -0.246 | 0.183 | [0.179 - -0.604] | -2.831 | 0.179 | 0.782 | 78.818 | 134 |
| **Left transverse temporal gyrus** | -0.240 | 0.136 | [0.078 - -0.507] | -3.900 | 0.078 | 0.543 | 58.447 | 134 |
| **Right lateral occipital cortex** | -0.215 | 0.128 | [0.094 - -0.466] | -2.574 | 0.094 | 0.550 | 49.934 | 134 |
| **Left supramarginal gyrus** | -0.203 | 0.131 | [0.121 - -0.46] | -2.234 | 0.121 | 0.653 | 51.123 | 134 |
| **Left pars triangularis** | -0.195 | 0.157 | [0.214 - -0.504] | -2.425 | 0.214 | 0.818 | 69.279 | 134 |
| **Right transverse temporal gyrus** | -0.188 | 0.084 | [0.025 - -0.352] | -3.084 | 0.025 | 0.543 | <0.001 | 134 |
| **Right banks superior temporal sulcus** | -0.183 | 0.156 | [0.24 - -0.488] | -2.648 | 0.240 | 0.839 | 64.199 | 125 |
| **Right superior temporal gyrus** | -0.179 | 0.125 | [0.15 - -0.423] | -2.182 | 0.150 | 0.701 | 42.030 | 129 |
| **Left lateral occipital cortex** | -0.165 | 0.085 | [0.052 - -0.332] | -1.931 | 0.052 | 0.543 | 1.775 | 134 |
| **Left hempisphere average thickness** | -0.151 | 0.170 | [0.376 - -0.485] | -1.122 | 0.376 | 0.878 | 72.733 | 134 |
| **Left isthmus cingulate cortex** | -0.150 | 0.083 | [0.071 - -0.314] | -2.338 | 0.071 | 0.543 | <0.001 | 134 |
| **Left insula** | -0.147 | 0.171 | [0.388 - -0.482] | -1.557 | 0.388 | 0.878 | 75.662 | 134 |
| **Left middle temporal gyrus** | -0.140 | 0.130 | [0.279 - -0.394] | -1.683 | 0.279 | 0.847 | 46.096 | 122 |
| **Right precuneus** | -0.131 | 0.115 | [0.252 - -0.356] | -1.317 | 0.252 | 0.840 | 32.651 | 134 |
| **Right cuneus** | -0.128 | 0.125 | [0.305 - -0.373] | -1.587 | 0.305 | 0.847 | 44.156 | 134 |
| **Right pars opercularis** | -0.127 | 0.162 | [0.431 - -0.444] | -1.288 | 0.431 | 0.878 | 67.341 | 134 |
| **Right posterior cingulate cortex** | -0.126 | 0.122 | [0.304 - -0.366] | -1.697 | 0.304 | 0.847 | 41.140 | 134 |
| **Right supramarginal gyrus** | -0.117 | 0.092 | [0.204 - -0.297] | -1.206 | 0.204 | 0.818 | 7.957 | 133 |
| **Left superior temporal gyrus** | -0.114 | 0.136 | [0.402 - -0.38] | -1.399 | 0.402 | 0.878 | 50.494 | 124 |
| **Right postcentral gyrus** | -0.113 | 0.220 | [0.608 - -0.545] | -1.288 | 0.608 | 0.893 | 89.727 | 134 |
| **Right hemisphere average thickness** | -0.102 | 0.200 | [0.61 - -0.494] | -0.749 | 0.610 | 0.893 | 81.576 | 134 |
| **Left caudal anterior cingulate cortex** | -0.097 | 0.139 | [0.489 - -0.37] | -2.203 | 0.489 | 0.878 | 52.573 | 134 |
| **Right inferior temporal gyrus** | -0.089 | 0.117 | [0.445 - -0.319] | -0.974 | 0.445 | 0.878 | 31.114 | 134 |
| **Left precentral gyrus** | -0.088 | 0.219 | [0.687 - -0.518] | -1.017 | 0.687 | 0.895 | 87.483 | 134 |
| **Left precuneus** | -0.088 | 0.085 | [0.299 - -0.254] | -0.843 | 0.299 | 0.847 | <0.001 | 134 |
| **Right fusiform gyrus** | -0.085 | 0.085 | [0.315 - -0.251] | -0.834 | 0.315 | 0.847 | 0.741 | 134 |
| **Left entorhinal cortex** | -0.078 | 0.119 | [0.514 - -0.311] | -1.796 | 0.514 | 0.878 | 33.658 | 133 |
| **Right pericalcarine cortex** | -0.077 | 0.109 | [0.48 - -0.289] | -1.121 | 0.480 | 0.878 | 22.983 | 134 |
| **Right parahippocampal gyrus** | -0.075 | 0.183 | [0.68 - -0.433] | -1.646 | 0.680 | 0.895 | 74.902 | 133 |
| **Right caudal anterior cingulate cortex** | -0.072 | 0.205 | [0.725 - -0.474] | -1.609 | 0.725 | 0.895 | 81.806 | 134 |
| **Left paracentral gyrus** | -0.061 | 0.086 | [0.479 - -0.229] | -0.693 | 0.479 | 0.878 | <0.001 | 134 |
| **Left banks superior temporal sulcus** | -0.059 | 0.091 | [0.514 - -0.238] | -0.871 | 0.514 | 0.878 | <0.001 | 119 |
| **Right isthmus cingulate cortex** | -0.058 | 0.087 | [0.506 - -0.227] | -0.914 | 0.506 | 0.878 | <0.001 | 134 |
| **Right inferior parietal cortex** | -0.057 | 0.170 | [0.738 - -0.391] | -0.645 | 0.738 | 0.895 | 71.132 | 134 |
| **Right precentral gyrus** | -0.056 | 0.214 | [0.792 - -0.476] | -0.672 | 0.792 | 0.895 | 85.225 | 134 |
| **Right rostral anterior cingulate cortex** | -0.049 | 0.186 | [0.79 - -0.413] | -1.004 | 0.790 | 0.895 | 75.611 | 134 |
| **Left pars opercularis** | -0.049 | 0.095 | [0.607 - -0.236] | -0.467 | 0.607 | 0.893 | 10.108 | 134 |
| **Left cuneus** | -0.039 | 0.127 | [0.761 - -0.288] | -0.478 | 0.761 | 0.895 | 40.612 | 134 |
| **Left superior parietal cortex** | -0.037 | 0.136 | [0.786 - -0.303] | -0.362 | 0.786 | 0.895 | 50.090 | 134 |
| **Left inferior parietal cortex** | -0.030 | 0.164 | [0.857 - -0.351] | -0.336 | 0.857 | 0.909 | 66.820 | 134 |
| **Left temporal pole** | -0.029 | 0.095 | [0.757 - -0.216] | -0.596 | 0.757 | 0.895 | 8.668 | 134 |
| **Right paracentral gyrus** | -0.018 | 0.194 | [0.927 - -0.398] | -0.203 | 0.927 | 0.940 | 79.144 | 134 |
| **Right pars triangularis** | -0.017 | 0.087 | [0.846 - -0.187] | -0.194 | 0.846 | 0.909 | <0.001 | 134 |
| **Right middle temporal gyrus** | -0.012 | 0.092 | [0.898 - -0.192] | -0.131 | 0.898 | 0.925 | 3.375 | 127 |
| **Right caudal middle frontal gyrus** | 3.42E-04 | 0.207 | [0.999 - -0.406] | 0.004 | 0.999 | 0.999 | 82.209 | 134 |
| **Left medial orbitofrontal cortex** | 0.017 | 0.087 | [0.843 - -0.153] | 0.268 | 0.843 | 0.909 | <0.001 | 134 |
| **Right superior parietal cortex** | 0.030 | 0.122 | [0.806 - -0.209] | 0.299 | 0.806 | 0.896 | 37.055 | 134 |
| **Left lingual gyrus** | 0.033 | 0.087 | [0.706 - -0.138] | 0.380 | 0.706 | 0.895 | <0.001 | 134 |
| **Left frontal pole** | 0.035 | 0.228 | [0.877 - -0.412] | 0.746 | 0.877 | 0.916 | 88.200 | 134 |
| **Left pars orbitalis** | 0.040 | 0.087 | [0.644 - -0.13] | 0.586 | 0.644 | 0.893 | <0.001 | 134 |
| **Left rostral middle frontal gyrus** | 0.044 | 0.123 | [0.722 - -0.197] | 0.423 | 0.722 | 0.895 | 35.809 | 134 |
| **Right temporal pole** | 0.056 | 0.093 | [0.548 - -0.127] | 1.233 | 0.548 | 0.893 | 7.379 | 134 |
| **Left pericalcarine cortex** | 0.063 | 0.086 | [0.464 - -0.105] | 0.884 | 0.464 | 0.878 | <0.001 | 134 |
| **Right pars orbitalis** | 0.064 | 0.086 | [0.457 - -0.105] | 0.925 | 0.457 | 0.878 | <0.001 | 134 |
| **Left rostral anterior cingulate cortex** | 0.067 | 0.087 | [0.442 - -0.104] | 1.308 | 0.442 | 0.878 | <0.001 | 134 |
| **Right lingual gyrus** | 0.078 | 0.167 | [0.642 - -0.25] | 0.904 | 0.642 | 0.893 | 70.391 | 134 |
| **Right rostral middle frontal gyrus** | 0.087 | 0.171 | [0.61 - -0.249] | 0.847 | 0.610 | 0.893 | 71.886 | 134 |
| **Left superior frontal gyrus** | 0.092 | 0.197 | [0.639 - -0.293] | 0.938 | 0.639 | 0.893 | 79.597 | 134 |
| **Right lateral orbitofrontal cortex** | 0.093 | 0.198 | [0.638 - -0.295] | 1.097 | 0.638 | 0.893 | 81.143 | 134 |
| **Right superior frontal gyrus** | 0.099 | 0.220 | [0.651 - -0.331] | 0.969 | 0.651 | 0.893 | 85.055 | 134 |
| **Right entorhinal cortex** | 0.139 | 0.114 | [0.222 - -0.084] | 3.349 | 0.222 | 0.818 | 29.303 | 133 |
| **Right medial orbitofrontal cortex** | 0.141 | 0.201 | [0.481 - -0.252] | 2.397 | 0.481 | 0.878 | 87.310 | 134 |
| **Left lateral orbitofrontal cortex** | 0.155 | 0.085 | [0.07 - -0.012] | 1.890 | 0.070 | 0.543 | <0.001 | 134 |
| **Right frontal pole** | 0.177 | 0.091 | [0.05 - 0] | 3.763 | 0.050 | 0.543 | 9.214 | 134 |

**^a^** Included Samples: Houston, MMDP 3T, Muenster Cohort, DepOx, Sydney.

HDRS-17: Hamilton Depression Rating Scale with 17 items.

**Adolescent meta-analyses results for cortical surface area**

**Supplementary Table S45**: Full meta-analytic results for surface area of each structure for the Diagnosis by Sex interaction controlling for age, sex and scan center. Adjusted Cohen's d is reported.

|  | **Cohen's d ^a^** | **Std. Err.** | **95% CI** | **% Difference** | **P-value** | **FDR P-value** | **I^2^** | **# Controls** | **# Patients** |
| --- | --- | --- | --- | --- | --- | --- | --- | --- | --- |
|  | **(Dx by Sex)** |  |  |  |  |  |  |  |  |
| **Left transverse temporal gyrus** | -0.185 | 0.194 | [-0.566 - 0.195] | -3.069 | 0.339 | 0.625 | 65.451 | 294 | 213 |
| **Right frontal pole** | -0.168 | 0.235 | [-0.629 - 0.293] | -2.584 | 0.475 | 0.711 | 76.117 | 294 | 213 |
| **Right rostral anterior cingulate cortex** | -0.075 | 0.107 | [-0.285 - 0.136] | -1.696 | 0.488 | 0.711 | <0.001 | 292 | 212 |
| **Right pars orbitalis** | -0.056 | 0.107 | [-0.266 - 0.154] | -0.844 | 0.602 | 0.766 | <0.001 | 294 | 211 |
| **Left superior temporal gyrus** | -0.041 | 0.167 | [-0.368 - 0.286] | -0.511 | 0.806 | 0.881 | 51.253 | 283 | 198 |
| **Left pars orbitalis** | -0.032 | 0.107 | [-0.242 - 0.178] | -0.483 | 0.766 | 0.864 | <0.001 | 292 | 213 |
| **Left isthmus cingulate cortex** | -0.011 | 0.221 | [-0.445 - 0.422] | -0.189 | 0.960 | 0.988 | 73.203 | 293 | 212 |
| **Right lingual gyrus** | 0.003 | 0.143 | [-0.276 - 0.283] | 0.043 | 0.983 | 0.988 | 37.815 | 294 | 213 |
| **Left lingual gyrus** | 0.004 | 0.259 | [-0.504 - 0.511] | 0.054 | 0.988 | 0.988 | 80.479 | 294 | 213 |
| **Left posterior cingulate cortex** | 0.021 | 0.107 | [-0.189 - 0.231] | 0.305 | 0.847 | 0.898 | <0.001 | 293 | 213 |
| **Right isthmus cingulate cortex** | 0.022 | 0.269 | [-0.506 - 0.549] | 0.350 | 0.936 | 0.978 | 81.867 | 293 | 213 |
| **Left fusiform gyrus** | 0.027 | 0.107 | [-0.183 - 0.237] | 0.395 | 0.799 | 0.881 | <0.001 | 293 | 213 |
| **Right paracentral gyrus** | 0.030 | 0.155 | [-0.274 - 0.335] | 0.426 | 0.845 | 0.898 | 46.223 | 291 | 213 |
| **Left temporal pole** | 0.033 | 0.107 | [-0.176 - 0.242] | 0.526 | 0.758 | 0.864 | <0.001 | 294 | 213 |
| **Right pericalcarine cortex** | 0.046 | 0.137 | [-0.223 - 0.315] | 0.771 | 0.737 | 0.864 | 33.129 | 293 | 212 |
| **Left paracentral gyrus** | 0.060 | 0.107 | [-0.15 - 0.27] | 0.836 | 0.577 | 0.749 | <0.001 | 294 | 212 |
| **Left frontal pole** | 0.060 | 0.107 | [-0.15 - 0.27] | 0.936 | 0.574 | 0.749 | <0.001 | 294 | 213 |
| **Left superior frontal gyrus** | 0.060 | 0.175 | [-0.283 - 0.404] | 0.741 | 0.730 | 0.864 | 57.529 | 293 | 212 |
| **Right posterior cingulate cortex** | 0.062 | 0.191 | [-0.313 - 0.437] | 0.949 | 0.747 | 0.864 | 64.487 | 293 | 213 |
| **Left rostral anterior cingulate cortex** | 0.063 | 0.107 | [-0.147 - 0.272] | 1.380 | 0.559 | 0.749 | <0.001 | 293 | 213 |
| **Left precuneus** | 0.069 | 0.177 | [-0.277 - 0.415] | 0.875 | 0.697 | 0.856 | 58.294 | 294 | 213 |
| **Left lateral orbitofrontal cortex** | 0.070 | 0.107 | [-0.14 - 0.279] | 1.037 | 0.515 | 0.736 | <0.001 | 294 | 213 |
| **Left parahippocampal gyrus** | 0.076 | 0.122 | [-0.164 - 0.315] | 1.614 | 0.535 | 0.749 | 18.046 | 294 | 211 |
| **Right precuneus** | 0.079 | 0.166 | [-0.246 - 0.405] | 1.030 | 0.633 | 0.791 | 53.112 | 294 | 213 |
| **Right superior parietal cortex** | 0.083 | 0.107 | [-0.127 - 0.293] | 0.969 | 0.439 | 0.699 | 0.004 | 293 | 213 |
| **Left pars triangularis** | 0.084 | 0.107 | [-0.126 - 0.294] | 1.297 | 0.433 | 0.699 | 0.003 | 293 | 213 |
| **Right transverse temporal gyrus** | 0.085 | 0.117 | [-0.145 - 0.314] | 1.404 | 0.470 | 0.711 | 12.836 | 294 | 213 |
| **Right superior temporal gyrus** | 0.085 | 0.143 | [-0.196 - 0.366] | 1.000 | 0.553 | 0.749 | 38.297 | 292 | 208 |
| **Right caudal middle frontal gyrus** | 0.089 | 0.107 | [-0.121 - 0.3] | 1.611 | 0.405 | 0.675 | <0.001 | 294 | 211 |
| **Right lateral occipital cortex** | 0.095 | 0.107 | [-0.114 - 0.305] | 1.259 | 0.373 | 0.652 | <0.001 | 294 | 213 |
| **Left pars opercularis** | 0.099 | 0.107 | [-0.112 - 0.309] | 1.594 | 0.357 | 0.640 | <0.001 | 293 | 211 |
| **Left caudal anterior cingulate cortex** | 0.102 | 0.107 | [-0.108 - 0.313] | 1.932 | 0.339 | 0.625 | <0.001 | 291 | 213 |
| **Right pars opercularis** | 0.105 | 0.107 | [-0.105 - 0.315] | 1.688 | 0.326 | 0.625 | <0.001 | 292 | 213 |
| **Right rostral middle frontal gyrus** | 0.111 | 0.107 | [-0.099 - 0.321] | 1.574 | 0.299 | 0.625 | <0.001 | 292 | 213 |
| **Right caudal anterior cingulate cortex** | 0.112 | 0.107 | [-0.097 - 0.322] | 2.180 | 0.293 | 0.625 | <0.001 | 293 | 213 |
| **Right insula** | 0.118 | 0.107 | [-0.093 - 0.328] | 1.607 | 0.274 | 0.618 | 0.001 | 292 | 213 |
| **Right lateral orbitofrontal cortex** | 0.120 | 0.108 | [-0.091 - 0.331] | 1.689 | 0.264 | 0.617 | 0.159 | 294 | 211 |
| **Left medial orbitofrontal cortex** | 0.120 | 0.171 | [-0.215 - 0.456] | 1.885 | 0.483 | 0.711 | 55.118 | 283 | 210 |
| **Left middle temporal gyrus** | 0.121 | 0.124 | [-0.122 - 0.363] | 1.793 | 0.331 | 0.625 | 16.729 | 283 | 196 |
| **Left banks superior temporal sulcus** | 0.122 | 0.123 | [-0.119 - 0.363] | 2.106 | 0.322 | 0.625 | 14.943 | 279 | 190 |
| **Left pericalcarine cortex** | 0.126 | 0.107 | [-0.085 - 0.336] | 2.172 | 0.241 | 0.603 | <0.001 | 294 | 213 |
| **Left caudal middle frontal gyrus** | 0.140 | 0.107 | [-0.07 - 0.35] | 2.302 | 0.192 | 0.517 | 0.004 | 294 | 213 |
| **Left entorhinal cortex** | 0.150 | 0.108 | [-0.061 - 0.361] | 3.343 | 0.163 | 0.476 | 0.001 | 292 | 209 |
| **Left supramarginal gyrus** | 0.151 | 0.107 | [-0.059 - 0.361] | 2.202 | 0.159 | 0.476 | <0.001 | 292 | 213 |
| **Left lateral occipital cortex** | 0.151 | 0.134 | [-0.111 - 0.413] | 1.950 | 0.258 | 0.617 | 29.962 | 294 | 212 |
| **Left superior parietal cortex** | 0.162 | 0.107 | [-0.048 - 0.372] | 1.951 | 0.131 | 0.444 | 0.008 | 294 | 213 |
| **Right cuneus** | 0.166 | 0.107 | [-0.045 - 0.377] | 2.375 | 0.123 | 0.444 | 0.003 | 292 | 212 |
| **Right fusiform gyrus** | 0.173 | 0.107 | [-0.037 - 0.384] | 2.583 | 0.107 | 0.444 | <0.001 | 294 | 211 |
| **Right pars triangularis** | 0.176 | 0.107 | [-0.034 - 0.386] | 2.858 | 0.100 | 0.444 | 0.001 | 293 | 213 |
| **Right temporal pole** | 0.180 | 0.136 | [-0.086 - 0.447] | 2.931 | 0.185 | 0.517 | 32.156 | 294 | 213 |
| **Right parahippocampal gyrus** | 0.188 | 0.123 | [-0.054 - 0.429] | 3.068 | 0.128 | 0.444 | 19.133 | 294 | 212 |
| **Left insula** | 0.191 | 0.109 | [-0.023 - 0.406] | 2.157 | 0.081 | 0.403 | 2.598 | 291 | 213 |
| **Left inferior temporal gyrus** | 0.193 | 0.108 | [-0.019 - 0.404] | 3.180 | 0.074 | 0.398 | <0.001 | 290 | 212 |
| **Left hemisphere total surface area** | 0.214 | 0.135 | [-0.049 - 0.478] | 2.230 | 0.111 | 0.444 | 30.649 | 294 | 213 |
| **Right postcentral gyrus** | 0.217 | 0.255 | [-0.282 - 0.716] | 2.597 | 0.394 | 0.673 | 79.683 | 289 | 212 |
| **Right superior frontal gyrus** | 0.233 | 0.107 | [0.022 - 0.443] | 2.984 | 0.030 | 0.236 | 0.004 | 294 | 213 |
| **Right hemisphere total surface area** | 0.234 | 0.120 | [-0.001 - 0.469] | 2.440 | 0.051 | 0.355 | 15.556 | 294 | 213 |
| **Left cuneus** | 0.235 | 0.186 | [-0.13 - 0.6] | 3.519 | 0.207 | 0.538 | 62.504 | 294 | 213 |
| **Right inferior temporal gyrus** | 0.245 | 0.107 | [0.035 - 0.455] | 4.142 | 0.022 | 0.226 | 0.010 | 291 | 213 |
| **Right inferior parietal cortex** | 0.257 | 0.107 | [0.047 - 0.468] | 3.561 | 0.017 | 0.226 | <0.001 | 293 | 213 |
| **Right medial orbitofrontal cortex** | 0.267 | 0.108 | [0.055 - 0.48] | 4.116 | 0.014 | 0.226 | <0.001 | 282 | 211 |
| **Left postcentral gyrus** | 0.275 | 0.148 | [-0.016 - 0.566] | 3.204 | 0.064 | 0.398 | 41.770 | 293 | 212 |
| **Left rostral middle frontal gyrus** | 0.284 | 0.107 | [0.074 - 0.494] | 3.999 | 0.008 | 0.188 | <0.001 | 294 | 213 |
| **Right banks superior temporal sulcus** | 0.285 | 0.199 | [-0.106 - 0.676] | 4.472 | 0.153 | 0.476 | 67.045 | 293 | 203 |
| **Right precentral gyrus** | 0.288 | 0.132 | [0.029 - 0.546] | 3.270 | 0.029 | 0.236 | 27.849 | 290 | 213 |
| **Right middle temporal gyrus** | 0.296 | 0.107 | [0.085 - 0.506] | 4.207 | 0.006 | 0.188 | <0.001 | 293 | 206 |
| **Left inferior parietal cortex** | 0.308 | 0.107 | [0.097 - 0.518] | 4.414 | 0.004 | 0.188 | 0.009 | 294 | 213 |
| **Left precentral gyrus** | 0.313 | 0.172 | [-0.025 - 0.65] | 3.448 | 0.069 | 0.398 | 55.965 | 291 | 212 |
| **Right entorhinal cortex** | 0.332 | 0.221 | [-0.101 - 0.766] | 7.321 | 0.133 | 0.444 | 72.179 | 291 | 209 |
| **Right supramarginal gyrus** | 0.345 | 0.151 | [0.048 - 0.641] | 4.932 | 0.023 | 0.226 | 43.283 | 288 | 211 |

**^a^** Included Samples: MMDP 3T, Melbourne, Muenster Cohort, QTIM, Sydney.

Dx: Diagnosis.

**Supplementary Table S46**: Full meta-analytic results for surface area of each structure for the Diagnosis by Age interaction controlling for age, sex and scan center. Adjusted Cohen's d is reported.

|  | **Cohen's d ^a^** | **Std. Err.** | **95% CI** | **% Difference** | **P-value** | **FDR P-value** | **I^2^** | **# Controls** | **# Patients** |
| --- | --- | --- | --- | --- | --- | --- | --- | --- | --- |
| **(Dx by Age)** |
| **Left frontal pole** | -0.286 | 0.229 | [-0.734 - 0.163] | -4.444 | 0.212 | 0.982 | 74.737 | 294 | 213 |
| **Right postcentral gyrus** | -0.285 | 0.115 | [-0.509 - -0.06] | -3.404 | 0.013 | 0.458 | 8.535 | 289 | 212 |
| **Right lateral occipital cortex** | -0.283 | 0.108 | [-0.494 - -0.071] | -3.735 | 0.009 | 0.458 | 0.378 | 294 | 213 |
| **Left inferior parietal cortex** | -0.256 | 0.181 | [-0.61 - 0.099] | -3.665 | 0.158 | 0.982 | 59.937 | 294 | 213 |
| **Right fusiform gyrus** | -0.250 | 0.148 | [-0.541 - 0.041] | -3.731 | 0.092 | 0.982 | 40.942 | 294 | 211 |
| **Left paracentral gyrus** | -0.247 | 0.174 | [-0.588 - 0.093] | -3.469 | 0.155 | 0.982 | 56.922 | 294 | 212 |
| **Left cuneus** | -0.220 | 0.107 | [-0.43 - -0.01] | -3.295 | 0.040 | 0.938 | <0.001 | 294 | 213 |
| **Left lingual gyrus** | -0.187 | 0.177 | [-0.534 - 0.161] | -2.625 | 0.293 | 0.982 | 58.566 | 294 | 213 |
| **Left precentral gyrus** | -0.186 | 0.212 | [-0.602 - 0.23] | -2.054 | 0.380 | 0.982 | 70.725 | 291 | 212 |
| **Right entorhinal cortex** | -0.171 | 0.109 | [-0.385 - 0.042] | -3.776 | 0.116 | 0.982 | <0.001 | 291 | 209 |
| **Left middle temporal gyrus** | -0.171 | 0.205 | [-0.573 - 0.23] | -2.548 | 0.403 | 0.982 | 67.198 | 283 | 196 |
| **Left pars orbitalis** | -0.170 | 0.108 | [-0.381 - 0.04] | -2.572 | 0.113 | 0.982 | 0.014 | 292 | 213 |
| **Right parahippocampal gyrus** | -0.168 | 0.116 | [-0.396 - 0.06] | -2.747 | 0.149 | 0.982 | 11.157 | 294 | 212 |
| **Left posterior cingulate cortex** | -0.141 | 0.112 | [-0.361 - 0.078] | -2.083 | 0.207 | 0.982 | 6.501 | 293 | 213 |
| **Right middle temporal gyrus** | -0.141 | 0.147 | [-0.43 - 0.148] | -2.009 | 0.338 | 0.982 | 41.224 | 293 | 206 |
| **Right superior temporal gyrus** | -0.135 | 0.143 | [-0.416 - 0.146] | -1.589 | 0.346 | 0.982 | 38.097 | 292 | 208 |
| **Left banks superior temporal sulcus** | -0.131 | 0.158 | [-0.441 - 0.179] | -2.263 | 0.408 | 0.982 | 45.298 | 279 | 190 |
| **Right hemisphere total surface area** | -0.130 | 0.135 | [-0.395 - 0.134] | -1.360 | 0.334 | 0.982 | 31.202 | 294 | 213 |
| **Right isthmus cingulate cortex** | -0.129 | 0.107 | [-0.339 - 0.081] | -2.084 | 0.228 | 0.982 | <0.001 | 293 | 213 |
[truncated: 185,014 more chars]
