# Supplementary material for: Cortical abnormalities in adults and adolescents with major depression based on brain scans from 20 cohorts worldwide in the ENIGMA Major Depressive Disorder Working Group
Source: Mol Psychiatry. 2016 May 3;22(6):900–9. doi: 10.1038/mp.2016.60 (PMC5444023; doi:10.1038/mp.2016.60)
Supplement: Supplementary Figures [file mp201660x3.docx]

Supplementary Figures

**Content:**

**Supplemental Figure S1**: Overview of research institutes participating in the ENIGMA-Major Depressive Disorder Working Group, displayed on a world map.

- **Figures displaying effect sizes on the brain surface**

**Adults:**

**Supplemental Figure S2:** Meta-analysis effect sizes for regions that showed a significant (P<0.05) difference in cortical thickness between adult recurrent MDD patients and healthy controls. Negative effect sizes *d* indicate cortical thinning in MDD compared to controls.

**Supplemental Figure S3:** Meta-analysis effect sizes for regions that showed a significant (P<0.05) difference in cortical thickness between adult first episode MDD patients and healthy controls. Negative effect sizes *d* indicate cortical thinning in MDD compared to controls.

**Supplemental Figure S4:** Meta-analysis effect sizes for regions that showed a significant (P<0.05) difference in cortical thickness between adult late age of onset MDD patients and healthy controls. Negative effect sizes *d* indicate cortical thinning in MDD compared to controls.

**Supplemental Figure S5:** Meta-analysis effect sizes for regions that showed a significant (P<0.05) difference in cortical thickness between adult MDD patients taking antidepressants at time of scanning and healthy controls. Negative effect sizes *d* indicate cortical thinning in MDD compared to controls.

**Supplemental Figure S6:** Meta-analysis effect sizes for regions that showed a significant (P<0.05) difference in cortical thickness between adult MDD patients not taking antidepressants at time of scanning and healthy controls. Negative effect sizes *d* indicate cortical thinning in MDD compared to controls.

**Supplemental Figure S7:** Meta-analysis effect sizes for regions that showed a significant (P<0.05) association of cortical thickness and symptom severity at study inclusion measured by the HDRS-17. Negative effect sizes *d* indicate cortical thinning in MDD compared to controls.

**Adolescents:**

**Supplemental Figure S8:** Meta-analysis effect sizes for regions that showed a significant (P<0.05) difference in cortical surface area between adolescent recurrent MDD patients and healthy controls. Negative effect sizes *d* indicate reduced cortical surface area in MDD compared to controls.

**Supplemental Figure S9:** Meta-analysis effect sizes for regions that showed a significant (P<0.05) difference in cortical surface area between adolescent antidepressant not taking MDD patients at time of scanning and healthy controls. Negative effect sizes *d* indicate reduced cortical surface in MDD compared to controls.

**Supplemental Figure S10:** Meta-analysis effect sizes for regions that showed a significant (P<0.05) difference in cortical thickness between adolescent MDD patients taking antidepressants and adolescent MDD patients not taking antidepressants at time of scanning. Postitive effect sizes *d* indicate larger cortical surface in adolescent MDD patients taking antidepressants compared to adolescent MDD patients not taking antidepressants.

- **Forest plots for significant meta-analytic effect sizes**

**Adults:**

**Supplemental Figure S11:** Forest plots of meta-analytic effect sizes of regional cortical thickness with *p*<0.05: adult MDD patients versus controls.

**Supplemental Figure S12:** Forest plots of meta-analytic effect sizes of regional cortical thickness with *p*<0.05: adult recurrent MDD patients versus controls.

**Supplemental Figure S13:** Forest plots of meta-analytic effect sizes of regional cortical thickness with *p*<0.05: adult first episode MDD patients versus controls.

**Supplemental Figure S14:** Forest plots of meta-analytic effect sizes of regional cortical thickness with *p*<0.05: adult late age of onset MDD patients versus controls.

**Supplemental Figure S15:** Forest plots of meta-analytic effect sizes of regional cortical thickness with *p*<0.05: adult MDD patients taking antidepressants at time of scanning versus controls.

**Supplemental Figure S16:** Forest plots of meta-analytic effect sizes of regional cortical thickness with *p*<0.05: adult MDD patients not taking antidepressants at time of scanning versus controls.

**Supplemental Figure S17:** Forest plots of meta-analytic effect sizes of regional cortical thickness with *p*<0.05: association with symptom severity at study inclusion measured by the HDRS-17.

**Adolescents:**

**Supplemental Figure S18:** Forest plots of meta-analytic effect sizes of regional cortical surface area with *p*<0.05: adolescent MDD patients versus controls.

**Supplemental Figure S19:** Forest plots of meta-analytic effect sizes of regional cortical surface area with *p*<0.05: adolescent recurrent MDD patients versus controls.

**Supplemental Figure S20:** Forest plots of meta-analytic effect sizes of regional cortical surface area with *p*<0.05: adolescent MDD patients taking antidepressants at time of scanning versus controls.

**Supplemental Figure S21:** Forest plots of meta-analytic effect sizes of regional cortical surface area with *p*<0.05: adolescent MDD patients taking antidepressants versus adolescent MDD patients not taking antidepressants at time of scanning.

- **Post-hoc moderator analysis**

**Supplemental Figure S22:** Scatterplot showing the mean age of patients in the adolescent-onset adult MDD group versus the effect size for the medial OFC for the adolescent-onset MDD patients versus controls comparison.


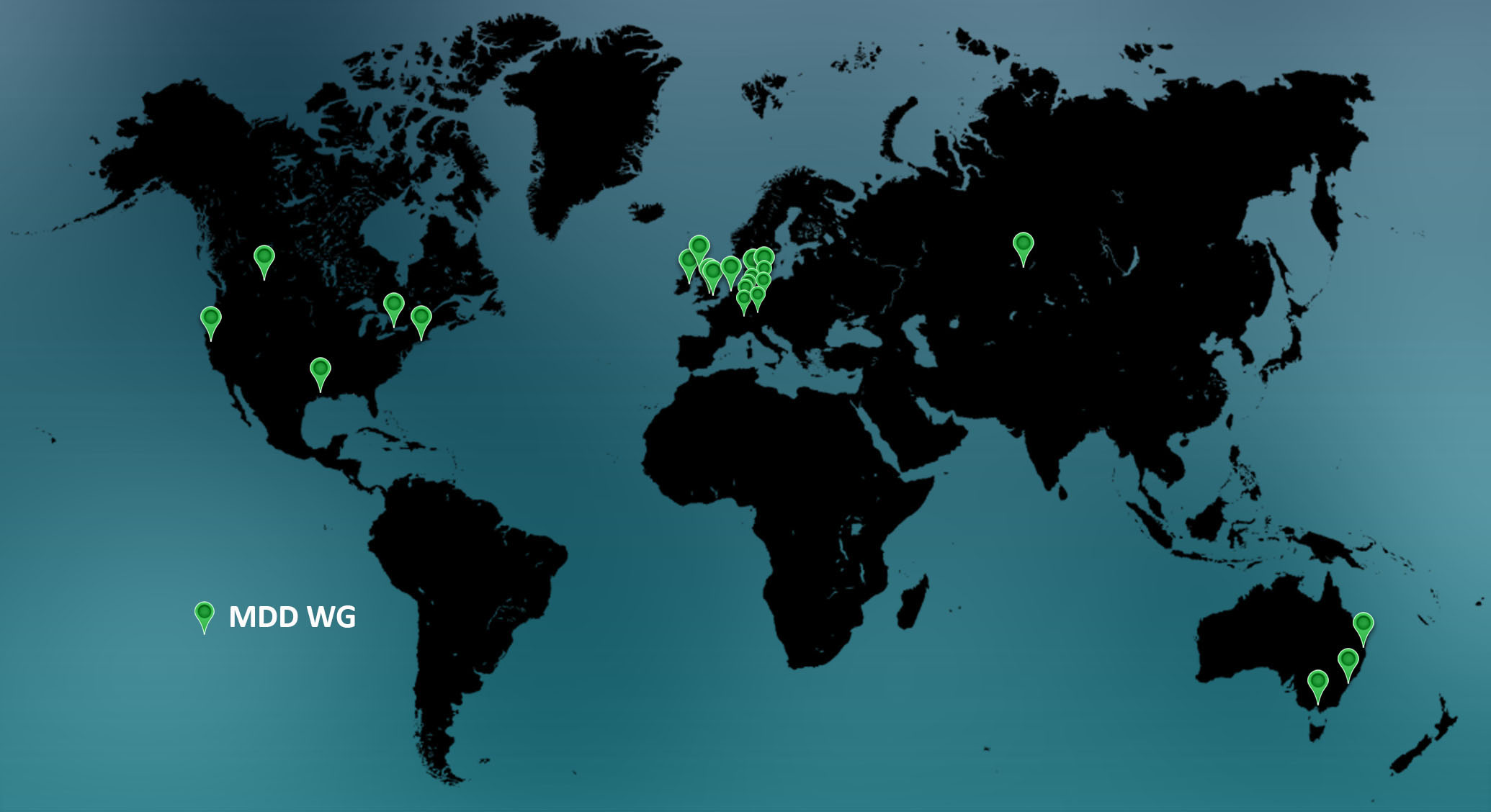


**Supplemental Figure S1**: Overview of research institutes participating in the ENIGMA-Major Depressive Disorder Working Group, displayed on a world map.

**Adult effect size figures:**

**
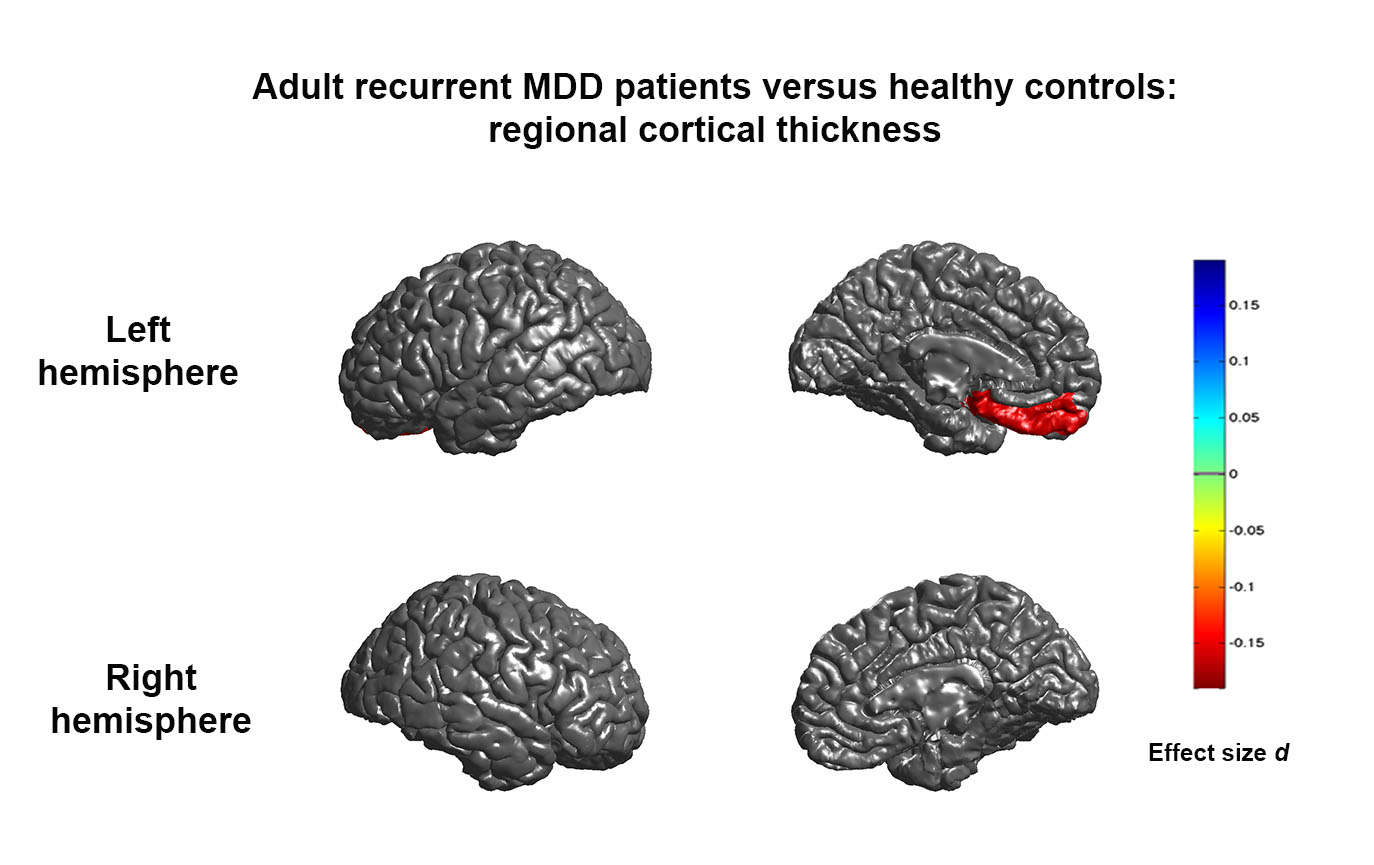
**

**Supplemental Figure S2:** Meta-analysis effect sizes for regions that showed a significant (P<0.05) difference in cortical thickness between adult recurrent MDD patients and healthy controls. Negative effect sizes *d* indicate cortical thinning in MDD compared to controls.


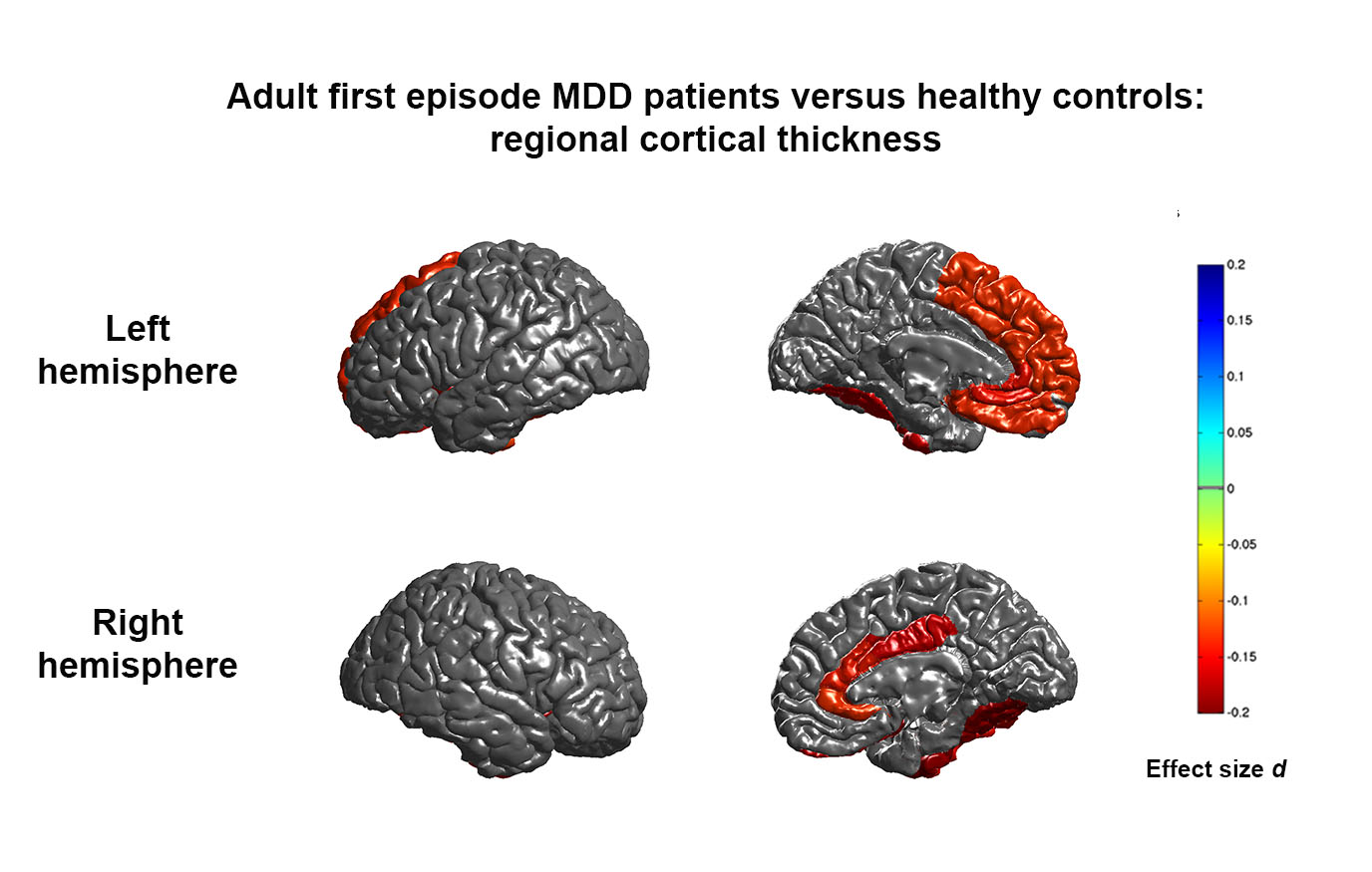


**Supplemental Figure S3:** Meta-analysis effect sizes for regions that showed a significant (P<0.05) difference in cortical thickness between adult first episode MDD patients and healthy controls. Negative effect sizes d indicate cortical thinning in MDD compared to controls.


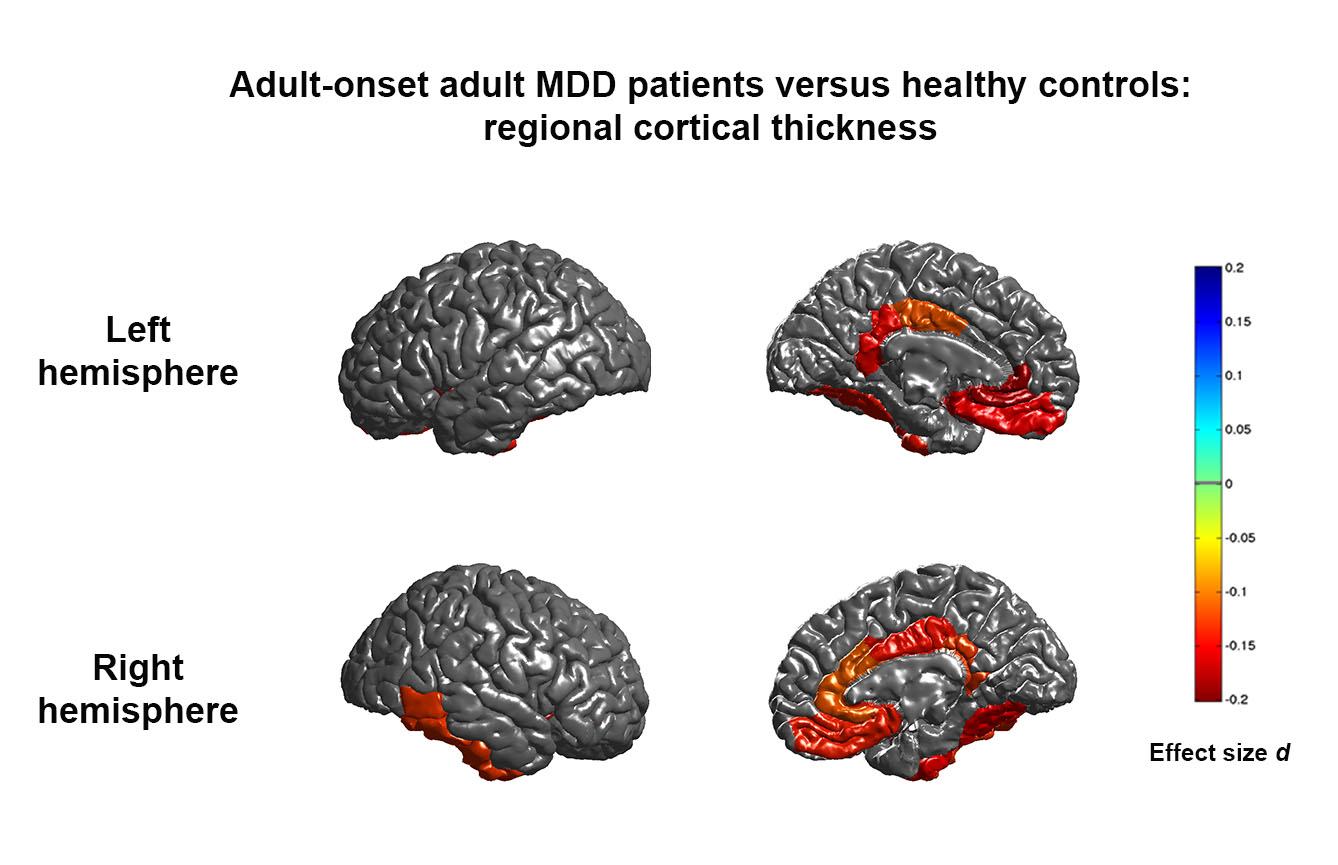


**Supplemental Figure S4:** Meta-analysis effect sizes for regions that showed a significant (P<0.05) difference in cortical thickness between adult age of onset adult MDD patients and healthy controls. Negative effect sizes *d* indicate cortical thinning in MDD compared to controls.


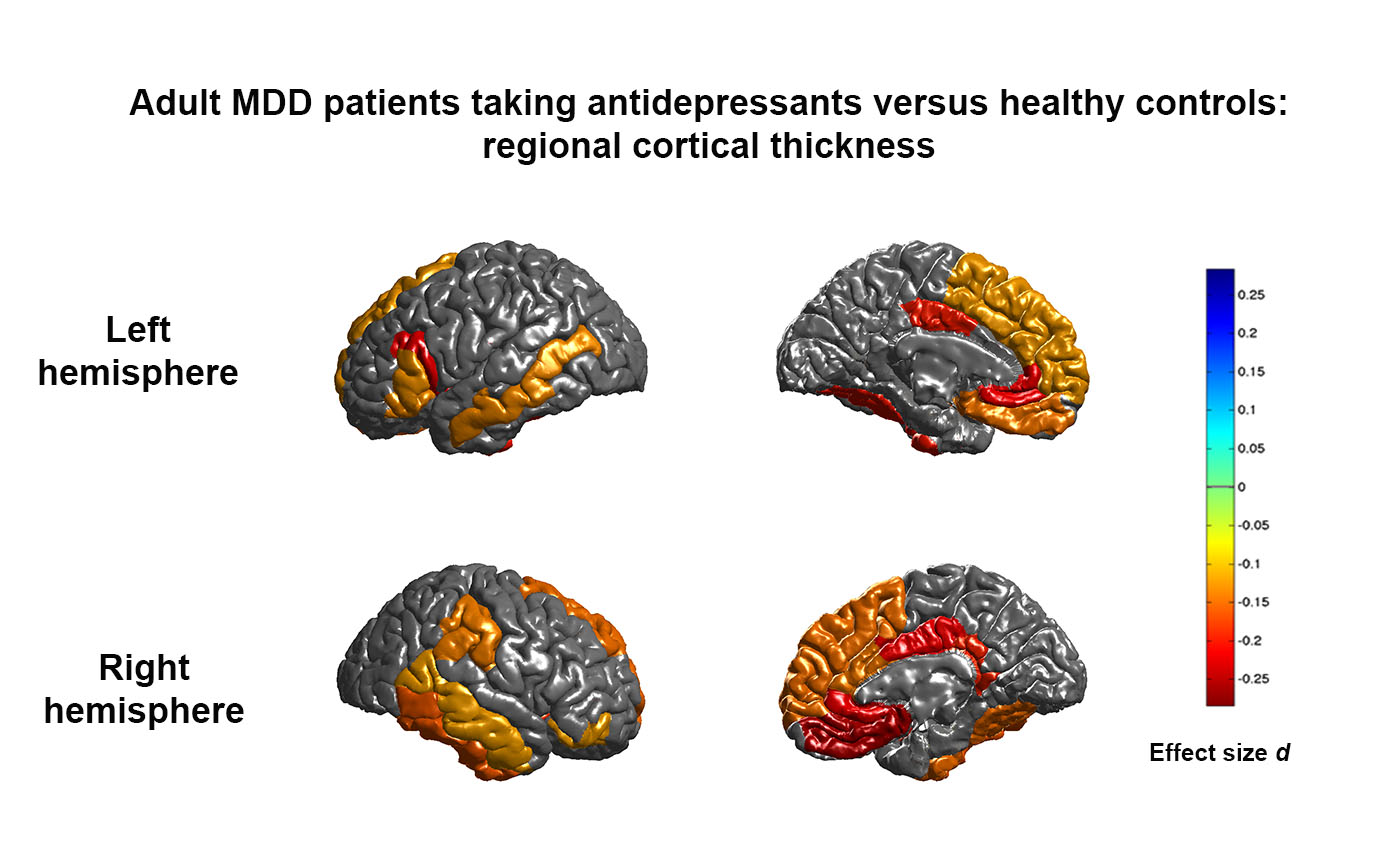


**Supplemental Figure S5:** Meta-analysis effect sizes for regions that showed a significant (P<0.05) difference in cortical thickness between adult MDD patients taking antidepressants at time of scanning and healthy controls. Negative effect sizes *d* indicate cortical thinning in MDD compared to controls.

**
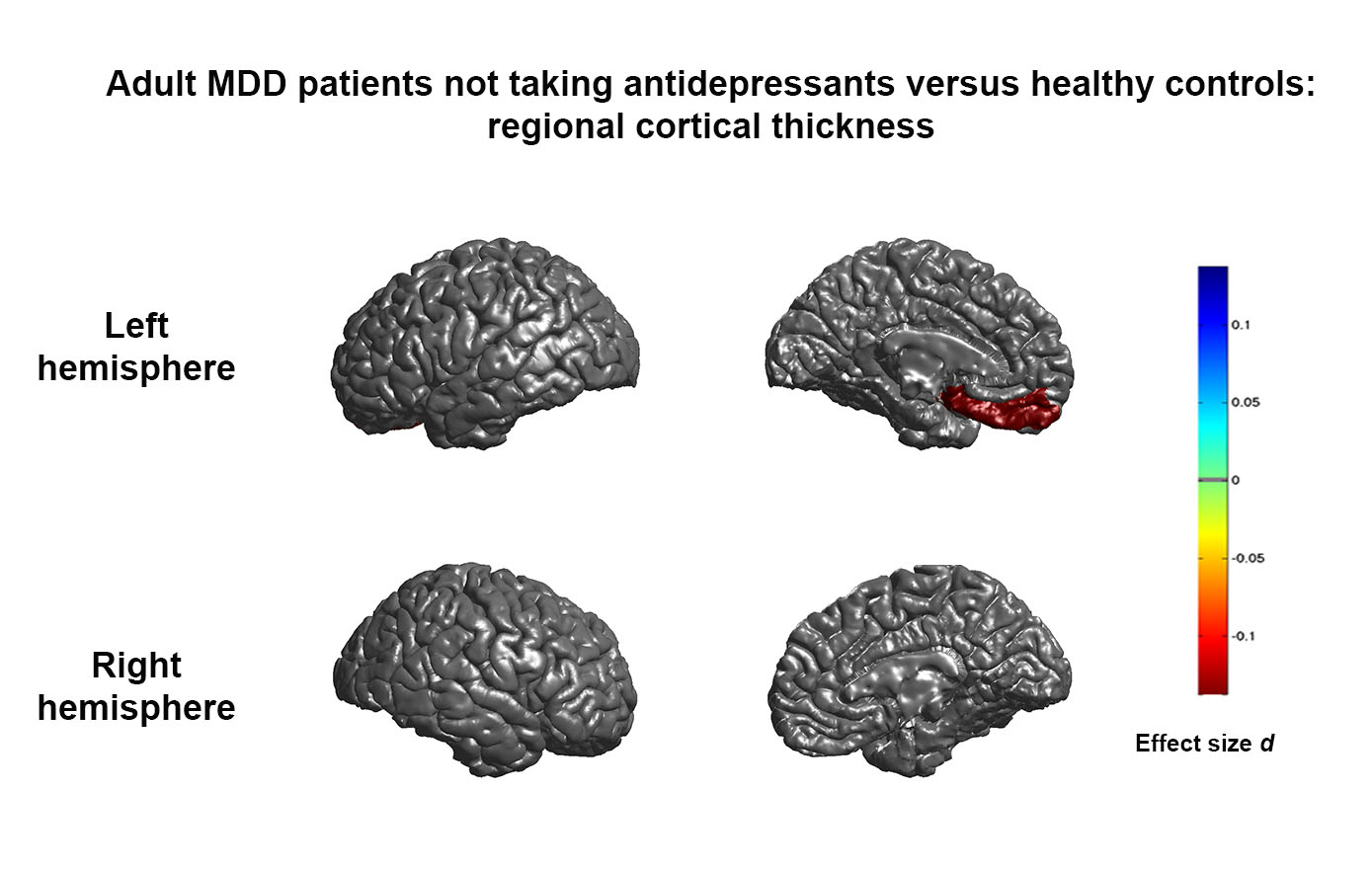
**

**Supplemental Figure S6:** Meta-analysis effect sizes for regions that showed a significant (P<0.05) difference in cortical thickness between adult MDD patients not taking antidepressants at time of scanning and healthy controls. Negative effect sizes *d* indicate cortical thinning in MDD compared to controls.


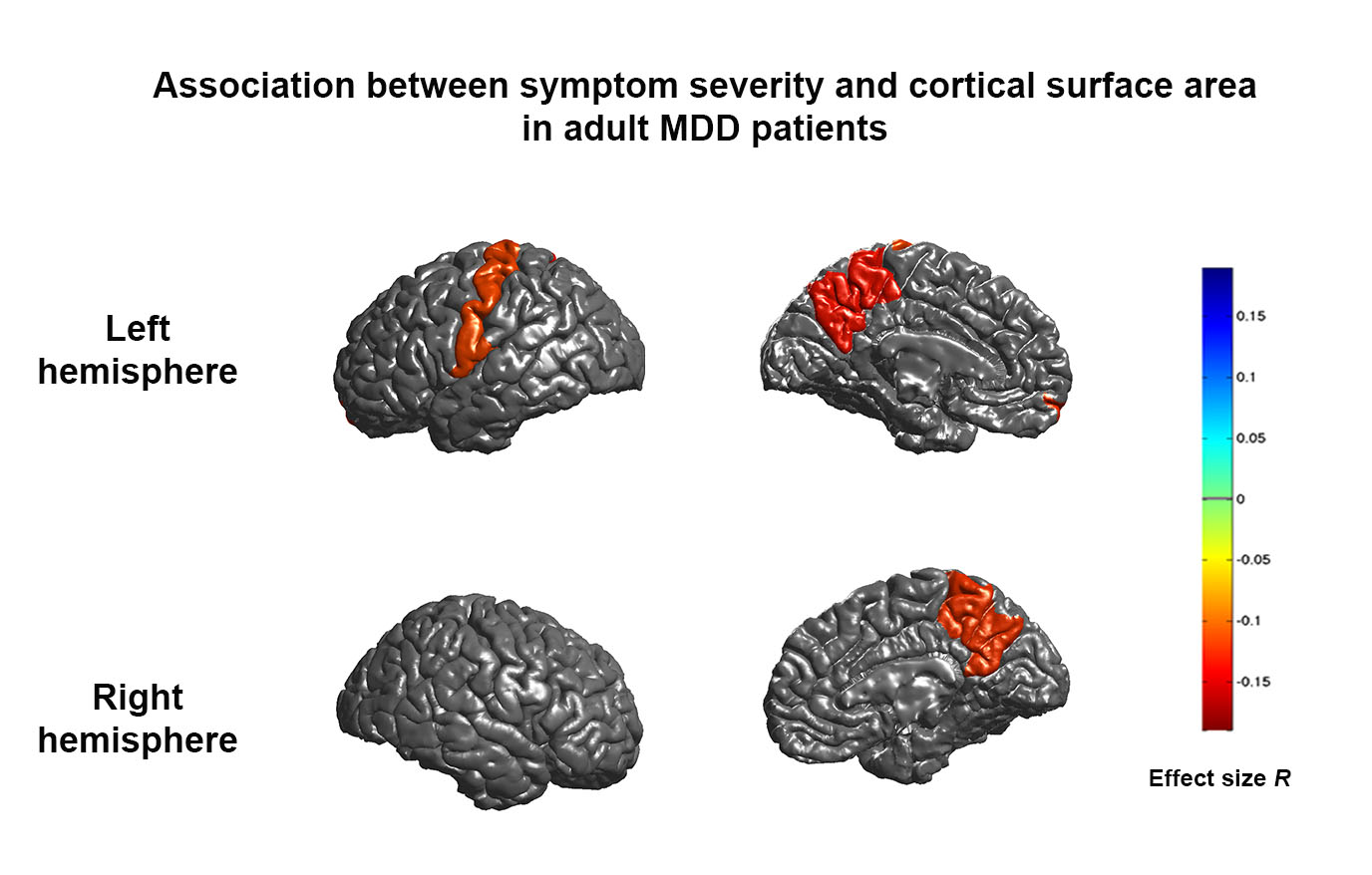


**Supplemental Figure S7:** Meta-analysis effect sizes for regions that showed a significant (P<0.05) association of cortical surface area and symptom severity at study inclusion measured by the BDI. Negative effect sizes *R* indicate negative association between BDI scores and cortical surface area.

**Adolescent effect size figures:**

**
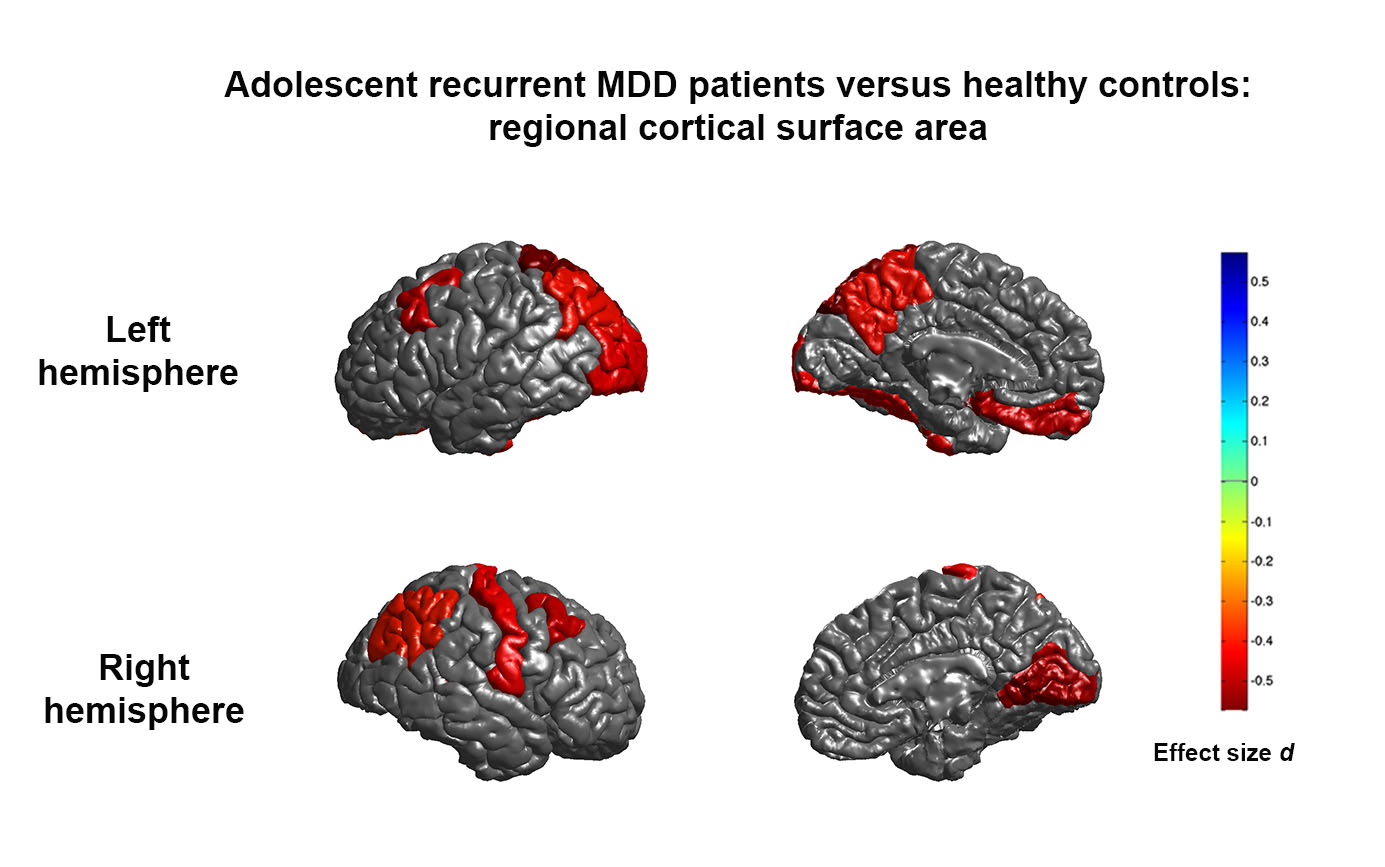
**

**Supplemental Figure S8:** Meta-analysis effect sizes for regions that showed a significant (P<0.05) difference in cortical surface area between adolescent recurrent MDD patients and healthy controls. Negative effect sizes *d* indicate reduced cortical surface area in MDD compared to controls.

**
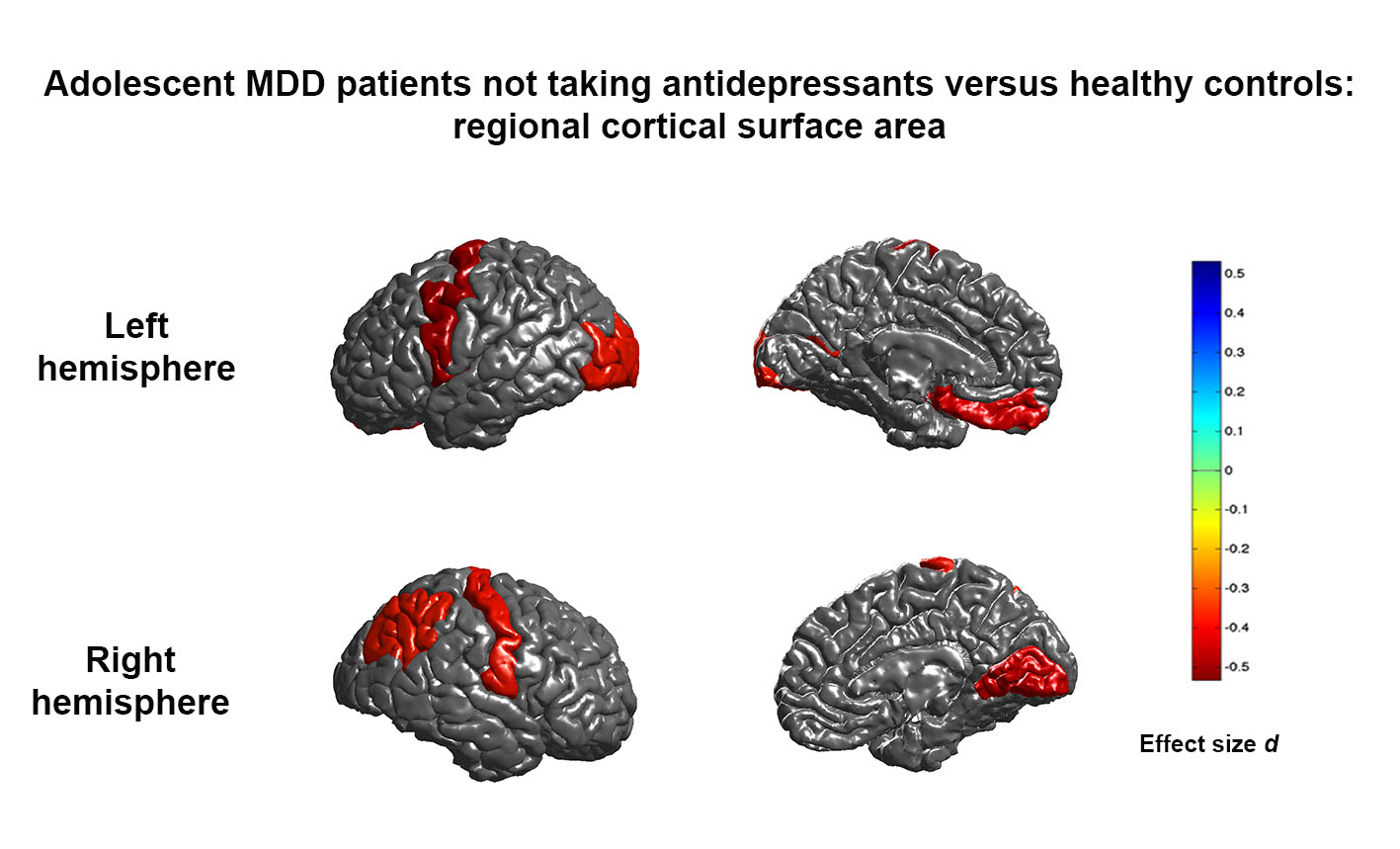
**

**Supplemental Figure S9:** Meta-analysis effect sizes for regions that showed a significant (P<0.05) difference in cortical surface area between adolescent antidepressant not taking MDD patients at time of scanning and healthy controls. Negative effect sizes *d* indicate reduced cortical surface in MDD compared to controls.

**
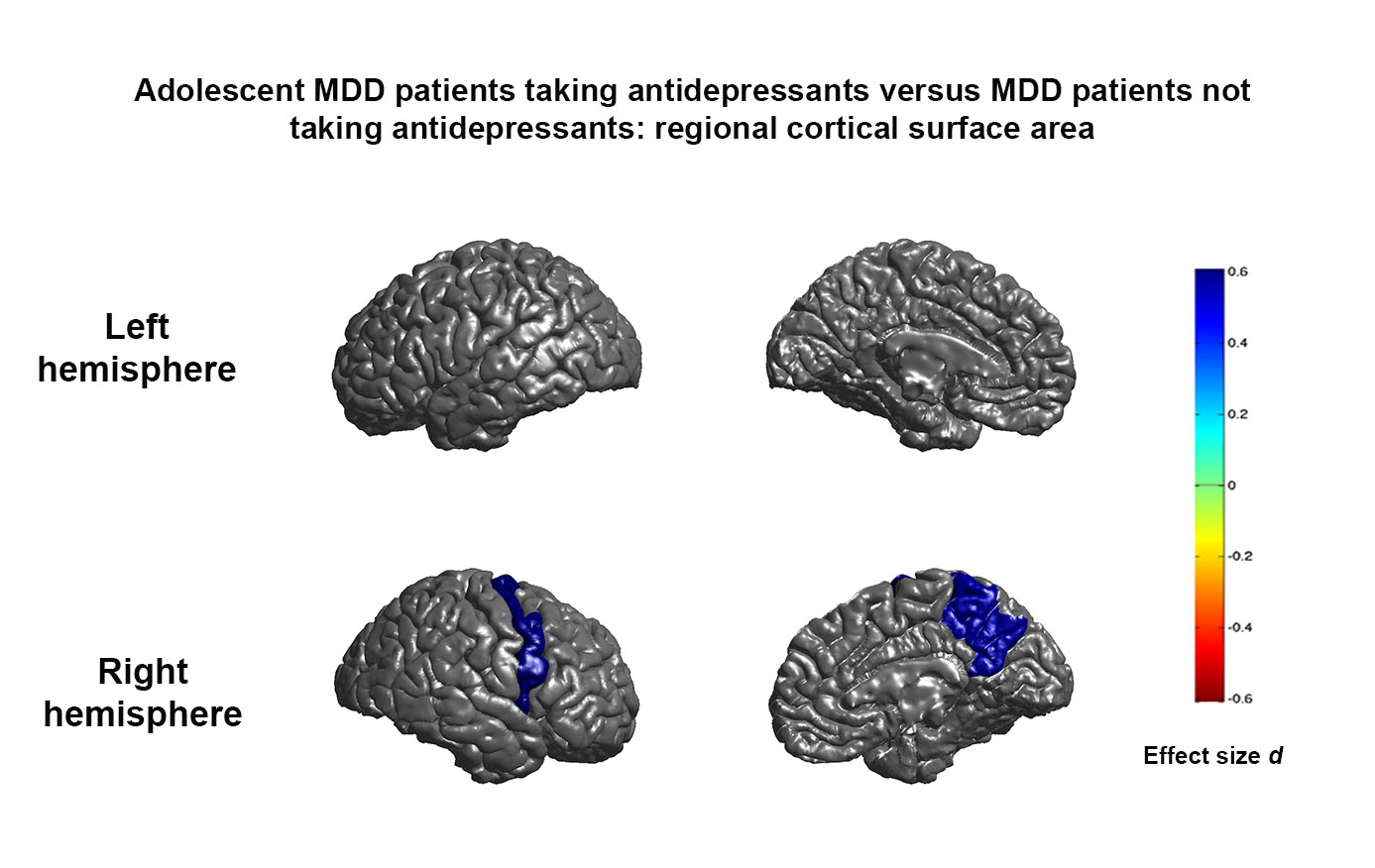
**

**Supplemental Figure S10:** Meta-analysis effect sizes for regions that showed a significant (P<0.05) difference in cortical thickness between adolescent MDD patients taking antidepressants and adolescent MDD patients not taking antidepressants at time of scanning. Positive effect sizes *d* indicate larger cortical surface in adolescent MDD patients taking antidepressants compared to adolescent MDD patients not taking antidepressants.

**Adult forest plots:**

**Adult MDD patients versus healthy controls: cortical thickness**

**Supplemental Figure S11:** Forest plots of meta-analytic effect sizes of regional cortical thickness with *p*<0.05: adult MDD patients versus controls.

OFC: orbitofrontal cortex; MTG: middle temporal gyrus; PCC: posterior cingulate cortex; ACC: anterior cingulate cortex; ITG: inferior temporal gyrus.

**Adult recurrent MDD patients versus healthy controls: cortical thickness**

**Supplemental Figure S12:** Forest plots of meta-analytic effect sizes of regional cortical thickness with *p*<0.05: adult recurrent MDD patients versus controls.

OFC: orbitofrontal cortex.

**Adult first episode MDD patients versus healthy controls: cortical thickness**

**Supplemental Figure S13:** Forest plots of meta-analytic effect sizes of regional cortical thickness with *p*<0.05: adult first episode MDD patients versus controls.

OFC: orbitofrontal cortex; PCC: posterior cingulate cortex; ACC: anterior cingulate cortex; SFG: superior frontal gyrus.

**Adult-onset adult MDD patients versus healthy controls: cortical thickness**

**Supplemental Figure S14:** Forest plots of meta-analytic effect sizes of regional cortical thickness with *p*<0.05: adult-onset adult MDD patients versus controls.

OFC: orbitofrontal cortex; PCC: posterior cingulate cortex; ACC: anterior cingulate cortex; ITG: inferior temporal gyrus.

**Adult MDD patients taking antidepressants versus healthy controls: cortical thickness**

**Supplemental Figure S15:** Forest plots of meta-analytic effect sizes of regional cortical thickness with *p*<0.05: adult MDD patients taking antidepressants at time of scanning versus controls. OFC: orbitofrontal cortex; MTG: middle temporal gyrus; PCC: posterior cingulate cortex; ACC: anterior cingulate cortex; SFG: superior frontal gyrus; bankssts: banks of superior temporal sulcus; ITG: inferior temporal gyrus.

**Adult MDD patients not taking antidepressants versus healthy controls: cortical thickness**

**Supplemental Figure S16:** Forest plots of meta-analytic effect sizes of regional cortical thickness with *p*<0.05: adult MDD patients not taking antidepressants at time of scanning versus controls. OFC: orbitofrontal gyrus.

**Association symptom severity (BDI) and cortical surface area in adult MDD**

**Supplemental Figure S17:** Forest plots of meta-analytic effect sizes of regional cortical thickness with *p*<0.05: association with symptom severity at study inclusion measured by the BDI.

**Adolescent forest plots:**

**Adolescent MDD patients versus healthy controls: cortical surface area**

**Supplemental Figure S18:** Forest plots of meta-analytic effect sizes of regional cortical surface area with *p*<0.05: adolescent MDD patients versus controls. OFC: orbitofrontal cortex; IPC: inferior parietal cortex; SFG: superior frontal gyrus.

**Adolescent recurrent MDD patients versus healthy controls: cortical surface area**

**Supplemental Figure S19:** Forest plots of meta-analytic effect sizes of regional cortical surface area with *p*<0.05: adolescent recurrent MDD patients versus controls. OFC: orbitofrontal cortex; IPC: inferior parietal cortex; MFG: middle frontal gyrus; SPC: superior parietal cortex; bankssts: banks of superior temporal sulcus.

**Adolescent MDD patients not taking antidepressants versus healthy controls: cortical surface area**

**Supplemental Figure S20:** Forest plots of meta-analytic effect sizes of regional cortical surface area with *p*<0.05: adolescent MDD patients not taking antidepressants at time of scanning versus controls. OFC: orbitofrontal cortex; IPC: inferior parietal cortex.

**Adolescent MDD patients taking antidepressants versus Adolescent MDD patients not taking antidepressants: cortical surface area**

**Supplemental Figure S21:** Forest plots of meta-analytic effect sizes of regional cortical surface area with *p*<0.05: adolescent MDD patients taking antidepressants versus adolescent MDD patients not taking antidepressants at time of scanning.

**Association between mean age of adolescent-onset adult MDD patients and effect size for the medial orbitofrontal cortex (OFC) for each site.**

**
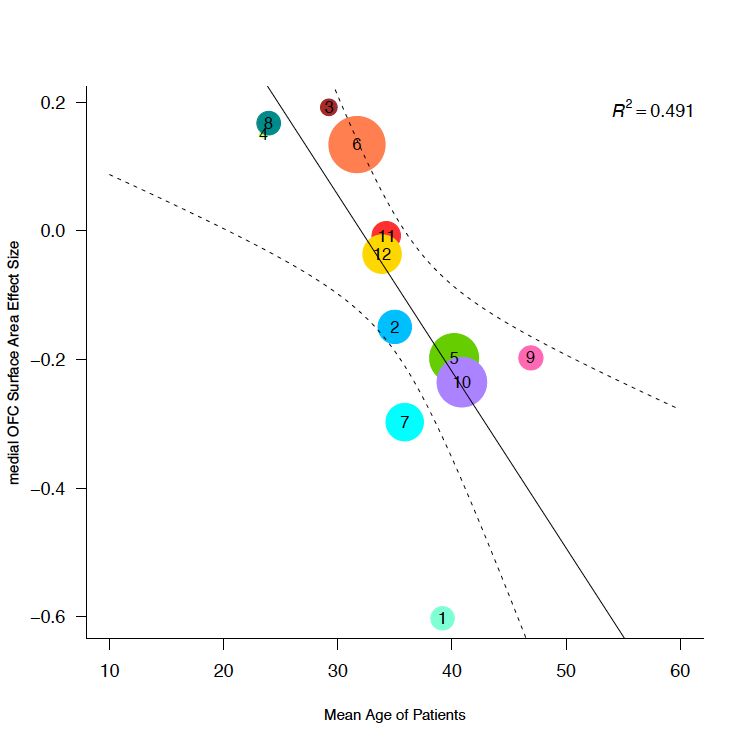
**

**Supplemental Figure S22:** Scatterplot showing the mean age of patients in the adolescent-onset adult MDD group versus the effect size for the medial OFC for the adolescent-onset MDD patients versus controls comparison. Point numbers reflect the studies included in the EAO versus controls meta-analysis: 1) Imaging Genetics Dublin, 2) Houston, 3) MMDP 3T, 4) Melbourne, 5) MPIP, 6) Muenster cohort, 7) NESDA, 8) QTIM, 9) SHIP, 10) SHIP-trend, 11) Stanford, 12) Sydney.

The size of each point corresponds to the inverse of the standard error for the effect size at each site (i.e., sites with larger samples have bigger points). The solid black line represents the effect of the mean age of patients on the effect size (the result of the moderator analysis) weighted by the inverse of the standard error in each sample. A trendwise significant negative association between the mean age of patients and the medial OFC effect size was observed using a FDR significance threshold for comparisons of 70 brain regions, i.e. greater medial OFC thinning in EAO patients relative to controls was observed as the mean age of patients increased.
